# Novel Genomic and Evolutionary Insight of WRKY Transcription Factors in Plant Lineage

**DOI:** 10.1038/srep37309

**Published:** 2016-11-17

**Authors:** Tapan Kumar Mohanta, Yong-Hwan Park, Hanhong Bae

**Affiliations:** 1 Free Major of Natural Sciences, College of Basic Studies, Yeungnam University, Gyeongsan, 38541, Republic of Korea; 2 School of Biotechnology, Yeungnam University, Gyeongsan, 38541, Republic of Korea

## Abstract

The evolutionarily conserved WRKY transcription factor (TF) regulates different aspects of gene expression in plants, and modulates growth, development, as well as biotic and abiotic stress responses. Therefore, understanding the details regarding WRKY TFs is very important. In this study, large-scale genomic analyses of the WRKY TF gene family from 43 plant species were conducted. The results of our study revealed that WRKY TFs could be grouped and specifically classified as those belonging to the monocot or dicot plant lineage. In this study, we identified several novel WRKY TFs. To our knowledge, this is the first report on a revised grouping system of the WRKY TF gene family in plants. The different forms of novel chimeric forms of WRKY TFs in the plant genome might play a crucial role in their evolution. Tissue-specific gene expression analyses in *Glycine max* and *Phaseolus vulgaris* showed that *WRKY11-1*, *WRKY11-2* and *WRKY11-3* were ubiquitously expressed in all tissue types, and *WRKY15-2* was highly expressed in the stem, root, nodule and pod tissues in *G. max* and *P. vulgaris*.

Plants are continuously subjected to biotic and abiotic stresses throughout their life cycle. Hence, they have developed an evolutionarily complex series of signaling mechanisms to perceive and respond to different signals via different signaling pathways. Transcriptional regulation plays remarkable roles in response of different signaling events. It has progressed from ancient life forms to advanced life forms and is inseparably connected through developmental progression. Such transcriptional progression mechanisms are regulated by different types of transcriptional machinery commonly known as transcription factors (TFs). The TFs possess the ability to activate or repress the expression of target genes responsible for the regulation of different signaling cascades^1^,^2^,^3^. The WRKY TF is one such TF found in plants. WRKY TFs are characterized by the presence of a unique WRKY domain of approximately 60 amino acid residues^3^,^4^,^5^. The domain contains a highly conserved WRKYGQK heptapeptide amino acid sequence and conserved C_2_H_2_ or C_2_HC zinc finger motif. The conserved WRKY domain plays a crucial role by binding to the W-box DNA motif TTGACC/T of the target gene^3^,^5^,^6^. Almost all WRKY TFs identified thus far preferentially binds to a specific core DNA sequence^7^. In addition to binding to the W-box DNA motif, some WRKY TFs also bind to other sites. For example, *Oryza sativa* OSWRKY13 binds to PRE4 (pathogen-responsive element; TGCGCTT), and *Hordeum vulgare* HvWRKY46 binds to SURE (sugar-responsive element) (TAAAGATTACTAATAGGAA)^8^,^9^. The binding of a WRKY TF to the W-box and other elements leads to synergistic transcriptional activation in plants^10^. In addition to this process, the conserved WRKY amino acid sequences are occasionally replaced by WRRY, WSKY, WKRY, WVKY or WKKY domains^11^.

The model plant *Arabidopsis thaliana* encodes 74 WRKY TFs in its genome. Based on the similarity in sequence and phylogenetic relationships, WRKY TFs are divided into three groups (I, II, and III); group II is further divided into several sub-groups (e.g IIa, IIb, IIc, IId, IIe, IIf, and IIg)^4^,^12^. There are two different types of WRKY TFs: (1) contains a single WRKY domain at the C-terminal end, (2) the other contain two WRKY domains, one at the N-terminal and other at the C-terminal end. The WRKY proteins that contain a single WRKY domain fall in group II and III while the WRKY protein that contains double WRKY domain (N- and C-terminals) are fall in group I^4^,^12^. The WRKY proteins that contain two WRKY domains are functionally redundant^13^. The N-terminal WRKY domain increases the affinity and specificity to bind the target gene, whereas the C-terminal WRKY domain constitutes the major DNA-binding domain^4^,^14^,^15^,^16^. The single WRKY domain-containing WRKY TFs (groups II and III) are considerably more similar in sequence to the C-terminal WRKY domain rather than to the N-terminal domain of group I WRKY TFs. These findings suggest that the C-terminal WRKY domain of group I WRKY TFs and the single WRKY domain of groups II and III WRKY TFs are functionally commensurate, and share the major DNA-binding domain^4^.

The WRKY TFs have been reported to play important roles in cellular and physiological processes, including seed germination^17^,^18^, root development^19^, plant growth^20^, seed development^21^,^22^,^23^ and senescence^24^,^25^,^26^. Furthermore, they are involved in diverse responses to biotic stress caused by insect herbivores^27^,^28^, bacterial pathogens^29^,^30^, fungi^31^ and viruses^32^. They respond to different signaling molecules such as indole-3-acetic acid^19^, jasmonic acid^33^, salicylic acid^34^, abscisic acid^35^,^36^, and gibberellic acid^37^. In addition, WRKY TFs respond to different abiotic stresses^38^ such as UV radiation^39^, high and low temperatures^40^,^41^, H_2_O_2_^42^,^43^, and salt and drought stresses^44^,^45^. Therefore, understanding the basic biology and genomics of WRKY TFs in plants is very important.

Numerous studies have been conducted with WRKY TFs in different plant species, including *Arabidopsis thaliana*^4^, *Brachypodium distachyon*^14^, *Gossypium raimondii*^46^, *Lotus japonicas*^47^, *Oryza sativa*^48^, *Riccinus communis*^49^, *Setaria italica*^50^, *Solanum lycopersicum*^51^, *Triticum aestivum*^52^, and *Vitis vinifera*^53^. Different research groups have provided different grouping systems for the WRKY TFs, leading to lack of consistency in the grouping system. Thus, it was highly important to formulate a new and clear grouping system for all WRKY TFs of the plant kingdom identified so far. Xi *et al.*^11^ reported about the presence of a deduced WRKY domain^11^. Therefore, we were also very interested in determining whether WRKY TFs possess any additional novel, modified WRKY domains in its genome. Rinerson *et al.*^54^ reported the presence of chimeric WRKY TFs that contain combinations of novel protein domains and WRKY TF domains as well^54^. Hence, it was also very interesting to elucidate more details about these chimeric proteins. Genome sequencing data from different plant species are currently increasing rapidly that has provided an excellent platform for better understanding the WRKY TF gene family. Therefore, we conducted genome-wide identification of the WRKY TF gene family from 43 plant species and analysed their genomic, phylogenetic, and other basic characteristics to decipher their novel genomic constitution.

## Results

### Identification of WRKY TFs

Genome-wide identification of WRKY TF gene family members was performed using 43 plant species across the evolutionary lineage of the plant kingdom (Table 1). These plant species included a wide mixture of dicots (27), monocots (7), algae (5), bryophytes (1), pteridophytes (1), gymnosperms (1) and amoebae (1). In total, 3035 WRKY TFs were identified from these species. Of the studied species, the monocot plant *Panicum virgatum* encoded the maximum number of WRKY TFs (167), whereas, the green algae *Chlamydomonas reinhardtii* and *Coccomyxa subellipsoidea* encoded the minimum (only one). Among dicots, *Brassica rapa* and *Glycine max* encoded 145 WRKY TFs, whereas the amoeba *Dictyostelium purpureum* encoded nine. The WRKY TFs of the algae *C. reinhardtii*, *C. subellipsoidea*, and *M. pusilla* contained only a single WRKY domain (C-terminal WRKY domain) whereas *O. lucimarinus* and *V. carteri* contain both single and double WRKY domains. The WRKY TF gene family of the amoeba *D. purpureum* contained both single (C-terminal) and double (N- and C-terminals) WRKY domains.

### Genomics of WRKY TFs

The transcript organization of WRKY TFs has been shown to be highly variable in nature. *F. vesca* FvWRKY70–7 contains the largest transcript, encoding an open reading frame (ORF) of 5949 nucleotides (1982 amino acids). Similarly, the *M. domestica* MdWRKY61-2 encodes the smallest WRKY TF containing only 135 nucleotides (44 amino acids). The intron organization of WRKY TFs is very dynamic, ranging from zero to twenty introns per gene. The number of plant WRKY TFs that contain various numbers of introns is as follows: zero (46), one (338), two (1440), three (488), four (375), five (223), six (61), seven (20), eight (5), nine (9), ten (12), eleven (4), twelve (3), thirteen (3), fourteen (0), fifteen (2), sixteen (1), seventeen (0), eighteen (2), nineteen (0), and twenty (2).

### Novel WRKY TFs

**Figure 1:**
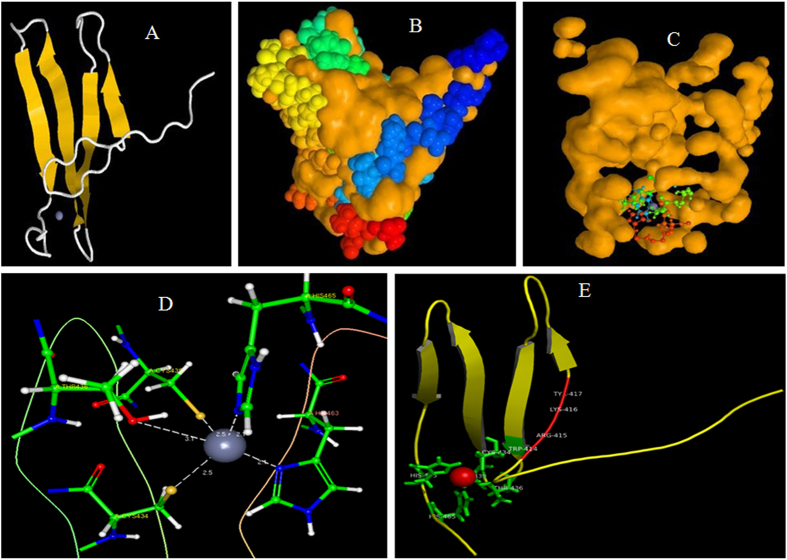
The schematic representation of the secondary and tertiary structures of WRKY TFs. (**A**) General secondary structure of the WRKY TF with the Zn ligand, (**B**) space fill model of a WRKY TF showing the Zn ligand in red and WRKY domain in blue, (**C**) position of a Zn ligand in the cavity of WRKY TF (**D**) hydrogen bonding of Zn ligand with WRKY TF, (**E**) secondary structure of a WRKY TF showing the position of WRKY domain and hydrogen bonding of the Zn ligand. The molecular structure of WRKY TF was predicted by using the GENO3D server using AtWRKY1 as query search.

**Figure 2:**
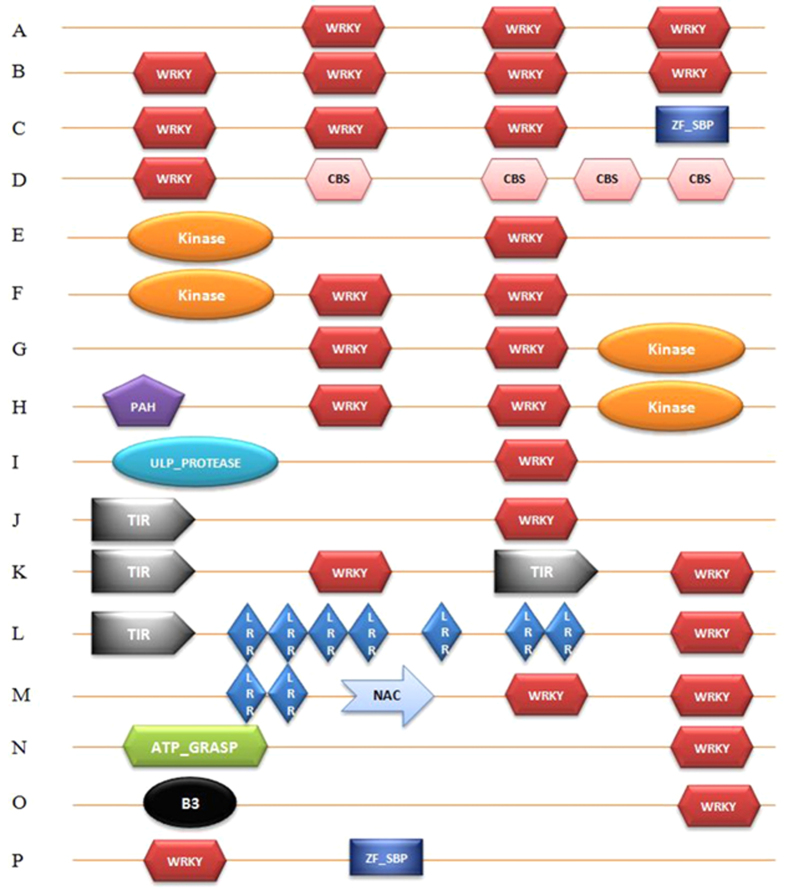
Novel WRKY TFs of plants. In addition to the presence of classic WRKY TFs in plants, the present study revealed the presence of novel WRKY TFs. These novel- WRKY TFs are as follows: (**A**) WRKY TFs with three WRKY domains (GrWRKY12, GrWRKY21-5, LuWRKY3-7), (**B**) WRKY TFs with four WRKY domains (AcWRKY1, SlWRKY4-2), (**C**) WRKY TFs with three WRKY domains followed by a ZF_SBP TF domain (LuWRKY3-5, LuWRKY3-6), (**D**) WRKY domain followed by three calcium binding CBS domains (BrWRKY36-2), (**E**) kinase domain followed by one WRKY domain (FvWRKY59), (**F**) kinase domain followed by two WRKY domains (PhWRKY59), (**G**) two WRKY domains followed by a kinase domain (BrWRKY58-1, BrWRKY58-2), (**H**) PAH domain followed by two WRKY domain and kinase domain (AtWRKY19), (**I**) protease domain followed by a WRKY domain (OsWRKY57, PvWRKY57-1, SbWRKY57), (**J**) TIR domain followed by WRKY domain (FvWRKY52, GmWRKY55-3), (**K**) TIR domain followed by a WRKY domain twice (FvWRKY70-7), (**L**) TIR domain followed by a LRR domain and a WRKY domain (FvWRKY16), (**M**) LRR and NAC domain followed by two WRKY domains (SbWRKY59), (**N**) ATP_GRASP domain followed by a WRKY domain (AlWRKY16), (**O**) B3 domain followed by a WRKY domain (PvWRKY94-1), and (**P**) WRKY domain followed by a ZF_SBP domain (SiWRKY59-2).

In general, WRKY TFs are characterized by the presence of either one (Fig. 1) or two WRKY domains. In this study, we identified 16 chimeric forms of WRKY TFs in plants (Fig. 2). In addition, we identified different WRKY TFs that contain three (GrWRKY12, GrWRKY21-5, and LuWRKY3-7) (Fig. 2-A); and four (AcWRKY1, SlWRKY4-2) (Fig. 2-B) WRKY domains; three WRKY domains with the ZF_SBP TF domain (LuWRKY3–5, LuWRKY3–6) (Fig. 2-C); a single WRKY domain with three CBS domains (BrWRKY36-2) (Fig. 2-D); a kinase domain followed by a single WRKY domain (FvWRKY59) (Fig. 2-E); a kinase domain followed by two WRKY domains (PhWRKY59) (Fig. 2-F); two WRKY domains followed by a kinase domain (BrWRKY58-1, BrWRKY58-2) (Fig. 2-G); a PAH domain followed by two WRKY domains and one kinase domain (AtWRKY19) (Fig. 2-H); an ULP_protease domain followed by a WRKY domain (OsWRKY57, PvWRKY57-1, and SbWRKY57) (Fig. 2-I); a TIR domain followed by a WRKY domain (FvWRKY52, GmWRKY55-3) (Fig. 2-J); a TIR domain followed by two WRKY domains (FvWRKY70-7) (Fig. 2-K); a TIR domain followed by seven LRR domains and a WRKY domain (FvWRKY16) (Fig. 2-L); two LRR domains followed by an NAC domain and two WRKY domains (SbWRKY59) (Fig. 2-M); an ATP_GRASP domain followed by a WRKY domain (AlWRKY16) (Fig. 2-N); a B3 domain followed by a WRKY domain (PvWRKY94-1) (Fig. 2-O); and a WRKY domain followed by a ZF_SBP domain (Fig. 2-P).

### Conserved domains of WRKY TFs

**Figure 3:**
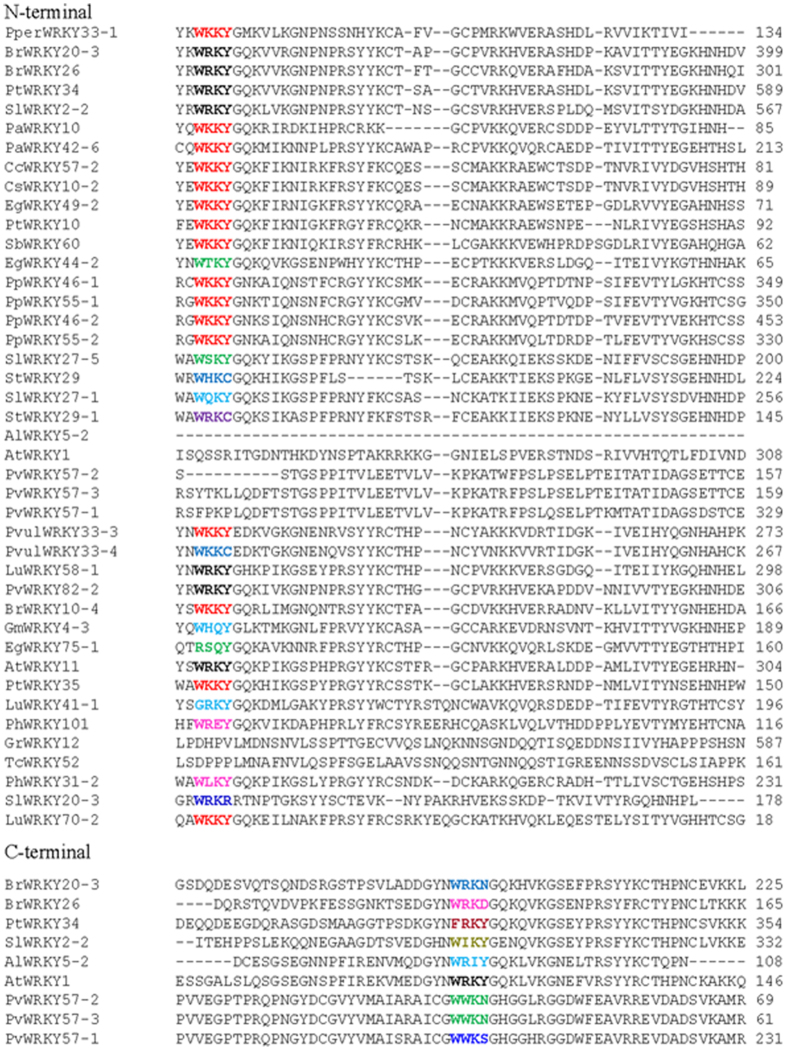
Substitute WRKY domain of plants. Different novel substitutes of WRKY domains were found in the N- and C-terminal regions of WRKY TFs. The conserved WRKY amino acids were replaced by different types of amino acids. The N- and C-terminal WRKY domains of *A. thaliana* AtWRKY were aligned with these novel substitutes of WRKY domains. This indicates that WRKY amino acids have been replaced by these novel amino acids. Multiple sequence alignment of WRKY TF was performed using multalin software (http://multalin.toulouse.inra.fr/multalin/) by using the protein weight matrix BLOSUM62.

To understand the conserved domains of WRKY TFs, multiple sequence alignments of single (C-terminal domain) and double WRKY domain (both N- and C-terminal domain) proteins were analyzed separately. The single WRKY domain (C-terminal)-containing proteins included the conserved W-R-K-Y-G-Q-K, P-R-x-Y-Y-x-C-x_5_-C, K-x-V, and H-x-H domains as well as several conserved amino acid residues (Supplementary Figure 1). The N- terminal region of double WRKY domain proteins contain conserved D-G-Y-N-W-R-K-Y-G-Q-K and R-S-Y-Y-x-C-x_4_-C-x_22_-H-x-H domains. The C-terminal region of the double WRKY domain protein contains conserved D-G-Y-R-W-R-K-Y-G-Q-K, R-S-Y-Y-x-C-x_4_-C, V-R-K-H-V-E, and H-x-H domains (Supplementary Figure 2). In some cases, the conserved WRKY amino acids in the WRKY domain were replaced with some other amino acids including W-K-K-Y (BrWRKY10-4, CcWRKY57-2, CsWRKY10-2, EgWRKY49-2, LuWRKY70-2, PvulWRKY33-3, PvulWRKY33-4, PpWRKY46-1, PpWRKY46-2, PpWRKY55-1, PpWRKY52-2, PaWRKY10, PaWRKY42-6, PtWRKY10, PtWRKY35, PperWRKY33-1 and SbWRKY60), W-R-I-Y (AlWRKY5-2), W-R-K-N (BrWRKY20-3), W-R-K-D (BrWRKY26), W-H-Q-Y (GmWRKY4-3), W-R-I-S (GrWRKY12), W-R-Q-V (LuWRKY58-1), G-R-K-Y (LuWRKY41-1), W-L-K-Y (PhWRKY31-2), W-R-E-Y (PhWRKY101), A-R-K-M (PvWRKY57-1, PvWRKY57-2, PvWRKY57-3), W-W-K-N (PvWRKY57-2, PvWRKY57-3), W-R-M-Y (PvWRKY82-2), W-R-K-R (SlWRKY20-3), W-I-K-Y (SlWRKY2-2), W-S-K-Y (SlWRKY27-5), W-Q-K-Y (SlWRKY27-1), W-H-K-C (StWRKY29), W-R-C-I (TcWRKY52), F-R-K-Y (PtWRKY34), R-S-Q-Y (EgWRKY75-1), W-T-K-Y (EgWRKY44-2), W-K-K-C (PvulWRKY33-4) and W-R-K-C (StWRKY29-1) (Fig. 3).

### Phylogeny of WRKY TFs

**Figure 4:**
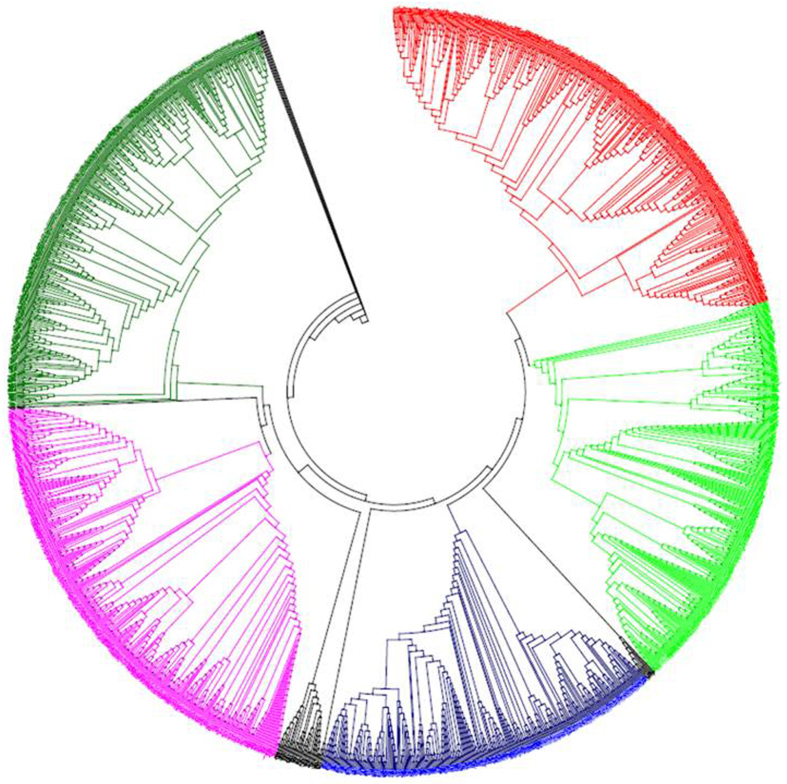
Unrooted phylogenetic tree of WRKY TFs of monocot, dicot, and lower eukaryotic (amoeba, algae, bryophyte, pteridophyte, and gymnosperm) plants. The phylogenetic tree shows eight independent groups. We named them as groups I (red), II (lime), III (black), IV (blue), V (black), VI (pink), VII (green), and VIII (black). To get details about distribution of different WRKY TF in different group, please refer to Supplementary Figure 3. The phylogenetic tree revealed that, the WRKY family members of one group overlapped with another group. The phylogenetic tree was constructed using MEGA6.

**Figure 5:**
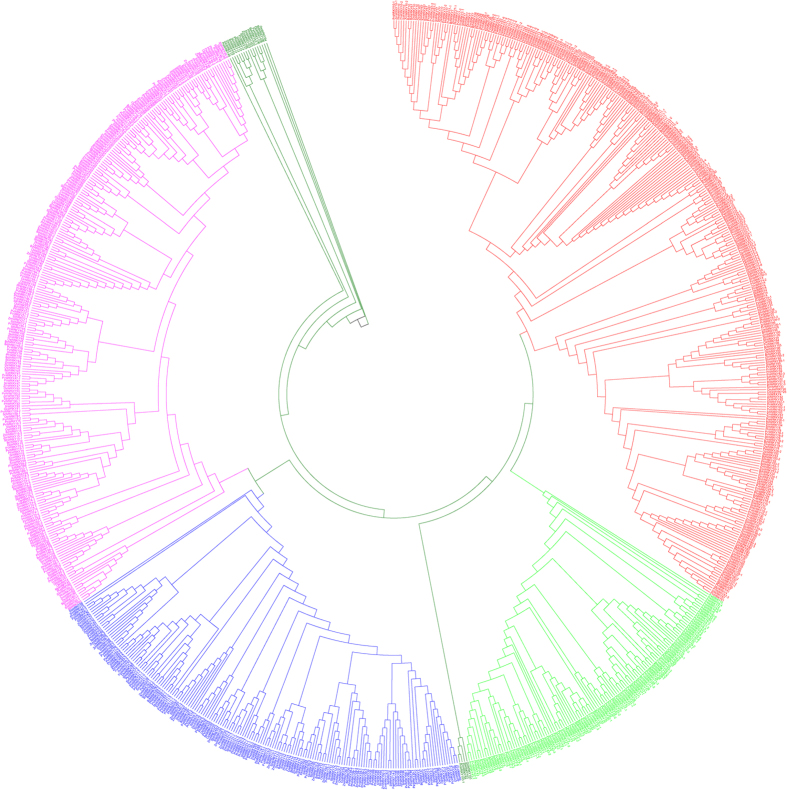
Unrooted phylogenetic tree of WRKY TFs of monocot and lower eukaryotic (amoeba, algae, bryophyte, pteridophyte and gymnosperm) plants. The phylogenetic tree shows six independent phylogenetic groups. We named them as groups I (red), II (lime), III (green), IV (blue), V (pink) and VI (green). The WRKY TF group members are specific to their groups and no WRKY TF members in one group overlap with those in any other group. The phylogenetic tree was constructed using MEGA6.

**Figure 6:**
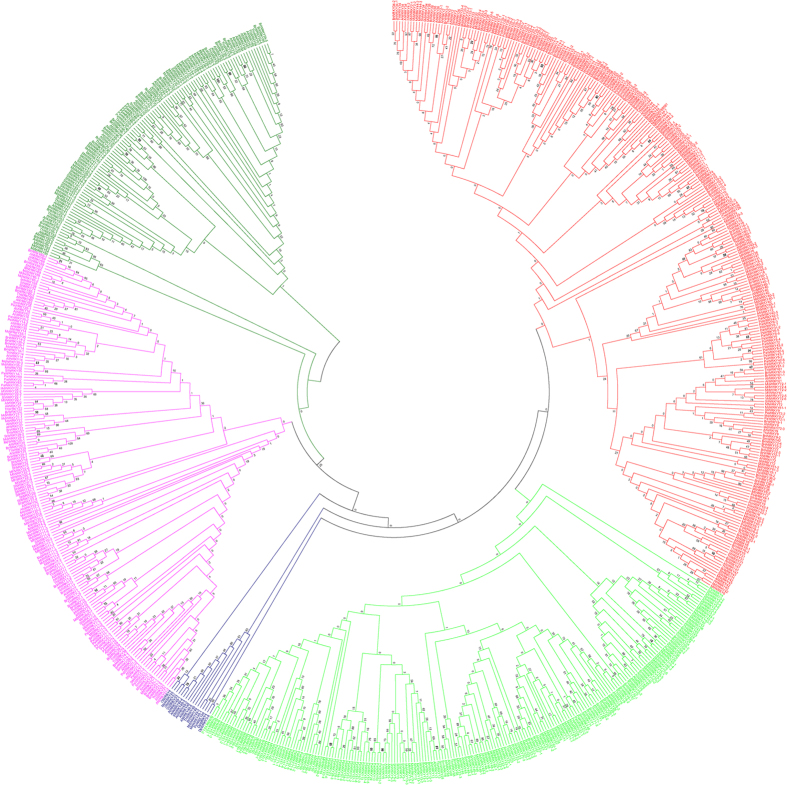
Unrooted phylogenetic tree of WRKY TFs of dicot and lower eukaryotic (amoeba, algae, bryophyte, pteridophyte, and gymnosperm) plants. The phylogenetic tree shows the presence of three phylogenetically distinct groups. We named them as groups I (pink), IIa (red), IIb (lime), IIc (blue), and III (green). The WRKY TF group members of group IIa, IIb and IIc overlap with each other and were hence retained under sub-group of group II. The classification of groups I, II, and III resembled that used in previous studies. The WRKY TF members of groups I and III did not overlap with one another and resembled the grouping system of used in previously published studies. The phylogenetic tree was constructed by using MEGA6.

**Figure 7:**
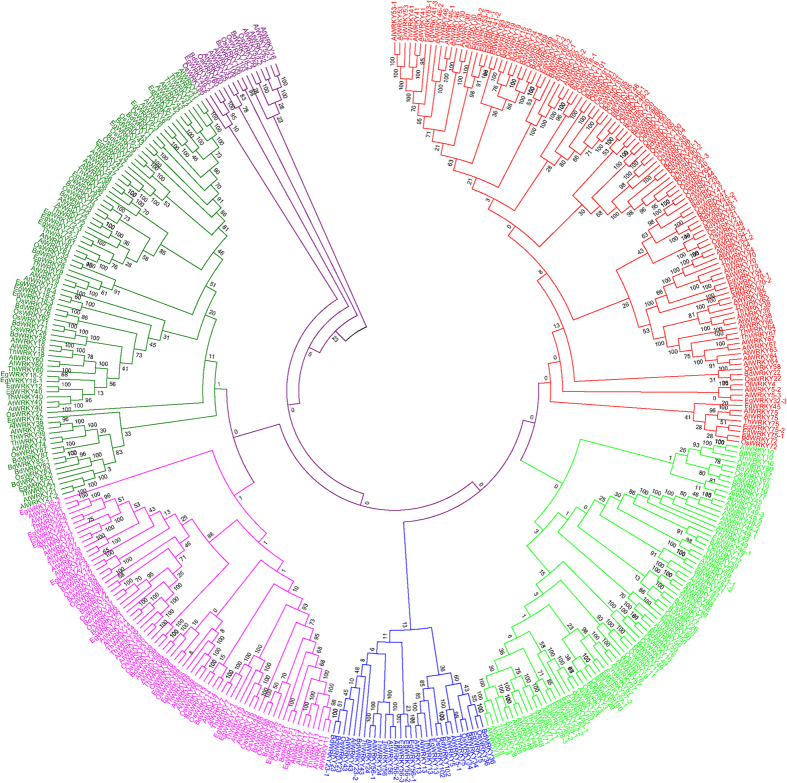
Unrooted phylogenetic tree of C-terminal WRKY domain containing WRKY TFs. The phylogenetic tree shows six phylogenetically independent groups, I (red), II (lime), III (blue), IV (pink), V (green) and VI (purple). The phylogenetic tree was constructed by using MEGA6.

**Figure 8:**
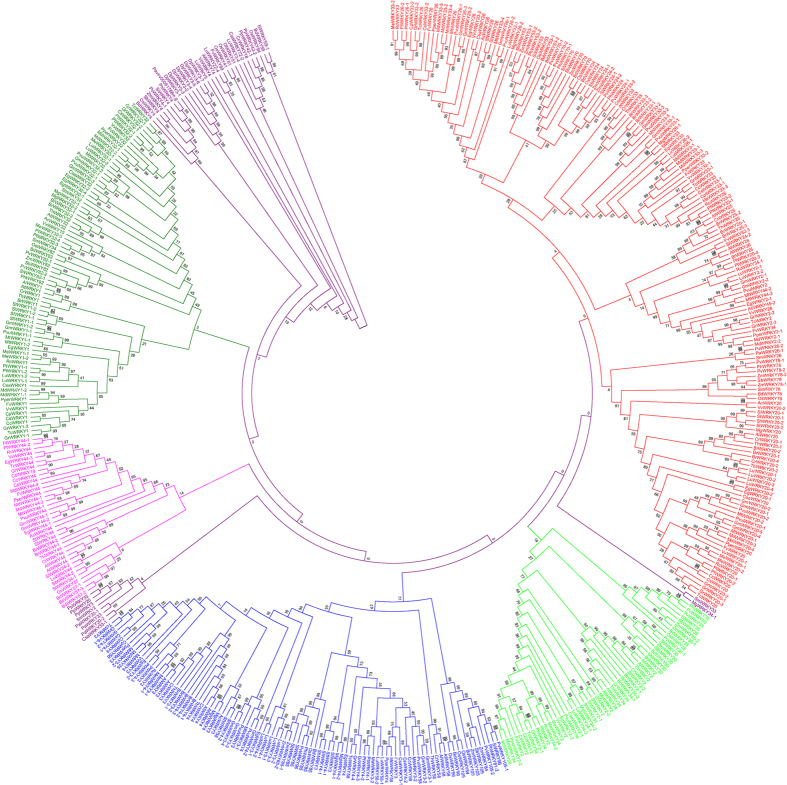
Unrooted phylogenetic tree of N- and C-terminal WRKY domains containing WRKY TFs. The phylogenetic tree shows the presence of seven phylogenetically distinct groups, I (red), II (lime), III (blue), IV (purple), V (pink), VI (green) and VII (purple). The phylogenetic tree was constructed by using MEGA6.

The phylogenetic trees of plant WRKY TFs were constructed in order to better understand the phylogenetic relationship among them. Five phylogenetic trees were constructed by dividing the WRKY TFs into different groups to better understand the grouping and phylogenetic relationship among them. In the first case, the WRKY TFs of monocots, dicots and basal eukaryotic (amoebae, algae, bryophytes, pteridophytes and gymnosperms) plants were combined and used to construct a phylogenetic tree. The results showed the presence of eight phylogenetically distinct and independent groups that were denoted as groups I (red), II (lime), III (black), IV (blue), V (black), VI (pink), VII (green) and VIII (black) (Fig. 4, Table 2). The phylogenetic tree generated from monocots and lower eukaryotic plants formed six phylogenetically distinct groups and named as groups I (red), II (lime), III (green), IV (blue), V (pink) and VI (green) (Fig. 5, Table 3). The sub-group of group II was absent in monocot plants. The phylogenetic tree formed from dicot and lower eukaryotic WRKY TFs yielded three groups namely, groups I (pink), IIa (red), IIb (lime), IIc (blue), and III (green) (Fig. 6, Table 4). When all the WRKY TFs of monocot, dicot, and lower eukaryotic plants that contain only C-terminal WRKY domain were combined, the phylogenetic tree resulted in six groups namely groups I (red), II (lime), III (blue), IV (pink), V (green) and VI (purple) (Fig. 7, Table 5). Similarly, all WRKY TFs of monocot, dicot and lower eukaryotic plants that contained both N- and C-terminal WRKY domains were combined; this resulted in the generation of a phylogenetic tree containing seven groups. The groups are named as group I (red), II (lime), III (blue), IV (purple), V (pink), VI (green) and VII (purple) (Fig. 8, Table 6).

The substitution pattern and evolution rates were estimated by analyzing the shape parameters for the discrete gamma distributions. The rates were estimated using the Jones-Taylor-Thornton (JTT) model (+G). A discrete gamma distribution was used to model evolutionary rate differences among sites (5 categories, [+G]). The mean evolutionary rates for dicot and lower eukaryotic WRKY protein were 0.15, 0.42, 0.75, 1.23, and 2.45 substitutions per site. The amino acid frequencies were 7.69% (A), 4.25% (N), 5.13% (D), 2.03% (C), 4.11% (Q), 6.18% (E), 7.47% (G), 2.30% (H), 5.26% (I), 9.11% (L), 5.95% (K), 2.34% (M), 4.05% (F), 5.05% (P), 6.82% (S), 5.85% (T), 1.43% (W), 3.23% (Y), and 6.64% (V). For estimating ML values, a tree topology was automatically computed. The maximum log likelihood for this computation was −19363.118. The analysis involved 774 amino acid sequences. The mean evolutionary rates for monocot and lower eukaryotic WRKY proteins were 0.15, 0.42, 0.75, 1.23 and 2.44 substitutions per site. The amino acid frequencies were 7.69% (A), 5.11% (R), 4.25% (N), 5.13% (D), 2.03% (C), 4.11% (Q), 6.18% (E), 7.47% (G), 2.30% (H), 5.26% (I), 9.11% (L), 5.95% (K), 2.34% (M), 4.05% (F), 5.05% (P), 6.82% (S), 5.85% (T), 1.43% (W), 3.23% (Y) and 6.64% (V). The maximum log likelihood for this computation was −16801.681 and the analysis involved 896 amino acid sequences. The mean evolutionary rates for WRKY proteins that contained a single WRKY domain were 0.13, 0.40, 0.73, 1.23, and 2.51 substitutions per site. The amino acid frequencies are 7.69% (A), 5.11% (R), 4.25% (N), 5.13% (D), 2.03% (C), 4.11% (Q), 6.18% (E), 7.47% (G), 2.30% (H), 5.26% (I), 9.11% (L), 5.95% (K), 2.34% (M), 4.05% (F), 5.05% (P), 6.82% (S), 5.85% (T), 1.43% (W), 3.23% (Y), and 6.64% (V). The maximum log likelihood for this computation was -13476.656. The analysis involved 445 amino acid sequences. The mean evolutionary rates for WRKY proteins that contained double WRKY domains were 0.11, 0.36, 0.70, 1.22, and 2.60 substitutions per site. The amino acid frequencies were 7.69% (A), 5.11% (R), 4.25% (N), 5.13% (D), 2.03% (C), 4.11% (Q), 6.18% (E), 7.47% (G), 2.30% (H), 5.26% (I), 9.11% (L), 5.95% (K), 2.34% (M), 4.05% (F), 5.05% (P), 6.82% (S), 5.85% (T), 1.43% (W), 3.23% (Y), and 6.64% (V). The maximum log likelihood for this computation was -30333.349. The analysis involved 480 amino acid sequences. All positions with less than 95% site coverage were eliminated. Thus, fewer than 5% alignment gaps, missing data, and ambiguous bases were allowed at any position.

### Statistical analysis of WRKY TFs

Tajima’s relative rate test was conducted to determine the statistical significance of the investigated WRKY TFs. In all three replicate analyses, the *p-*values were found to be significant. The *X*^2^ –test results with one degree of freedom were 5.76 (for monocot, dicot and lower eukaryotic WRKY TFs), 13.76 (for monocot and lower eukaryotic WRKY TFs), 4.45 (for dicot and lower eukaryotic WRKY TFs), 5.00 (for single WRKY domain containing WRKY TFs), and 7.41 (for double WRKY domain containing WRKY TFs) (Table 7).

### Gene expression profile of WRKY TFs

The expression profile of the WRKY TFs was elucidated by investigating the gene expression data for *G. max* and *P. vulgaris* and analyzing their transcription levels. In *G. max*, the transcription profile was determined for different tissue samples, including roots, root hair, leaves, stems, flowers, pods, seeds, nodules and shoot apical meristem. In *G. max*, the expression level of *GmWRKY65-1* was found to be the highest (105.342) among all other WRKY transcription factors (Supplementary Table 2). The expression levels of *GmWRKY6-4* and *GmWRKY6-5* in the root were found to be 74.668 and 43.341, respectively. Some other WRKY TFs, the expression levels of which were relatively higher than those of others were *GmWRKY6-6*, *GmWRKY11-2*, *GmWRKY11-3*, *GmWRKY11-4*, *GmWRKY11-6*, and *GmWRKY15-2* (Supplementary Table 2). Further, *GmWRKY4-3*, *GmWRKY5-1*, *GmWRKY5-2*, *GmWRKY10*, *GmWRKY13-4*, *GmWRKY18*, *GmWRKY33-2*, *GmWRKY33-3*, *GmWRKY35-1*, *GmWRKY35-2*, *GmWRKY47-1*, *GmWRKY47-2*, *GmWRKY47-3*, *GmWRKY50-1*, *GmWRKY50-2*, *GmWRKY54-1*, *GmWRKY57-1*, *GmWRKY69-1*, *GmWRKY69-2*, *GmWRKY70-3*, *GmWRKY71-2*, *GmWRKY72-1*, and *GmWRKY72-2* were not expressed in the root tissues (Supplementary Table 2). Unlike the higher expression in roots, the expression of *GmWRKY65-1*(35.199) was also found to be the highest in the root hair. Some other WRKY TFs that were expressed relatively at higher levels were *GmWRKY6-4*, *GmWRKY11-1*, *GmWRKY11-2*, *GmWRKY11-3*, *GmWRKY11-4*, *GmWRKY11-6*, *GmWRKY11-7*, *GmWRKY11-8*, *GmWRKY15-1*, and *GmWRKY15-2* (Supplementary Table 2). The WRKY TFs, the expression of which was not detected in root tissues, were *GmWRKY4-3*, *GmWRKY6-3*, *GmWRKY10*, *GmWRKY13-4*, *GmWRKY29-1 GmWRKY54-1*, *GmWRKY54-2*, and *GmWRKY56-1* (Supplementary Table 2). In the leaf tissue, the expression level of *GmWRKY6-5* (81.847) was found to be highest among other WRKY TFs. The expression of *GmWRKY26-2* in the leaf tissue was found to be 80.957. Some other WRKY TFs, the expression of which was found to be higher in the leaf tissue, were *GmWRKY6-4*, *GmWRKY15-1*, *GmWRKY15-2*, *GmWRKY26-3*, *GmWRKY41-1*, *GmWRKY41-2*, *GmWRKY41-3*, and *GmWRKY41-7* (Supplementary Table 2). The WRKY TFs, expression of which was not detected in the leaves were *GmWRKY4-3*, *GmWRKY6-3*, *GmWRKY10*, *GmWRKY13-4*, *GmWRKY40-1*, *GmWRKY40-9*, *GmWRKY41-4*, *GmWRKY41-6*, *GmWRKY47-1*, *GmWRKY50-1*, *GmWRKY50-2*, *GmWRKY51-1*, *GmWRKY51-2*, *GmWRKY51-3*, *GmWRKY51-4*, *GmWRKY55-1*, *GmWRKY55-3*, *GmWRKY56-1*, *GmWRKY56-3*, *GmWRKY70-1*, *GmWRKY70-2*, *GmWRKY70-3*, *GmWRKY70-6*, and *GmWRKY70-7* (Supplementary Table 2). In flowers, a higher level of expression was detected in *WRKY26-2* (67.456), *WRKY26-3* (51.836), *WRKY70-6* (61.053), and *WRKY70-7* (63.153) whereas, that of *GmWRKY10*, *GmWRKY13-4*, *GmWRKY29-1*, *GmWRKY50-2*, *GmWRKY67*, *GmWRKY70-4*, *GmWRKY72-2*, and *GmWRKY72-4* was not detected. The expression of *GmWRKY44-2* (17.882), *GmWRKY23-4* (10.417), *GmWRKY11-5* (9.898), *GmWRKY11-6* (9.725) and *GmWRKY3-1* (9.665) was higher in pods. The expressions of *GmWRKY4-3*, *GmWRKY6-3*, *GmWRKY10*, *GmWRKY13-4*, *GmWRKY21-2*, *GmWRKY21-3*, *GmWRKY29-1*, *GmWRKY40-4*, *GmWRKY48-2*, *GmWRKY50-2*, *GmWRKY54-1*, *GmWRKY55-2*, *GmWRKY56-1*, *GmWRKY70-3*, *GmWRKY70-4*, *GmWRKY72-1*, *GmWRKY72-2*, *GmWRKY72-4*, *and GmWRKY72-6* was not detected in pods. In seeds, the expression of *GmWRKY 21-2* (11.200), and *GmWRKY21-3* (31.762) was higher whereas that of *GmWRKY3-4*, *GmWRKY5-1*, *GmWRKY6-3*, *GmWRKY10*, *GmWRKY13-4*, *GmWRKY18*, *GmWRKY21-1*, *GmWRKY29-1*, *GmWRKY30-1*, *GmWRKY32-1*, *GmWRKY32-2*, *GmWRKY32-3*, *GmWRKY40-1*, *GmWRKY40-2*, *GmWRKY40-3*, *GmWRKY40-4*, *GmWRKY40-9*, *GmWRKY40-10*, *GmWRKY41-1*, *GmWRKY47-1*, *GmWRKY50-1*, *GmWRKY50-2*, *GmWRKY51-1*, *GmWRKY51-2*, *GmWRKY54-1*, *GmWRKY54-2*, *GmWRKY55-1*, *GmWRKY55-2*, *GmWRKY56-1*, *GmWRKY56-2*, *GmWRKY56-3*, *GmWRKY67*, *GmWRKY70-1*, *GmWRKY70-2*, *GmWRKY70-3*, *GmWRKY70-4*, *GmWRKY70-5*, *GmWRKY70-6*, *GmWRKY71-1*, *GmWRKY72-1*, *GmWRKY72-2*, *GmWRKY72-3*, *GmWRKY72-4*, *GmWRKY72-5*, *GmWRKY72-6* and *GmWRKY75-3* was not detected. The expression of *GmWRKY65-1* (39.186) was the highest in the nodules. Some other genes, the expression of which was higher in the nodules were *GmWRKY* (30.341), *GmWRKY11-2* (36.175), *GmWRKY11-3* (18.965), *GmWRKY11-4* (20.702), *GmWRKY11-7* (21.960), *GmWRKY11-8* (17.019), *GmWRKY15-1* (17.912), *GmWRKY15-2* (18.552), and *GmWRKY69-1* (17.523). The expression of *GmWRKY70-7* (35.173) was the highest in the shoot apical meristem. Some other WRKY TFs that showed higher expression in the shoot apical meristem were *GmWRKY11-8* (18.974), *GmWRKY21-3* (18.442), and *GmWRKY70-6* (16.468). The expression of *GmWRKY4-3*, *GmWRKY6-1*, *GmWRKY6-3*, *GmWRKY10*, *GmWRKY13-2*, *GmWRKY13-4*, *GmWRKY29-1*, *GmWRKY30-2*, *GmWRKY40-4*, *GmWRKY50-1*, *GmWRKY50-2*, *GmWRKY55-2*, *GmWRKY56-1*, *GmWRKY56-2*, *GmWRKY56-3*, *GmWRKY67*, *GmWRKY70-4*, *GmWRKY72-2*, *GmWRKY72-4*, and *GmWRKY72-6* was not detected in shoot apical meristem.

In *P. vulgaris*, the expression of WRKY TFs in different tissue samples, including young trifoliates, leaves, flowers, flower buds, young pods, stems, roots, and nodules was analysed (Supplementary Table 2). In *P. vulgaris* trifoliates, *PvulWRKY17* (37.519) showed the highest expression. Some other genes that showed relatively higher expression in young trifoliates included *PvulWRKY11-2* (21.790), *PvulWRKY15-1* (18.590), *PvulWRKY15-2* (24.308), and *PvulWRKY19-1* (24.328). In contracts, *PvulWRKY9-2*, *PvulWRKY27-1*, *PvulWRKY29-1*, *PvulWRKY35*, *PvulWRKY43-1*, *PvulWRKY47-2*, *PvulWRKY51-1*, *PvulWRKY59-1*, *PvulWRKY59-2*, *PvulWRKY69-1*, *PvulWRKY73-2*, *PvulWRKY73-3*, *PvulWRKY73-4*, *PvulWRKY79-1* and *PvulWRKY79-2* were not expressed in young trifoliates. In the leaf tissue, *PvulWRKY11-2* (25.292) and *PvulWRKY26-1* (25.724) showed higher expression. Some other genes that showed higher expression in leaves were *PvulWRKY7 (19.048*), *PvulWRKY19-1 (16.433*), *PvulWRKY23-1 (19.076*), *and PvulWRKY58 (18.863*). In contrast, *PvulWRKY1-2*, *PvulWRKY5-1*, *PvulWRKY9-2*, *PvulWRKY14*, *PvulWRKY19-2*, *PvulWRKY29-1*, *PvulWRKY35*, *PvulWRKY43-1*, *PvulWRKY47-2*, *PvulWRKY51-1*, *PvulWRKY59-1*, *PvulWRKY59-2*, *PvulWRKY69-1*, *PvulWRKY73-1*, *PvulWRKY73-2*, *PvulWRKY73-3*, *PvulWRKY73-4*, and *PvulWRKY79-1* were not expressed in the leaves. In flowers, *PvulWRKY19-1* (78.755) showed the highest expression followed by *PvulWRKY15-2* (49.015), *PvulWRKY17* (66.844), *PvulWRKY26-1* (76.970), and *PvulWRKY58* (50.788) whereas, *PvulWRKY59-1*, *PvulWRKY59-2*, *PvulWRKY69-1*, *PvulWRKY73-2*, *PvulWRKY73-4*, *PvulWRKY79-1* and *PvulWRKY79-3* were not expressed. The expression of *PvulWRKY11-2* (50.119) was highest in flower buds followed by *PvulWRKY17* (46.894), *PvulWRKY19-1* (23.965) and *PvulWRKY44* (19.068), whereas, *PvulWRKY5-1*, *PvulWRKY5-3*, *PvulWRKY9-2*, *PvulWRKY29-1*, *PvulWRKY43-1*, *PvulWRKY43-2*, *PvulWRKY51-1*, *PvulWRKY59-1*, *PvulWRKY59-2*, *PvulWRKY69-1*, *PvulWRKY73-3*, *PvulWRKY79-1* and *PvulWRKY79-3* were not expressed (Supplementary Table 2). In young pods, *PvulWRKY17* (58.155), *PvulWRKY15-2* (41.848), and *PvulWRKY19-1* (38.820) showed higher expression whereas, *PvulWRKY9-2*, *PvulWRKY51-1*, *PvulWRKY59-1*, *PvulWRKY69-1*, *PvulWRKY73-2*, *PvulWRKY79-1* and *PvulWRKY79-3* were not expressed (Supplementary Table 2). In stems, *PvulWRKY17* (61.321) showed the highest expression whereas *PvulWRKY9-2*, *PvulWRKY29-1*, *PvulWRKY51-1*, *PvulWRKY59-1*, *PvulWRKY59-2*, *PvulWRKY69-1*, *PvulWRKY73-3*, *PvulWRKY79-1*, *PvulWRKY79-2*, and *PvulWRKY79-3* were not expressed. In roots *PvulWRKY11-2* (134.816) showed the highest expression whereas *PvulWRKY29-1*, *PvulWRKY45-1*, *PvulWRKY45-2*, *PvulWRKY59-1*, *PvulWRKY59-2*, and *PvulWRKY69-1* were not detected. In nodules, *PvulWRKY11-2* (79.023) showed the highest expression followed by *PvulWRKY9-2* (48.761), *PvulWRKY11-1* (36.555), and *PvulWRKY69-3* (45.336), whereas *PvulWRKY29-1*, *PvulWRKY45-1*, *PvulWRKY45-2*, *PvulWRKY59-1*, *PvulWRKY59-2*, and *PvulWRKY69-1* were not detected (Supplementary Table 2).

## Discussion

### Identification and nomenclature of WRKY TFs

Advancements in genome sequencing technology and available of well annotated genome database led us to identify the WRKY TF gene family of 43 species. Predicting the potential function and activity of newly sequenced genes and their protein products in every organism is very difficult. The major cellular roles of newly identified genes/proteins can be inferred from previously characterized orthologous gene members of the same family. Large-scale comparative genomic studies can reveal important information regarding the function and evolutionary relationship of orthologous species^55^. The same principle can be applied at the gene family level as well (e.g. WRKY TF gene family). Therefore, we identified and analysed the WRKY TF gene family members from 43 different plant species. All identified WRKY TFs were assigned a specific name according to the orthology based nomenclature system^55^,^56^,^57^,^58^. Providing a unique name to every gene is necessary for its future identification. The role of a genome is insignificant unless a comparative genomics study is conducted.

### Genomics of WRKY TFs

Availability of large-scale genomic data from various plant species allowed the detailed investigation of the WRKY TF gene family in plants. The WRKY TF gene family members vary across species likely because of gene duplication, whole genome duplication, ploidy, gene deletion or mutation. WRKY TFs are considered to be evolutionary conserved and supposed to be present only in plants^4^,^7^,^59^. However, the WRKY TF gene family was also found in amoeba, fungi and diplomonad species^54^,^60^. *Dictyostelium purpureum*, the amoeba that lives in soil belongs to the phylum mycetozoa. The genome of this species encodes nine WRKY TFs. The tetraploid monocot plant *P. virgatum* encodes the highest number (167) of WRKY TFs, whereas, the unicellular *C. reinhardtii* and *C. subellipsoidea* encode for the lowest number (only one) of WRKY TFs. In general it is a general assumption that, larger the genome size more will be the number of WRKY TFs in the genome; however, this concept is not true. Genome size is not directly related to the number of genes of a gene family in the genome (Mohanta *et al.* 2015; Mohanta *et al.* 2015; Mohanta *et al.* 2015). Therefore, the presence of a higher or lower number of genes in a gene family of a particular species can be attributed to its functional requirement and diverse cellular processes. Cai *et al.*^46^ reported the presence of 120 WRKY TFs in *Gossypium raimondii*, which is similar to the number of WRKY TFs identified in our study^46^. Li *et al.*^49^ reported the presence of only 47 WRKY TFs in *Ricinus communis*^49^, however, in our study, 57 WRKY TFs were identified. Muthamilarasan *et al.*^50^ reported the presence of 105 WRKY TFs in *Setaria italica*^50^, whereas, in our study 106 WRKY TFs were identified. Wen *et al.*^14^ reported the presence of 86 WRKY TFs in *Brachypodium distachyon*^14^ whereas only 81 WRKY TFs were identified in this study. Wen *et al.*^14^ have included locus ID LOC100843345, LOC100834454, LOC100845846, and LOC100837754 as locus ID for the gene name *BdWRKY52*, *BdWRKY69*, *BdWRKY73* and *BdWRKY75*, respectively (Supplementary Table 1 of Wen *et al.*^14^); however, we did not find any such sequences from the phytozome database. This indicates that these locus IDs do not belong to *B. distachyon* and hence *B. distachyon* do not encode 86 WRKY TFs. We also compared our results with plant transcription factor databases http://plntfdb.bio.uni-potsdam.de/v3.0/ 61 and http://planttfdb.cbi.pku.edu.cn/ 62. In the majority of the cases, our study results were consistent with those of previous studies where splice variants were excluded as a gene. Splice variants are variants of a particular gene/locus; therefore, they cannot be considered as an independent gene locus. The dicot plant *Linum usitatissimum* encodes the highest number (26) of double WRKY domain proteins, whereas the tetraploid plant *B. distachyon*, which has a larger genome, encodes only 17 double WRKY domain proteins. This shows that the genome size plays no role in determining whether single or double WRKY domain proteins are encoded and this might be completely based on the functional requirement of an organism. Further, we found that the lower eukaryotic organisms *Chlamydomonas reinhardtii*, *Coccomyxa subellipsoidea*, *Ostreococcus lucimarinus*, *Physcomitrella patens* and *Volvox carteri* encoded at least one WRKY TF that contained a double WRKY domain. Three and four WRKY domain containing WRKY proteins were absent in lower eukaryotes, and are only present in a few higher eukaryotic plants. This shows that these three and four WRKY domain-containing WRKY TFs might have evolved recently. The WRKY TF gene family of *Oryza sativa* was previously reported to contain 102 WRKY TFs^63^. In this study, we eliminated OsWRKY94 since it was not found to contain any WRKY domain. Ross *et al.*^63^ also reported the absence of any WRKY domain in OsWRKY94^63^.

In the present study, we identified several novel chimeric WRKY TFs from different plant species (Fig. 2) with varying numbers of WRKY domains and other novel domains fused with them (Fig. 2A to P). These chimeric WRKY TFs might have evolved recently via fusion with other domains^64^. The kinase domain phosphorylates to its target protein. Thus, determining whether, these fused kinase domains play any crucial role in the auto-phosphorylation events in the WRKY TFs to which they are fused, and hence regulate gene expression. In some cases, the kinase domain is followed by a WRKY domain (Fig. 2E and F), whereas, in other cases the WRKY domain is followed by a kinase domain (Fig. 2G). The kinase domains of WRKY TFs most likely get phosphorylated by the cognate up-stream kinase, and regulate the expression of WRKY TFs^65^. The position of the kinase domain might be speculated to be very important in the regulation of WRKY TFs and the phosphorylation events in plants. In some other cases, the WRKY domain is fused with the toll-interleukin receptor (TIR) domain (Fig. 2J and K), which mediates the interactions between the toll-like receptor and signal transduction components^66^,^67^,^68^. Plant proteins that harbor TIR motifs are associated with plant resistance to disease^67^,^69^,^70^. Therefore, the WRKY TFs that harbors the TIR motif might control disease resistance in plants. The leucine-rich repeat (LRR) motif also involved in plant resistance to diseases^69^, and the WRKY TFs that harbor both the TIR and LRR motifs might also remarkably contribute to plant disease resistance.

The diploid species, *B. rapa* encodes 145 WRKY TFs. Of them three encode novel chimeric WRKY TFs (Fig. 2). Among the three novel WRKY TFs, one is fused with the CBS domain (Fig. 2D), and the other two are fused with the kinase domain (Fig. 2G). The CBS domain is found in various other proteins, including adenosine monophosphate (AMP)-activated protein kinase. The CBS domain binds to AMP, adenosine triphosphate (ATP) or s-adenosylmethionine residues, and regulates the activity of associated enzymes^71^. Similarly, the tetraploid species *G. max* encodes 145 WRKY TFs. Among them, only one encodes a chimeric WRKY TF that is fused with the TIR domain (Fig. 2-J). Plant proteins associated with a toll-like receptor mediate disease resistance in plant. The monocot species *Panicum virgatum* encodes two chimeric WRKY TFs; one chimeric WRKY TF is fused with a protease domain (Fig. 2I) and the other with the B3 domain. The B3 domain was previously reported to be a DNA-binding domain present in combination with auxin response factor (ARF); it has been found with the WRKY protein, abscisic acid insensitive 3 (ABI3), or related to ABI3/VP1 (RAV) like TFs. The results of this study showed that the B3 domain, which is present in combination with WRKY TFs might mediate auxin and abscisic acid signaling. The model monocot plant *O. sativa* encodes 101 WRKY TFs, of which one contains a chimeric WRKY TF, which is fused with the protease domain (Fig. 2-I). Presence of an ULP protease domain in conjunction with the WRKY protein indicates that WRKY TFs plays a crucial role in the ubiquitination process of the SUMO protein. *Linum usitatissimum* and *Brassica rapa* encode chimeric WRKY TFs that contain squamosa promoter-binding proteins (ZF_SBP) domain. The SBPs are a major family of plant-specific TFs related to flower development^72^. The SBP zinc finger binds to the consensus sequence TNCGTACAA^73^. The presence of ZF_SBP domain along with WRKY TFs might increase the binding efficiency of WRKY TFs to other consensus sequences such as TNCGTACAA. In addition, the role of the SBP domain in flower development indicates that WRKY TFs with three WRKY domains and a ZF_SBP domain might regulate flower development in plants. The paired amphipathic helix (PAH) domain is found in the components of a co-repressor complex that silences the transcription process and plays a remarkable role in the transition between proliferation and differentiation^74^. The presence of a PAH domain along with a WRKY domain suggests its role in the translational co-repression of cellular proliferation and differentiation. The ATP_GRASP super-family genes regulate several metabolic pathways, including de novo purine biosynthesis, and the biosynthesis of fatty acids, peptidoglycan, glutathione, ribosome, arginine, pyrimidine, polyphosphate, lysine and dipeptide^75^. The fusion of WRKY TFs with the ATP_GRASP domain suggests that these WRKY TFs might be involved in diverse cellular process. All novel genomic rearrangements appear to have evolved recently. In addition, their abundance is very limited; they are present only in a fewer number of species. Once formed, these chimeric genes undergo positive selection when they combine with different components of signaling pathways. This might lead to the creation of a new and diverse signaling pathway, or accelerate the existing signaling process via short-circuiting signaling pathways.

### Conserved domains of WRKY TFs

Multiple sequence alignment of C-terminal WRKY TFs revealed the presence of conserved W-R-K-Y-G-Q-K and C-x_(7)_-C-x_(26)_-H-x-H domains (Supplementary Figure 1). When multiple sequence alignment was conducted using WRKY TFs that contained only double WRKY domains (both N- and C-terminal), the N-terminal region showed the presence of conserved W-R-K-Y-G-Q-K and C-x_(5)_-C-x_(23)_-H-x-H whereas the C-terminal region showed the presence of conserved W-R-K-Y-G-Q-K and C-x_(4)_-C-x_(23)_-H-x-H domains (Supplementary Figure 2). Although the W-R-K-Y-G-Q-K heptapeptide sequence was highly conserved, sequence similarity beyond the domain was considerably low among most genes. Instead of harboring the W-R-K-Y domain, several WRKY TFs were found to contain W-K-K-Y, W-T-K-Y, W-S-K-Y, W-H-K-C, W-Q-K-Y, W-R-K-C, W-K-K-C, W-H-Q-Y, R-S-Q-Y, G-R-K-Y, W-R-E-Y, W-L-K-Y, W-R-K-R, W-R-K-N, W-R-K-D, F-R-K-Y, W-I-K-Y, W-R-I-Y, W-W-K-N and W-W-K-S domains (Fig. 3). These domains were exactly aligned with the W-R-K-Y domains and hence assumed to be newly evolved. Among these new domains, W-K-K-Y, W-T-K-Y, W-S-K-Y, W-H-K-C, W-Q-K-Y, W-R-K-C, W-K-K-C, W-H-Q-Y, R-S-Q-Y, G-R-K-Y, W-R-E-Y, W-L-K-Y, and W-R-K-R are present in the N-terminal region, whereas W-R-K-N, W-R-K-D, F-R-K-Y, W-I-K-Y, W-R-I-Y, W-W-K-N and W-W-K-S are present in the C-terminal region (Fig. 3). Therefore, the entire WRKY TF gene family which might result from long-time evolutionary history, represents divergent WRKY domains even in very closely related gene pairs. Characterization of these novel motifs might shed new insight into their functional significance.

### Phylogeny and grouping of WRKY TFs

The WRKY TF gene family from various plant species, including *A. thaliana*^4^,^76^, *B. distachyon*^14^, *G. raimondii*^46^, *O. sativa*^48^, *S. lycopersicum*^51^, *T. aestivum*^52^ has been well elucidated. Surprisingly, when we combined the data from several published reports, none of them were found to be correlated with one another (Table 8). The WRKY TF group members of different species vary and are not consistent (Table 8). Different researchers have used different nomenclature and grouping systems for WRKY TFs. Eulgem *et al.*^4^ has grouped WRKY TFs as groups I, IIa, IIb, IIc, IId, IIe, and III^4^ whereas, Wang *et al.*^76^ grouped them as IN, IC, IIa, IIb, IIc, IId, IIe and III^76^. Wu *et al.*^48^ grouped the WRKY TF gene family of *O. sativa* as Ia [NTWD (N-terminal WRKY domain), CTWD (C-terminal WRKY domain)], Ib, IIa, IIb, IIc, IId and III^48^, whereas Okay *et al.*^52^ grouped the WRKY TFs of *T. aestivum* as groups I, IIa, IIb, IIc, IId, IIe, and III^52^. Thus, there are hardly any consistencies in the grouping system of WRKY TFs. Moreover, none of the WRKY TF group members of one research group are consistent with those of other research groups. For example, according to Wang *et al.*^76^, *A. thaliana* WRKY TFs 1, 2, 3, 4, 20, 25, 26, 32, 33, 34, 44, and 58 and 8, 12, 13, 23, 24, 28, 43, 45, 48, 56, 68, 71, and 75 are present in groups IN and IC respectively whereas, Eulgem *et al.*^4^ reported that WRKY TFs 1, 2, 3, 4, 10, 20, 25, 26, 32, 33, 34, 44, 45, and 58 are present in group I^4^,^76^. The WRKY TF group members 8, 12, 13, 23, 24, 28, 43, 48, 56, 68, 71, and 75 classified by Wang *et al.*^76^ are absent from group I of Eulgem *et al.*^4^. The *A. thaliana* WRKY group member 10 of Eulgem *et al.*^4^ is absent in group IC and IN of Wang *et al.*^76^ (Table 8). According to Eulgem *et al.*^4^, group IIc contains 8, 12, 13, 23, 24, 28, 43, 48, 49, 50, 51, 56, 57, and 59; group IId contains 7, 11, 15, 17, 21, and 39; and group IIe contains 14, 16, 22, 27, 29, and 35 WRKY TFs, whereas Wang *et al.*^76^ reported the absence of WRKY TF family members in groups IIc, IId and IIe^4^,^76^. According to Wu *et al.*^48^, there is absence of a WRKY TF family member in group IIe (Table 8)^48^. Similar inconsistent grouping exists in other studies as well (Table 8). These inconsistencies might be attributed to the improper nomenclature of WRKY TFs, or improper citations of previously published manuscripts. Notable different sub-groups of a specific group are generally present within that group (e.g., if IIa, IIb, IIc, and IId, others are a sub-group of group II, they would be included itself). However, this concept of grouping was not followed correctly during the grouping of WRKY TFs. In the grouping system developed by Wen *et al.*^14^ (Fig. 3 of Wen *et al.*, 2012), sub-groups IIa and IIb are confined to a phylogenetically distinct group, sub-groups IId and IIe are confined to another phylogenetically distinct group, and sub-group IIc is confined to yet another phylogenetically distinct group. However, how sub-groups IIa and IIb, IId and IIe, and IIc can be sub-group members of group II if they are confined to phylogenetically distinct groups and are phylogenetically far away from other is not clear. Personal correspondence with Wen *et al.*^14^ arrived at a certain conclusion regarding the discrepancies in nomenclature and grouping system for WRKY TFs. Hence, in this study we developed a unified grouping system for WRKY TFs in plants.

The inconsistencies in distribution of different WRKY TF family members within and between groups were overcome by developing an appropriate naming system for all WRKY TFs. In general, the sequences that are highly similar tend to fall into the same group as far as orthology-based similarity is concerned^55^,^56^. The orthology based nomenclature system of WRKY TFs has the potential to overcome this problem; therefore, we developed an unique nomenclature system to all WRKY TFs of 43 species^55^,^56^,^58^. In total, 3035 WRKY TF genes from the 43 species were identified and classified according to the unique naming system (Supplementary Table 1). The nomenclature is described in detail in the Materials and Methods section. Orthology also lends the legitimacy to common ancestry and evolutionary history of function. Therefore, the orthology-based nomenclature system can provide ideas regarding the possible function of specific genes in the plant species being investigated. This nomenclature system can also be extended to the newly identified gene family of other plant species.

A proper grouping system of WRKY TFs was developed by first dividing the studied plant species into different groups. The groups were (I) WRKY TFs of monocot, dicot, and lower eukaryotic (algae, bryophytes, pteridophytes and gymnosperms) plants; (II) WRKY TFs of monocots with lower eukaryotic plants; and (III) WRKY TFs of dicots with lower eukaryotic plants. When phylogenetic trees were constructed by considering the WRKY TFs from monocot, dicot, and lower eukaryotic plants, eight groups were identified (Fig. 4, Table 2, Supplementary Figure 3). In the resultant phylogenetic tree, WRKY TF gene family members were not consistent with any specific group and overlapped in two or more groups. For example, WRKY TFs 3, 5, 7, 8, 10, 11, 13, 16, 17, 19, 22, 23, 24, 25, 26, 28, 29, 33, 34, 36, 43, 45, 48, 49, 50, 51, 56, 57, 58, 59, 67, 68, 71, 72, 75, 77, 84, 102, 103, and 106 belonged to group A and 1, 2, 3, 4, 5, 10, 19, 20, 24, 25, 26, 30, 32, 33, 34, 35, 44, 45, 53, 57, 58, 59, 70, 78, 80, 81, 82, 84, 85, 90, 96, and 105 belonged to group II (Fig. 4, Table 2, Supplementary Figure 3). The WRKY TF members 3, 5, 19, 24, 25, 26, 33, 34, 45, 57, 58, 59, and 84 were distributed in both the groups (group I and II). Similar trends were observed in other WRKY groups as well. Therefore, the grouping of WRKY TFs based on a combined study of monocots, dicots and lower eukaryotic plants did not prove to be suitable. When the phylogenetic tree was constructed by considering the WRKY TF gene family members of monocot and lower eukaryotic plants, six phylogenetically distinct groups were formed; they were named as groups I (red), II (lime), III (green), IV (blue), V (pink) and VI (green) (Fig. 5, Table 3). The WRKY TF gene family members of monocot and lower eukaryotic plants were very specific to their concerned group. In this case, no single WRKY TF member of one specific group overlapped with another group. When the phylogenetic tree constructed by considering WRKY TF gene family members of dicot and lower eukaryotic plants, three different groups were generated where group II contained three sub-groups (Fig. 6, Table 4). We named the groups as I (pink), IIa (red), IIb (lime), IIc (blue), and III (green). We found that the WRKY TF members of groups I and III were very specific to their respective group and did not overlap with one another (Table 4). These results clearly showed that the WRKY TF grouping system is very specific to the lineages (monocot/dicot). The WRKY TF grouping system of monocot and dicot plants differs remarkably; this might be one of the most important reasons why co-linearity was absent in the grouping system of WRKY TF gene family members (Table 8). Therefore, in this study, we proposed that WRKY TF grouping should be specific to monocot or dicot plant lineages. The monocot-specific WRKY TFs can be grouped into six groups (groups I, II, III, IV, V, and VI) whereas dicot-specific WRKY TFs can be grouped into three groups (groups I, IIa, IIb, IIc and III). The phylogenetic tree of monocot and dicot plants varied markedly. This might be due to fact that monocot plant lineage is comparatively more conserved than dicot lineage owing to early ploidy and whole genome duplication^77^,^78^. Therefore, monocot and dicot plants should be grouped according to the grouping system of monocot plants and dicot plants, respectively.

We conducted another analysis by dividing WRKY TFs into single WRKY domain-containing (C-terminal) and double WRKY domain-containing (N- and C-terminal) groups. The phylogenetic analysis in the single WRKY domain group resulted in six phylogenetically distinct groups, whereas the double WRKY domain group resulted in seven phylogenetically distinct groups (Tables 5 and 6). The WRKY TF members of domain specific studies were not confined to any specific group and the group members were overlapped with each other. Although single and double WRKY domain-containing TFs resulted into six and seven phylogenetically independent groups, respectively; only group II of previously studies could be sub-grouped into IIa, IIb, IIc, IId and IIe is not clear. However, the permutation and combination study showed that WRKY TFs could be grouped as monocot and dicot lineage-specific. The WRKY TFs of monocot plants can be grouped into six groups, and dicot plants can be grouped into three groups. Earlier reported grouping systems such as groups I, II (IIa, IIb and IIc) and III can be applied to dicot plants, but it is ensuring that WRKY TF group members are confined to their specific groups is important.

The substitution rate of monocot and lower eukaryotic WRKY was slightly higher than that of dicot and lower eukaryotic WRKY proteins. No considerable difference was observed in the substitution and evolutionary rate of WRKY proteins with a single or double domain. This explains why WRKY proteins are highly conserved across the plant lineage. The phylogenetic analysis of all plant species showed that all WRKY TFs were present in monocot, dicot and lower eukaryotes, indicating that the appearance of most WRKY TFs in plants predates the divergence of these species. No species-specific group, or sub-group or clades were observed in the phylogenetic tree. This implies that the WRKY TF gene family was more conserved during evolution. In addition, the WRKY domains from the same lineage tended to cluster together in the phylogenetic tree, which was not observed in this study. This suggests that they experienced duplication after divergence. The WRKY TFs that clustered together are orthologous ones that are evolutionarily closer than others. The phylogenetic similarity found in this study showed that WRKY TFs evolved conservatively. Only few WRKY TFs were found in lower eukaryotes, including *C. reinhardtii*, *C. subellipsoidea*, *M. pusilla*, and *V. carteri* whereas higher plants possessed a larger number of *WRKY* TF genes. This indicated that the earliest evolutionary origin of the gene containing the WRKY TF was from unicellular green algae. This suggested that WRKY proteins evolved before plants transitioned from an aquatic to a terrestrial habitat. With the continuous evolution of species, land plants have evolved a series of highly sophisticated signaling mechanisms that helped them to adapt to the ever changing environmental conditions, and hence, the number of WRKY TFs increased in different species. Presence of the WRKY TF gene in diplomonands, amoebozoa, and fungi sheds new light on the early evolution of WRKY genes.

Understanding the evolution of the WRKY TFs in plant lineage is very challenging. If the concept of early evolution is considered, in green algae, a BED finger-like C2H2 zinc finger domain incorporated a WRKY domain N-terminal to the zinc finger. This single-domain WRKY TFs served as the progenitor for all other WRKY genes^54^. Subsequently, this single-domain WRKY TFs fused via addition or recombination to yield a double WRKY domain by maintaining the original copy intact. Thereafter, independent lateral gene transfer to non-plant lineage and plant lineage occurred during the early evolution of WRKY TFs. This led to the transfer of WRKY TFs to fungi, amoeba and other species. The amoeba species, *D. purpureum* and the green algae *O. lucimarinus* and *V. carteri* contain both double and single WRKY domain proteins. However, *C. reinhardtii* contains only the double WRKY domain protein. This shows that the single double WRKY domains have coevolved from the green plant lineage. All these events seemed to have occurred before the transition of green plants to a terrestrial habitat. During these evolutionary processes, the chimeric WRKY protein evolved to contain either kinase, NAC, B3, LRR, PAH, CBS, ZF_SBP, ULP_protease, TIR, or ATP_GRASP domain. These chimeric WRKY TFs are not found in all plant species, and are restricted to only the flowering plant lineage. WRKY TFs with other novel domains can be expected from other plant species the genomes of which are yet to be sequenced.

### Gene duplication and evolution

Evolution by gene duplication is one of the most important processes responsible for the supply of raw genetic material to an organism for its biological evolution^79^. Duplication can occur via recombination, aneuploidy, retro-transposition or whole genome duplication. *A. thaliana* encodes about 16,574 (65%) duplicated genes among its total of 25498 genes^79^,^80^. In the present study, we found several duplicated WRKY TFs (Supplementary Table 1). Most duplicated WRKY TF genes are present as paralogous genes^79^. More specifically, gene duplication analysis of some novel WRKY TFs (Fig. 2, Table 9), performed using Pinda (pipeline for intraspecies duplication analysis) server revealed that most of the WRKY TFs are duplicated. Some of the novel WRKY TFs, such as SbWRKY59, PvWRKY94-1 and SiWRKY59-2, were found to be nonduplicated. The Z-score values of these non-duplicated WRKY TFs ranged from 1.11 to 1.78. A z-score value of less than four indicates a non-duplicated gene^81^.

### Statistical analysis

Tajima’s relative rate test, the simplest test that can be applied to test the molecular evolutionary clock, can be applied to both nucleotide and amino acid sequences. This method yields results as the Chi-square test, and can even be applied when the pattern of substitution is unknown or the substitution rate varies across sites^82^. In Tajima’s relative rate test of WRKY TFs, the *p-*value and Chi-square test were found to be significant (Table 7).

### Gene expression profile of WRKY TFs

Understanding the tissue-specific expression of genes can lead to elucidation of the molecular mechanisms and the role of the genes in tissue development and function. Understanding the genes, how they expressed and were regulated in different tissues is a challenging and fundamental question. Therefore, we investigated the tissue-specific expression of WRKY TFs of *G. max* and *P. vulgaris* (Supplementary Table 2). In *G. max*, expression analysis was conducted in the roots, root hairs, leaves, stems, flowers, pods, seeds, nodules and shoot apical meristem tissue. Of the total of 145 *G. max* WRKY TFs, 143 were found to be expressed in either of the mentioned tissues. Expressions of *GmWRKY65-1* (105.342), *GmWRKY6-4* (74.668), and *GmWRKY6-5* (43.341) were found to be significantly higher than those of others in the roots, suggesting their important role in root development. Expression of 24 *GmWRKY* was not detected in root tissue (Supplementary Table 2), indicating that these genes might not play any active role in root development. The expression level of *GmWRKY65-1* (35.199) was found to be the highest in root hair, suggesting its active role in the development of root hair. Expression levels of at least eight genes were not detected in root hairs. The expression levels of *GmWRKY6-4* (51.394), *GmWRKY6-5* (81.847), *GmWRKY26-2* (80.957), *GmWRKY26-3* (72.911), and *GmWRKY41-3* (72.788) were significantly higher in the leaf tissues than in any other tissues, suggests that these genes might play crucial roles in leaf development. Expression levels of at least 24 genes were not detected in leaf tissues. In stems, the expression levels of *GmWRKY21-3* (47.276), *GmWRKY11-6* (24.872), and *GmWRKY15-2* (24.886) were found to be significantly higher than that of other genes, suggesting their role in stem development. Expression levels of at least 15 genes were not detected in the stem tissue. In flowers, the expression levels of *GmWRKY26-2* (67.456), *GmWRKY26-3* (51.836), *GmWRKY70-6* (61.053) and *GmWRKY70-7* (63.153) were found to be significantly higher than those of other genes, suggesting that these genes might plays an important role in flower development. Expression levels of at least eight genes were not detected in flower tissue (Supplementary Table 2). In pods, the expression level of *GmWRKY44-2* (17.882) was found to be significantly higher than that of other genes, suggesting its important role in pod development. The expression levels of at least 19 genes were not detected in pod. In seeds, the expression level of *GmWRKY21-3* (31.762) was found to be significantly higher than that of other genes, suggesting its important role in seed development. In nodules, the expression level of *GmWRKY65-1* (39.186) was significantly higher than that of other genes, suggesting its important role in nodule development. The expression level of *GmWRKY65-1* was higher in root and root hairs as well. Thus, *GmWRKY65-1* might play a crucial role in root, root hair, and nodule development. In the shoot apical meristem, the expression level of *GmWRKY70-7* (35.173) was found to be significantly higher than that of other genes, suggesting its crucial role in apical meristem development. Expression levels of at least 21 genes were not detected in the apical meristem tissue. Considering the ubiquitous expression of WRKY TFs in *G. max*, we found that *GmWRKY6-4*, *GmWRKY6-5*, *GmWRKY11-1*, *GmWRKY11-2*, *GmWRKY11-3*, *GmWRKY11-4*, *GmWRKY11-5*, *GmWRKY11-6*, *GmWRKY11-7*, *GmWRKY11-8*, *GmWRKY15-1*, *GmWRKY15-2*, *GmWRKY20-2*, *GmWRKY20-4*, *GmWRKY22-3*, *GmWRKY22-4*, *GmWRKY26-3*, *GmWRKY35-3*, and *GmWRKY41-7* were highly expressed in all the studied tissues (Supplementary Table 2). Similarly, the expression levels of *GmWRKY10* and *GmWRKY13-4* were not detected in any tissue, while those of *GmWRKY4-3*, *GmWRKY6-3*, *GmWRKY29-1*, *GmWRKY50-1*, *GmWRKY50-2*, *GmWRKY54-1*, *GmWRKY55-1*, *GmWRKY56-1*, *GmWRKY56-3*, *GmWRKY67*, *GmWRKY70-3*, *GmWRKY70-4*, and *GmWRKY72-2* were almost negligible or absent in the major tissue types (Supplementary Table 2).

In *P. vulgaris*, expression analysis was conducted in eight tissue types that included young trifoliates, leaves, flowers, flower buds, young pods, stems, roots, and nodules. In young trifoliates, the expression level of *PvulWRKY17* (37.519) was found to be significantly highest than those of others, suggesting its important role in early stages of plant development. Expression levels of 15 *PvulWRKY* genes were not detected in young trifoliates. In leaves, the expression levels of *PvulWRKY7* (19.048), *PvulWRKY11-2* (25.292), *PvulWRKY19-1* (16.433), *PvulWRKY23-1* (19.076), *PvulWRKY26-1* (25.724) and *PvulWRKY58* (18.863) were found to be significantly higher than those of others, suggesting that these genes might play a significant role in leaf development in *P. vulgaris*. Expression levels of 18 genes were not detected in the leaf tissue. In flowers, the expression levels of *PvulWRKY11-2* (47.243), *PvulWRKY15-2* (49.015), *PvulWRKY17* (66.844), *PvulWRKY19-1* (78.755), *PvulWRKY26-1* (76.970), and *PvulWRKY58* (50.788) were found to be significantly higher than those of other WRKY genes, suggesting their important role in flower development. Unlike in flower development, the expression level of *PvulWRKY11-2* was found to be the highest in flower bud, suggesting that this gene might be involved in flower and flower bud development. In young pods, the expression levels of *PvulWRKY17* (58.155), *PvulWRKY15-2* (41.848), and *PvulWRKY19-1* (38.820) were found to be significantly higher than those of other genes, suggesting their role in pod development. The expression levels of seven genes were not detected in young pods. In stems, the expression levels of *PvulWRKY11-1*, *PvulWRKY11-2* and *PvulWRKY17* were found to be significantly higher than those of other genes, suggesting their role in stem development. In roots, the expression levels of *PvulWRKY11-1*, *PvulWRKY11-2*, *PvulWRKY17* and *PvulWRKY69-3* were found to be significantly higher, suggesting that these genes might significantly regulate root development in *P. vulgaris*. In nodules, the expression of *PvulWRKY9-2* (48.761), *PvulWRKY11-2* (79.023), and *PvulWRKY69-3* (45.336), was higher than those of other genes, suggesting their important role in nodule development. In *P. vulgaris*, few genes were found to be ubiquitously expressed in all tissue type such as *PvulWRKY7*, *PvulWRKY11-1*, *PvulWRKY11-2*, *PvulWRKY11-3*, *PvulWRKY15-1*, *PvulWRKY15-2*, *PvulWRKY17*, *PvulWRKY19-1*, *PvulWRKY20-1*, *PvulWRKY20-2*, *PvulWRKY21*, *PvulWRKY22-2*, *PvulWRKY23-1*, *PvulWRKY23-2*, *PvulWRKY58*, *PvulWRKY69-3* and *PvulWRKY71-2* (Supplementary Table 2). Comparative expression studies between *G. max* and *P. vulgaris* WRKY genes showed that *WRKY11-1*, *WRKY11-2* and *WRKY11-3* were ubiquitously expressed in all tissues of *G. max* and *P. vulgaris*. Similarly, *WRKY15-2* was also found to be highly expressed in the stems, roots, nodules, and pods of *G. max* and *P. vulgaris*, suggesting their common function in both the plants and similar tissue types. *WRKY65* was also found to be highly expressed in the root and nodule tissues in *G. max* and *P. vulgaris*, suggesting that this gene might be extensively involved in root and nodule development in both the plants.

## Conclusion

Analysis of the WRKY TF gene family across the plant lineage revealed the presence of novel WRKY TFs. The monocot or dicot lineage specific grouping and orthologous-based nomenclature system of WRKY TFs might be crucial in future studies. Expression analysis showed that *WRKY11-1*, *WRKY11-2*, and *WRKY11-3* were highly expressed in all tissue types in *G. max* and *P. vulgaris*. Similarly, *WRKY15-2* was found to be highly expressed in the stems, roots, nodules and pods in *G. max* and *P. vulgaris*, suggesting its important role in the development of these tissues. Understanding the functional role of novel WRKY TFs will help to understand their functional and evolutionary roles.

## Material and Methods

### Identification of WRKY TFs

WRKY TFs from the model organisms *A. thaliana* and *O. sativa* were downloaded from The Arabidopsis Information Resource (TAIR) database and the Rice Genome Annotation project respectively^83^,^84^. The protein sequences of WRKY TFs from *A. thaliana* and *O. sativa* were used as query sequences to search the WRKY TFs in other plant species in the phytozome database^85^. The WRKY TFs from *O. sativa* were used to search the WRKY TFs from monocot plants, and *A. thaliana* WRKY TFs were used to search the TFs from dicot and other plant species. Overall, WRKY TFs gene families of 43 plant species were investigated. The Hidden Markov Model (HMM) and BLASTP program was used as well to search the WRKY TFs of the investigated plant species by using the default parameters of the phytozome database. The sequences generated by BLASTP searches were collected for further analysis to confirm whether they were WRKY TFs. All the collected sequences were then analysed using the scanprosite and MEME software to confirm the presence of WRKY domains^86^,^87^. Default parameters were used in the scanprosite software to identify the WRKY domains. The identified sequences that contained the WRKY domain were retained for further validation which was accomplished by subjecting the sequences to BLASTP analysis in the TAIR and rice genome annotation project database using the default parameters. Further, all the sequences were analysed using HMMER web server to identify the interactive sequence similarities^88^. Sequences that resulted in BLASTP hits with WRKY TFs in the TAIR or rice genome annotation database were confirmed as WRKY TFs.

### Nomenclature of WRKY TFs

All identified WRKY TFs were assigned a specific name. Nomenclature of the WRKY TFs was assigned according to an orthology-based nomenclature system proposed by different researchers^55^,^56^,^89^. In the nomenclature system, names were assigned by considering the first letter of the genus in upper case and the first letter of the species in lower case followed by the WRKY and orthology-based number of *A. thaliana* or *O. sativa*. When redundancies were found in the nomenclature system, 2 to 4 letters of the species name were considered for the nomenclature. When more than one orthologous gene was found, they were considered as paralogous genes which were numbered by including a hyphen. For example, if there are two *OsWRKY46* in *O. sativa*, they would be named *OsWRKY46-1* and *OsWRKY46-2*.

### Multiple sequence alignment

The multiple sequence alignment of WRKY TFs was conducted using the Multalin software (http://multalin.toulouse.inra.fr/multalin/) with default parameters which were as follows: protein weight matrix, Blossum62-12-12; gap penalties at opening, default; gap penalty at extension, default; gap penalty at extremities, none; one iteration only, any; high consensus value, 90% (default); and low consensus value, 50% (default). The multiple sequence alignment of proteins containing single and double WRKY domain was conducted separately by using the same parameters.

### Construction of phylogenetic tree

Unrooted phylogenetic trees were constructed to understand the closeness and evolutionary relatedness of WRKY TFs in plants. We constructed different phylogenetic trees by grouping the WRKY TFs into different groups. Groupings included (1) monocot, dicot and lower eukaryotic plants, (2) monocot and lower eukaryotes, (3) dicot and lower eukaryotes (4) single WRKY domain (C-terminal WRKY domain)-containing WRKY TFs and (5) double WRKY domain (N- and C-terminal WRKY domain)-containing WRKY TFs. To construct the phylogenetic trees, we created clustal files for each group using the clustalW or clustal omega program^90^,^91^. The generated clustal files were converted to the MEGA file format, after which the MEGA files were run in MEGA6 software to construct the phylogenetic tree^92^. Different statistical parameters used to construct the phylogenetic trees included the following: analysis, phylogeny reconstruction; statistical method, maximum likelihood; test of phylogeny, bootstrap method; number of bootstrap replicates, 1000; substitution type, amino acids; model/method, Poisson model; rates among sites, uniform rates; gap/missing data treatment, partial deletion/use all sites; site coverage, 95%; ML heuristic method, nearest-neighbor-interchange (NNI); and branch swap filter, very strong.

### Statistical analysis

Different statistical analyses were performed to understand the evolutionary aspects of WRKY TFs using the MEGA6 program^92^. The MEGA files of all five groups that were used in the construction of the phylogenetic tree were subjected to the MEGA6 program for statistical analysis. Tajima’s relative rate test was conducted to evaluate the statistical significance of WRKY TFs to understand whether there were significant variations in molecular evolution. In this test, sequences 1, 2, and 3 were considered simultaneously where sequence 3 was considered as an out group. If n_ijk_ was the observed number of sites in which sequences 1, 2 and 3 have protein/nucleotides I, j, and k. under the molecular clock hypothesis, E(n_ijk_) = E(n_jik_) irrespective of the substitution model used and whether the substitution rate varied across the sites. If the hypothesis is rejected, then the molecular clock hypothesis of evolution can be rejected for the given set of sequences 1, 2 and 3. The statistical parameters used to perform Tajima’s relative rate test were as follows; analysis, Tajima’s relative rate test; scope, for 3 chosen sequences; substitution type, amino acids; and gaps/missing data treatment, complete deletion.

### Gene duplication analysis

Gene duplication analysis of some selective WRKY TFs performed using the online server Pinda (http://orion.mbg.duth.gr/Pinda)^81^.

All the data used in this study were obtained from publicly available database (https://phytozome.jgi.doe.gov/pz/portal.html, http://congenie.org/start) available in the public domain.

### Gene expression data

The expression data of *G. max* and *P. vulgaris* were downloaded from the phytomine database (https://phytozome.jgi.doe.gov/phytomine/template.do?name=One_Gene_Expression&scope=global) of phytozome. Locus ID of *G. max* and *P. vulgaris* were used for to searching the expression data in different tissue samples.

## Additional Information

**How to cite this article**: Mohanta, T. K. *et al.* Novel Genomic and Evolutionary Insight of WRKY Transcription Factors in Plant Lineage. *Sci. Rep.* **6**, 37309; doi: 10.1038/srep37309 (2016).

**Publisher’s note**: Springer Nature remains neutral with regard to jurisdictional claims in published maps and institutional affiliations.

## Supplementary Material

Supplementary Information

## Figures and Tables

**Table 1:** WRKY TF gene family of 43 species.

Sl. No	Name of Plant Species	Classification	Ploidy level	Abbreviation of WRKY Gene	Single WRKY domain proteins	Double WRKY proteins	Novel WRKY proteins	Total No. of WRKY TFs
1	*Aquilegia coerulea*	Dicot	Diploid	AcWRKY	26	6	1	33
2	*Arabidopsis lyrata*	Dicot	Diploid	AlWRKY	68	10	1	79
3	*Arabidopsis thaliana*	Dicot	Diploid	AtWRKY	62	11	1	74
4	*Brachypodium distachyon*	Monocot	Diploid	BdWRKY	64	17		81
5	*Brassica rapa*	Dicot	Diploid	BrWRKY	118	24	3	145
6	*Capsella rubella*	Dicot	Diploid	CrWRKY	61	11		72
7	*Carica papaya*	Dicot	Diploid	CpWRKY	44	6		50
8	*Chlamydomonas reinhardtii*	Algae	Haploid	CreinWRKY	0	1		1
9	*Citrus clementina*	Dicot	Diploid	CcWRKY	45	7		52
10	*Citrus sinensis*	Dicot	Diploid	CsWRKY	44	9		53
11	*Coccomyxa subellipsoidea*	Algae	Haploid	CsubWRKY	0	1		1
12	*Cucumis sativus*	Dicot	Diploid	CsaWRKY	52	10		62
13	*Dictyostelium purpureum*	Amoeba	Haploid	DpWRKY	2	7		9
14	*Eucalyptus grandis*	Dicot	Diploid	EgWRKY	63	15		78
15	*Fragaria vesca*	Dicot	Diploid	FvWRKY	42	10	5	56
16	*Glycine max*	Dicot	Tetraploid	GmWRKY	120	24	1	145
17	*Gossypium raimondii*	Dicot	Diploid	GrWRKY	100	18	2	120
18	*Linum usitatissimum*	Dicot	Diploid	LuWRKY	76	26	3	105
19	*Malus domestica*	Dicot	Diploid	MdWRKY	103	23		126
20	*Manihot esculenta*	Dicot	Diploid	MeWRKY	89	13		102
21	*Medicago truncatula*	Dicot	Diploid	MtWRKY	63	13		76
22	*Micromonas pusilla*	Algae	Haploid	MpWRKY	2	0		2
23	*Mimulus guttatus*	Dicot	Diploid	MgWRKY	53	12		65
24	*Oryza sativa*	Monocot	Diploid	OsWRKY	86	14	1	102
25	*Ostreococcus lucimarinus*	Algae	Haploid	OlWRKY	1	1		2
26	*Panicum hallii*	Monocot	Diploid	PhWRKY	87	9	1	97
27	*Panicum virgatum*	Monocot	Tetraploid	PvWRKY	150	17	2	168
28	*Phaseolus vulgaris*	Dicot	Diploid	PvulWRKY	73	15		88
29	*Physcomitrella patens*	Bryophyte	Haploid	PpWRKY	30	5		35
30	*Picea abies*	Gymnosperm	Diploid	PaWRKY	56	5	1	62
31	*Populus trichocarpa*	Dicot	Diploid	PtWRKY	80	22		102
32	*Prunus persica*	Dicot	Diploid	PperWRKY	50	10		60
33	*Ricinus communis*	Dicot	Diploid	RcWRKY	48	9		57
34	*Selaginella moellendorffii*	Pteridophyte	Haploid	SmWRKY	15	4		19
35	*Setaria italica*	Monocot	Diploid	SiWRKY	93	13	1	106
36	*Solanum lycopersicum*	Dicot	Diploid	SlWRKY	64	14	1	79
37	*Solanum tuberosum*	Dicot	Diploid	StWRKY	66	13		79
38	*Sorghum bicolor*	Monocot	Diploid	SbWRKY	77	10	3	90
39	*Thellungiella halophila*	Dicot	Diploid	ThWRKY	54	12		66
40	*Theobroma cacao*	Dicot	Diploid	TcWRKY	49	10		59
41	*Vitis vinifera*	Dicot	Diploid	VvWRKY	46	11		56
42	*Volvox carteri*	Algae	Haploid	VcWRKY	1	1		2
43	*Zea mays*	Monocot	Diploid	ZmWRKY	100	16		116

Different species encode different numbers of WRKY TFs loci. Amoeba species *D. purpureum* encode for 9 WRKY TFs. Transcript variants were not included in this study.

**Table 2:** Phylogenetic tree of WRKY TFs of monocot, dicot, and lower eukaryotic (amoeba, algae, bryophyte, pteridophyte, and gymnosperm) plants.

Group I (red)	Group II (lime)	Group III (black)	Group IV (blue)	Group V (black)	Group VI (pink)	Group VII (green)	Group VIII (black)
3, 5, 7, 8, 10, 11, 13, 16, 17, 19, 22, 23, 24, 25, 26, 28, 29, 33, 34, 36, 43, 45, 48, 49, 50, 51, 56, 57, 58, 59, 67, 68, 71, 72, 75, 77, 84, 102, 103, 106	1, 2, 3, 4, 5, 10, 19, 20, 24, 25, 26, 30, 32, 33, 34, 35, 44, 45, 53, 57, 58, 59, 70, 78, 80, 81, 82, 84, 85, 90, 96, 105	4, 59, 10	1, 2, 5, 6, 9, 12, 18, 27, 28, 31, 32, 36, 40, 42, 43, 47, 60, 61, 62, 71, 73, 76, 97	10, 12, 16, 17, 49, 52, 57, 60, 103	2, 4, 6, 7, 11, 12, 13, 14, 15, 16, 17, 21, 22, 25, 27, 29, 31, 35, 37, 39, 43, 51, 52, 57, 64, 65, 66, 68, 69, 74, 83, 87, 88, 94	4, 15, 16, 18, 19, 20, 21, 22, 30, 33, 38, 40, 41, 44, 45, 46, 47, 48, 50, 51, 52, 53, 54, 55, 56, 59, 60, 61, 62, 63, 64, 65, 66, 67, 69, 70, 74, 79, 81, 82, 86, 90, 91, 93, 94, 95, 98, 100, 103, 104	21, 22, 50, 69, 89, 94

The phylogenetic tree revealed eight different groups, but the WRKY TF gene families were not restricted to any specific group, and one or more member of WRKY TFs were distributed in two or more groups. The numbers indicate the number of WRKY TFs (for example 1, 2, and 3 and others indicate WRKY1, WRKY2, and WRKY3 and so on). The phylogenetic tree was constructed using the MEGA6 software and the Poisson substitution model by using 1000 bootstrap replicates.

**Table 3:** Phylogenetic tree of WRKY TFs of monocot and lower eukaryotic plants.

Group I (red)	Group II (lime)	Group III (green)	Group IV (blue)	Group V (pink)	Group VI (green)
15, 17, 18, 19, 20, 21, 22, 40, 44, 45, 46, 47, 48, 50, 52, 53, 54, 55, 56, 57, 58, 59, 60, 61, 64, 65, 69, 70, 74, 79, 81, 84, 86, 90, 91, 93, 94	2, 6, 12, 13, 14, 25, 31, 37, 39, 41, 42, 51, 61, 66, 68, 83, 87, 88, 89, 92, 94	58, 103	4, 24, 30, 35, 53, 59, 70, 78, 80, 82, 85, 90, 96, 105	3, 7, 8, 10, 11, 16, 23, 26, 29, 34, 36, 49, 58, 67, 72, 77, 84, 102	1, 5, 9, 18, 27, 28, 32, 43, 57, 62, 73, 76, 97

Representative WRKY TF members belonging to monocot and lower eukaryotic (amoeba, algae, bryophyte, pteridophyte and gymnosperm) plants. The phylogenetic tree contained six distinct groups. The members of the WRKY TFs were significantly specific to their respective groups. The numbers indicate the WRKY TF members distributed in different groups (1, 2, etc. indicates WRKY1, and WRKY2 and so on). This table confirms that the nomenclature of the entire WRKY TFs gene family is accurate. The phylogenetic tree was constructed using MEGA6 software and the Poisson substitution model using 1000 bootstrap replicates.

**Table 4:** Phylogenetic tree of WRKY TFs of dicot and lower eukaryotic plants.

Group IIa (red)	Group IIb (lime)	Group IIc (blue)	Group I (pink)	Group III (green)
1, 2, 3, 4, 5, 6, 9, 10, 12, 18, 19, 20, 21, 25, 26, 31, 32, 33, 34, 36, 40, 42, 44, 47, 58, 60, 61, 72	3, 4, 5, 8, 10, 13, 23, 24, 28, 43, 45, 48, 49, 50, 51, 56, 57, 59, 68, 71, 75	3, 4, 33, 45, 51	7, 11, 14, 15, 16, 17, 19, 20, 21, 22, 27, 29, 35, 39, 52, 64, 65, 69, 74	30, 38, 41, 46, 53, 54, 55, 62, 64, 66, 67, 70

The phylogenetic tree revealed three phylogenetically distinct groups. The WRKY TF members of groups I, II and III are distributed redundantly. The WRKY members of one group were present in the other groups. This grouping was similar to that reported in previously studies such as groups IIa, IIb, and IIc. The members of groups I and III are significantly specific to their own groups, no members of one group overlap with another. These findings confirm that the nomenclatures of all WRKY TFs are correct. This nomenclature and grouping system should be applied to dicot plants only. Groups I, II, III, IV, V and VI of monocot plants is not the same as that of the respective group of dicots. Hence it is highly recommended to follow lineage specific grouping system to avoid any confusion. The numbers indicate the WRKY TF members distributed in different groups (1, 2, etc. indicate WRKY1, WRKY2 etc.). The phylogenetic tree was constructed using MEG6 software and Poisson substitution model by using 1000 bootstrap replicates.

**Table 5:** Phylogenetic tree of WRKY TFs of monocot, dicot and lower eukaryotic plants that contain only a single WRKY domain (C-terminal WRKY TFs).

Group I (red)	Group II (lime)	Group III (blue)	Group IV (pink)	Group V (green)	Group VI (purple)
4, 5, 15, 18, 19, 20, 21, 22, 30, 32, 38, 40, 41, 44, 45, 46, 47, 48, 50, 52, 53, 54, 55, 56, 58, 62, 63, 64, 65, 66, 67, 69, 70, 74, 75, 79, 81, 93, 98, 100, 101	3, 7, 8, 10, 11, 16, 17, 23, 26, 28, 29, 45, 48, 49, 50, 51, 57, 59, 67, 68, 71, 77, 80	5, 13, 23, 24, 34, 36, 43, 56, 102	1, 6, 7, 11, 12, 13, 14, 15, 17, 22, 25, 27, 29, 31, 35, 37, 39, 42, 51, 65, 66, 68, 69, 88, 89, 92	1, 5, 6, 9, 12, 18, 21, 27, 28, 31, 32, 36, 39, 40, 42, 43, 47, 60, 61, 62, 71, 72, 73, 74, 76, 83, 87, 97	16, 18, 20, 25, 33, 49, 52, 59, 60, 95, 103, 106

The phylogenetic tree was divided into six distinct phylogenetic groups. The numbers indicate WRKY TF members distributed in different groups (1, 2, etc. indicate WRKY1, WRKY2, etc.). Different WRKY TF members are distributed redundantly. For example, WRKY5 is distributed in group I, III and V. The phylogenetic tree was constructed by using the MEGA6 software and the Poisson substitution model using 1000 bootstrap replicates.

**Table 6:** Phylogenetic tree of WRKY TFs of monocot, dicot and lower eukaryotic plants that contain only double WRKY domain (N-terminal and C-terminal WRKY domains).

Group I (red)	Group II (lime)	Group III (blue)	Group IV (purple)	Group V (pink)	Group VI (green)	Group VII (purple)
2, 20, 24, 25, 26, 33, 34, 44, 45, 53, 70, 78, 82	2, 26, 30, 34, 35, 80	3, 4, 58, 81, 84, 85, 96, 105	2, 3, 20, 33	19, 34, 44, 57	1, 10, 32, 82	3, 4, 5, 18, 22, 41, 42, 51, 59, 61, 69, 72, 74, 94

The phylogenetic tree contained seven distinct groups. The numbers indicate the WRKY TF members distributed in different groups (1, 2, etc. indicate WRKY1, WRKY2, etc.). Analysis showed that different WRKY TFs overlap among groups. The phylogenetic tree was constructed using the MEGA6 software and Poisson substitution model by using 1000 bootstrap replicates.

**Table 7:** Tajima’s relative rate test.

Configuration	Monocot, Dicot and lower eukaryotes	Monocot with lower eukaryotes	Dicot with lower eukaryotes	Single Domain	Double domain
Identical sites in all three sequences	20	31	20	24	8
Divergent sites in all three sequences	14	4	20	24	63
Unique differences in Sequence A	16	19	9	15	37
Unique differences in Sequence B	5	2	2	5	17
Unique differences in Sequence C	5	0	7	12	4
*P*-value	0.01638	0.00021	0.03481	0.02535	0.00650
*X*^*2*^ test	5.76	13.76	4.45	5.00	7.41
Degree of freedom	1	1	1	1	1

Equality of evolutionary rate analysis between sequences A (SmWRKY54) and B (SmWRKY55), with sequence C (SmWRKY9) being analysed for monocot, dicot and lower eukaryotic WRKY TFs. The sequences of A (SmWRKY35), and B (SmWRKY6), with sequence C (SmWRKY9) being considered for monocot with lower eukaryotic group; sequences A (SmWRKY15), and B (MdWRKY11-6), with sequence C (SmWRKY9) being considered for dicots with lower eukaryotic group; sequences A (SmWRKY54), and B (ThWRKY50), with sequence C (CsWRKY23) being considered for single WRKY domain containing group; and sequences A (SmWRKY35), and B (PaWRKY72), with sequence C (AtWRKY60) were considered for double domain containing group as per default selection in the MEGA program in Tajima’s relative rate test. The statistical results are presented in Table 7. A *P*-value of less than 0.05 was used to reject the null hypothesis of equal rates between lineages. The analysis involved three amino acid sequences in each group. All positions containing gaps and missing data were eliminated. Evolutionary analyses were conducted using MEGA6.

**Table 8:** Classification and grouping of plant WRKY TFs published by different research groups at different times.

Group 1N	Group 1C	Group IIa	Group IIb	Group IIc	Group IId	Group IIe	Group III		References
1, 2, 3, 4, 20, 25, 26, 32, 33, 34, 44, 58	8, 12, 13, 23, 24, 28, 43, 45, 48, 56, 68, 71, 75	6, 9, 18, 31, 36, 40, 42, 47, 60, 61, 72	7, 11, 14, 15, 17, 21, 22, 27, 29, 35, 39, 52, 65, 69, 74				30, 38, 41, 46, 53, 54, 55, 62, 63, 64, 66, 67, 70		76
1, 2, 3, 4, 10, 20, 25, 26, 32, 33, 34, 44, 45, 58		18, 40, 60	6, 9, 31, 36, 42, 47, 61	8, 12, 13, 23, 24, 28, 43, 48, 49, 50, 51, 56, 57, 59	7, 11, 15, 17, 21, 39	14, 16, 22, 27, 29, 35	30, 41, 46, 53, 54, 55		4
Ia NTWD	Ia CTWD	Ib	25, 66, 81, 82	2, 4, 6, 8, 30, 46, 59, 63	27, 35, 38, 40, 44, 73, 102	9, 14, 16, 26, 29, 39, 43, 45, 61, 65, 85	IIIa	IIIb	48
22, 33, 36, 41, 51, 52, 70, 71, 74, 76, 86, 95, 101	22, 33, 36, 41, 51, 52, 70, 71, 74, 76, 83, 101	1, 3, 7, 10, 12, 13, 15, 23, 24, 28, 34, 37, 42, 47, 56, 57, 58, 62, 67, 92					5, 11, 49, 60, 64, 68, 69, 75, 80, 93, 94	17, 18, 19, 20, 21, 31, 32, 48, 50, 53, 54, 55, 72, 77, 84, 87, 88, 89, 90, 91, 96, 97, 98, 99, 100	
7, 18, 27, 32, 37, 38, 45, 46, 47, 59, 60, 61, 62, 64, 65, 66, 67	7, 18, 27, 32, 37, 38, 45, 46, 47, 59, 60, 61, 64, 65, 66, 67, 75	14, 39, 68	1, 4, 5, 12, 48, 70	1,3, 16, 23, 24, 26, 30, 33, 41, 49, 50, 52, 53, 54, 56, 57, 58, 71, 72, 76	6, 31, 34, 35, 63, 74	13, 21, 36, 42, 69, 73, 77, 80, 81, 82	8, 9, 10, 15, 17, 19, 22, 25, 29, 40, 43, 44, 51, 55, 78, 84, 85		14
1, 2, 3, 4, 5, 14, 15, 18, 20, 31, 32, 33, 34, 36, 44	1, 2, 3, 4, 5, 14, 15, 18, 20, 31, 32, 33, 34, 36, 44	39, 40, 43, 45, 46	6, 9, 16, 17, 72, 73, 74, 76	12, 13, 23, 28, 30, 38, 47, 48, 50, 51, 55, 56, 57, 61, 71, 75	7, 8, 10, 11, 21, 24,	22, 25, 26, 29, 35, 37, 62, 63, 64, 65, 66, 67, 68, 69, 77, 78, 79	19, 41, 42, 52, 53, 54, 58, 59, 60, 80, 81		51
1, 2, 14, 17, 18, 19, 26, 27, 37, 53, 61, 65, 78, 82		4, 8, 34, 39, 43, 52, 54, 60, 71, 79, 80, 81	29, 75, 77	2, 3, 6, 10, 14, 22, 23, 24, 27, 36, 38, 44, 48, 49, 50, 57, 58, 66, 67, 72, 86, 90	8, 9, 16, 20, 21, 51, 56, 59, 68, 74	7, 13, 28, 30, 41, 47, 69	1, 5, 1011, 12, 13, 15, 19, 25, 31, 32, 33, 35, 40, 41, 42, 45, 46, 55, 62, 63, 64, 70, 73, 74, 76, 83, 84, 85, 87, 88, 89, 91		52

The results clearly showed that none of the WRKY TF group members of one plant species matched with those of other species. These findings indicate that no previous research groups followed specific and proper principles to name and group the WRKY TFs in plants. The number indicates the name of the WRKY TF for example 1, 2, and others indicate WRKY1, WRKY2, and so on).

**Table 9:** Gene duplication analysis of novel WRKY TFs identified during this study.

Figure 1	Genes	Z-score	Percentage of confidence Level
A	GrWRKY12	8.12	100
B	AcWRKY1	6.7	100
C	LuWRKY3-5	4.42	100
D	BrWRKY36-2	8.22	100
E	FvWRKY59	20.58	100
F	PhWRKY59	19.82	100
G	BrWRKY58-1	25.80	100
H	AtWRKY19	25.98	100
I	OsWRKY57	8.43	100
J	FvWRKY52	15.34	100
K	FvWRKY70-7	18.80	100
L	FvWRKY16	6.67	100
M	SbWRKY59	1.78	92.5
N	AlWRKY16	12.53	100
O	PvWRKY94-1	1.11	73.2
P	SiWRKY59-2	1.11	73.4

The result showed that SbWRKY29, PvWRKY94-1 and SiWRKY59-2 are non duplicated WRKY TFs. A z-score value above four is considered duplicated, whereas a value below four was considered nonduplicated. The duplication analysis was performed as described in Pinda (pipeline for intraspecies duplication analysis)^81^.
