# Supplementary material for: Novel Genomic and Evolutionary Insight of WRKY Transcription Factors in Plant Lineage
Source: Sci Rep. 2016 Nov 17;6:37309. doi: 10.1038/srep37309 (PMC5112548; doi:10.1038/srep37309)

Novel Genomic and Evolutionary Insight of WRKY Transcription Factor in Plant Lineage

Tapan Kumar Mohanta[1]*, Yong-Hwan Park[2], Hanhong Bae[2*]

[1]Free Major of Natural Sciences, College of Basic Studies, Yeungnam University, Gyeongsan, 38541, Republic of Korea

[2]School of Biotechnology, Yeungnam University, Gyeongsan, 38541, Republic of Korea.

*Correspondence

Tapan Kumar Mohanta, Email: nostoc.tapan@gmail.com

Hanhong Bae, Email: hanhongbae@ynu.ac.kr, Fax: +82-53-810-4769

Supplementary Table 1

Table showing WRKY genes for which nomenclature was applied, locus IDs and other genomic information regarding plant WRKY transcription factors. The WRKY transcription factor data were downloaded from the Arabidopsis Information Resources (http://www.arabidopsis.org/), the rice genome annotation project (http://rice.plantbiology.msu.edu/), the phytozome database (www.phytozome.net) and the JGI genome portal (http://genome.jgi.doe.gov/) [1–4].

WRKY Gene Name	Locus ID	ORF Length	No. of Amino acids	No. of Introns	3'-5' Coordinates	HMMER E-value
Aquilegia coerulea						
AcWRKY1	Aquca_005_00232	2949	982	6	scaffold_5: 2361204 - 2368192	6.5e-115
AcWRKY3	Aquca_002_00721	1614	537	3	scaffold_2: 5595436 - 5599993	8.6e-197
AcWRKY5	Aquca_053_00058	672	223	3	scaffold_53: 778849 - 782435	2.8e-93
AcWRKY7-1	Aquca_007_00730	1176	391	2	scaffold_7: 5707232 - 5709600	1.4e-144
AcWRKY7-2	Aquca_083_00093	708	235	2	scaffold_83: 748397 - 750150	4.6e-61
AcWRKY8	Aquca_022_00127	987	328	2	scaffold_22: 1355618 - 1357672	3.8e-102
AcWRKY9	Aquca_023_00066	1479	492	4	scaffold_23: 2154836 - 2156885	9.7e-113
AcWRKY11	Aquca_024_00171	1071	356	2	scaffold_24: 3887234 - 3888839	5.3e-129
AcWRKY14	Aquca_030_00369	1386	461	2	scaffold_30: 2694262 - 2697065	2.9e-123
AcWRKY20	Aquca_005_00231	2004	667	5	scaffold_5: 2344028 - 2352075	1.9e-189
AcWRKY21-1	Aquca_049_00056	1143	380	3	scaffold_49: 638305 - 641475	2.6e-137
AcWRKY21-2	Aquca_049_00050	1080	359	2	scaffold_49: 587590 - 591270	1.9e-137
AcWRKY22	Aquca_013_00193	1047	348	2	scaffold_13: 1237691 - 1239032	3.4e-93
AcWRKY26	Aquca_005_00209	1179	592	4	scaffold_5: 2129227 - 2132102	2.8e-249
AcWRKY31	Aquca_029_00028	1005	335	2	scaffold_29: 445844 - 447044	4.9e-109
AcWRKY32	Aquca_016_00233	1413	470	3	scaffold_16: 3223392 - 3227803	7.5e-96
AcWRKY40	Aquca_016_00214	924	307	3	scaffold_16: 3100907 - 3102328	3.2e-103
AcWRKY41	Aquca_013_00135	1041	346	2	scaffold_13: 908760 - 910875	2.6e-104
AcWRKY42	Aquca_066_00022	1992	663	5	scaffold_66: 367318 - 370158	1.4e-209
AcWRKY43	Aquca_013_00218	624	207	1	scaffold_13: 1385362 - 1386568	7.1e-66
AcWRKY44	Aquca_027_00161	1497	498	6	scaffold_27: 1528215 - 1532593	2.9e-162

AcWRKY45-1	Aquca_004_00724	2070	689	11	scaffold_4: 8716518 - 8724909	2.9e-95
AcWRKY45-2	Aquca_058_00185	2478	825	4	scaffold_58: 1137106 - 1141693	1.9e-236
AcWRKY48	Aquca_003_00498	915	304	2	scaffold_3: 6711681 - 6713059	2.1e-79
AcWRKY49	Aquca_003_00104	939	312	2	scaffold_3: 1976003 - 1978372	2.1e-81
AcWRKY55	Aquca_030_00182	1137	378	2	scaffold_30: 1293879 - 1295332	3.0e-69
AcWRKY57	Aquca_002_00789	933	310	3	scaffold_2: 5992053 - 5996417	1.1e-99
AcWRKY65-1	Aquca_021_00165	879	292	2	scaffold_21: 3682765 - 3684408	9.9e-78
AcWRKY65-2	Aquca_039_00057	843	280	2	scaffold_39: 626530 - 628067	2.4e-84
AcWRKY70	Aquca_027_00037	996	331	2	scaffold_27: 668402 - 670774	3.7e-71
AcWRKY72-1	Aquca_002_00516	1488	495	4	scaffold_2: 4256210 - 4258627	7.3e-109
AcWRKY72-2	Aquca_074_00051	1839	612	6	scaffold_74: 624390 - 627193	1.1e-169
AcWRKY75	Aquca_011_00107	522	173	1	scaffold_11: 2178385 - 2179954	1.5e-78
Arabidopsis lyrata						
AlWRKY1	905826	426	141	0	scaffold_5: 12577179 - 12577644	1.7e-274
AlWRKY2	950284	2061	686	3	scaffold_8: 17407377 - 17409738	0.0e+00
AlWRKY3	484242	1533	510	6	scaffold_5: 1784141 - 1786793	0.0e+00
AlWRKY4	471557	1533	510	3	scaffold_1: 5858986 - 5861764	0.0e+00
AlWRKY5-1	483604	657	218	3	scaffold_4: 21798124 - 21799797	1.5e-144
AlWRKY5-2	891177	438	145	1	scaffold_1: 17172574 - 17173151	2.3e-28
AlWRKY5-3	916062	441	146	0	scaffold_7: 21486253 - 21486733	2.3e-186
AlWRKY6	475116	1662	553	7	scaffold_2: 1850703 - 1853262	0.0e+00
AlWRKY7	945679	1050	349	2	scaffold_7: 7689928 - 7691472	1.5e-233
AlWRKY8	494337	981	326	2	scaffold_8: 1043997 - 1046808	1.2e-219
AlWRKY9	908362	1083	360	5	scaffold_2: 12827437 - 12829301	9.9e-218
AlWRKY10	337643	1488	495	4	scaffold_1: 29398224 - 29400455	0.0e+00
AlWRKY11	944827	1008	335	2	scaffold_7: 4295876 - 4297317	3.3e-224
AlWRKY13	490719	912	303	2	scaffold_7: 549246 - 551666	8.3e-186
AlWRKY14	922132	1278	425	2	scaffold_1: 13788182 - 13790713	1.3e-288
AlWRKY15	343891	945	314	2	scaffold_4: 1770885 - 1772017	3.2e-203
AlWRKY16	915648	4014	1337	7	scaffold_8: 2623905 - 2628796	0.0e+00
AlWRKY17	481313	960	319	3	scaffold_4: 2987446 - 2989377	2.5e-211
AlWRKY18	328547	1827	608	6	scaffold_7: 4175818 - 4179180	6.0e-156
AlWRKY20	945405	1686	561	5	scaffold_7: 6573477 - 6576989	0.0e+00

AlWRKY21-1	490447	903	300	2	scaffold_6: 24436095 - 24437666	7.3e-200
AlWRKY21-2	933561	1113	370	2	scaffold_4: 14265900 - 14267352	1.4e-246
AlWRKY23	902782	1014	337	2	scaffold_4: 22982627 - 22983989	1.4e-192
AlWRKY25	481938	1185	394	4	scaffold_4: 14076363 - 14078173	6.7e-269
AlWRKY26	325325	948	315	4	scaffold_6: 2713980 - 2715390	1.0e-216
AlWRKY27	495347	1098	365	4	scaffold_8: 15297318 - 15298864	1.1e-205
AlWRKY28	493050	960	319	3	scaffold_7: 11003793 - 11005263	1.5e-212
AlWRKY29	492514	912	303	2	scaffold_7: 8021855 - 8023128	1.3e-201
AlWRKY30	489268	915	304	3	scaffold_6: 10382551 - 10384147	1.7e-186
AlWRKY31	329411	1617	538	5	scaffold_7: 8829916 - 8832881	0.0e+00
AlWRKY32	328625	2313	770	10	scaffold_7: 4556025 - 4560558	7.0e-257
AlWRKY33	482857	1518	505	4	scaffold_4: 18691660 - 18694027	0.0e+00
AlWRKY34	329038	1707	568	3	scaffold_7: 6670741 - 6672708	0.0e+00
AlWRKY35	482446	1251	416	3	scaffold_4: 16563642 - 16566286	5.0e-233
AlWRKY36	339216	1149	382	3	scaffold_2: 14011565 - 14013515	2.1e-253
AlWRKY38	941764	879	292	2	scaffold_6: 9443043 - 9444230	1.4e-197
AlWRKY39	477731	999	332	3	scaffold_3: 1606487 - 1608449	1.7e-225
AlWRKY40	477161	909	302	3	scaffold_2: 18850430 - 18852249	1.5e-193
AlWRKY41	489946	942	313	2	scaffold_6: 20734835 - 20736495	1.2e-213
AlWRKY42	327396	1590	529	5	scaffold_6: 22370718 - 22373413	0.0e+00
AlWRKY43	322266	342	113	1	scaffold_4: 22403749 - 22404189	5.1e-74
AlWRKY44	482718	1302	433	6	scaffold_4: 17813521 - 17815687	6.6e-265
AlWRKY45	927555	489	162	1	scaffold_3: 41924 - 42520	4.1e-81
AlWRKY46-1	483794	882	293	5	scaffold_4: 22508397 - 22509686	3.1e-172
AlWRKY46-2	324601	816	271	2	scaffold_5: 20404546 - 20405556	4.6e-79
AlWRKY47	490404	1470	489	8	scaffold_6: 24156253 - 24160169	0.0e+00
AlWRKY48	949445	1191	396	2	scaffold_8: 13140161 - 13142158	7.8e-249
AlWRKY49-1	497253	818	272	2	scaffold_97: 8093 - 9280	1.6e-187
AlWRKY49-2	952633	939	312	2	scaffold_97: 2180 - 3433	6.1e-181
AlWRKY49-3	948846	939	312	2	scaffold_8: 4545444 - 4546697	6.1e-181
AlWRKY49-4	915921	1401	466	5	scaffold_7: 20685830 - 20687752	3.2e-185
AlWRKY49-5	948847	861	286	3	scaffold_8: 4551370 - 4552589	2.7e-139
AlWRKY50	351629	522	173	2	scaffold_6: 11899118 - 11899990	2.1e-112

AlWRKY51	496697	585	194	2	scaffold_8: 21683217 - 21684558	3.5e-128
AlWRKY52-1	915663	4083	1360	6	scaffold_8: 2768079 - 2773021	0.0e+00
AlWRKY52-2	915586	3783	1260	7	scaffold_8: 2126189 - 2132181	0.0e+00
AlWRKY53-1	492487	981	326	4	scaffold_7: 7891901 - 7893536	1.2e-201
AlWRKY54-1	901655	1014	337	2	scaffold_4: 18125715 - 18127447	1.8e-209
AlWRKY54-2	321761	1014	337	2	scaffold_4: 19866070 - 19867810	1.8e-209
AlWRKY55-1	898582	807	268	2	scaffold_5: 17918917 - 17920050	2.6e-65
AlWRKY55-2	321760	900	299	2	scaffold_4: 19862087 - 19864279	9.5e-202
AlWRKY56-1	493831	540	179	2	scaffold_7: 21435648 - 21437232	2.2e-109
AlWRKY56-2	474956	588	195	2	scaffold_2: 541114 - 543135	1.8e-124
AlWRKY57	476056	858	285	9	scaffold_2: 13681176 - 13684797	6.8e-181
AlWRKY58	317082	1407	468	3	scaffold_3: 405325 - 407429	5.1e-250
AlWRKY59	320036	609	202	2	scaffold_4: 566403 - 568287	2.3e-137
AlWRKY60	481352	816	271	7	scaffold_4: 3596130 - 3598006	3.3e-177
AlWRKY61	335144	1449	482	2	scaffold_1: 7975906 - 7977915	7.9e-260
AlWRKY62	892017	1059	352	1	scaffold_1: 26545448 - 26546643	2.5e-159
AlWRKY64	908152	726	241	2	scaffold_2: 11324804 - 11326123	1.9e-128
AlWRKY65	336031	780	259	1	scaffold_1: 12903516 - 12904393	3.7e-173
AlWRKY67	338870	705	234	2	scaffold_2: 11328558 - 11329667	6.2e-119
AlWRKY68	486738	807	268	5	scaffold_5: 20781766 - 20783610	1.2e-132
AlWRKY69	486325	816	271	2	scaffold_5: 19001767 - 19003563	2.6e-182
AlWRKY70	486080	897	298	5	scaffold_5: 17921880 - 17923430	3.4e-202
AlWRKY71	313633	846	281	2	scaffold_1: 13155154 - 13156374	2.9e-191
AlWRKY72	940996	1668	555	3	scaffold_6: 6153760 - 6156176	0.0e+00
AlWRKY74	489657	993	330	4	scaffold_6: 13917601 - 13918818	1.3e-201
AlWRKY75	488124	441	146	1	scaffold_6: 5241509 - 5242850	4.9e-95
Arabidopsis thaliana						
AtWRKY1	AT2G04880	1464	487	3	Chr2: 1717888-1720526	0.0e+00
AtWRKY2	AT5G56270	2064	687	4	Chr5: 22779693-22783268	0.0e+00
AtWRKY3	AT2G03340	1542	513	3	Chr2: 1014350-1017148	0.0e+00
AtWRKY4	AT1G13960	1545	514	3	Chr1: 4776463-4779400	0.0e+00
AtWRKY5	AT2G44745	657	218	3	Chr2: 18447273-18449009	4.8e-147
AtWRKY6	AT1G62300	1662	553	5	Chr1: 23016683-23019279	0.0e+00

AtWRKY7	AT4G24240	1062	353	2	Chr4: 12571766-12573725	1.7e-235
AtWRKY8	AT5G46350	981	326	2	Chr5: 18801218-18804043	4.6e-219
AtWRKY9	AT1G68150	1120	374	4	Chr1: 25543969-25545717	2.7e-246
AtWRKY10	AT1G55600	1458	485	4	Chr1: 20774046-20776294	0.0e+00
AtWRKY11	AT4G31550	978	325	2	Chr4: 15289947-15291539	6.8e-217
AtWRKY12	AT3G32090	549	182	3	Chr3: 13078956-13079921	1.0e-118
AtWRKY13	AT4G39410	915	304	2	Chr4: 18332878-18334789	9.8e-206
AtWRKY14	AT1G30650	1293	430	2	Chr1: 10868303-10871297	3.8e-293
AtWRKY15	AT2G23320	954	317	2	Chr2: 9924933-9926372	3.3e-203
AtWRKY16	AT5G45050	4119	1372	6	Chr5: 18176914-18181805	0.0e+00
AtWRKY17	AT2G24570	966	321	2	Chr2: 10437398-10439298	5.9e-213
AtWRKY18	AT4G31800	933	310	4	Chr4: 15383201-15385029	3.3e-204
AtWRKY19	AT4G12020	5688	1895	13	Chr4: 7201656-7209648	0.0e+00
AtWRKY20	AT4G26640	1674	557	5	Chr4: 13437071-13440835	0.0e+00
AtWRKY21	AT2G30590	1143	380	2	Chr2: 13033476-13035593	4.7e-251
AtWRKY22	AT4G01250	897	298	2	Chr4: 522604-524216	3.7e-198
AtWRKY23	AT2G47260	1014	337	2	Chr2: 194048820-19407084	1.1e-224
AtWRKY24	AT5G41570	540	179	1	Chr5: 16624214-16625977	1.7e-121
AtWRKY25	AT2G30250	1182	393	4	Chr2: 12903236-12905198	5.2e-267
AtWRKY26	AT5G07100	930	309	4	Chr5: 2204248-2205811	8.6e-212
AtWRKY27	AT5G52830	1047	348	2	Chr5: 21410852-21412340	1.7e-235
AtWRKY28	AT4G18170	957	318	2	Chr4: 10061673-10062841	2.8e-213
AtWRKY29	AT4G23550	915	304	2	Chr4: 12291820-12293095	2.1e-201
AtWRKY30	AT5G24110	912	303	2	Chr5: 8153115-8154709	2.3e-205
AtWRKY31	AT4G22070	1617	538	5	Chr4: 11691378-11694392	0.0e+00
AtWRKY32	AT4G30935	1401	466	4	Chr4: 15051814-15054042	6.2e-309
AtWRKY33	AT2G38470	1560	519	4	Chr2: 16108361-16110766	0.0e+00
AtWRKY34	AT4G26440	1707	568	3	Chr4: 13357596-13359551	0.0e+00
AtWRKY35	AT2G34830	1284	427	2	Chr2: 14693756-14696378	4.0e-285
AtWRKY36	AT1G69810	1164	387	3	Chr1: 26277238-26279118	1.6e-255
AtWRKY38	AT5G22570	870	289	2	Chr5: 7495539-7496784	5.4e-195
AtWRKY39	AT3G04670	993	330	2	Chr3: 1266242-1268350	6.6e-224
AtWRKY40	AT1G80840	909	302	3	Chr1: 30383691-30385499	1.0e-195

AtWRKY41	AT4G11070	942	313	2	Chr4: 6759303-6760794	6.9e-214
AtWRKY42	AT4G04450	1587	528	5	Chr4: 2218379-2221114	0.0e+00
AtWRKY43	AT2G46130	330	109	1	Chr2: 18957226-18957911	7.0e-71
AtWRKY44	AT2G37260	1290	429	4	Chr2: 15644840-15647065	2.8e-289
AtWRKY45	AT3G01970	444	147	1	Chr3: 326397-327412	2.6e-100
AtWRKY46	AT2G46400	888	295	2	Chr2: 19043414-19044826	5.7e-194
AtWRKY47	AT4G01720	1470	489	5	Chr4: 744921-748554	0.0e+00
AtWRKY48	AT5G49520	1200	399	2	Chr5: 20090776-20093346	2.2e-261
AtWRKY49	AT5G43290	825	274	2	Chr5: 17371838-17373201	6.3e-190
AtWRKY50	AT5G26170	522	173	2	Chr5: 9147176-9148128	6.4e-114
AtWRKY51	AT5G64810	585	194	2	Chr5: 25908413-25909803	7.9e-127
AtWRKY52	AT5G45260	3867	1288	6	Chr5: 18326203-18332609	0.0e+00
AtWRKY53	AT4G23810	975	324	1	Chr4: 12392370-12393982	4.0e-220
AtWRKY54	AT2G40750	1041	346	2	Chr2: 17000454-17002468	1.1e-234
AtWRKY55	AT2G40740	879	292	2	Chr2: 16997177-16999277	4.8e-195
AtWRKY56	AT1G64000	588	195	1	Chr1: 23747304-23749053	3.6e-132
AtWRKY57	AT1G69310	864	287	4	Chr1: 26054308-26058105	4.0e-194
AtWRKY58	AT3G01080	1272	423	4	Chr3: 25507-27449	1.1e-293
AtWRKY59	AT2G21900	609	202	2	Chr2: 9334149-933622	7.8e-136
AtWRKY60	AT2G25000	816	271	4	Chr2: 10629737-10631413	2.6e-177
AtWRKY61	AT1G18860	1443	480	2	Chr1: 6509492-6511227	0.0e+00
AtWRKY62	AT5G01900	792	263	1	Chr5: 351008-352069	2.5e-178
AtWRKY63	AT1G66600	726	241	2	Chr1: 24848320-24849364	4.4e-164
AtWRKY64	AT1G66560	750	249	2	Chr1: 24833579-24834631	1.3e-169
AtWRKY65	AT1G29280	780	259	1	Chr1: 10236367-10237467	8.0e-174
AtWRKY66	AT1G80590	708	235	2	Chr1: 30296210-30297156	1.4e-161
AtWRKY67	AT1G66550	765	254	2	Chr1: 24828502-24829639	2.7e-175
AtWRKY68	AT3G62340	834	277	2	Chr3: 23069516-23071207	2.1e-190
AtWRKY69	AT3G58710	819	272	2	Chr3: 21715007-21716916	2.1e-180
AtWRKY70	AT3G56400	885	294	2	Chr3: 20908928-2091048	3.8e-199
AtWRKY71	AT1G29860	849	282	2	Chr1: 10454482-10455785	1.9e-189
AtWRKY72	AT5G15130	1647	548	3	Chr5: 4904347-4907094	0.0e+00
AtWRKY74	AT5G28650	993	330	2	Chr5: 10677716-10679204	1.7e-221

AtWRKY75	AT5G13080	438	145	1	Chr5: 4149740-4151150	7.1e-94
Brachypodium distachyon						
BdWRKY1	Bradi2g08620	1902	633	6	Bd2: 6965062 - 6969356	0.0e+00
BdWRKY2	Bradi3g34567	1059	352	1	Bd3: 36924565 - 36925829	2.9e-235
BdWRKY3	Bradi1g08106	945	314	3	Bd1: 5717785 - 5722044	3.3e-204
BdWRKY4	Bradi1g07970	1242	413	3	Bd1: 5619653 - 5623406	8.2e-283
BdWRKY7	Bradi2g18530	732	243	1	Bd2: 16474142 - 16475652	5.3e-156
BdWRKY8	Bradi2g15360	912	303	2	Bd2: 13707176 - 13708809	1.3e-201
BdWRKY9	Bradi2g11170	1542	513	4	Bd2: 9403194 - 9408009	0.0e+00
BdWRKY10	Bradi2g05510	636	211	0	Bd2: 3983627 - 3984262	1.0e-145
BdWRKY11	Bradi2g44090	1029	342	2	Bd2: 44578672 - 44580858	1.3e-236
BdWRKY12	Bradi2g44035	1017	338	2	Bd2: 44527990 - 44529664	2.2e-228
BdWRKY13	Bradi2g49906	903	300	2	Bd2: 49904315 - 49905775	4.5e-201
BdWRKY14	Bradi2g48907	828	275	1	Bd2: 49070580 - 49071586	2.5e-183
BdWRKY15	Bradi2g45480	963	320	2	Bd2: 45857664 - 45858974	2.7e-217
BdWRKY16	Bradi2g45900	1146	381	2	Bd2: 46341174 - 46344108	1.3e-254
BdWRKY17	Bradi2g62130	762	253	2	Bd2: 58924622 - 58926999	5.1e-168
BdWRKY18-1	Bradi2g53495	861	286	2	Bd2: 52632487 - 52636282	6.7e-196
BdWRKY18-2	Bradi4g30370	756	251	3	Bd4: 36119270 - 36124180	1.3e-168
BdWRKY19	Bradi2g15877	681	226	1	Bd2: 14040691 - 14041846	3.7e-148
BdWRKY20	Bradi2g53500	849	282	2	Bd2: 52646076 - 52649410	7.5e-194
BdWRKY21	Bradi2g53520	852	283	1	Bd2: 52664751 - 52666466	1.9e-185
BdWRKY22	Bradi2g53480	282	75	2	Bd2: 52621457 - 52623072	8.9e-47
BdWRKY23-1	Bradi2g49020	669	222	1	Bd2: 49173622 - 49177995	1.4e-152
BdWRKY23-2	Bradi2g19070	717	238	1	Bd2: 16801079 - 16803652	1.3e-155
BdWRKY24	Bradi2g53760	1731	576	3	Bd2: 52860054 - 52862718	0.0e+00
BdWRKY25	Bradi3g18580	921	306	0	Bd3: 17079848 - 17080892	8.0e-196
BdWRKY26	Bradi2g48090	651	216	2	Bd2: 48426948 - 48428121	2.8e-143
BdWRKY28	Bradi1g30870	981	326	1	Bd1: 26329079 - 26330580	7.6e-207
BdWRKY30	Bradi3g39340	1698	565	4	Bd3: 41662537 - 41665191	0.0e+00
BdWRKY32	Bradi3g57710	1680	559	4	Bd3: 57392174 - 57394564	0.0e+00
BdWRKY34	Bradi3g50360	735	244	3	Bd3: 51584113 - 51587575	1.3e-171
BdWRKY35	Bradi5g13090	2187	728	4	Bd5: 16639846 - 16643118	0.0e+00

BdWRKY36	Bradi5g17395	591	196	3	Bd5: 20674827 - 20679112	9.0e-135
BdWRKY37	Bradi5g20290	1200	399	2	Bd5: 23193818 - 23196993	4.6e-268
BdWRKY39	Bradi3g09810	1077	358	2	Bd3: 8007304 - 8009064	4.9e-241
BdWRKY43	Bradi2g16150	1689	562	3	Bd2: 14220256 - 14222873	0.0e+00
BdWRKY45-1	Bradi2g30695	834	277	2	Bd2: 30388047 - 30389483	1.8e-190
BdWRKY45-2	Bradi2g44270	879	292	2	Bd2: 44782012 - 44783510	4.8e-196
BdWRKY46	Bradi4g25717	720	239	2	Bd4: 31073069 - 31076118	2.1e-161
BdWRKY47	Bradi1g17660	984	327	2	Bd1: 14207355 - 14209349	1.0e-215
BdWRKY49	Bradi2g16357	1254	417	2	Bd2: 14427911 - 14430851	1.5e-283
BdWRKY51	Bradi5g04817	936	311	2	Bd5: 6203333 - 6204805	5.5e-206
BdWRKY53-1	Bradi4g01950	1326	441	3	Bd4: 1276705 - 1278729	1.9e-298
BdWRKY53-2	Bradi2g00280	1161	386	3	Bd2: 37729 - 39452	4.2e-263
BdWRKY55	Bradi1g63910	630	209	2	Bd1: 63118295 - 63119746	9.5e-144
BdWRKY57-1	Bradi1g47690	885	294	4	Bd1: 46394072 - 46396455	2.2e-201
BdWRKY57-2	Bradi4g45290	1008	335	4	Bd4: 48418327 - 48421147	1.6e-229
BdWRKY57-3	Bradi1g02327	1011	336	3	Bd1: 1580840 - 1582503	7.6e-231
BdWRKY59	Bradi4g09890	2847	948	4	Bd4: 9350003 - 9357060	0.0e+00
BdWRKY60-1	Bradi1g13207	396	131	2	Bd1: 10018214 - 10019283	1.3e-85
BdWRKY60-2	Bradi2g30800	1032	343	2	Bd2: 30398035 - 30400975	2.2e-230
BdWRKY65	Bradi4g44342	981	326	1	Bd4: 47782844 - 47784664	6.7e-221
BdWRKY66	Bradi3g52420	1371	456	2	Bd3: 53316886 - 53321459	1.0e-312
BdWRKY67	Bradi2g33540	498	165	0	Bd2: 33674702 - 33675549	1.5e-110
BdWRKY68	Bradi5g20700	936	311	2	Bd5: 23482100 - 23483388	7.2e-202
BdWRKY69	Bradi3g34850	927	308	1	Bd3: 37214494 - 37216071	9.4e-208
BdWRKY70	Bradi2g22440	1524	507	2	Bd2: 19960366 - 19962573	0.0e+00
BdWRKY71	Bradi3g06070	1038	345	1	Bd3: 4354442 - 4356174	9.1e-229
BdWRKY72	Bradi4g19060	627	208	1	Bd4: 21655844 - 21659743	2.0e-139
BdWRKY73	Bradi1g51030	1878	625	6	Bd1: 49453669 - 49457672	0.0e+00
BdWRKY74	Bradi4g28280	978	325	2	Bd4: 33637416 - 33639521	4.5e-219
BdWRKY76	Bradi4g30360	1020	339	3	Bd4: 36105434 - 36107137	1.3e-220
BdWRKY78	Bradi1g23340	1818	605	5	Bd1: 18715369 - 18721624	0.0e+00
BdWRKY79	Bradi1g63220	933	310	2	Bd1: 62421625 - 62422845	5.7e-200
BdWRKY80	Bradi4g33370	2073	690	5	Bd4: 39074575 - 39078767	0.0e+00

BdWRKY82	Bradi3g19640	1755	584	3	Bd3: 18514071 - 18520937	0.0e+00
BdWRKY83-1	Bradi4g02680	1062	353	3	Bd4: 1992612 - 1995531	7.0e-235
BdWRKY83-2	Bradi1g14300	1038	345	3	Bd1: 11188970 - 11191904	1.6e-231
BdWRKY85	Bradi4g06690	1506	501	3	Bd4: 5576052 - 5581378	0.0e+00
BdWRKY86	Bradi2g53510	753	250	2	Bd2: 52655598 - 52657803	4.0e-167
BdWRKY87	Bradi1g09170	1266	421	3	Bd1: 6545010 - 6548310	1.5e-282
BdWRKY90-1	Bradi4g44360	756	251	2	Bd4: 47797837 - 47799556	3.4e-166
BdWRKY90-2	Bradi4g44350	780	259	2	Bd4: 47786980 - 47788750	2.5e-172
BdWRKY90-3	Bradi4g44370	819	272	2	Bd4: 47804750 - 47805919	3.5e-183
BdWRKY91	Bradi2g22230	894	297	2	Bd2: 19779563 - 19782548	1.3e-192
BdWRKY92	Bradi2g15405	930	309	2	Bd2: 13729497 - 13730737	6.4e-202
BdWRKY93	Bradi1g48770	1068	355	2	Bd1: 47430434 - 47433797	9.2e-240
BdWRKY96	Bradi1g22680	1344	447	3	Bd1: 18197079 - 18200281	7.6e-310
BdWRKY97	Bradi2g05500	1599	532	2	Bd2: 3979821 - 3981719	0.0e+00
BdWRKY102	Bradi2g05234	681	226	2	Bd2: 3818524 - 3823413	1.1e-153
BdWRKY105	Bradi1g16120	1476	491	3	Bd1: 13043037 - 13049431	0.0e+00
BdWRKY106	Bradi2g54720	864	287	1	Bd2: 53553539 - 53556063	2.3e-195
Brassica rapa						
BrWRKY1	Bra013187	1344	447	3	A03: 19971615 - 19973447	4.1e-294
BrWRKY2-1	Bra028939	1350	449	2	A03: 5507782 - 5509290	4.3e-303
BrWRKY2-2	Bra002828	2022	673	3	A10: 7308456 - 7310721	0.0e+00
BrWRKY3-1	Bra026554	1428	475	3	A02: 20181641 - 20183548	1.2e-30
BrWRKY3-2	Bra024780	1461	486	3	A06: 23398422 - 23400354	0.0e+00
BrWRKY4-1	Bra026869	1443	480	2	A09: 34052814 - 34054790	0.0e+00
BrWRKY4-2	Bra019697	1482	493	3	A06: 4910724 - 4912882	0.0e+00
BrWRKY4-3	Bra016716	1398	465	3	A08: 19652034 - 19653979	1.1e-309
BrWRKY5-1	Bra037637	546	181	3	A04: 18386470 - 18387608	1.5e-119
BrWRKY5-2	Bra004864	648	215	3	A05: 2169762 - 2171241	5.0e-145
BrWRKY5-3	Bra000362	633	210	3	A03: 10731875 - 10733550	5.2e-142
BrWRKY6-1	Bra027057	1659	552	5	A09: 8074041 - 8076449	0.0e+00
BrWRKY6-2	Bra034482	1524	507	6	Scaffold000096: 241928 - 244473	0.0e+00
BrWRKY7-1	Bra013779	432	143	3	A01: 7725525 - 7726849	1.3e-93
BrWRKY7-2	Bra019239	1047	348	3	A03: 25526576 - 25528473	4.6e-232

BrWRKY8-1	Bra022033	954	317	2	A02: 18718294 - 18724024	1.2e-29
BrWRKY8-2	Bra025021	972	323	2	A06: 24733571 - 24736005	8.8e-217
BrWRKY8-3	Bra017561	966	321	2	A09: 16500923 - 16504686	4.2e-246
BrWRKY9-1	Bra033956	2133	710	15	A02: 10623625 - 10628895	0.0e+00
BrWRKY9-2	Bra004285	987	238	4	A07: 17788706 - 17790449	5.9e-214
BrWRKY10-1	Bra030847	1344	447	4	A08: 239211 - 241785	5.2e-309
BrWRKY10-2	Bra038006	1965	654	4	A06: 152241 - 154947	0.0e+00
BrWRKY10-3	Bra037106	1299	432	4	A05: 10124867 - 10126756	1.3e-295
BrWRKY10-4	Bra030848	612	203	3	A08: 251554 - 252566	4.3e-135
BrWRKY11-1	Bra011282	969	322	2	A01: 2951676 - 2953056	7.0e-214
BrWRKY11-2	Bra010231	999	332	2	A08: 13321033 - 13322465	5.4e-220
BrWRKY11-3	Bra023998	987	328	2	A03: 28325809 - 28327190	2.2e-220
BrWRKY13-1	Bra010683	891	296	2	A08: 15966809 - 15969142	3.9e-203
BrWRKY13-2	Bra033616	879	292	2	A06: 25990144 - 25992652	4.2e-200
BrWRKY13-3	Bra011861	897	298	2	A01: 182416 - 184570	5.8e-205
BrWRKY14-1	Bra041029	1224	407	2	Scaffold000396: 12230 - 14468	5.4e-30
BrWRKY14-2	Bra026228	1191	396	2	A01: 10537643 - 10539822	8.5e-267
BrWRKY15	Bra032174	960	319	2	A04: 10336494 - 10337630	6.3e-208
BrWRKY17	Bra032084	978	325	2	A04: 10985430 - 10986845	1.0e-214
BrWRKY18-1	Bra010220	927	308	4	A08: 13246799 - 13248642	7.0e-205
BrWRKY18-2	Bra011299	954	317	4	A01: 2868582 - 2869908	2.6e-205
BrWRKY20-1	Bra026414	1608	535	5	A01: 9431629 - 9434410	0.0e+00
BrWRKY20-2	Bra010431	1638	545	5	A08: 14491517 - 14494585	0.0e+00
BrWRKY20-3	Bra019095	2265	754	12	A03: 26340990 - 26350088	0.0e+00
BrWRKY20-4	Bra028971	1458	485	5	A03: 5651051 - 5653183	0.0e+00
BrWRKY21-1	Bra021652	966	321	2	A04: 13660758 - 13661885	3.8e-214
BrWRKY21-2	Bra018303	1008	335	2	A05: 7312863 - 7314031	1.2e-223
BrWRKY21-3	Bra022808	1017	338	2	A03: 7166015 - 7167214	7.3e-225
BrWRKY22	Bra037368	897	298	2	A09: 846055 - 847193	1.8e-196
BrWRKY23-1	Bra021393	972	323	2	A01: 25404906 - 25406091	2.5e-218
BrWRKY23-2	Bra004466	960	319	2	A05: 274575 - 276116	2.2e-213
BrWRKY24	Bra025490	525	174	1	A04: 8404080 - 8407561	1.2e-119
BrWRKY25-1	Bra018325	1122	373	4	A05: 7467357 - 7468865	6.4e-254

BrWRKY25-2	Bra022786	1137	378	4	A03: 7048063 - 7049645	1.6e-257
BrWRKY25-3	Bra021623	1053	350	4	A04: 13500274 - 13501648	1.2e-235
BrWRKY26	Bra028707	960	319	3	A02: 2443741 - 2445256	4.7e-29
BrWRKY27	Bra022612	1035	344	2	A02: 8534510 - 8535740	1.8e-30
BrWRKY28-1	Bra012614	939	312	2	A03: 23210602 - 23211724	1.9e-208
BrWRKY28-2	Bra013289	927	308	2	A01: 4763091 - 4764234	9.5e-204
BrWRKY28-3	Bra040926	885	294	2	Scaffold000316: 18877 - 20035	6.5e-31
BrWRKY29-1	Bra019297	900	299	2	A03: 25119154 - 25120401	6.8e-199
BrWRKY29-2	Bra020814	927	308	2	A08: 11972081 - 11973603	2.0e-199
BrWRKY29-3	Bra013708	891	296	2	A01: 7304758 - 7306085	2.2e-197
BrWRKY30-1	Bra009734	942	313	2	A06: 16910398 - 16911760	4.0e-211
BrWRKY30-2	Bra026467	939	312	2	A01: 9132853 - 9134359	9.2e-212
BrWRKY31	Bra013584	1617	538	5	A01: 6468156 - 6471174	0.0e+00
BrWRKY32-1	Bra024035	1770	589	4	A03: 28045703 - 28048028	0.0e+00
BrWRKY32-2	Bra010266	1374	457	4	A08: 13536241 - 13538271	4.4e-302
BrWRKY33-1	Bra017117	1392	463	3	A04: 16526854 - 16528558	0.0e+00
BrWRKY33-2	Bra005104	1374	457	4	A05: 3439967 - 3443129	2.6e-309
BrWRKY33-3	Bra000064	1422	473	4	A03: 9129420 - 9131325	4.2e-315
BrWRKY34-1	Bra026438	1605	534	3	A01: 9303958 - 9305798	0.0e+00
BrWRKY34-2	Bra019105	1590	529	3	A03: 26297993 - 26299836	0.0e+00
BrWRKY35-1	Bra021934	1191	396	2	A04: 15267024 - 15269622	4.0e-267
BrWRKY35-2	Bra005388	1212	403	2	A05: 5155344 - 5157950	1.4e-264
BrWRKY36-1	Bra007884	1152	383	3	A02: 11577278 - 11579396	3.6e-29
BrWRKY36-2	Bra016263	2247	748	7	A07: 18564900 - 18568514	0.0e+00
BrWRKY38-1	Bra002421	867	288	2	A10: 9693974 - 9695104	1.2e-195
BrWRKY38-2	Bra020196	855	284	2	A02: 5509636 - 5510789	1.4e-194
BrWRKY38-3	Bra020197	843	280	3	A02: 5515325 - 5516544	3.4e-190
BrWRKY39-1	Bra040396	912	303	2	Scaffold000203: 12865 - 13907	4.4e-204
BrWRKY39-2	Bra001117	960	319	2	A03: 14838129 - 14839229	1.5e-214
BrWRKY40-1	Bra008435	873	290	3	A02: 15608697 - 15610784	8.6e-186
BrWRKY40-2	Bra035148	903	300	3	A07: 22365779 - 22367318	3.2e-193
BrWRKY40-3	Bra003588	894	297	3	A07: 13952832 - 13954369	6.4e-190
BrWRKY41	Bra033158	996	331	2	A02: 17058445 - 17059657	1.4e-228

BrWRKY42-1	Bra000793	1560	519	5	A03: 13151695 - 13154162	0.0e+00
BrWRKY42-2	Bra029491	1479	492	6	A09: 17616128 - 17618992	0.0e+00
BrWRKY44-1	Bra005210	1230	409	3	A05: 4035604 - 4037093	1.0e-275
BrWRKY44-2	Brara.C01871	1263	420	3	A03: 8742927 - 8744451	1.8e-282
BrWRKY45	Bra040557	435	144	1	Scaffold000217: 68236 - 68758	1.4e-30
BrWRKY46-1	Bra004540	858	285	2	A05: 715036 - 716110	4.0e-189
BrWRKY46-2	Bra000423	846	281	2	A03: 11037238 - 11038263	1.3e-24
BrWRKY46-3	Bra014426	834	277	2	A04: 570067 - 571085	4.7e-185
BrWRKY47	Bra036315	1473	490	5	A09: 495154 - 498255	1.5e-26
BrWRKY48-1	Bra020628	1179	392	2	A02: 24110310 - 24112266	2.3e-258
BrWRKY48-2	Bra036138	1062	353	2	A09: 2551608 - 2553378	4.8e-232
BrWRKY48-3	Bra010032	1200	399	2	A06: 18743149 - 18745198	3.4e-265
BrWRKY49	Bra027480	987	238	2	A09: 13999956 - 14001207	4.0e-221
BrWRKY50-1	Bra020546	507	168	2	A02: 24663990 - 24665001	1.2e-29
BrWRKY50-2	Bra036563	477	158	2	A09: 1779667 - 1780630	1.4e-101
BrWRKY50-3	Bra009885	531	176	2	A06: 17889671 - 17890550	9.2e-117
BrWRKY51-1	Bra031900	594	197	2	A02: 27215815 - 27216988	2.5e-132
BrWRKY51-2	Bra024328	621	206	2	A06: 15006857 - 15008072	5.4e-139
BrWRKY51-3	Bra037796	507	168	2	A09: 3630921 - 3631940	6.1e-111
BrWRKY53-1	Bra013732	951	316	2	A01: 7481180 - 7482306	5.9e-214
BrWRKY53-2	Bra019265	972	323	2	A03: 25365665 - 25366833	2.1e-220
BrWRKY54-1	Bra016974	993	330	2	A04: 17312303 - 17313823	5.3e-224
BrWRKY54-2	Bra000203	1023	340	2	A03: 9851591 - 9853154	1.1e-231
BrWRKY54-3	Bra004586	1020	339	2	A05: 898887 - 900534	2.0e-233
BrWRKY54-4	Bra008456	747	248	2	A02: 15741116 - 15742088	9.3e-17
BrWRKY54-5	Bra008459	777	258	2	A02: 15758758 - 15759687	1.0e-26
BrWRKY55-1	Bra016975	855	284	2	A04: 17308786 - 17310419	1.8e-194
BrWRKY55-2	Bra000202	903	300	2	A03: 9847657 - 9849612	1.7e-206
BrWRKY55-3	Bra014693	795	264	2	A04: 2291755 - 2293716	2.8e-180
BrWRKY56-1	Bra027768	564	187	1	A09: 6335477 - 6336522	1.4e-126
BrWRKY56-2	Bra027675	570	189	1	A09: 6983636 - 6988361	2.4e-128
BrWRKY57-1	Bra004370	885	294	2	A07: 18299620 - 18301569	9.1e-198
BrWRKY57-2	Bra004002	870	289	2	A07: 16234546 - 16236403	4.3e-191

BrWRKY57-3	Bra038313	900	299	2	A02: 11335646 - 11337496	4.1e-199
BrWRKY58-1	Bra039147	3249	1082	11	A05: 23763093 - 23768561	4.3e-28
BrWRKY58-2	Bra001023	3195	1064	10	A03: 14419206 - 14424267	5.3e-23
BrWRKY59-1	Bra031221	594	197	2	A09: 31958040 - 31959294	6.7e-135
BrWRKY59-2	Bra030273	591	196	2	A04: 9440773 - 9442655	1.2e-131
BrWRKY60-1	Bra019123	585	194	3	A03: 26212225 - 26213388	3.1e-128
BrWRKY60-2	Bra023983	1038	345	4	A03: 28445361 - 28447835	9.8e-228
BrWRKY61-1	Bra016535	1425	474	2	A08: 18921284 - 18923182	1.9e-313
BrWRKY61-2	Bra031053	1413	470	2	A09: 32729063 - 32730858	1.8e-198
BrWRKY61-3	Bra025693	864	287	1	A06: 7337785 - 7338754	3.4e-186
BrWRKY62-1	Bra023211	777	258	2	A09: 20688619 - 20689569	3.1e-173
BrWRKY62-2	Bra028918	873	290	2	A02: 1398534 - 1399579	1.2e-312
BrWRKY65-1	Bra010839	786	261	1	A08: 16731567 - 16732487	2.5e-174
BrWRKY65-2	Bra032300	789	262	1	A09: 22733156 - 22734048	4.1e-175
BrWRKY66	Bra008454	597	198	3	A02: 15731941 - 15732891	3.5e-25
BrWRKY68	Bra007674	897	298	2	A09: 30354013 - 30355104	2.6e-204
BrWRKY69-1	Bra007413	813	270	2	A09: 29067821 - 29069232	2.5e-182
BrWRKY69-2	Bra014556	837	278	2	A04: 1434942 - 1436495	5.0e-188
BrWRKY70-1	Bra014692	858	285	2	A04: 2286661 - 2287981	3.9e-197
BrWRKY70-2	Bra003239	852	283	2	A07: 12088289 - 12089820	5.9e-192
BrWRKY70-3	Bra007243	831	276	2	A09: 28166831 - 28168237	9.9e-188
BrWRKY71-1	Bra032340	822	273	2	A09: 22427782 - 22428867	1.5e-183
BrWRKY71-2	Bra030178	834	277	2	A07: 5904814 - 5906274	6.7e-192
BrWRKY72-1	Bra006283	1584	527	3	A03: 2840808 - 2843014	0.0e+00
BrWRKY72-2	Bra008719	1656	551	3	A10: 13554962 - 13557389	0.0e+00
BrWRKY72-3	Bra023505	1551	516	3	A02: 3670275 - 3672453	2.6e-31
BrWRKY72-4	Bra040505	1608	535	3	A01: 28592291 - 28594507	0.0e+00
BrWRKY74-1	Bra020617	1014	337	2	A02: 24185557 - 24186711	5.3e-226
BrWRKY74-2	Bra010005	1026	341	2	A06: 18593112 - 18594661	4.7e-233
BrWRKY75-1	Bra008858	444	147	1	A10: 14163515 - 14164515	2.5e-94
BrWRKY75-2	Bra006178	444	147	1	A03: 2366855 - 2367892	2.3e-94
Capsella rubella						
CrWRKY1	Carubv10013546m.g	1470	489	4	scaffold_3: 9903832 - 9908690	0.0e+00

CrWRKY2	Carubv10026001m.g	2076	691	4	scaffold_8: 8879381 - 8883328	0.0e+00
CrWRKY3	Carubv10017039m.g	1542	513	3	scaffold_5: 963455 - 966061	1.5e-279
CrWRKY4	Carubv10008859m.g	1566	521	3	scaffold_1: 4857209 - 4860288	0.0e+00
CrWRKY5	Carubv10023987m.g	699	232	3	scaffold_4: 13666550 - 13668364	4.8e-158
CrWRKY6	Carubv10020051m.g	1725	574	5	scaffold_2: 1161926 - 1164495	0.0e+00
CrWRKY7	Carubv10005147m.g	1059	352	2	scaffold_7: 6264738 - 6266985	1.6e-233
CrWRKY8	Carubv10026746m.g	1011	336	2	scaffold_8: 597428 - 600123	2.6e-227
CrWRKY9	Carubv10021479m.g	1050	349	4	scaffold_2: 8918432 - 8919978	7.7e-232
CrWRKY10	Carubv10011619m.g	1542	513	4	scaffold_1: 19328797 - 19331203	0.0e+00
CrWRKY11	Carubv10005215m.g	999	332	2	scaffold_7: 3441010 - 3442782	6.7e-222
CrWRKY13	Carubv10005178m.g	1032	343	2	scaffold_7: 482554 - 485403	6.8e-233
CrWRKY14	Carubv10009191m.g	1299	342	2	scaffold_1: 10632445 - 10635121	9.8e-294
CrWRKY15	Carubv10023662m.g	957	318	2	scaffold_4: 1042867 - 1044448	1.2e-204
CrWRKY16	Carubv10025744m.g	4083	1360	6	scaffold_8: 1365382 - 1370221	0.0e+00
CrWRKY17	Carubv10023561m.g	1032	343	2	scaffold_4: 1807089 - 1808584	2.1e-229
CrWRKY18	Carubv10005349m.g	909	302	4	scaffold_7: 3347638 - 3349514	1.9e-198
CrWRKY20-1	Carubv10004390m.g	1848	615	6	scaffold_7: 5347702 - 5352521	0.0e+00
CrWRKY20-2	Carubv10027981m.g	1689	562	5	scaffold_8: 8634914 - 8637711	0.0e+00
CrWRKY21	Carubv10023424m.g	1146	381	2	scaffold_4: 7740465 - 7742613	2.3e-255
CrWRKY22	Carubv10001446m.g	981	326	2	scaffold_6: 16254206 - 16255961	4.1e-217
CrWRKY23	Carubv10023565m.g	1026	341	2	scaffold_4: 14745207 - 14746811	9.1e-230
CrWRKY24	Carubv10006263m.g	549	182	1	scaffold_7: 15476709 - 15477672	2.3e-122
CrWRKY25	Carubv10024687m.g	1173	390	4	scaffold_4: 7590469 - 7592097	5.0e-264
CrWRKY27	Carubv10026599m.g	1134	377	2	scaffold_8: 7489184 - 7490847	1.9e-256
CrWRKY28	Carubv10005249m.g	972	323	2	scaffold_7: 8751223 - 8753033	3.5e-217
CrWRKY29	Carubv10005155m.g	1056	351	4	scaffold_7: 6646263 - 6648310	7.4e-237
CrWRKY30	Carubv10001559m.g	915	304	2	scaffold_6: 8176565 - 8178023	4.0e-209
CrWRKY31	Carubv10004543m.g	1623	540	6	scaffold_7: 7218079 - 7222785	0.0e+00
CrWRKY32	Carubv10006890m.g	1509	502	4	scaffold_7: 3674663 - 3676878	0.0e+00
CrWRKY33	Carubv10023006m.g	1569	522	4	scaffold_4: 11167415 - 11170428	0.0e+00
CrWRKY34	Carubv10007808m.g	1587	528	3	scaffold_7: 5432917 - 5434780	0.0e+00
CrWRKY35	Carubv10023228m.g	1323	440	2	scaffold_4: 9617920 - 9620757	1.1e-294
CrWRKY36	Carubv10022409m.g	1164	387	3	scaffold_2: 9732847 - 9734811	2.9e-256

CrWRKY38	Carubv10002472m.g	873	290	2	scaffold_6: 7524053 - 7525258	2.2e-197
CrWRKY39	Carubv10015434m.g	993	330	2	scaffold_3: 1270000 - 1271182	2.3e-223
CrWRKY40	Carubv10020726m.g	891	296	5	scaffold_2: 13713965 - 13717378	1.9e-148
CrWRKY41	Carubv10001458m.g	972	323	2	scaffold_6: 13541328 - 13543386	2.7e-222
CrWRKY42	Carubv10000703m.g	1578	525	5	scaffold_6: 14602467 - 14605216	0.0e+00
CrWRKY44	Carubv10023273m.g	1281	426	4	scaffold_4: 10666173 - 10668542	1.3e-287
CrWRKY45	Carubv10016335m.g	450	149	1	scaffold_3: 40481 - 41037	6.0e-101
CrWRKY46-1	Carubv10023578m.g	1011	336	2	scaffold_4: 14349812 - 14351193	1.9e-220
CrWRKY46-2	Carubv10019489m.g	876	291	2	scaffold_5: 13062694 - 13063784	8.7e-192
CrWRKY47	Carubv10000809m.g	1482	493	5	scaffold_6: 15929489 - 15932717	0.0e+00
CrWRKY48	Carubv10026463m.g	1275	424	2	scaffold_8: 5969032 - 5971300	4.7e-281
CrWRKY49	Carubv10026841m.g	918	305	2	scaffold_8: 2385348 - 2386532	1.1e-207
CrWRKY50	Carubv10002099m.g	522	173	2	scaffold_6: 9111908 - 9113611	5.1e-112
CrWRKY51	Carubv10027178m.g	585	194	2	scaffold_8: 12283805 - 12285414	5.5e-129
CrWRKY52	Carubv10025742m.g	4236	1411	6	scaffold_8: 1163667 - 1169265	0.0e+00
CrWRKY53	Carubv10006884m.g	957	318	1	scaffold_7: 6460067 - 6461117	2.8e-217
CrWRKY54	Carubv10024972m.g	885	294	3	scaffold_4: 12141619 - 12143180	3.6e-200
CrWRKY55-1	Carubv10023773m.g	870	289	2	scaffold_4: 12137702 - 12140014	3.0e-194
CrWRKY55-2	Carubv10017859m.g	786	261	2	scaffold_5: 11149025 - 11150970	5.5e-180
CrWRKY56	Carubv10022129m.g	585	194	1	scaffold_2: 345364 - 346538	2.5e-131
CrWRKY57	Carubv10020765m.g	840	279	2	scaffold_2: 9464403 - 9466127	1.8e-187
CrWRKY58	Carubv10013623m.g	1410	469	3	scaffold_3: 344558 - 346942	0.0e+00
CrWRKY59	Carubv10025063m.g	603	200	2	scaffold_4: 332760 - 334591	8.5e-138
CrWRKY60	Carubv10023876m.g	783	260	4	scaffold_4: 2080266 - 2082230	1.4e-172
CrWRKY62	Carubv10012720m.g	795	264	1	scaffold_24: 38651 - 39611	1.4e-178
CrWRKY61	Carubv10008977m.g	1464	487	2	scaffold_1: 6548436 - 6550262	0.0e+00
CrWRKY63	Carubv10022120m.g	663	220	2	scaffold_2: 3955705 - 3956684	1.2e-149
CrWRKY64	Carubv10021899m.g	744	247	2	scaffold_2: 8006794 - 8007750	7.4e-172
CrWRKY65	Carubv10010013m.g	810	269	1	scaffold_1: 10003548 - 10004843	7.0e-179
CrWRKY66	Carubv10021420m.g	693	230	2	scaffold_2: 8019137 - 8020072	6.1e-157
CrWRKY67	Carubv10021860m.g	684	227	2	scaffold_2: 8011455 - 8012388	2.2e-158
CrWRKY68	Carubv10019242m.g	879	292	2	scaffold_5: 13334589 - 13335750	1.3e-198
CrWRKY69	Carubv10017819m.g	825	274	2	scaffold_5: 12001127 - 12003911	1.8e-184

CrWRKY70	Carubv10017349m.g	1239	412	2	scaffold_5: 11152805 - 11154527	1.5e-279
CrWRKY71	Carubv10009934m.g	858	285	2	scaffold_1: 10242856 - 10244159	9.3e-194
CrWRKY72	Carubv10000606m.g	1677	558	3	scaffold_6: 4949529 - 4952204	0.0e+00
CrWRKY74	Carubv10001454m.g	975	324	2	scaffold_6: 10413373 - 10414784	1.4e-215
CrWRKY75	Carubv10002187m.g	459	152	1	scaffold_6: 4149186 - 4150378	6.5e-100
Carica papaya						
CpWRKY1	evm.TU.supercontig_136.16	1167	388	3	supercontig_136: 206315 - 207886	7.5e-125
CpWRKY2	evm.TU.supercontig_12.58	2244	747	4	supercontig_12: 485616 - 488901	0.0e+00
CpWRKY3	evm.TU.supercontig_14.134	294	97	0	supercontig_14: 1611277 - 1611570	2.4e-54
CpWRKY5	evm.TU.supercontig_22.34	578	193	3	supercontig_22: 818084 - 820697	1.9e-82
CpWRKY6-1	evm.TU.supercontig_152.36	1899	632	4	supercontig_152: 269384 - 272002	2.6e-209
CpWRKY6-2	evm.TU.supercontig_87.103	1857	618	4	supercontig_87: 670547 - 672817	1.6e-240
CpWRKY7	evm.TU.supercontig_21.159	1131	376	2	supercontig_21: 1622493 - 1624174	1.8e-161
CpWRKY11	evm.TU.supercontig_768.1	1017	338	2	supercontig_768: 2106 - 3934	1.3e-168
CpWRKY12	evm.TU.supercontig_2.29	1128	375	3	supercontig_2: 580656 - 582323	3.2e-60
CpWRKY13	evm.TU.supercontig_72.18	648	215	2	supercontig_72: 405689 - 412913	1.7e-80
CpWRKY15	evm.TU.supercontig_55.104	1092	363	2	supercontig_55: 632234 - 633633	1.7e-147
CpWRKY18	evm.TU.supercontig_127.25	1065	354	2	supercontig_127: 422032 - 423346	9.3e-88
CpWRKY20	evm.TU.supercontig_12.196	1269	422	5	supercontig_12: 1751070 - 1756934	1.2e-185
CpWRKY22	evm.TU.supercontig_184.33	897	298	2	supercontig_184: 394065 - 395369	7.2e-84
CpWRKY23	evm.TU.supercontig_18.82	966	321	2	supercontig_18: 548927 - 550235	4.4e-108
CpWRKY27	evm.TU.supercontig_107.128	1176	391	2	supercontig_107: 687517 - 688860	3.6e-71
CpWRKY29	evm.TU.supercontig_180.9	966	321	2	supercontig_180: 123327 - 124517	7.2e-75
CpWRKY32	evm.TU.supercontig_46.151	990	329	2	supercontig_46: 1518234 - 1522477	3.1e-109
CpWRKY33-1	evm.TU.supercontig_5.248	1749	582	4	supercontig_5: 2162660 - 2165047	3.5e-235
CpWRKY33-2	evm.TU.supercontig_2.345	1830	609	4	supercontig_2: 4002808 - 4005630	2.5e-272
CpWRKY35-1	evm.TU.supercontig_18.56	807	268	2	supercontig_18: 399883 - 401123	2.9e-85
CpWRKY35-2	evm.TU.supercontig_2011.1	1411	471	2	supercontig_2011: 7464 - 10808	2.9e-113
CpWRKY38	evm.TU.supercontig_86.65	534	177	2	supercontig_86: 834868 - 835555	2.2e-42
CpWRKY39	evm.TU.supercontig_52.143	1050	349	2	supercontig_52: 1335130 - 1337065	2.1e-184
CpWRKY40-1	evm.TU.supercontig_10.75	921	306	4	supercontig_10: 1268958 - 1271020	6.2e-75
CpWRKY40-2	evm.TU.supercontig_127.27	882	293	3	supercontig_127: 458779 - 460471	2.9e-67
CpWRKY42	evm.TU.supercontig_684.1	1605	534	5	supercontig_684: 5486 - 8518	1.0e-124

CpWRKY44	evm.TU.supercontig_114.62	1395	464	4	supercontig_114: 806190 - 808122	6.6e-203
CpWRKY46	evm.TU.contig_32256	1062	353	2	contig_32256: 706 - 2593	4.1e-121
CpWRKY47	evm.TU.supercontig_57.21	1473	490	5	supercontig_57: 356699 - 359700	8.1e-191
CpWRKY48	evm.TU.supercontig_9.36	1182	393	2	supercontig_9: 151328 - 152907	1.9e-102
CpWRKY49	evm.TU.supercontig_169.18	381	126	1	supercontig_169: 259526 - 260026	8.0e-28
CpWRKY50	evm.TU.supercontig_126.49	540	179	2	supercontig_126: 512086 - 514000	6.7e-51
CpWRKY51	evm.TU.supercontig_919.2	432	143	2	supercontig_919: 20754 - 22643	7.8e-52
CpWRKY53-1	evm.TU.supercontig_3.53	1125	374	2	supercontig_3: 312632 - 314116	1.8e-115
CpWRKY53-2	evm.TU.supercontig_79.60	1053	350	2	supercontig_79: 1185551 - 1186820	1.2e-134
CpWRKY56-1	evm.TU.supercontig_11.70	483	160	1	supercontig_11: 730462 - 731171	8.2e-46
CpWRKY56-2	evm.TU.supercontig_180.6	348	115	0	supercontig_180: 105515 - 105862	2.4e-24
CpWRKY55	evm.TU.supercontig_19.45	999	332	2	supercontig_19: 376137 - 377338	3.2e-79
CpWRKY57	evm.TU.supercontig_1.104	942	313	2	supercontig_1: 1701963 - 1705388	2.4e-122
CpWRKY58	evm.TU.supercontig_1.76	1626	541	3	supercontig_1: 1377429 - 1381442	5.8e-216
CpWRKY67	evm.TU.supercontig_86.64	786	261	2	supercontig_86: 829391 - 831277	5.1e-59
CpWRKY69	evm.TU.supercontig_7.92	786	261	2	supercontig_7: 828842 - 829924	7.4e-110
CpWRKY71	evm.TU.supercontig_1195.2	357	118	0	supercontig_1195: 13615 - 13971	1.3e-37
CpWRKY70	evm.TU.supercontig_19.44	861	286	2	supercontig_19: 374219 - 375477	6.8e-75
CpWRKY72-1	evm.TU.supercontig_14.32	1809	602	4	supercontig_14: 357594 - 359995	6.6e-186
CpWRKY72-2	evm.TU.supercontig_17.220	1812	603	5	supercontig_17: 2581193 - 2584970	2.0e-201
CpWRKY74	evm.TU.supercontig_1244.3	1074	357	2	supercontig_1244: 9823 - 12104	5.7e-173
CpWRKY75-1	evm.TU.supercontig_43.86	552	183	1	supercontig_43: 1118553 - 1120033	7.5e-60
CpWRKY75-2	evm.TU.supercontig_807.4	561	186	1	supercontig_807: 19181 - 20131	1.1e-54
Chlamydomonas reinhardtii						
CreinWRKY26	Cre04.g228400	1920	639	10	chromosome_4: 3560165 - 3564466	0.0e+00
Citrus clementina						
CcWRKY1	Ciclev10015049m.g	1467	488	4	scaffold_2: 23826323 - 23829613	0.0e+00
CcWRKY4	Ciclev10031225m.g	1578	525	3	scaffold_4: 24231402 - 24235138	0.0e+00
CcWRKY5	Ciclev10023956m.g	726	241	3	scaffold_3: 50663970 - 50666133	3.6e-164
CcWRKY6	Ciclev10014642m.g	1824	607	5	scaffold_2: 35513796 - 35516719	0.0e+00
CcWRKY7-1	Ciclev10001636m.g	1074	357	2	scaffold_5: 39928705 - 39930585	2.7e-238
CcWRKY7-2	Ciclev10025925m.g	1092	363	2	scaffold_7: 3541924 - 3543863	4.6e-240
CcWRKY9	Ciclev10030455m.g	1185	394	4	scaffold_659: 2206 - 4049	2.3e-260

CcWRKY11	Ciclev10008836m.g	1029	342	2	scaffold_1: 526813 - 529150	2.2e-227
CcWRKY14	Ciclev10014984m.g	1506	501	2	scaffold_2: 30971742 - 30974734	0.0e+00
CcWRKY16	Ciclev10021624m.g	825	274	2	scaffold_3: 46498627 - 46500232	1.1e-168
CcWRKY18	Ciclev10026105m.g	957	318	4	scaffold_7: 14560604 - 14562711	7.2e-209
CcWRKY20	Ciclev10007882m.g	1692	563	5	scaffold_1: 23073033 - 23077659	0.0e+00
CcWRKY21	Ciclev10020867m.g	1059	352	2	scaffold_3: 43758957 - 43761623	8.1e-235
CcWRKY22	Ciclev10020943m.g	1041	346	2	scaffold_3: 48640071 - 48641944	1.7e-226
CcWRKY23	Ciclev10021174m.g	969	322	2	scaffold_3: 46685981 - 46687931	6.1e-216
CcWRKY26	Ciclev10000654m.g	1785	594	4	scaffold_5: 12677414 - 12680761	0.0e+00
CcWRKY27	Ciclev10005095m.g	1212	403	2	scaffold_9: 29596219 - 29597957	7.1e-272
CcWRKY29	Ciclev10024061m.g	948	315	2	scaffold_3: 1452876 - 1454058	1.3e-214
CcWRKY30	Ciclev10020744m.g	1095	364	2	scaffold_3: 48321396 - 48324697	5.4e-248
CcWRKY31-1	Ciclev10007917m.g	1644	547	5	scaffold_1: 22651299 - 22653980	0.0e+00
CcWRKY31-2	Ciclev10014665m.g	1800	599	4	scaffold_2: 22459166 - 22461667	0.0e+00
CcWRKY32	Ciclev10008135m.g	1458	485	4	scaffold_1: 28433972 - 28437921	0.0e+00
CcWRKY33	Ciclev10011386m.g	1701	566	4	scaffold_6: 17113588 - 17116563	0.0e+00
CcWRKY34	Ciclev10007904m.g	1662	553	4	scaffold_1: 24328901 - 24333161	0.0e+00
CcWRKY36	Ciclev10031162m.g	1632	543	3	scaffold_4: 23602551 - 23606799	0.0e+00
CcWRKY39	Ciclev10028715m.g	1080	359	2	scaffold_8: 17468874 - 17471267	3.1e-242
CcWRKY40-1	Ciclev10009250m.g	777	258	3	scaffold_1: 1041086 - 1043376	5.4e-174
CcWRKY40-2	Ciclev10008930m.g	966	321	4	scaffold_1: 1051155 - 1052947	1.1e-212
CcWRKY41	Ciclev10005165m.g	1146	381	2	scaffold_9: 30033675 - 30035553	1.9e-259
CcWRKY43	Ciclev10024257m.g	567	188	1	scaffold_3: 48618880 - 48620746	7.0e-127
CcWRKY44	Ciclev10011660m.g	1407	468	5	scaffold_6: 11954097 - 11958888	0.0e+00
CcWRKY45	Ciclev10032943m.g	489	162	1	scaffold_4: 12596113 - 12597155	6.7e-106
CcWRKY47-2	Ciclev10006728m.g	417	138	3	scaffold_9: 81344 - 82445	5.6e-91
CcWRKY47-1	Ciclev10019820m.g	1506	501	5	scaffold_3: 47121093 - 47124518	0.0e+00
CcWRKY48	Ciclev10005203m.g	1119	372	2	scaffold_9: 700795 - 702522	2.6e-250
CcWRKY49	Ciclev10013616m.g	912	303	2	scaffold_6: 14334820 - 14336151	4.1e-202
CcWRKY50	Ciclev10026733m.g	417	138	2	scaffold_7: 18561339 - 18565286	5.9e-90
CcWRKY51	Ciclev10009761m.g	480	159	2	scaffold_1: 25604509 - 25605857	4.0e-106
CcWRKY53	Ciclev10021038m.g	1011	336	2	scaffold_3: 1054257 - 1056078	4.9e-227
CcWRKY54	Ciclev10012055m.g	1071	356	2	scaffold_6: 17675267 - 17676898	1.7e-238

CcWRKY55	Ciclev10033384m.g	1071	356	2	scaffold_4: 2328696 - 2330790	4.0e-241
CcWRKY57-1	Ciclev10032285m.g	876	291	3	scaffold_4: 24341642 - 24346954	4.1e-198
CcWRKY57-2	Ciclev10017173m.g	384	127	2	scaffold_2: 220534 - 222401	2.6e-84
CcWRKY58	Ciclev10013781m.g	2751	916	10	scaffold_6: 25306829 - 25315243	0.0e+00
CcWRKY65	Ciclev10016229m.g	825	274	2	scaffold_2: 34412152 - 34415059	8.4e-187
CcWRKY70	Ciclev10032192m.g	930	309	2	scaffold_4: 2333547 - 2335111	3.9e-208
CcWRKY71-1	Ciclev10032012m.g	1029	342	2	scaffold_4: 16046322 - 16048971	8.3e-232
CcWRKY71-2	Ciclev10018230m.g	951	316	3	scaffold_2: 34859930 - 34862118	1.0e-213
CcWRKY72-1	Ciclev10011501m.g	1551	516	4	scaffold_6: 24662454 - 24665352	0.0e+00
CcWRKY72-2	Ciclev10019383m.g	1809	602	4	scaffold_3: 2226805 - 2230144	0.0e+00
CcWRKY75-1	Ciclev10017531m.g	552	183	1	scaffold_2: 13341557 - 13343067	6.1e-124
CcWRKY75-2	Ciclev10032816m.g	585	194	1	scaffold_4: 20998708 - 21001535	9.7e-129
Citrus sinensis						
CsWRKY1	orange1.1g011340m.g	1467	488	4	scaffold00077: 307046 - 310334	0.0e+00
CsWRKY2	orange1.1g004963m.g	2166	721	5	scaffold00013: 1838851 - 1843181	0.0e+00
CsWRKY4-1	orange1.1g009051m.g	1638	545	4	scaffold00003: 3348929 - 3352620	0.0e+00
CsWRKY4-2	orange1.1g010802m.g	1506	501	3	scaffold00015: 1591146 - 1594814	0.0e+00
CsWRKY5	orange1.1g026216m.g	726	241	3	scaffold00001: 354623 - 357157	3.6e-164
CsWRKY6	orange1.1g007099m.g	1857	618	6	scaffold00007: 1958471 - 1961762	0.0e+00
CsWRKY7-1	orange1.1g018215m.g	1080	359	2	scaffold00002: 1863592 - 1865359	2.0e-239
CsWRKY7-2	orange1.1g017930m.g	1092	363	2	scaffold00006: 1346375 - 1348315	4.6e-240
CsWRKY9	orange1.1g036819m.g	807	269	4	scaffold00568: 96439 - 97905	3.0e-173
CsWRKY10-1	orange1.1g040283m.g	402	134	0	scaffold00162: 293448 - 293849	9.2e-88
CsWRKY10-2	orange1.1g032690m.g	408	135	2	scaffold00123: 233020 - 234931	3.3e-90
CsWRKY13	orange1.1g026950m.g	693	230	2	scaffold01166: 10456 - 13713	4.6e-155
CsWRKY14	orange1.1g012605m.g	1383	460	2	scaffold00008: 2345601 - 2348134	3.3e-307
CsWRKY17	orange1.1g019404m.g	1026	341	2	scaffold00025: 483188 - 485204	6.7e-227
CsWRKY19	orange1.1g014629m.g	1266	421	3	scaffold00172: 174342 - 178393	3.0e-286
CsWRKY20	orange1.1g008458m.g	1695	564	5	scaffold00049: 532438 - 537337	0.0e+00
CsWRKY21	orange1.1g018659m.g	1059	352	3	scaffold00022: 289543 - 292094	3.9e-235
CsWRKY22	orange1.1g019126m.g	1041	346	2	scaffold00001: 2333743 - 2335147	3.1e-226
CsWRKY23	orange1.1g020713m.g	969	322	2	scaffold00001: 4236944 - 4238415	1.2e-215
CsWRKY27	orange1.1g015616m.g	1212	403	2	scaffold00097: 230834 - 232265	9.1e-272

CsWRKY29	orange1.1g021896m.g	921	306	2	scaffold00009: 1025605 - 1026760	3.9e-207
CsWRKY31	orange1.1g007546m.g	1800	599	4	scaffold00121: 389458 - 391889	0.0e+00
CsWRKY32	orange1.1g036653m.g	1464	487	4	scaffold00068: 482854 - 486663	8.9e-313
CsWRKY33-1	orange1.1g013222m.g	1344	447	4	scaffold00042: 689265 - 692549	7.0e-303
CsWRKY33-2	orange1.1g011483m.g	1455	484	3	scaffold00626: 42818 - 45853	0.0e+00
CsWRKY35	orange1.1g023982m.g	825	274	2	scaffold00001: 4402815 - 4404405	4.7e-184
CsWRKY40-1	orange1.1g020831m.g	966	321	4	scaffold00025: 1006589 - 1008372	1.1e-212
CsWRKY40-2	orange1.1g025097m.g	777	258	3	scaffold00025: 996904 - 999151	1.1e-173
CsWRKY41-1	orange1.1g019737m.g	1011	336	2	scaffold00009: 1409048 - 1410893	2.7e-227
CsWRKY41-2	orange1.1g045032m.g	1065	354	2	scaffold00104: 283550 - 285259	1.1e-237
CsWRKY42	orange1.1g008964m.g	1644	547	5	scaffold00049: 126215 - 128926	0.0e+00
CsWRKY43	orange1.1g046286m.g	567	188	1	scaffold00001: 2355401 - 2357338	1.6e-127
CsWRKY45	orange1.1g031298m.g	489	162	1	scaffold00035: 211943 - 212982	7.3e-106
CsWRKY46	orange1.1g017895m.g	1095	364	2	scaffold00001: 2647888 - 2651028	2.1e-248
CsWRKY47	orange1.1g040711m.g	1506	501	5	scaffold00001: 3810277 - 3813167	0.0e+00
CsWRKY48	orange1.1g017479m.g	1116	371	2	scaffold00016: 1122184 - 1123697	1.2e-249
CsWRKY49-1	orange1.1g038951m.g	912	303	2	scaffold00189: 257377 - 258688	9.9e-203
CsWRKY49-2	orange1.1g031482m.g	480	159	2	scaffold00013: 646034 - 647324	4.0e-106
CsWRKY51	orange1.1g029257m.g	591	196	3	scaffold00362: 80427 - 84896	2.6e-80
CsWRKY54	orange1.1g021598m.g	933	310	2	scaffold00024: 87173 - 88697	8.6e-209
CsWRKY55-1	orange1.1g018407m.g	1071	356	2	scaffold00024: 82311 - 84401	6.9e-241
CsWRKY55-2	orange1.1g041468m.g	375	124	2	scaffold00037: 611554 - 612440	5.7e-80
CsWRKY57	orange1.1g045509m.g	876	291	2	scaffold00003: 3456550 - 3459372	3.7e-198
CsWRKY65	orange1.1g022353m.g	897	298	3	scaffold00007: 901284 - 905441	6.9e-205
CsWRKY71-1	orange1.1g021142m.g	954	317	2	scaffold00007: 1347875 - 1349215	9.4e-214
CsWRKY71-2	orange1.1g019375m.g	1029	342	2	scaffold00057: 464133 - 466331	7.6e-232
CsWRKY72-1	orange1.1g010903m.g	1497	498	2	scaffold00003: 2727603 - 2730035	0.0e+00
CsWRKY72-2	orange1.1g009794m.g	1578	525	2	scaffold00009: 279247 - 281128	0.0e+00
CsWRKY72-3	orange1.1g045987m.g	1533	510	3	scaffold00015: 942103 - 943915	0.0e+00
CsWRKY70	orange1.1g020291m.g	987	328	2	scaffold00036: 115494 - 117084	1.6e-218
CsWRKY74	orange1.1g018255m.g	1080	359	2	scaffold00124: 381097 - 383496	5.6e-242
CsWRKY75-2	orange1.1g043122m.g	321	106	1	scaffold00003: 164635 - 166589	6.2e-69
CsWRKY75-1	orange1.1g030050m.g	552	183	2	scaffold00038: 626400 - 630108	5.8e-124

Coccomyxa subellipsoidea						
CsubWRKY26	gw1.1.725.1	513	171	4	scaffold_1: 2028937 - 2030784	2.0e-113
Cucumis sativus						
CsaWRKY1	Cucsa.100440	1302	433	6	scaffold00926: 914397 - 918705	3.5e-286
CsaWRKY2-1	Cucsa.041700	2259	752	6	scaffold00540: 270292 - 275266	0.0e+00
CsaWRKY2-2	Cucsa.356720	1584	527	4	scaffold03577: 1574124 - 1581251	0.0e+00
CsaWRKY3-1	Cucsa.158040	1521	506	3	scaffold01142: 195925 - 200710	0.0e+00
CsaWRKY3-2	Cucsa.284730	1479	492	3	scaffold02653: 1369003 - 1372205	0.0e+00
CsaWRKY9	Cucsa.049620	1245	414	4	scaffold00550: 97472 - 100733	1.8e-280
CsaWRKY11-1	Cucsa.282040	849	282	2	scaffold02641: 173928 - 175382	4.9e-191
CsaWRKY11-2	Cucsa.311060	1053	350	2	scaffold02995: 721845 - 723766	4.4e-231
CsaWRKY13-1	Cucsa.101460	735	244	2	scaffold00927: 712502 - 715002	1.6e-167
CsaWRKY13-2	Cucsa.250310	741	246	3	scaffold02219: 466299 - 468905	1.4e-165
CsaWRKY13	Cucsa.260060	660	219	3	scaffold02229: 6346998 - 6349594	2.8e-152
CsaWRKY14	Cucsa.048520	1548	515	2	scaffold00542: 3200793 - 3203774	0.0e+00
CsaWRKY15-1	Cucsa.352410	1179	392	2	scaffold03533: 261807 - 263639	2.6e-249
CsaWRKY15-2	Cucsa.073190	885	294	2	scaffold00765: 793624 - 795654	2.6e-188
CsaWRKY15-3	Cucsa.102600	885	294	2	scaffold00927: 1610330 - 1611544	1.5e-192
CsaWRKY20	Cucsa.042320	1602	533	5	scaffold00540: 759912 - 770172	0.0e+00
CsaWRKY21-1	Cucsa.388650	1059	352	2	scaffold03967: 1065684 - 1067688	2.6e-239
CsaWRKY21-2	Cucsa.168370	555	184	2	scaffold01160: 128002 - 129600	2.5e-182
CsaWRKY22-1	Cucsa.258700	1011	336	2	scaffold02229: 5548838 - 5550224	7.6e-227
CsaWRKY22-2	Cucsa.106960	810	269	1	scaffold00930: 410428 - 412046	3.1e-121
CsaWRKY23-1	Cucsa.204920	793	263	4	scaffold01416: 77428 - 81403	2.8e-137
CsaWRKY23-2	Cucsa.300610	999	332	3	scaffold02931: 163188 - 166573	2.2e-178
CsaWRKY24-1	Cucsa.106970	603	200	1	scaffold00930: 415357 - 417323	7.2e-135
CsaWRKY24-2	Cucsa.258730	540	179	1	scaffold02229: 5556162 - 5556853	4.5e-120
CsaWRKY26	Cucsa.111910	1731	576	4	scaffold00953: 587817 - 590545	0.0e+00
CsaWRKY27	Cucsa.201710	978	326	2	scaffold01376: 1242 - 2616	4.0e-180
CsaWRKY31	Cucsa.045040	1872	623	5	scaffold00542: 1143492 - 1146237	4.4e-261
CsaWRKY32	Cucsa.093390	1524	507	4	scaffold00915: 21612 - 25288	0.0e+00
CsaWRKY33-1	Cucsa.000200	852	283	2	scaffold00009: 1869 - 4028	2.4e-190
CsaWRKY33-2	Cucsa.176780	1434	477	4	scaffold01225: 872033 - 875284	0.0e+00

CsaWRKY35-1	Cucsa.096680	831	276	2	scaffold00919: 2211901 - 2213366	6.1e-172
CsaWRKY35-2	Cucsa.167500	1141	381	2	scaffold01154: 862999 - 866145	2.1e-260
CsaWRKY40-1	Cucsa.177230	948	315	4	scaffold01225: 1247154 - 1248879	1.6e-222
CsaWRKY40-2	Cucsa.162810	939	312	3	scaffold01144: 2340752 - 2342668	1.9e-207
CsaWRKY40-3	Cucsa.271570	978	325	4	scaffold02500: 391891 - 395153	5.7e-215
CsaWRKY41-1	Cucsa.012940	1049	348	3	scaffold00164: 728174 - 729549	1.6e-222
CsaWRKY41-2	Cucsa.107310	1068	355	2	scaffold00930: 705007 - 707249	9.7e-246
CsaWRKY41-3	Cucsa.259110	1014	337	2	scaffold02229: 5753147 - 5755379	2.7e-228
CsaWRKY42	Cucsa.148640	1314	437	4	scaffold01107: 707841 - 710754	5.3e-289
CsaWRKY44	Cucsa.307270	1029	342	3	scaffold02977: 498026 - 500262	1.3e-226
CsaWRKY45	Cucsa.087850	399	132	1	scaffold00888: 347131 - 348272	3.9e-84
CsaWRKY48-1	Cucsa.247040	981	326	1	scaffold02188: 75312 - 77735	3.8e-221
CsaWRKY48-2	Cucsa.252440	942	313	2	scaffold02229: 1365532 - 1366989	3.9e-208
CsaWRKY49	Cucsa.066300	943	313	2	scaffold00696: 829823 - 831928	4.9e-191
CsaWRKY50-1	Cucsa.056150	492	163	1	scaffold00602: 334670 - 336496	9.1e-15
CsaWRKY50-2	Cucsa.101530	525	174	2	scaffold00927: 797073 - 798838	2.8e-112
CsaWRKY51	Cucsa.250350	618	205	2	scaffold02219: 507446 - 511565	2.0e-128
CsaWRKY53	Cucsa.078810	891	296	2	scaffold00793: 1259288 - 1260978	4.1e-203
CsaWRKY55	Cucsa.143840	954	317	2	scaffold01080: 213348 - 219326	5.4e-90
CsaWRKY57-1	Cucsa.327760	921	306	2	scaffold03159: 104776 - 108670	2.8e-110
CsaWRKY57-2	Cucsa.118200	444	148	2	scaffold00998: 29643 - 31603	3.8e-87
CsaWRKY60	Cucsa.162820	516	172	2	scaffold01144: 2350607 - 2351535	3.0e-111
CsaWRKY65	Cucsa.212870	858	285	2	scaffold01543: 671733 - 673416	2.5e-193
CsaWRKY69	Cucsa.195730	1218	405	1	scaffold01352: 73603 - 76079	1.3e-170
CsaWRKY70-1	Cucsa.177060	843	280	1	scaffold01225: 1045156 - 1046394	6.0e-193
CsaWRKY70-2	Cucsa.143830	849	282	2	scaffold01080: 207400 - 211268	3.4e-187
CsaWRKY71-1	Cucsa.047510	786	261	2	scaffold00542: 2512823 - 2514698	4.1e-173
CsaWRKY71-2	Cucsa.239590	862	288	2	scaffold02046: 380323 - 382497	6.3e-190
CsaWRKY72-1	Cucsa.197550	1773	590	4	scaffold01357: 915560 - 919333	0.0e+00
CsaWRKY72-2	Cucsa.097890	1590	529	3	scaffold00923: 86796 - 89984	0.0e+00
CsaWRKY75-1	Cucsa.054040	513	170	2	scaffold00582: 298992 - 302104	9.1e-115
CsaWRKY75-2	Cucsa.095090	648	215	1	scaffold00919: 1054988 - 1056251	2.0e-142
Dictyostelium purpureum						

DpWRKY3-1	10723	525	174	2	scaffold_39:96720-97331	9.0e-80
DpWRKY3-2	53418	1197	398	2	scaffold_39:96386-97709	1.8e-120
DpWRKY3-3	45948	819	272	2	scaffold_39:96386-97331	3.8e-121
DpWRKY3-4	53422	1284	427	1	scaffold_39:96386-97709	2.7e-131
DpWRKY3-5	76129	2619	872	1	scaffold_39:96426-99044	2.8e-130
DpWRKY3-6	96960	2659	852	1	scaffold_39:96386-99044	2.8e-130
DpWRKY3-7	149043	2619	872	1	scaffold_39:96426-99044	2.8e-130
DpWRKY3-8	28943	555	184	1	scaffold_39:97155-97709	5.7e-41
DpWRKY4	28901	546	181	1	scaffold_39:97164-97709	2.5e-39
Eucalyptus grandis						
EgWRKY1	Eucgr.A01053	1353	450	3	scaffold_1: 16314489 - 16316763	6.9e-298
EgWRKY2-1	Eucgr.B01415	1800	600	3	scaffold_2: 23333012 - 23335510	0.0e+00
EgWRKY2-2	Eucgr.F01981.1	2256	751	5	scaffold_6: 26563379 - 26567799	0.0e+00
EgWRKY3-1	Eucgr.G02469	1569	522	4	scaffold_7: 43236902 - 43240562	0.0e+00
EgWRKY3-2	Eucgr.B03189	1590	529	3	scaffold_2: 56937297 - 56942606	0.0e+00
EgWRKY4	Eucgr.I01998	1698	565	4	scaffold_9: 29693123 – 29697273	0.0e+00
EgWRKY5	Eucgr.D01219	669	222	3	scaffold_4: 22004480 - 22009874	5.0e-152
EgWRKY6-1	Eucgr.E04011	1818	605	5	scaffold_5: 69015136 - 69017796	0.0e+00
EgWRKY6-2	Eucgr.F00740	1524	507	4	scaffold_6: 9696493 - 9701259	0.0e+00
EgWRKY6-3	Eucgr.K00786	1884	627	4	scaffold_11: 9055812 - 9058766	0.0e+00
EgWRKY7-1	Eucgr.F00187.1	1314	437	3	scaffold_6: 2897331 - 2900217	3.5e-292
EgWRKY7-2	Eucgr.I00882	1041	346	2	scaffold_9: 18107095 - 18109750	6.8e-229
EgWRKY9	Eucgr.G00619	1326	441	4	scaffold_7: 10713778 - 10716223	3.9e-298
EgWRKY11-1	Eucgr.C04011	1032	343	2	scaffold_3: 74743583 - 74745824	1.4e-225
EgWRKY11-2	Eucgr.C00077	864	287	1	scaffold_3: 3374239 - 3375650	1.2e-190
EgWRKY12	Eucgr.J03117	948	315	4	scaffold_10: 38255672 - 38258552	3.8e-205
EgWRKY13	Eucgr.I00305	822	273	2	scaffold_9: 5900669 - 5904355	3.3e-183
EgWRKY18-1	Eucgr.C02660	867	288	4	scaffold_3: 50777598 - 50779966	5.4e-192
EgWRKY18-2	Eucgr.C01943	699	232	2	scaffold_3: 33718962 - 33719970	3.5e-155
EgWRKY18-3	Eucgr.D01605	489	162	1	scaffold_4: 29746310 - 29747218	6.8e-103
EgWRKY18-4	Eucgr.F04317	801	266	3	scaffold_6: 51841944 - 51843982	5.9e-178
EgWRKY18-5	Eucgr.C02659	801	266	3	scaffold_3: 50768111 - 50770634	1.7e-178
EgWRKY20-1	Eucgr.F03522	1803	600	5	scaffold_6: 43873432 - 43878504	0.0e+00

EgWRKY20-2	Eucgr.F00621	1791	596	6	scaffold_6: 8242460 - 8248572	0.0e+00
EgWRKY21	Eucgr.B01503	1032	343	2	scaffold_2: 24748680 - 24751505	2.1e-229
EgWRKY22-1	Eucgr.E00098	1077	358	2	scaffold_5: 834768 - 836429	5.0e-239
EgWRKY22-2	Eucgr.D02324	1191	396	3	scaffold_4: 37139105 - 37140698	2.1e-255
EgWRKY23-1	Eucgr.H00996.1	975	324	2	scaffold_8: 12224321 - 12226370	1.5e-215
EgWRKY23-2	Eucgr.H04061	1107	368	3	scaffold_8: 58494151 - 58496780	1.1e-242
EgWRKY26	Eucgr.B04010	1659	552	4	scaffold_2: 63952095 - 63954766	0.0e+00
EgWRKY27-1	Eucgr.A02718	1197	398	2	scaffold_1: 37801890 - 37803291	8.8e-265
EgWRKY27-2	Eucgr.D01811	855	284	2	scaffold_4: 32076541 - 32078152	1.1e-191
EgWRKY28-1	Eucgr.A01990	726	241	2	scaffold_1: 30609302 - 30611790	3.0e-160
EgWRKY28-2	Eucgr.K01219	1008	335	2	scaffold_11: 15395781 - 15397506	9.6e-228
EgWRKY28-3	Eucgr.D00336	897	298	2	scaffold_4: 5580615 - 5582270	6.9e-202
EgWRKY32-1	Eucgr.C00348	1461	486	4	scaffold_3: 6785474 - 6789716	0.0e+00
EgWRKY32-2	Eucgr.C02545	1452	483	4	scaffold_3: 48605472 - 48611721	0.0e+00
EgWRKY32-3	Eucgr.C02487	381	126	1	scaffold_3: 47521462 - 47522311	3.0e-76
EgWRKY32-4	Eucgr.C02481	1641	546	5	scaffold_3: 47447776 - 47454168	2.5e-300
EgWRKY33	Eucgr.K02940	1683	560	4	scaffold_11: 37455769 - 37458568	0.0e+00
EgWRKY35-1	Eucgr.K01971	1353	450	2	scaffold_11: 25335850 - 25338428	4.1e-309
EgWRKY35-2	Eucgr.H01007	870	289	1	scaffold_8: 12328171 - 12329731	4.0e-194
EgWRKY36	Eucgr.G02535	1656	551	2	scaffold_7: 43853092 - 43855278	0.0e+00
EgWRKY40	Eucgr.F03955	1026	341	4	scaffold_6: 47868684 - 47871318	4.4e-222
EgWRKY41-1	Eucgr.G00258	774	257	3	scaffold_7: 4110565 - 4112568	2.2e-173
EgWRKY41-2	Eucgr.K00331	1050	349	4	scaffold_11: 3886353 - 3888437	5.0e-237
EgWRKY41-3	Eucgr.D02359	1014	337	2	scaffold_4: 37468304 - 37470736	5.2e-231
EgWRKY42	Eucgr.H04135	786	261	2	scaffold_8: 59253266 - 59255494	2.6e-171
EgWRKY44-1	Eucgr.K03196	1389	462	6	scaffold_11: 40611942 - 40617240	5.6e-306
EgWRKY44-2	Eucgr.J02655	993	330	5	scaffold_10: 32642356 - 32646102	3.4e-220
EgWRKY44-3	Eucgr.A01195	1404	467	6	scaffold_1: 18649484 - 18654669	4.7e-313
EgWRKY45	Eucgr.H03349	561	186	1	scaffold_8: 49110786 - 49113127	7.3e-125
EgWRKY47	Eucgr.A01001	1608	535	5	scaffold_1: 15633648 - 15637795	0.0e+00
EgWRKY49-1	Eucgr.K03115	918	305	2	scaffold_11: 39724385 - 39725703	3.7e-208
EgWRKY49-2	Eucgr.J01756	339	112	3	scaffold_10: 22891086 - 22892865	2.4e-71
EgWRKY50	Eucgr.C00675	480	159	2	scaffold_3: 11929214 - 11930562	1.3e-106

EgWRKY51-1	Eucgr.I00316	573	190	2	scaffold_9: 6044956 - 6048416	9.0e-128
EgWRKY51-2	Eucgr.I00317	576	191	2	scaffold_9: 6069028 - 6073301	1.7e-127
EgWRKY53-1	Eucgr.E00850	1083	360	2	scaffold_5: 8949421 - 8951955	9.3e-247
EgWRKY53-2	Eucgr.G00268	870	289	3	scaffold_7: 4239973 - 4243006	5.6e-201
EgWRKY53-3	Eucgr.J02653	363	121	1	scaffold_10: 32596348 - 32597306	5.2e-82
EgWRKY54	Eucgr.G03145	966	321	2	scaffold_7: 49883728 - 49885656	7.8e-217
EgWRKY55-1	Eucgr.G03144	570	189	2	scaffold_7: 49877930 - 49880843	3.3e-127
EgWRKY55-2	Eucgr.J00107	1092	363	2	scaffold_10: 1375836 - 1379405	1.4e-245
EgWRKY56-1	Eucgr.E00096	636	211	1	scaffold_5: 827344 - 830213	1.9e-140
EgWRKY56-2	Eucgr.D01809	624	207	1	scaffold_4: 32064347 - 32065452	7.1e-143
EgWRKY56-3	Eucgr.D02326	372	123	1	scaffold_4: 37146617 - 37147614	8.1e-77
EgWRKY57-1	Eucgr.G02459	936	311	2	scaffold_7: 43141732 - 43147322	5.3e-210
EgWRKY57-2	Eucgr.B03165	984	327	2	scaffold_2: 56770199 - 56773947	2.3e-221
EgWRKY65	Eucgr.E02783	1113	370	3	scaffold_5: 45370118 - 45372351	1.5e-251
EgWRKY69	Eucgr.K01151	834	277	2	scaffold_11: 14565246 - 14567796	8.0e-184
EgWRKY70-1	Eucgr.J00106	960	319	2	scaffold_10: 1369903 - 1371673	2.9e-215
EgWRKY70-2	Eucgr.K01289	753	251	2	scaffold_11: 16072134 - 16073251	1.1e-165
EgWRKY72-1	Eucgr.K02453	1641	546	4	scaffold_11: 31858355 - 31862519	0.0e+00
EgWRKY72-2	Eucgr.B00437	1641	546	2	scaffold_2: 5560588 - 5562746	0.0e+00
EgWRKY72-3	Eucgr.I01927	1194	397	3	scaffold_9: 29205003 - 29207324	2.4e-268
EgWRKY75-1	Eucgr.I01633	543	180	2	scaffold_9: 26294756 - 26296529	1.1e-119
EgWRKY75-2	Eucgr.B03520	633	210	1	scaffold_2: 59850633 - 59853374	3.6e-141
Fragaria vesca						
FvWRKY1	gene21591-v1.0-hybrid	1518	505	3	LG3: 3591920 - 3593898	1.0e-194
FvWRKY2	gene01197-v1.0-hybrid	2205	734	4	LG3: 28842077 - 28845292	0.0e+00
FvWRKY3	gene04391-v1.0-hybrid	1560	519	3	LG6: 33441504 - 33444478	4.0e-25
FvWRKY5	gene04864-v1.0-hybrid	918	305	4	LG7: 10593300 - 10597571	7.1e-73
FvWRKY6-1	gene03411-v1.0-hybrid	1644	547	5	LG3: 13659296 - 13661974	4.0e-162
FvWRKY6-2	gene19478-v1.0-hybrid	1941	646	8	LG3: 635329 - 638908	7.9e-222
FvWRKY6-3	gene23963-v1.0-hybrid	2124	707	8	LG6: 34573480 - 34579047	1.2e-272
FvWRKY7	gene09147-v1.0-hybrid	1005	334	2	LG2: 21403605 - 21404884	1.1e-122
FvWRKY9	gene11774-v1.0-hybrid	1698	565	4	LG5: 21244362 - 21246895	2.6e-171
FvWRKY11-1	gene08720-v1.0-hybrid	1029	342	2	LG2: 19889909 - 19891443	2.9e-153

FvWRKY11-2	gene12308-v1.0-hybrid	906	301	2	LG5: 26018822 - 26019967	2.5e-135
FvWRKY13	gene21124-v1.0-hybrid	717	238	2	LG1: 16977774 - 16980821	3.6e-101
FvWRKY15	gene23862-v1.0-hybrid	831	276	4	LG6: 35103225 - 35110058	2.0e-54
FvWRKY16	gene03900-v1.0-hybrid	4032	1343	10	LG7: 9804380 - 9813690	2.1e-244
FvWRKY19	gene19013-v1.0-hybrid	534	177	0	LG7: 2541061 - 2541594	1.3e-39
FvWRKY20	gene03549-v1.0-hybrid	1557	518	5	LG3: 14487626 - 14495950	2.1e-305
FvWRKY22	gene19019-v1.0-hybrid	1038	345	2	LG7: 2562323 - 2563625	2.5e-120
FvWRKY23	gene20560-v1.0-hybrid	954	317	2	LG1: 20082299 - 20083620	2.4e-98
FvWRKY26	gene28174-v1.0-hybrid	1863	620	4	LG3: 20524762 - 20527524	1.4e-277
FvWRKY27	gene13173-v1.0-hybrid	1293	430	2	LG7: 21242062 - 21243525	1.2e-95
FvWRKY28	gene24904-v1.0-hybrid	1068	355	2	LG3: 4220487 - 4222135	3.5e-113
FvWRKY29	gene31843-v1.0-hybrid	2283	760	9	LG5: 2002496 - 2009299	1.6e-282
FvWRKY32	gene10079-v1.0-hybrid	1401	466	4	LG1: 281288 - 284149	1.1e-204
FvWRKY33	gene13803-v1.0-hybrid	1686	561	5	LG6: 7284891 - 7287320	7.4e-221
FvWRKY34	gene16807-v1.0-hybrid	2286	761	5	LG6: 1447575 - 1451329	0.0e+00
FvWRKY35-1	gene02269-v1.0-hybrid	942	313	3	LG5: 26381817 - 26383230	4.1e-97
FvWRKY35-2	gene20152-v1.0-hybrid	1479	492	2	LG3: 8405751 - 8408398	2.4e-174
FvWRKY40-1	gene15841-v1.0-hybrid	1509	502	7	LG6: 22439617 - 22446682	1.3e-168
FvWRKY40-2	gene15014-v1.0-hybrid	1149	382	4	LG2: 21673839 - 21675524	1.6e-89
FvWRKY40-3	gene15013-v1.0-hybrid	996	331	2	LG2: 21669642 - 21670851	4.7e-71
FvWRKY41	gene13108-v1.0-hybrid	1119	372	2	LG7: 20935978 - 20937321	8.5e-112
FvWRKY44	gene18152-v1.0-hybrid	1578	525	5	LG6: 4327714 - 4330593	2.3e-258
FvWRKY46	gene19219-v1.0-hybrid	1062	353	2	LG7: 2055090 - 2057069	2.2e-127
FvWRKY47	gene24479-v1.0-hybrid	1494	497	6	LG1: 12364984 - 12367865	4.7e-90
FvWRKY49	gene18438-v1.0-hybrid	891	296	2	LG6: 5465141 - 5466865	7.2e-135
FvWRKY50	gene15421-v1.0-hybrid	504	167	3	LG6: 13781559 - 13782341	1.1e-44
FvWRKY51	gene22024-v1.0-hybrid	573	190	2	LG1: 15966976 - 15969351	1.8e-62
FvWRKY53	gene32076-v1.0-hybrid	1068	355	2	LG5: 2661783 - 2663245	1.0e-149
FvWRKY52	gene16678-v1.0-hybrid	3582	1193	9	LG6: 802048 - 808355	1.2e-227
FvWRKY55	gene21364-v1.0-hybrid	1038	345	2	LG7: 18245294 - 18247359	7.5e-113
FvWRKY59	gene01596-v1.0-hybrid	1812	603	8	LG3: 30785013 - 30789621	5.4e-302
FvWRKY61	gene01256-v1.0-hybrid	1476	491	3	LG6: 1608399 - 1610329	1.8e-159
FvWRKY65	gene29704-v1.0-hybrid	834	277	2	LG3: 5236525 - 5237616	1.3e-99

FvWRKY68	gene05066-v1.0-hybrid	1455	484	3	LG4: 22160877 - 22162817	1.6e-128
FvWRKY69	gene15798-v1.0-hybrid	1860	619	10	LG6: 21741467 - 21754337	1.2e-84
FvWRKY70-1	gene21365-v1.0-hybrid	1023	340	2	LG7: 18248035 - 18250011	2.0e-83
FvWRKY70-2	gene13554-v1.0-hybrid	894	297	2	LG6: 7804584 - 7805985	1.9e-51
FvWRKY70-3	gene13549-v1.0-hybrid	696	231	2	LG6: 7783840 - 7785119	2.6e-48
FvWRKY70-4	gene13561-v1.0-hybrid	1854	617	9	LG6: 7828500 - 7832084	2.2e-138
FvWRKY70-5	gene13547-v1.0-hybrid	1035	344	2	LG6: 7777604 - 7779180	2.0e-89
FvWRKY70-6	gene13546-v1.0-hybrid	1344	447	3	LG6: 7769418 - 7771442	9.5e-85
FvWRKY70-7	gene21370-v1.0-hybrid	5949	1982	20	LG7: 18263740 - 18277966	7.2e-179
FvWRKY71	gene28720-v1.0-hybrid	1107	368	2	LG6: 29859819 - 29861803	1.1e-131
FvWRKY72	gene25959-v1.0-hybrid	2019	672	5	LG5: 6871174 - 6874246	1.7e-229
FvWRKY74	gene16584-v1.0-hybrid	960	319	2	LG6: 257908 - 259177	2.6e-103
FvWRKY75	gene01340-v1.0-hybrid	627	208	1	LG6: 37734638 - 37735974	7.7e-56
Glycine max						
GmWRKY1-1	Glyma.14G010100	1527	508	4	Gm14: 528643 - 532717	0.0e+00
GmWRKY1-2	Glyma.02G306300	1524	507	3	Gm02: 51172225 - 51175238	0.0e+00
GmWRKY2-1	Glyma.09G250500	2205	734	4	Gm09: 43917299 - 43921359	0.0e+00
GmWRKY2-2	Glyma.18G242000	2235	744	4	Gm18: 57313933 - 57318035	0.0e+00
GmWRKY3-1	Glyma.08G240800	1572	523	3	Gm08: 20589275 - 20594206	0.0e+00
GmWRKY3-2	Glyma.18G263400	1563	520	3	Gm18: 59146800 - 59153340	0.0e+00
GmWRKY3-3	Glyma.20G028000	1320	439	3	Gm20: 3214242 - 3219009	3.5e-296
GmWRKY3-4	Glyma.07G227200	1602	533	3	Gm07: 40532555 - 40536935	0.0e+00
GmWRKY4-1	Glyma.02G112100	1368	455	3	Gm02: 10771329 - 10776673	3.1e-309
GmWRKY4-2	Glyma.01G053800	1368	455	3	Gm01: 6797683 - 6802949	5.4e-308
GmWRKY4-3	Glyma.10G171100	579	192	2	Gm10: 39913162 - 39914994	8.3e-129
GmWRKY5-1	Glyma.03G109100	717	238	3	Gm03: 33037355 - 33045604	2.0e-161
GmWRKY5-2	Glyma.07G116300	714	237	3	Gm07: 12609537 - 12618154	2.5e-160
GmWRKY6-1	Glyma.08G320200	1461	486	5	Gm08: 43093454 - 43098754	6.5e-316
GmWRKY6-2	Glyma.18G092200	1437	478	6	Gm18: 9200615 - 9205258	1.5e-311
GmWRKY6-3	Glyma.02G293400	1206	401	5	Gm02: 50283764 - 50286048	1.0e-226
GmWRKY6-4	Glyma.09G005700	1626	541	5	Gm09: 442505 - 444996	7.1e-254
GmWRKY6-5	Glyma.15G110300	1800	599	6	Gm15: 8605739 - 8608639	1.7e-236
GmWRKY6-6	Glyma.13G310100	1845	614	4	Gm13: 39385541 - 39388538	0.0e+00

GmWRKY6-7	Glyma.17G042300	1176	391	6	Gm17: 3142418 - 3147382	7.1e-254
GmWRKY6-8	Glyma.13G117600	1152	383	6	Gm13: 21520430 - 21524539	3.6e-250
GmWRKY9	Glyma.05G123000	1086	361	4	Gm05: 31405618 - 31408779	3.1e-245
GmWRKY10	Glyma.10G171200	1011	336	2	Gm10: 39917393 - 39919651	9.3e-230
GmWRKY11-1	Glyma.06G077400	903	300	2	Gm06: 5977269 - 5979353	3.9e-200
GmWRKY11-2	Glyma.04G076200	840	279	2	Gm04: 6305276 - 6307509	0.0e+00
GmWRKY11-3	Glyma.17G057100	963	320	2	Gm17: 4598261 - 4600061	5.3e-210
GmWRKY11-4	Glyma.13G102000	975	324	2	Gm13: 124039 - 126153	2.5e-213
GmWRKY11-5	Glyma.09G061900	891	296	2	Gm09: 5816890 - 5819213	3.5e-196
GmWRKY11-6	Glyma.15G168200	882	293	2	Gm15: 14920582 - 14923248	6.3e-192
GmWRKY11-7	Glyma.14G135400	951	316	2	Gm14: 19599747 - 19602939	7.1e-212
GmWRKY11-8	Glyma.17G197500	951	316	2	Gm17: 31439925 - 31443097	2.2e-211
GmWRKY13-1	Glyma.06G147500	711	236	2	Gm06: 12006001 - 12009597	1.2e-160
GmWRKY13-2	Glyma.04G218400	705	234	2	Gm04: 45815335 - 45818630	9.7e-159
GmWRKY13-3	Glyma.08G143400	708	235	2	Gm08: 11030859 - 11033309	8.1e-159
GmWRKY13-4	Glyma.05G185400	651	216	3	Gm05: 36943087 - 36944876	1.4e-144
GmWRKY14-1	Glyma.15G135600	1572	523	2	Gm15: 10844191 - 10848519	0.0e+00
GmWRKY14-2	Glyma.09G029800	1521	506	3	Gm09: 2438308 - 2442432	0.0e+00
GmWRKY15-1	Glyma.05G096500	1005	334	2	Gm05: 24873227 - 24875484	2.3e-220
GmWRKY15-2	Glyma.17G168900	999	332	2	Gm17: 15865432 - 15867630	2.5e-219
GmWRKY15-3	Glyma.01G189100	966	321	2	Gm01: 51531990 - 51534998	1.5e-210
GmWRKY15-4	Glyma.11G053100	966	321	2	Gm11: 3985041 - 3986941	2.1e-174
GmWRKY18	Glyma.10G113800	363	120	7	Gm10: 15478720 - 15479844	3.7e-79
GmWRKY20-1	Glyma.18G081200	1734	577	5	Gm18: 7809793 - 7816449	0.0e+00
GmWRKY20-2	Glyma.08G325800	1734	577	5	Gm08: 43556471 - 43562640	0.0e+00
GmWRKY20-3	Glyma.06G2242200	531	177	2	Gm06: 39491007 - 39494203	9.5e-124
GmWRKY20-4	Glyma.02G297400	1767	588	5	Gm02: 50580953 - 50586936	0.0e+00
GmWRKY20-5	Glyma.14G016200	1758	585	5	Gm14: 1152651 - 1158381	0.0e+00
GmWRKY21-1	Glyma.03G159700	1026	341	3	Gm03: 39496945 - 39499620	3.9e-231
GmWRKY21-2	Glyma.10G032900	1179	392	3	Gm10: 2862399 - 2866142	6.5e-269
GmWRKY21-3	Glyma.02G141000	942	313	3	Gm02: 14383057 - 14385983	1.6e-238
GmWRKY22-1	Glyma.16G031900	1008	335	2	Gm16: 2995485 - 2997591	6.0e-223
GmWRKY22-2	Glyma.09G254800	1047	348	2	Gm09: 44201235 - 44203532	4.6e-226

GmWRKY22-3	Glyma.18G238200	1056	351	2	Gm18: 56995337 - 56997541	4.1e-192
GmWRKY23-1	Glyma.02G010900	963	320	2	Gm02: 1024239 - 1026604	1.1e-215
GmWRKY23-2	Glyma.10G011300	972	323	3	Gm10: 1069805 - 1073166	0.0e+00
GmWRKY23-3	Glyma.19G217800	873	290	2	Gm19: 46938033 - 46940019	4.1e-192
GmWRKY23-4	Glyma.03G220800	864	287	2	Gm03: 44408648 - 44410652	2.9e-189
GmWRKY23-5	Glyma.07G238000	1176	391	3	Gm07: 41980063 - 41983090	9.1e-261
GmWRKY26-1	Glyma.11G163300	1647	548	4	Gm11: 30492982 - 30497039	0.0e+00
GmWRKY26-2	Glyma.02G232600	1743	580	4	Gm02: 45077200 - 45080698	0.0e+00
GmWRKY26-3	Glyma.14G200200	1728	575	4	Gm14: 47231177 - 47234695	0.0e+00
GmWRKY27-1	Glyma.16G177000	1227	408	2	Gm16: 33345959 - 33347937	1.9e-277
GmWRKY27-2	Glyma.16G176700	795	264	2	Gm16: 33312431 - 33313552	1.9e-176
GmWRKY27-3	Glyma.20G163200	966	321	2	Gm20: 38962125 - 38964176	1.9e-215
GmWRKY27-4	Glyma.10G230200	894	297	2	Gm10: 45452410 - 45454348	2.2e-198
GmWRKY29-1	Glyma.11G021200	645	214	1	Gm11: 1499929 - 1501106	7.9e-148
GmWRKY29-2	Glyma.08G018300	879	292	1	Gm08: 1472660 - 1474670	2.1e-201
GmWRKY30-1	Glyma.06G125600	1095	364	2	Gm06: 10228530 - 10230877	9.3e-248
GmWRKY30-2	Glyma.04G238300	1095	364	2	Gm04: 47554015 - 47556817	1.3e-245
GmWRKY32-1	Glyma.12G152600	1404	467	4	Gm12: 26693600 - 26703518	3.5e-310
GmWRKY32-2	Glyma.17G074000	1518	505	4	Gm17: 6042312 - 6050551	0.0e+00
GmWRKY32-3	Glyma.02G203800	1518	505	4	Gm02: 41991807 - 41999656	0.0e+00
GmWRKY33-1	Glyma.01G128100	1524	507	4	Gm01: 43157132 - 43160950	0.0e+00
GmWRKY33-2	Glyma.09G280200	1632	543	4	Gm09: 46340575 - 46343915	0.0e+00
GmWRKY33-3	Glyma.18G208800	1626	541	4	Gm18: 53613987 - 53617649	0.0e+00
GmWRKY35-1	Glyma.08G078700	1290	429	2	Gm08: 5992220 - 5996320	5.2e-291
GmWRKY35-2	Glyma.05G123600	1293	430	2	Gm05: 31474087 - 31478680	8.0e-291
GmWRKY35-3	Glyma.03G220100	726	253	2	Gm03: 44338246 - 44340268	1.0e-167
GmWRKY35-4	Glyma.19G217000	795	264	2	Gm19: 46863558 - 46865575	2.0e-188
GmWRKY40-1	Glyma.14G102900	837	278	3	Gm14: 10467577 - 10470309	3.0e-184
GmWRKY40-2	Glyma.17G222500	837	278	3	Gm17: 37829298 - 37831665	6.7e-185
GmWRKY40-3	Glyma06g06530	885	294	3	Gm06: 4658653 - 4660672	4.3e-194
GmWRKY40-4	Glyma.04G061400	663	220	3	Gm04: 4944422 - 4946197	2.8e-146
GmWRKY40-5	Glyma.08G218600	942	313	4	Gm08: 17848730 - 17851238	3.0e-184
GmWRKY40-6	Glyma.07G023300	936	311	4	Gm07: 1769990 - 1772543	1.4e-201

GmWRKY40-7	Glyma.13G370100	930	309	4	Gm13: 44119893 - 44122197	1.3e-201
GmWRKY40-8	Glyma.15G003300	993	330	4	Gm15: 289709 - 291897	5.1e-200
GmWRKY40-9	Glyma.14G103100	849	282	3	Gm14: 10543820 - 10545893	9.9e-212
GmWRKY40-10	Glyma.17G222300	939	312	3	Gm17: 37801991 - 37804561	1.3e-186
GmWRKY41-1	Glyma.08G021900	1080	359	2	Gm08:1759380 - 1761625	9.5e-249
GmWRKY41-2	Glyma.01G224800	969	322	2	Gm01: 54433422 - 54435852	9.2e-219
GmWRKY41-3	Glyma.05G215900	1092	363	2	Gm05: 40700822 - 40702765	2.7e-250
GmWRKY41-4	Glyma.19G254800	1089	362	2	Gm19: 49828036 - 49830844	1.1e-246
GmWRKY41-5	Glyma.03G256700	1089	362	2	Gm03: 47154487 - 47158248	1.7e-250
GmWRKY41-6	Glyma.16G026400	1122	373	2	Gm16: 2538810 - 2542188	2.6e-253
GmWRKY41-7	Glyma.07G057400	1110	369	2	Gm07: 5052597 - 5055648	9.6e-243
GmWRKY42	Glyma.15G186300	1356	451	5	Gm15: 19180353 - 19185976	2.1e-303
GmWRKY44-1	Glyma.03G176600	1347	448	6	Gm03: 40963184 - 40972913	4.7e-302
GmWRKY44-2	Glyma.19G177400	1416	471	6	Gm19: 43469225 - 43478174	0.0e+00
GmWRKY47-1	Glyma.02G007500	1455	484	6	Gm02: 777358 - 781690	0.0e+00
GmWRKY47-2	Glyma.03G224700	1626	541	5	Gm03: 44705517 - 44709700	0.0e+00
GmWRKY47-3	Glyma.19G221700	1551	516	5	Gm19: 47231197 - 47236101	0.0e+00
GmWRKY48-1	Glyma.15G139000	1071	356	3	Gm15: 11291206 - 11294060	9.7e-240
GmWRKY48-2	Glyma.09G034300	996	331	2	Gm09: 2824460 - 2827541	8.9e-221
GmWRKY49	Glyma.14G199800	999	332	0	Gm14: 47189224 - 47190551	8.0e-226
GmWRKY50-1	Glyma.04G054200	486	161	2	Gm04: 4349265 - 4350787	7.2e-109
GmWRKY50-2	Glyma.06G054500	528	175	2	Gm06: 4090868 - 4092314	3.0e-119
GmWRKY51-1	Glyma.06G147100	591	196	2	Gm06: 11960799 - 11964431	3.1e-132
GmWRKY51-2	Glyma.04G218700	591	196	2	Gm04: 45843086 - 45845805	2.6e-132
GmWRKY51-3	Glyma.08G142400	555	184	2	Gm08: 10932560 - 10935883	1.8e-122
GmWRKY51-4	Glyma.05G184500	567	188	2	Gm05: 36852753 - 36855968	4.3e-127
GmWRKY54-1	Glyma.14G186000	912	303	2	Gm14: 45729764 - 45734659	1.7e-205
GmWRKY54-2	Glyma.14G186100	723	240	2	Gm14: 45743350 - 45755195	7.1e-159
GmWRKY55-1	Glyma.06G142100	966	331	2	Gm06: 11540905 - 11545294	1.5e-220
GmWRKY55-2	Glyma.04G223200	1014	337	2	Gm04: 46249199 - 46253557	8.7e-225
GmWRKY55-3	Glyma.05G165800	4068	1355	4	Gm05: 35364051 - 35374699	0.0e+00
GmWRKY56-1	Glyma.16G031400	588	195	1	Gm16: 2926618 - 2929426	6.5e-130
GmWRKY56-2	Glyma.09G254400	579	192	1	Gm09: 44180119 - 44182004	1.7e-132

GmWRKY56-3	Glyma.18G238600	579	192	1	Gm18: 57020427 - 57022363	5.0e-131
GmWRKY57-1	Glyma.02G115200	882	293	3	Gm02: 11099498 - 11107140	1.2e-198
GmWRKY57-2	Glyma.01G056800	894	297	3	Gm01: 7343213 - 7351310	5.4e-201
GmWRKY65-1	Glyma.08G118200	786	261	2	Gm08: 9144547 - 9146579	1.4e-178
GmWRKY65-2	Glyma.05G160800	768	255	2	Gm05: 34988297 - 34990342	6.9e-173
GmWRKY67	Glyma.03G002300	816	271	2	Gm03: 226019 - 227944	3.5e-184
GmWRKY69-1	Glyma.13G289400	798	265	2	Gm13: 37784521 - 37787501	5.1e-178
GmWRKY69-2	Glyma.12G212300	792	263	2	Gm12: 37175797 - 37178917	6.6e-177
GmWRKY70-1	Glyma.06G142000	960	319	2	Gm06: 11533270 - 11536206	3.3e-214
GmWRKY70-2	Glyma.04G223300	954	317	2	Gm04: 46258375 - 46261231	3.1e-213
GmWRKY70-3	Glyma.13G267500	891	296	2	Gm13: 35876645 - 35879941	3.5e-196
GmWRKY70-4	Glyma.13G267600	903	300	2	Gm13: 35882273 - 35884554	4.2e-200
GmWRKY70-5	Glyma.13G267700	813	270	3	Gm13: 35889478 - 35896451	3.0e-181
GmWRKY70-6	Glyma.18G213200	900	299	2	Gm18: 54287738 - 54289805	3.2e-199
GmWRKY70-7	Glyma.09G274000	903	300	2	Gm09: 45810286 - 45812389	9.7e-202
GmWRKY71-1	Glyma.05G127600	1077	358	2	Gm05: 31804493 - 31806949	6.1e-241
GmWRKY71-2	Glyma.02G285900	1014	337	2	Gm02: 49752561 - 49755310	1.0e-228
GmWRKY71-3	Glyma.14G028900	1008	335	2	Gm14: 2101859 - 2104895	2.0e-226
GmWRKY72-1	Glyma.02G020300	1584	527	3	Gm02: 1814440 - 1822699	0.0e+00
GmWRKY72-2	Glyma.01G043300	1530	509	3	Gm01: 4685834 - 4692108	0.0e+00
GmWRKY72-3	Glyma.19G020600	1488	496	3	Gm19: 2182474 - 2185528	0.0e+00
GmWRKY72-4	Glyma.09G240000	1626	541	3	Gm09: 43034331 - 43037563	0.0e+00
GmWRKY72-5	Glyma.06G190800	1848	615	4	Gm06: 16715202 - 16720374	0.0e+00
GmWRKY72-6	Glyma.05G029000	1785	594	5	Gm05: 811057 - 817892	0.0e+00
GmWRKY75-1	Glyma.08G011300	444	147	1	Gm08: 898774 - 900025	7.1e-96
GmWRKY75-2	Glyma.19G094100	567	188	1	Gm19: 33118585 - 33121094	9.0e-124
GmWRKY75-3	Glyma.16G054400	588	195	1	Gm16: 5308516 - 5311399	6.6e-129
Gossypium raimondii						
GrWRKY1-1	Gorai.007G167100	1212	403	4	Chr07: 14851777 - 14854906	5.9e-269
GrWRKY1-2	Gorai.001G192900	1326	441	4	Chr01: 32649781 - 32652799	5.2e-294
GrWRKY2-1	Gorai.004G034600	2178	725	5	Chr04: 2832238 - 2836240	0.0e+00
GrWRKY2-2	Gorai.013G179400	2292	763	5	Chr13: 47309619 - 47316117	0.0e+00
GrWRKY2-3	Gorai.012G028200	2196	731	5	Chr12: 3473845 - 3479955	0.0e+00

GrWRKY4-1	Gorai.013G090200	1470	489	3	Chr13: 14470859 - 14473687	0.0e+00
GrWRKY4-2	Gorai.012G186000	1458	185	3	Chr12: 35310771 - 35314216	0.0e+00
GrWRKY4-3	Gorai.001G241200	1365	454	3	Chr01: 48091763 - 48094329	9.2e-311
GrWRKY4-4	Gorai.012G051500	1551	516	3	Chr12: 6660221 - 6663718	0.0e+00
GrWRKY4-5	Gorai.006G013300	1527	508	3	Chr06: 2815112 - 2818026	0.0e+00
GrWRKY5-1	Gorai.004G166800	624	207	3	Chr04: 46355569 - 46358423	8.5e-142
GrWRKY5-2	Gorai.004G069500	654	217	3	Chr04: 7297650 - 7301317	8.9e-146
GrWRKY5-3	Gorai.008G109600	651	216	3	Chr08: 34056566 - 34059350	8.5e-147
GrWRKY6-1	Gorai.001G214800	1683	560	4	Chr01: 42975207 - 42978080	0.0e+00
GrWRKY6-2	Gorai.007G171900	1563	520	4	Chr07: 15750859 - 15752772	0.0e+00
GrWRKY6-3	Gorai.002G091300	1578	525	5	Chr02: 11519176 - 11521924	0.0e+00
GrWRKY6-4	Gorai.011G012700	1641	546	4	Chr11: 896914 - 899719	0.0e+00
GrWRKY6-5	Gorai.001G094300	1548	515	4	Chr01: 10451369 - 10453540	0.0e+00
GrWRKY6-6	Gorai.009G180400	1698	565	5	Chr09: 13897793 - 13900521	0.0e+00
GrWRKY7-1	Gorai.010G022600	957	318	2	Chr10: 1787230 - 1789404	4.8e-215
GrWRKY7-2	Gorai.009G214800	1077	358	2	Chr09: 16695025 - 16697303	3.1e-238
GrWRKY7-3	Gorai.007G216700	1032	343	2	Chr07: 24508940 - 24510713	4.6e-227
GrWRKY7-4	Gorai.003G026600	951	316	2	Chr03: 2409493 - 2410991	1.8e-207
GrWRKY7	Gorai.009G276100	1065	354	2	Chr09: 23139562 - 23141441	1.1e-234
GrWRKY9-1	Gorai.002G207600	1440	479	4	Chr02: 55414404 - 55416230	0.0e+00
GrWRKY9-2	Gorai.008G041400	1518	505	4	Chr08: 5422702 - 5424801	0.0e+00
GrWRKY11-1	Gorai.009G066900	993	330	2	Chr09: 4768505 - 4770622	6.5e-222
GrWRKY11-2	Gorai.001G002200	1008	335	2	Chr01: 190590 - 192301	7.7e-220
GrWRKY12	Gorai.008G201000	2559	852	13	Chr08: 48660141 - 48668419	0.0e+00
GrWRKY13-1	Gorai.007G246500	669	222	2	Chr07: 37935680 - 37941173	1.8e-155
GrWRKY13-2	Gorai.003G048100	648	215	2	Chr03: 6528418 - 6530997	2.8e-150
GrWRKY15	Gorai.004G199500	879	292	3	Chr04: 52091620 - 52094366	6.9e-198
GrWRKY17	Gorai.010G121800	1053	350	2	Chr10: 24918506 - 24920740	4.9e-229
GrWRKY18-1	Gorai.009G062400	759	252	3	Chr09: 4480963 - 4482542	4.3e-169
GrWRKY18-2	Gorai.001G037800	786	261	3	Chr01: 3527619 - 3529351	1.9e-174
GrWRKY20-1	Gorai.002G096200	1707	568	5	Chr02: 12157902 - 12162232	0.0e+00
GrWRKY20-2	Gorai.004G033200	1671	556	4	Chr04: 2693188 - 2697619	0.0e+00
GrWRKY20-3	Gorai.013G155500	1686	561	5	Chr13: 42910955 - 42915250	0.0e+00

GrWRKY21-1	Gorai.004G219300	963	320	2	Chr04: 55302762 - 55305334	1.0e-217
GrWRKY21-2	Gorai.004G219400	972	323	2	Chr04: 55312239 - 55314972	2.5e-217
GrWRKY21-3	Gorai.013G104500	996	331	3	Chr13: 20662111 - 20665102	1.9e-225
GrWRKY21-4	Gorai.007G142800	1011	336	3	Chr07: 11891564 - 11893955	3.2e-224
GrWRKY21-5	Gorai.008G200800	4023	1340	18	Chr08: 48587929 - 48599304	0.0e+00
GrWRKY22-1	Gorai.004G160100	978	325	2	Chr04: 44999954 - 45002111	2.5e-221
GrWRKY22-2	Gorai.007G122300	804	267	3	Chr07: 9668171 - 9670097	4.1e-178
GrWRKY22-3	Gorai.007G103400	1008	335	2	Chr07: 7762198 - 7764080	2.4e-223
GrWRKY23-1	Gorai.008G137900	960	319	2	Chr08: 38795821 - 38797835	2.0e-217
GrWRKY23-2	Gorai.007G121200	939	312	2	Chr07: 9561627 - 9563642	3.0e-211
GrWRKY23-3	Gorai.004G144900	867	288	2	Chr04: 40803243 - 40804795	2.6e-197
GrWRKY24-1	Gorai.004G155100	597	198	1	Chr04: 43990867 - 43992065	3.7e-135
GrWRKY24-2	Gorai.007G103600	570	189	1	Chr07: 7770570 - 7772760	9.5e-128
GrWRKY27-1	Gorai.007G013300	1050	349	2	Chr07: 1048354 - 1049982	6.8e-235
GrWRKY27-2	Gorai.008G273700	1191	396	2	Chr08: 55234288 - 55236130	1.6e-267
GrWRKY28-1	Gorai.011G041400	945	314	2	Chr11: 3079853 - 3081670	1.3e-212
GrWRKY28-2	Gorai.009G157300	960	319	2	Chr09: 12048719 - 12050826	5.5e-219
GrWRKY28-3	Gorai.010G164700	831	276	2	Chr10: 47672133 - 47673899	4.0e-191
GrWRKY29-1	Gorai.003G085500	840	279	2	Chr03: 21690773 - 21692050	7.7e-191
GrWRKY29-2	Gorai.008G250300	900	299	2	Chr08: 53423419 - 53424581	3.0e-207
GrWRKY32-1	Gorai.010G113000	1491	491	5	Chr10: 21667464 - 21671276	0.0e+00
GrWRKY32-2	Gorai.001G032200	1509	502	4	Chr01: 2983827 - 2987746	0.0e+00
GrWRKY33-1	Gorai.012G119600	1536	511	4	Chr12: 27422106 - 27424439	0.0e+00
GrWRKY33-2	Gorai.003G150700	1722	573	4	Chr03: 41825754 - 41828702	0.0e+00
GrWRKY35-1	Gorai.011G006600	1158	385	2	Chr11: 524468 - 527564	1.1e-262
GrWRKY35-2	Gorai.009G116800	1386	461	3	Chr09: 8576437 - 8580079	1.1e-312
GrWRKY35-3	Gorai.008G138900	717	238	3	Chr08: 38956335 - 38958108	3.2e-161
GrWRKY40-1	Gorai.010G222400	981	326	3	Chr10: 59312770 - 59314535	2.3e-207
GrWRKY40-2	Gorai.009G124000	933	310	4	Chr09: 9269894 - 9271911	8.8e-203
GrWRKY40-3	Gorai.001G037700	918	305	4	Chr01: 3501855 - 3503815	8.5e-204
GrWRKY40-4	Gorai.010G118300	939	312	4	Chr10: 23470804 - 23472932	4.2e-209
GrWRKY40-5	Gorai.009G062300	942	313	4	Chr09: 4477630 - 4479767	6.6e-204
GrWRKY41-1	Gorai.004G134600	1068	355	2	Chr04: 36928943 - 36930774	8.5e-243

GrWRKY41-2	Gorai.008G253300	1068	355	2	Chr08: 53652234 - 53654501	4.1e-239
GrWRKY42	Gorai.010G182500	1656	551	4	Chr10: 53223679 - 53226173	0.0e+00
GrWRKY44	Gorai.009G421200	1401	466	6	Chr09: 65839443 - 65843032	2.0e-312
GrWRKY45-1	Gorai.012G085100	594	197	1	Chr12: 14499483 - 14501846	8.3e-133
GrWRKY45-2	Gorai.001G057600	486	161	1	Chr01: 5682018 - 5684119	3.3e-106
GrWRKY46-1	Gorai.007G107300	999	332	2	Chr07: 8060718 - 8063303	2.2e-225
GrWRKY46-2	Gorai.001G155800	1008	335	3	Chr01: 22002864 - 22004803	6.0e-227
GrWRKY47-1	Gorai.001G147900	1587	528	4	Chr01: 20252418 - 20255849	0.0e+00
GrWRKY47-2	Gorai.007G117200	1518	505	4	Chr07: 9168972 - 9172053	0.0e+00
GrWRKY47-3	Gorai.008G134300	1524	507	5	Chr08: 38158827 - 38164045	0.0e+00
GrWRKY48-1	Gorai.005G068100	1074	357	3	Chr05: 7292801 - 7295049	1.1e-245
GrWRKY48-2	Gorai.006G265200	879	292	2	Chr06: 50418693 - 50420443	1.4e-198
GrWRKY48-3	Gorai.011G201800	1071	356	2	Chr11: 48923571 - 48925654	9.4e-238
GrWRKY49-1	Gorai.012G104800	894	297	2	Chr12: 23539684 - 23541270	1.4e-200
GrWRKY49-2	Gorai.006G171700	903	300	2	Chr06: 43103488 - 43105647	1.2e-203
GrWRKY50-1	Gorai.010G098000	474	157	2	Chr10: 17032215 - 17033572	7.5e-105
GrWRKY50-2	Gorai.001G021500	522	173	2	Chr01: 2035562 - 2036973	5.8e-119
GrWRKY51-1	Gorai.003G047800	633	210	2	Chr03: 6483440 - 6487119	2.7e-123
GrWRKY51-2	Gorai.007G245200	546	181	2	Chr07: 36552367 - 36556177	2.7e-123
GrWRKY51-3	Gorai.011G114200	441	146	2	Chr11: 14639253 - 14641404	1.9e-97
GrWRKY51-4	Gorai.010G131300	543	180	2	Chr10: 28630558 - 28632381	2.7e-123
GrWRKY51-5	Gorai.009G259600	516	171	2	Chr09: 21405533 - 21406771	4.3e-117
GrWRKY53-1	Gorai.007G014600	999	332	2	Chr07: 1129337 - 1131192	1.5e-223
GrWRKY53-2	Gorai.008G270600	861	286	2	Chr08: 54962950 - 54964147	1.3e-195
GrWRKY54	Gorai.013G008300	918	305	2	Chr13: 556171 - 558759	1.6e-209
GrWRKY55	Gorai.012G014000	1122	373	2	Chr12: 1570284 - 1572777	1.5e-254
GrWRKY57-1	Gorai.013G092300	927	308	4	Chr13: 14878681 - 14885458	8.5e-207
GrWRKY57-2	Gorai.005G144500	900	299	3	Chr05: 39141475 - 39145239	1.4e-201
GrWRKY57-3	Gorai.008G178100	930	309	3	Chr08: 45484086 - 45491221	3.1e-210
GrWRKY65-1	Gorai.010G161000	831	276	2	Chr10: 45946955 - 45948589	2.2e-185
GrWRKY65-2	Gorai.009G160800	834	277	2	Chr09: 12340497 - 12342145	9.7e-185
GrWRKY69	Gorai.011G037500	825	274	2	Chr11: 2807773 - 2809365	8.1e-182
GrWRKY70-1	Gorai.009G292600	918	305	2	Chr09: 25326109 - 25327589	5.1e-207

GrWRKY70-2	Gorai.012G013900	909	302	2	Chr12: 1557156 - 1558483	2.9e-211
GrWRKY70-3	Gorai.010G219200	984	327	2	Chr10: 58811633 - 58814117	4.3e-223
GrWRKY70-4	Gorai.005G003900	924	307	2	Chr05: 216589 - 219052	1.2e-205
GrWRKY71-1	Gorai.006G008200	906	301	2	Chr06: 1694301 - 1696794	5.4e-202
GrWRKY71-2	Gorai.012G040700	957	318	2	Chr12: 5025230 - 5027054	4.4e-216
GrWRKY72-1	Gorai.013G082300	1458	485	2	Chr13: 11310159 - 11313327	0.0e+00
GrWRKY72-2	Gorai.008G191200	1824	607	5	Chr08: 47423917 - 47427268	0.0e+00
GrWRKY72-3	Gorai.012G056800	1665	554	4	Chr12: 7726941 - 7729440	0.0e+00
GrWRKY72-4	Gorai.006G025700	1503	500	3	Chr06: 6506690 - 6509020	0.0e+00
GrWRKY74-1	Gorai.004G247000	1002	333	2	Chr04: 58396398 - 58398829	1.5e-225
GrWRKY74-2	Gorai.007G278400	981	326	2	Chr07: 47621852 - 47623805	3.5e-219
GrWRKY75-1	Gorai.002G181600	453	150	1	Chr02: 47717326 - 47718427	2.2e-99
GrWRKY75-2	Gorai.005G164300	543	180	1	Chr05: 47752588 - 47754653	2.2e-118
GrWRKY75-3	Gorai.011G086300	498	165	1	Chr11: 8976191 - 8976914	5.1e-110
GrWRKY75-4	Gorai.001G273100	501	166	1	Chr01: 54991732 - 54993814	1.4e-110
GrWRKY75-5	Gorai.006G043200	477	158	1	Chr06: 12551443 - 12553732	1.8e-107
Linum usitatissimum						
LuWRKY1-1	Lus10006368.g	1521	506	3	scaffold1603: 52507 - 54326	3.0e-134
LuWRKY1-2	Lus10012322.g	1215	404	3	scaffold572: 127645 - 129160	2.5e-118
LuWRKY2-1	Lus10020215.g	1656	551	2	scaffold454: 660146 - 662154	5.9e-172
LuWRKY2-2	Lus10026838.g	2121	706	4	scaffold651: 66042 - 69780	1.2e-223
LuWRKY3-1	Lus10000173.g	966	321	3	C8365651: 13 - 1492	2.2e-116
LuWRKY3-2	Lus10036800.g	1290	429	3	scaffold31: 683634 - 685437	5.5e-131
LuWRKY3-3	Lus10037131.g	1290	429	3	scaffold462: 325462 - 327216	4.6e-134
LuWRKY3-4	Lus10012029.g	1386	461	3	scaffold931: 236215 - 238268	2.9e-46
LuWRKY3-5	Lus10012030.g	3597	1198	9	scaffold931: 239236 - 244342	6.1e-120
LuWRKY3-6	Lus10016282.g	3534	1177	9	scaffold947: 600805 - 605914	1.6e-112
LuWRKY3-7	Lus10012027.g	2850	949	9	scaffold931: 228831 - 233267	2.2e-143
LuWRKY4-1	Lus10030424.g	1371	456	3	scaffold917: 64180 - 66112	1.3e-135
LuWRKY4-2	Lus10026634.g	1326	441	3	scaffold617: 764017 - 765881	3.2e-134
LuWRKY5	Lus10012678.g	948	327	3	scaffold206: 8356 - 19449	6.5e-54
LuWRKY6-1	Lus10035286.g	1641	546	4	scaffold151: 64617 - 67212	4.1e-149
LuWRKY6-2	Lus10030040.g	1845	614	4	scaffold416: 1190676 - 1193392	8.4e-114

LuWRKY7	Lus10010053.g	1035	344	2	scaffold621: 251285 - 252496	1.2e-124
LuWRKY9-1	Lus10035971.g	1332	443	4	scaffold76: 505514 - 507425	3.7e-72
LuWRKY9-2	Lus10016682.g	1419	472	4	scaffold903: 2155 - 4199	1.6e-61
LuWRKY10	Lus10007326.g	804	267	1	scaffold302: 8668 - 10627	4.2e-32
LuWRKY11	Lus10020136.g	978	325	2	scaffold454: 229747 - 231386	1.0e-148
LuWRKY13	Lus10020832.g	798	265	2	scaffold711: 113031 - 118623	8.1e-54
LuWRKY15-1	Lus10041600.g	1044	347	2	scaffold272: 863959 - 865252	6.8e-134
LuWRKY15-2	Lus10006261.g	1047	348	2	scaffold535: 180482 - 181774	6.0e-134
LuWRKY17-1	Lus10005428.g	729	242	2	scaffold847: 91419 - 92992	3.4e-79
LuWRKY17-2	Lus10015229.g	798	265	2	scaffold924: 55542 - 57259	5.5e-77
LuWRKY17-3	Lus10026936.g	1011	336	2	scaffold651: 654889 - 656616	4.0e-152
LuWRKY20-1	Lus10016595.g	1686	561	5	scaffold915: 125806 - 129807	1.3e-237
LuWRKY20-2	Lus10003769.g	1797	598	4	scaffold566: 65332 - 69224	8.8e-227
LuWRKY20-3	Lus10032580.g	1185	394	3	scaffold140: 846806 - 849016	2.3e-137
LuWRKY20-4	Lus10043167.g	1572	523	5	scaffold25: 1539592 - 1543223	3.0e-184
LuWRKY21-1	Lus10014745.g	1071	356	2	scaffold584: 470023 - 471587	3.4e-170
LuWRKY21-2	Lus10033857.g	1068	355	2	scaffold222: 636777 - 638353	2.1e-168
LuWRKY21-3	Lus10018776.g	1008	335	2	scaffold461: 674915 - 676405	9.4e-136
LuWRKY21-4	Lus10024864.g	996	331	2	scaffold473: 458232 - 459707	8.5e-138
LuWRKY22-1	Lus10007907.g	1116	371	2	scaffold1317: 220425 - 221721	5.6e-87
LuWRKY22-2	Lus10036399.g	738	245	2	scaffold57: 489278 - 490202	4.6e-70
LuWRKY22-3	Lus10018815.g	1554	517	2	scaffold103: 2658 - 4496	1.4e-47
LuWRKY22-4	Lus10005132.g	393	130	0	scaffold846: 217286 - 217678	1.4e-15
LuWRKY26-1	Lus10012215.g	1641	546	4	scaffold273: 33449 - 35852	1.1e-182
LuWRKY26-2	Lus10001265.g	1557	518	4	scaffold352: 61863 - 64300	1.1e-180
LuWRKY27-1	Lus10024676.g	813	270	1	scaffold349: 723578 - 725166	9.5e-56
LuWRKY27-2	Lus10032303.g	891	296	1	scaffold291: 638036 - 639613	4.5e-60
LuWRKY29-1	Lus10027538.g	1239	412	2	scaffold96: 846700 - 848690	1.1e-71
LuWRKY29-2	Lus10039295.g	1005	334	2	scaffold33: 386387 - 388263	1.3e-64
LuWRKY30-1	Lus10001062.g	1056	351	2	scaffold1286: 38285 - 39851	6.5e-68
LuWRKY30-2	Lus10039331.g	1095	364	2	scaffold33: 601835 - 603987	2.9e-72
LuWRKY32-1	Lus10036268.g	1641	546	4	scaffold27: 646378 - 649748	4.0e-154
LuWRKY32-2	Lus10022150.g	1638	545	4	scaffold342: 149803 - 153178	6.4e-153

LuWRKY33-1	Lus10042243.g	1470	489	3	scaffold123: 1371062 - 1373109	1.3e-167
LuWRKY33-2	Lus10026409.g	633	210	1	scaffold898: 992906 - 993891	4.4e-93
LuWRKY33-3	Lus10022278.g	246	81	0	scaffold225: 517284 - 517529	1.1e-58
LuWRKY34-1	Lus10027139.g	2154	717	4	scaffold297: 1054688 - 1057345	5.0e-283
LuWRKY34-2	Lus10032887.g	2154	717	4	scaffold51: 35260 - 37924	1.5e-284
LuWRKY35	Lus10038426.g	885	294	2	scaffold28: 1265768 - 1268401	2.1e-84
LuWRKY36-1	Lus10037205.g	1431	476	2	scaffold462: 752584 - 754753	1.0e-93
LuWRKY36-2	Lus10036725.g	1446	481	2	scaffold31: 259503 - 261843	1.2e-95
LuWRKY39	Lus10021999.g	948	315	2	scaffold87: 224294 - 225588	6.1e-97
LuWRKY40-1	Lus10024074.g	1077	358	3	scaffold353: 394443 - 395811	1.7e-108
LuWRKY40-2	Lus10041660.g	1089	362	3	scaffold272: 1143406 - 1144792	1.4e-104
LuWRKY40-3	Lus10026082.g	1062	353	3	scaffold319: 613216 - 614689	1.3e-95
LuWRKY40-4	Lus10002309.g	1059	352	3	scaffold120: 38671 - 40247	2.4e-97
LuWRKY41-1	Lus10003894.g	966	321	2	scaffold254: 98824 - 99948	1.5e-95
LuWRKY41-2	Lus10001902.g	975	324	2	scaffold255: 35388 - 36525	1.2e-99
LuWRKY42-1	Lus10022959.g	1644	547	4	scaffold355: 304446 - 307009	7.2e-109
LuWRKY42-2	Lus10021554.g	1698	565	3	scaffold362: 756559 - 761238	1.7e-104
LuWRKY42-3	Lus10015546.g	1062	353	1	scaffold860: 497500 - 498692	4.1e-79
LuWRKY43-1	Lus10007906.g	669	222	1	scaffold1317: 212017 - 214482	2.2e-69
LuWRKY43-2	Lus10036401.g	648	215	1	scaffold57: 497491 - 499055	1.6e-70
LuWRKY44	Lus10019898.g	1263	420	6	scaffold1491: 565522 - 568464	1.6e-95
LuWRKY47-1	Lus10038028.g	1332	443	4	scaffold475: 930490 - 933471	4.6e-88
LuWRKY47-2	Lus10009969.g	627	208	1	scaffold1630: 41523 - 42774	2.3e-67
LuWRKY48-1	Lus10037785.g	1086	361	2	scaffold196: 1563871 - 1565325	3.9e-88
LuWRKY48-2	Lus10017067.g	1080	359	2	scaffold216: 45324 - 46783	1.4e-85
LuWRKY49-1	Lus10010851.g	693	230	2	scaffold440: 202315 - 203217	3.2e-54
LuWRKY49-2	Lus10024380.g	831	276	2	scaffold16: 599062 - 600091	1.2e-61
LuWRKY50	Lus10006848.g	741	246	2	scaffold1356: 94321 - 95261	3.3e-55
LuWRKY51-1	Lus10035841.g	663	220	2	scaffold464: 1579044 - 1580019	1.4e-45
LuWRKY51-2	Lus10036624.g	642	213	2	scaffold57: 1512688 - 1513791	1.9e-50
LuWRKY53-1	Lus10032372.g	1080	359	2	scaffold291: 969635 - 970903	2.5e-126
LuWRKY53-2	Lus10023099.g	1089	362	2	scaffold325: 379977 - 381261	3.5e-123
LuWRKY54-1	Lus10014177.g	906	301	1	scaffold231: 280648 - 282205	2.3e-41

LuWRKY54-2	Lus10022736.g	894	297	1	scaffold59: 702778 - 704104	1.6e-35
LuWRKY54-3	Lus10030517.g	951	316	2	scaffold917: 531187 - 532493	7.0e-45
LuWRKY54-4	Lus10012870.g	924	307	2	scaffold1313: 273878 - 275141	8.3e-44
LuWRKY55	Lus10029022.g	771	256	2	scaffold376: 128139 - 130867	3.3e-58
LuWRKY57-1	Lus10036891.g	1053	350	2	scaffold31: 1105718 - 1110475	4.8e-81
LuWRKY57-2	Lus10037094.g	1065	354	2	scaffold462: 182308 - 186177	2.9e-80
LuWRKY58-1	Lus10033000.g	1569	522	3	scaffold51: 1026515 - 1029919	1.6e-193
LuWRKY58-2	Lus10015377.g	1617	538	3	scaffold635: 207217 - 210613	2.6e-217
LuWRKY61	Lus10023615.g	1086	561	2	scaffold505: 169590 - 171705	8.0e-94
LuWRKY65-1	Lus10029252.g	735	244	2	scaffold360: 247939 - 249537	2.6e-63
LuWRKY65-2	Lus10007305.g	579	192	2	scaffold859: 180772 - 182205	3.0e-64
LuWRKY70-1	Lus10029021.g	882	293	2	scaffold376: 124994 - 127131	1.1e-51
LuWRKY70-2	Lus10034244.g	882	293	2	scaffold310: 226693 - 228086	1.6e-53
LuWRKY70-3	Lus10025216.g	1002	333	1	scaffold339: 28914 - 31054	2.3e-53
LuWRKY70-4	Lus10025133.g	966	321	1	scaffold305: 441610 - 443615	3.2e-53
LuWRKY71-1	Lus10004612.g	930	309	1	scaffold1170: 176700 - 178433	6.3e-77
LuWRKY71-2	Lus10004537.g	846	281	1	scaffold48: 15521 - 17335	6.5e-79
LuWRKY72	Lus10024246.g	2355	784	5	scaffold165: 704003 - 709151	6.0e-104
LuWRKY74	Lus10042538.g	936	311	2	scaffold67: 206729 - 208018	1.3e-96
LuWRKY75-1	Lus10011346.g	495	164	1	scaffold744: 89855 - 91916	3.2e-58
LuWRKY75-2	Lus10003128.g	480	159	1	scaffold1847: 55599 - 57808	3.4e-59
LuWRKY75-3	Lus10012547.g	357	118	1	scaffold6: 241387 - 244335	2.6e-59
LuWRKY75-4	Lus10041546.g	621	206	1	scaffold272: 628883 - 630242	3.2e-61
Malus domestica						
MdWRKY1-1	MDP0000256105	1455	484	3	Chr9: MDC002626.191: 15548 - 17594	5.4e-229
MdWRKY1-2	MDP0000260803	1458	485	3	Chr17: MDC017641.109: 702 - 3071	4.6e-209
MdWRKY2-1	MDP0000144203	2181	726	4	Chr4: MDC021803.105: 6499 - 9863	0.0e+00
MdWRKY2-2	MDP0000184044	2898	965	7	Chr12: MDC008173.377: 8987 - 14024	0.0e+00
MdWRKY3-1	MDP0000708692	1563	520	4	Chr4: MDC017895.316: 14103 - 16075	0.0e+00
MdWRKY3-2	MDP0000258212	1689	562	4	Chr16: MDC010756.337: 9879 - 12269	1.1e-257
MdWRKY4	MDP0000125782	1311	436	1	Chr13: MDC010756.328: 57371 -59642	4.1e-209
MdWRKY6	MDP0000301666	1890	629	5	Chr5: MDC015642.226: 3597 - 6162	0.0e+00
MdWRKY7	MDP0000129882	1056	351	2	Chr8: MDC017495.204: 3145 - 4808	1.6e-171

MdWRKY9-1	MDP0000179719	1719	572	4	Chr8: MDC007295.376: 5455 - 7851	6.4e-212
MdWRKY9-2	MDP0000137704	1821	606	7	Chr15: MDC005436.516: 4056 - 8281	1.1e-204
MdWRKY11-1	MDP0000130400	843	280	2	Unanch: MDC018338.206: 12128 -13229	5.8e-186
MdWRKY11-2	MDP0000716551	1149	382	3	Chr8: MDC011219.436: 764 - 3783	1.2e-222
MdWRKY11-3	MDP0000200748	495	164	2	Chr15: MDC013859.580: 2376 - 4634	2.5e-47
MdWRKY11-4	MDP0000185288	1026	341	2	Chr8: MDC011219.444: 11643 - 13146	6.3e-223
MdWRKY11-5	MDP0000272940	1017	338	2	Chr15: MDC004030.356: 5510 - 7048	1.4e-201
MdWRKY11-6	MDP0000381897	306	101	2	Chr15: MDC015490.71: 287 - 1652	3.3e-42
MdWRKY11-7	MDP0000184308	1278	425	3	Unanch: MDC008403.468: 4880 - 6990	1.1e-42
MdWRKY13-1	MDP0000259279	813	270	2	Chr8: MDC009195.339: 1419 - 4941	3.3e-187
MdWRKY13-2	MDP0000176224	963	320	5	Chr14: MDC006681.275: 14834 - 19349	6.0e-153
MdWRKY13-3	MDP0000517924	357	118	2	Chr2: MDC008679.195: 521 - 8016	1.1e-34
MdWRKY14	MDP0000566005	1614	537	4	Chr10: MDC010643.143: 15821 - 26034	2.5e-220
MdWRKY15-1	MDP0000231668	1029	342	2	Chr15: MDC015663.88: 14239 - 15750	1.8e-162
MdWRKY15-2	MDP0000946614	846	281	2	Chr3: MDC025970.13: 5867 - 6933	2.0e-156
MdWRKY18	MDP0000418900	477	158	1	Chr9: MDC000374.165: 15658 - 16205	7.4e-82
MdWRKY20-1	MDP0000294643	1029	342	2	Unanch: MDC013694.186: 527 - 3119	7.3e-175
MdWRKY20-2	MDP0000700887	564	187	0	Chr17: MDC017580.221: 10577 - 11140	3.0e-94
MdWRKY20-3	MDP0000184361	2079	692	9	Chr17: MDC007214.348: 13143 - 18505	0.0e+00
MdWRKY20-4	MDP0000289397	1851	616	6	Chr11: MDC010860.364: 12978 - 17093	0.0e+00
MdWRKY20-5						4.4e-205
MdWRKY21-1	MDP0000322257	2892	963	7	Chr6: MDC014216.250: 6886 - 12974	1.1e-249
MdWRKY21-2	MDP0000281965	1314	437	5	Chr10: MDC006536.318: 58151 - 61410	4.9e-228
MdWRKY22-1	MDP0000602139	1020	339	2	Chr1: MDC004965.191: 448 - 1728	1.3e-149
MdWRKY22-2	MDP0000909869	1047	348	2	Chr7: MDC010007.148: 5940 - 7212	1.2e-157
MdWRKY23-1	MDP0000657441	1155	384	3	Chr17: MDC016387.187: 17247 - 28445	5.2e-187
MdWRKY23-2	MDP0000127976	1053	350	2	Chr9: MDC013713.363: 12874 - 14316	4.0e-230
MdWRKY23-3	MDP0000652760	558	186	1	Chr9: MDC005142.216: 258 - 984	2.0e-119
MdWRKY23-4	MDP0000134105	1053	350	2	Chr9: MDC043608.9: 3250 - 4693	1.6e-229
MdWRKY24	MDP0000319700	4734	1577	13	Chr1: MDC004623.338: 5710 - 23201	0.0e+00
MdWRKY25	MDP0000849514	1581	526	3	Chr13: MDC001533.397: 54381 - 57091	1.8e-257
MdWRKY26	MDP0000648338	2202	733	4	Chr3: MDC010276.213: 1385 - 4453	0.0e+00
MdWRKY27-1	MDP0000294526	1821	606	3	Chr1: MDC012574.173: 12862 - 15231	1.9e-131

MdWRKY27-2	MDP0000135668	1302	433	2	Chr7: MDC001868.399: 37519 - 39078	5.5e-120
MdWRKY28-1	MDP0000118810	960	319	2	Chr5: MDC000149.363: 8502 - 10189	6.4e-143
MdWRKY28-2	MDP0000119590	984	327	2	Chr10: MDC001276.321: 20168 - 21851	4.2e-149
MdWRKY29-1	MDP0000247896	951	316	1	Chr7: MDC002450.221: 7666 - 9027	8.4e-125
MdWRKY29-2	MDP0000145953	765	254	1	Chr2: MDC002450.233: 5350 - 6613	2.2e-108
MdWRKY30-1	MDP0000455180	1044	347	2	Chr1: MDC004961.346: 7359 - 9364	8.2e-230
MdWRKY30-2	MDP0000191017	1071	356	2	Chr7: MDC010509.242: 9638 - 11856	1.6e-233
MdWRKY30-3	MDP0000727570	1044	347	2	Chr1: MDC016134.91: 5469 - 7475	8.2e-230
MdWRKY31	MDP0000263961	1059	352	6	Chr8: MDC000897.321: 208 - 4647	6.0e-83
MdWRKY32-1	MDP0000131218	1452	483	4	Chr15: MDC019618.62: 6578 - 9907	1.1e-248
MdWRKY32-2	MDP0000293456	1434	477	4	Chr2: MDC015165.59: 3226 - 6251	4.4e-234
MdWRKY33-1	MDP0000514115	1716	571	4	Chr3: MDC031925.10: 2839 - 5383	0.0e+00
MdWRKY33-2	MDP0000296025	1539	512	4	Chr12: MDC013913.203: 6056 - 8016	0.0e+00
MdWRKY33-3	MDP0000195385	1530	509	4	Chr12: MDC013913.134: 1721 - 3683	6.8e-309
MdWRKY33-4	MDP0000507805	1395	464	4	Chr3: MDC005987.255: 1605 - 3823	9.1e-312
MdWRKY33-5	MDP0000935996	1719	572	4	Chr11: MDC004410.707: 32932 - 35500	0.0e+00
MdWRKY34	MDP0000431358	2202	733	4	Chr3: MDC010276.288: 1035 - 4100	0.0e+00
MdWRKY35-1	MDP0000168871	1449	482	2	Chr14: MDC005368.121: 11847 - 16381	7.4e-215
MdWRKY35-2	MDP0000202292	807	268	2	Chr11: MDC013433.236: 57726 - 59087	3.7e-174
MdWRKY35-3	MDP0000294489	783	260	2	Chr3: MDC012992.425: 7822 - 9169	5.0e-139
MdWRKY36	MDP0000228838	837	278	3	Chr8: MDC026664.26: 3063 - 4394	1.4e-83
MdWRKY40-1	MDP0000177906	966	321	4	Chr17: MDC007908.251: 16040 - 17803	4.4e-205
MdWRKY40-2	MDP0000794439	963	320	4	Chr14: MDC018238.148: 9566 - 11266	5.3e-183
MdWRKY40-3	MDP0000307516	1005	334	4	Chr8: MDC017768.324: 6325 - 8169	4.5e-221
MdWRKY41-1	MDP0000468391	1044	347	2	Chr1: MDC010557.340: 14408 - 15940	1.1e-158
MdWRKY41-2	MDP0000299114	1056	351	2	Chr1: MDC015340.297: 16390 - 17961	7.8e-155
MdWRKY41-3	MDP0000146360	1056	351	2	Chr1: MDC000896.238: 3076 - 4647	7.8e-155
MdWRKY41-4	MDP0000767097	1029	342	2	Chr7: MDC019500.163: 20847 - 22392	6.6e-149
MdWRKY41-5	MDP0000315045	1068	355	2	Unanch: MDC022185.237: 26011 - 27819	4.5e-150
MdWRKY41-6	MDP0000219647	1044	347	2	Chr1: MDC019500.121: 805 - 2337	1.1e-158
MdWRKY42	MDP0000935652	1836	611	5	Chr10: MDC010173.125: 37712 - 40213	0.0e+00
MdWRKY43-1	MDP0000119031	669	222	1	Chr7: MDC000480.230: 1775 - 3275	2.1e-80
MdWRKY43-2	MDP0000228328	669	222	1	Chr7: MDC025197.34: 350 - 1850	2.1e-80

MdWRKY43-3	MDP0000288378	1020	339	5	Chr1: MDC011074.351: 2492 - 8514	8.7e-61
MdWRKY43-4	MDP0000241967	597	198	3	Chr1: MDC013240.189: 360 - 3733	2.2e-32
MdWRKY44-1	MDP0000169621	1416	471	4	Chr4: MDC004911.314: 16053 - 18330	2.3e-312
MdWRKY44-2	MDP0000268364	1413	470	4	Chr12: MDC000739.241: 8358 - 11295	6.7e-274
MdWRKY44-3	MDP0000598428	243	80	1	Chr5: MDC011122.248: 3936 - 4591	2.5e-58
MdWRKY44-4	MDP0000788581	441	146	1	Chr4: MDC006671.459: 1958 - 2799	9.5e-148
MdWRKY45-1	MDP0000836661	453	150	1	Chr6: MDC000005.468: 2938 - 3715	6.7e-55
MdWRKY45-2	MDP0000263900	408	135	1	Chr14: MDC001963.416: 5302 - 6051	2.2e-52
MdWRKY47	MDP0000263349	861	286	3	Chr1: MDC006277.244: 24504 - 26011	4.7e-84
MdWRKY48-1	MDP0000256514	1116	371	2	Chr16: MDC002916.99: 7457 - 9143	3.9e-207
MdWRKY48-2	MDP0000146390	1158	385	2	Chr13: MDC003788.288: 27928 - 29658	4.9e-255
MdWRKY49-1	MDP0000205962	891	297	2	Chr12: MDC016775.152: 1568 - 3459	2.4e-153
MdWRKY49-2	MDP0000215371	948	315	3	Chr4: MDC016775.162: 8241 - 10132	6.0e-151
MdWRKY50-1	MDP0000480547	768	255	4	Chr8: MDC022670.164: 6713 - 11639	9.9e-51
MdWRKY50-2	MDP0000121669	486	161	2	Chr6: MDC004691.361: 1254 - 2110	9.6e-58
MdWRKY50-3	MDP0000299555	759	252	6	Chr8: MDC014225.232: 5563 - 7509	4.1e-37
MdWRKY51	MDP0000253189	654	217	3	Chr17: MDC033588.6: 680 - 4284	2.8e-69
MdWRKY54	MDP0000460139	1035	344	2	Chr12: MDC007698.491: 6619 - 9223	2.1e-140
MdWRKY55	MDP0000828055	1029	342	2	Chr4: MDC035519.7: 14033 - 16185	1.2e-134
MdWRKY56	MDP0000756254	192	63	1	Chr1: MDC013411.132: 2593 - 2878	7.4e-38
MdWRKY57-1	MDP0000707539	741	246	2	Chr16: MDC021902.108: 14699 - 17114	1.6e-113
MdWRKY57-2	MDP0000193216	1197	398	3	Chr13: MDC010585.196: 20669 - 23938	1.3e-133
MdWRKY58	MDP0000179145	1587	528	3	Chr9: MDC008132.471: 25217 - 28758	0.0e+00
MdWRKY60	MDP0000689162	912	303	3	Chr1: MDC006277.244: 9521 - 10969	3.4e-160
MdWRKY61-1	MDP0000290374	1365	455	3	Chr17: MDC008814.323: 30424 - 32679	7.9e-173
MdWRKY61-2	MDP0000757476	135	44	1	Chr15: MDC016408.252: 7302 - 7710	9.1e-29
MdWRKY61-3	MDP0000273851	1731	576	3	Chr13: MDC004746.122: 2816 - 6582	4.9e-218
MdWRKY64	MDP0000128463	993	330	2	Chr2: MDC015181.101: 1734 - 2965	2.4e-214
MdWRKY65-1	MDP0000133918	801	266	2	Chr10: MDC037719.8: 4105 - 5329	6.0e-176
MdWRKY65-2	MDP0000304113	822	273	2	Chr5: MDC015524.135: 6399 - 7605	1.4e-149
MdWRKY67	MDP0000128464	1029	342	1	Chr17: MDC015186.45: 193 - 2000	4.4e-205
MdWRKY69	MDP0000676216	1065	354	4	Chr9: MDC013104.664: 2624 - 9198	8.5e-108
MdWRKY70-1	MDP0000754989	1368	455	4	Chr12: MDC042136.8: 7167 - 15527	1.5e-102

MdWRKY70-2	MDP0000228304	1089	362	4	Chr4: MDC035519.7: 9735 - 13107	6.2e-104
MdWRKY70-3	MDP0000175240	912	303	2	Chr12: MDC007698.491: 10296 - 13111	1.0e-117
MdWRKY71-1	MDP0000505247	1359	452	5	Chr9: MDC025315.21: 129 - 8192	5.9e-160
MdWRKY71-2	MDP0000257129	1098	365	2	Chr17: MDC005054.249: 11130 - 13484	1.8e-171
MdWRKY72-1	MDP0000161881	1782	593	5	Chr16: MDC000416.241: 1247 - 3883	1.6e-214
MdWRKY72-2	MDP0000416279	1758	585	4	Chr11: MDC006102.355: 6753 - 10026	2.6e-252
MdWRKY72-3	MDP0000452559	1239	412	2	Chr9: MDC008814.320: 9734 - 11448	1.3e-166
MdWRKY72-4	MDP0000196330	1758	585	4	Chr11: MDC011934.106: 307 - 3580	6.4e-254
MdWRKY72-5	MDP0000300712	2217	738	7	Chr6: MDC016784.314: 9788 - 15070	1.2e-264
MdWRKY72-6	MDP0000308261	2124	707	6	Chr6: MDC017781.114: 37370 - 42624	6.2e-265
MdWRKY74-1	MDP0000130716	993	330	2	Unanchor:MDC018874.95: 1608 - 2839	2.3e-214
MdWRKY74-2	MDP0000234335	960	319	3	Chr12: MDC016084.155: 9369 - 10917	2.3e-86
MdWRKY74-3	MDP0000231993	978	325	2	Chr4: MDC011695.186: 1697 - 2966	4.4e-124
MdWRKY75-1	MDP0000123888	573	190	1	Chr16: MDC008000.234: 1309 - 3257	1.4e-123
MdWRKY75-2	MDP0000142583	672	223	1	Chr17: MDC021067.62: 11874 - 13752	1.3e-85
MdWRKY75-3	MDP0000154734	672	233	2	Chr9: MDC023147.45: 4965 - 7678	4.1e-77
MdWRKY75-4	MDP0000263768	453	150	1	Chr6: MDC001645.308: 35228 - 36005	6.7e-55
MdWRKY75-5	MDP0000123467	192	63	1	Chr9: MDC007407.185: 1981 - 2255	7.2e-32
MdWRKY75-6	MDP0000792088	681	226	2	Chr13: MDC018107.153: 10042 - 11712	9.1e-103
Manihot esculenta						
MeWRKY1-2	cassava4.1_008041m.g	1299	432	5	scaffold07536: 38292 - 40423	7.4e-285
MeWRKY1-1	cassava4.1_009560m.g	1155	384	3	scaffold03317: 145274 - 147528	2.1e-256
MeWRKY2	cassava4.1_002389m.g	2214	737	5	scaffold03203: 179429 - 183397	0.0e+00
MeWRKY3-1	cassava4.1_006823m.g	1425	474	3	scaffold04233: 63799 - 68037	0.0e+00
MeWRKY3-2	cassava4.1_005267m.g	1605	534	3	scaffold06609: 532363 - 538474	0.0e+00
MeWRKY4-1	cassava4.1_005912m.g	1527	508	3	scaffold07873: 164300 - 167739	2.4e-221
MeWRKY4-2	cassava4.1_005605m.g	1560	519	3	scaffold11341: 800966 - 805076	0.0e+00
MeWRKY5-1	cassava4.1_027780m.g	618	205	3	scaffold11050: 5538 - 7515	4.4e-140
MeWRKY5-2	cassava4.1_016397m.g	618	205	3	scaffold08265: 3820169 - 3823424	6.2e-139
MeWRKY6-1	cassava4.1_021298m.g	549	183	3	scaffold12690: 49254 - 50156	2.1e-69
MeWRKY6-2	cassava4.1_024001m.g	1728	575	4	scaffold11251: 26632 - 28740	0.0e+00
MeWRKY6-3	cassava4.1_004331m.g	1749	582	5	scaffold06598: 271081 - 273620	0.0e+00
MeWRKY6-4	cassava4.1_030395m.g	1863	620	4	scaffold08001: 33833 - 36074	0.0e+00

MeWRKY6-5	cassava4.1_003305m.g	1953	650	4	scaffold03414: 3412 - 6072	1.1e-308
MeWRKY7-1	cassava4.1_010539m.g	1077	358	2	scaffold03484: 130961 - 132547	5.1e-238
MeWRKY7-2	cassava4.1_010007m.g	1113	370	2	scaffold00492: 165510 - 167223	7.1e-244
MeWRKY9-1	cassava4.1_032189m.g	1293	430	4	scaffold06914: 359445 - 361160	1.2e-286
MeWRKY9-2	cassava4.1_033113m.g	1530	509	5	scaffold05979: 103127 - 105387	0.0e+00
MeWRKY10	cassava4.1_026266m.g	375	124	2	scaffold06582: 784332 - 785524	7.5e-53
MeWRKY11-1	cassava4.1_011062m.g	1035	344	2	scaffold01046: 367570 - 369055	6.1e-226
MeWRKY11-2	cassava4.1_011056m.g	1035	344	2	scaffold06484: 9858 - 11444	1.0e-224
MeWRKY11-3	cassava4.1_011135m.g	1029	342	2	scaffold03874: 261993 - 263937	1.2e-227
MeWRKY13-1	cassava4.1_015466m.g	690	229	2	scaffold10954: 193689 - 196320	9.8e-125
MeWRKY13-2	cassava4.1_015440m.g	690	229	2	scaffold12231: 65733 - 68378	3.3e-117
MeWRKY14-1	cassava4.1_014297m.g	780	259	2	scaffold07571: 762773 - 763974	1.1e-175
MeWRKY14-2	cassava4.1_029757m.g	639	213	2	scaffold07301: 346062 - 347038	3.1e-118
MeWRKY20-1	cassava4.1_004372m.g	1740	579	5	scaffold03332: 324639 - 330792	0.0e+00
MeWRKY20-2	cassava4.1_004461m.g	1725	574	5	scaffold06278: 79089 - 84498	2.4e-309
MeWRKY21-1	cassava4.1_032017m.g	966	322	2	scaffold07035: 910534 - 911654	2.7e-232
MeWRKY21-2	cassava4.1_011033m.g	1038	345	2	scaffold02906: 214860 - 217706	1.1e-112
MeWRKY21-3	cassava4.1_028148m.g	906	302	2	scaffold11581: 195833 - 197023	7.8e-233
MeWRKY21-4	cassava4.1_011038m.g	1038	349	2	scaffold11998: 359241 - 361642	9.5e-122
MeWRKY22-1	cassava4.1_024248m.g	984	327	2	scaffold02658: 618480 - 619729	9.1e-221
MeWRKY22-2	cassava4.1_027012m.g	1029	342	2	scaffold03581: 1062737 - 1063959	9.5e-229
MeWRKY23-1	cassava4.1_012593m.g	915	304	2	scaffold07571: 656975 - 658228	2.8e-202
MeWRKY23-2	cassava4.1_012748m.g	903	300	2	scaffold00080: 65278 - 66511	3.2e-200
MeWRKY27-1	cassava4.1_008781m.g	1230	409	2	scaffold06089: 490434 - 491835	2.8e-280
MeWRKY27-2	cassava4.1_033531m.g	1377	458	2	scaffold00341: 351424 - 352981	1.3e-314
MeWRKY27-3	cassava4.1_020980m.g	939	312	2	scaffold05938: 86354 - 87511	4.1e-208
MeWRKY27-4	cassava4.1_028977m.g	639	212	2	scaffold07052: 1 - 903	2.3e-102
MeWRKY28	cassava4.1_029981m.g	1011	336	2	scaffold07520: 765376 - 767005	4.9e-231
MeWRKY32-1	cassava4.1_032932m.g	849	283	2	scaffold02022: 14374 - 15374	5.3e-140
MeWRKY32-2	cassava4.1_027662m.g	1146	381	2	scaffold00847: 2022942 - 2024601	2.4e-174
MeWRKY32-3	cassava4.1_008787m.g	1227	408	3	scaffold03049: 438605 - 440785	6.0e-271
MeWRKY33-1	cassava4.1_007752m.g	1329	442	4	scaffold06814: 508088 - 509823	9.3e-298
MeWRKY33-2	cassava4.1_004465m.g	1725	574	4	scaffold07519: 161519 - 163882	0.0e+00

MeWRKY33-3	cassava4.1_009059m.g	1200	399	2	scaffold03718: 21962 - 23602	5.1e-270
MeWRKY34	cassava4.1_002430m.g	2202	733	5	scaffold01441: 31631 - 36579	0.0e+00
MeWRKY35-1	cassava4.1_030132m.g	1326	441	2	scaffold07478: 706320 - 708761	2.0e-296
MeWRKY35-2	cassava4.1_027742m.g	1386	461	2	scaffold10322: 252574 - 255072	6.1e-307
MeWRKY39	cassava4.1_010602m.g	1071	356	2	scaffold03614: 2735461 - 2737434	1.2e-241
MeWRKY40-1	cassava4.1_033249m.g	888	295	3	scaffold00847: 1986892 - 1988243	1.1e-115
MeWRKY40-2	cassava4.1_024650m.g	900	299	4	scaffold03049: 373112 - 374338	4.8e-191
MeWRKY40-3	cassava4.1_012109m.g	951	316	3	scaffold02915: 262126 - 263656	6.6e-200
MeWRKY40-4	cassava4.1_011696m.g	987	328	3	scaffold05843: 146504 - 148035	1.0e-211
MeWRKY40-5	cassava4.1_014614m.g	756	251	3	scaffold00847: 1991461 - 1992835	5.5e-165
MeWRKY40-6	cassava4.1_014368m.g	774	257	3	scaffold03049: 380756 - 382555	9.1e-167
MeWRKY41-1	cassava4.1_032717m.g	1023	340	2	scaffold00341: 185431 - 187084	3.5e-230
MeWRKY41-2	cassava4.1_010398m.g	1086	361	2	scaffold06089: 251681 - 253588	7.8e-244
MeWRKY41-3	cassava4.1_011518m.g	1002	333	2	scaffold03175: 92714 - 94463	9.2e-227
MeWRKY41-4	cassava4.1_011106m.g	1032	343	2	scaffold09399: 194689 - 196277	1.7e-234
MeWRKY41-5	cassava4.1_010768m.g	1059	352	2	scaffold03581: 1192731 - 1195409	1.9e-231
MeWRKY41-6	cassava4.1_011089m.g	1032	343	2	scaffold02658: 712078 - 713983	9.9e-242
MeWRKY42-1	cassava4.1_023145m.g	1506	501	5	scaffold06278: 461756 - 464271	0.0e+00
MeWRKY42-2	cassava4.1_021542m.g	1686	561	6	scaffold03241: 68161 - 70997	0.0e+00
MeWRKY43-1	cassava4.1_022398m.g	516	171	1	scaffold03581: 1036456 - 1037257	2.8e-114
MeWRKY43-2	cassava4.1_033595m.g	564	187	1	scaffold02658: 610430 - 611373	1.3e-125
MeWRKY44	cassava4.1_007976m.g	1305	434	5	scaffold04651: 152250 - 155989	1.6e-295
MeWRKY47-1	cassava4.1_006003m.g	1515	504	5	scaffold00080: 388340 - 391378	0.0e+00
MeWRKY47-2	cassava4.1_021176m.g	1518	505	5	scaffold07571: 322902 - 325531	0.0e+00
MeWRKY48-1	cassava4.1_011894m.g	969	322	2	scaffold01551: 3414603 - 3416232	1.2e-213
MeWRKY48-2	cassava4.1_011940m.g	966	321	2	scaffold00708: 367528 - 369055	2.1e-164
MeWRKY49	cassava4.1_032605m.g	858	285	2	scaffold05873: 10175 - 12560	1.2e-190
MeWRKY50-1	cassava4.1_032004m.g	450	149	2	scaffold00206: 486601 - 487231	1.3e-97
MeWRKY50-2	cassava4.1_031483m.g	492	163	2	scaffold07859: 539800 - 540488	2.3e-110
MeWRKY51	cassava4.1_016594m.g	606	201	2	scaffold10954: 23861 - 25493	1.1e-135
MeWRKY55-1	cassava4.1_023169m.g	948	315	2	scaffold03454: 260885 - 262793	1.1e-209
MeWRKY55-2	cassava4.1_027942m.g	939	312	2	scaffold10729: 113727 - 115432	7.9e-209
MeWRKY56	cassava4.1_022257m.g	522	174	1	scaffold06935: 1365 - 1987	3.3e-74

MeWRKY57-1	cassava4.1_026566m.g	912	303	2	scaffold12373: 44814 - 47819	1.6e-208
MeWRKY57-2	cassava4.1_012575m.g	915	304	2	scaffold00109: 89941 - 93589	1.2e-205
MeWRKY59	cassava4.1_008611m.g	1245	414	3	scaffold06711: 258341 - 262733	2.9e-277
MeWRKY65-1	cassava4.1_014035m.g	798	265	2	scaffold04003: 29864 - 31436	2.1e-181
MeWRKY65-2	cassava4.1_032956m.g	792	263	2	scaffold00834: 106694 - 107828	5.8e-148
MeWRKY70-1	cassava4.1_011680m.g	987	328	2	scaffold09360: 273486 - 275435	2.3e-225
MeWRKY70-2	cassava4.1_033846m.g	822	274	2	scaffold03614: 552094 - 553501	4.2e-106
MeWRKY70-3	cassava4.1_012154m.g	948	315	2	scaffold10729: 109107 - 110558	5.4e-216
MeWRKY70-4	cassava4.1_013417m.g	849	282	2	scaffold03454: 258099 - 259608	2.0e-189
MeWRKY70-5	cassava4.1_014812m.g	741	246	1	scaffold11287: 22154 - 23391	2.7e-166
MeWRKY71-1	cassava4.1_011936m.g	966	321	2	scaffold09120: 62859 - 64289	2.3e-219
MeWRKY71-2	cassava4.1_012482m.g	924	307	2	scaffold06582: 166435 - 167738	4.9e-210
MeWRKY71-3	cassava4.1_015949m.g	651	216	1	scaffold09702: 339821 - 340893	2.6e-53
MeWRKY72-1	cassava4.1_004929m.g	1647	548	3	scaffold08359: 2218663 - 2220806	0.0e+00
MeWRKY72-2	cassava4.1_032698m.g	1662	553	3	scaffold12794: 1580383 - 1582481	7.3e-227
MeWRKY72-3	cassava4.1_026701m.g	1677	558	3	scaffold11495: 423070 - 425756	0.0e+00
MeWRKY72-4	cassava4.1_026182m.g	1587	528	3	scaffold09180: 368608 - 371775	0.0e+00
MeWRKY72-5	cassava4.1_027917m.g	999	332	4	scaffold06050: 105342 - 107529	7.0e-222
MeWRKY75-1	cassava4.1_028994m.g	573	190	1	scaffold06711: 428863 - 430192	2.4e-125
MeWRKY75-2	cassava4.1_023502m.g	582	193	1	scaffold03219: 357170 - 358201	1.3e-127
MeWRKY75-3	cassava4.1_017208m.g	552	183	1	scaffold06028: 403119 - 404529	2.4e-122
MeWRKY75-4	cassava4.1_017214m.g	552	183	1	scaffold00325: 479801 - 480856	1.3e-122
MeWRKY75-5	cassava4.1_017377m.g	540	179	1	scaffold01701: 195921 - 196832	1.3e-117
Medicago truncatula						
MtWRKY1-1	Medtr5g097490	1176	391	3	chr5: 41665699 - 41667186	9.9e-261
MtWRKY1-2	Medtr5g097500	936	311	1	chr5: 41673897 - 41674989	1.8e-91
MtWRKY3-1	Medtr5g042920	1302	433	3	chr5: 18458993 - 18462875	1.0e-293
MtWRKY3-2	Medtr7g011820	1566	521	3	chr7: 2738150 - 2745216	0.0e+00
MtWRKY4-1	Medtr4g082580	1167	388	3	chr4: 28042631 - 28045908	4.4e-267
MtWRKY4-2	Medtr6g046600	357	118	0	chr6: 9798476 - 9798832	4.6e-76
MtWRKY5	AC202489_18	1548	515	3	AC202489.12: 64789 - 70103	0.0e+00
MtWRKY6-1	Medtr2g088000	1809	602	4	chr2: 27320043 - 27323462	0.0e+00
MtWRKY6-2	Medtr4g047580	1761	586	4	chr4: 13808222 - 13812118	0.0e+00

MtWRKY6	Medtr1g013790	768	255	3	chr1: 3532717 - 3533977	3.3e-166
MtWRKY11-1	Medtr5g018380	537	178	2	chr5: 6591918 - 6593332	2.8e-113
MtWRKY11-2	Medtr3g108960	1212	403	2	chr3: 39033440 - 39035025	2.5e-269
MtWRKY11-3	Medtr4g127040	954	317	2	chr4: 44094293 - 44095449	3.4e-207
MtWRKY12	Medtr2g045360	1266	421	5	chr2: 16225588 - 16229077	9.5e-274
MtWRKY13-1	Medtr3g095080	735	244	2	chr3: 32628073 - 32630168	1.2e-168
MtWRKY13-2	Medtr8g092140	648	215	2	chr8: 26076085 - 26077862	2.7e-145
MtWRKY15	Medtr5g016610	816	271	2	chr5: 5721691 - 5723310	3.8e-178
MtWRKY17	Medtr1g009380	1023	340	2	chr1: 1920992 - 1923320	3.4e-225
MtWRKY19	Medtr7g104980	648	215	1	chr7: 33725547 - 33726355	2.4e-140
MtWRKY20-1	Medtr5g094430	1638	545	5	chr5: 40230167 - 40234129	0.0e+00
MtWRKY20-2	Medtr3g056100	1788	595	5	chr3: 17614774 - 17621276	0.0e+00
MtWRKY22-1	Medtr7g079040	987	328	2	chr7: 22190788 - 22192273	1.0e-217
MtWRKY22-2	Medtr7g038380	1017	338	2	chr7: 11103701 - 11105381	2.0e-223
MtWRKY22-3	Medtr8g067750	2103	700	3	chr8: 17346636 - 17350627	0.0e+00
MtWRKY22-4	Medtr7g105000	1077	358	2	chr7: 33730293 - 33731583	1.2e-236
MtWRKY22-5	Medtr8g067650	1563	520	2	chr8: 17302535 - 17305136	0.0e+00
MtWRKY22-6	Medtr8g067350	1317	438	2	chr8: 17145617 - 17147973	1.1e-252
MtWRKY23-1	Medtr7g109800	885	294	2	chr7: 34922486 - 34923943	2.6e-194
MtWRKY23-2	Medtr1g086790	978	325	2	chr1: 23201879 - 23203731	1.1e-215
MtWRKY23-3	Medtr4g122530	1251	416	2	chr4: 42405829 - 42407632	5.4e-277
MtWRKY26	Medtr5g074400	1746	581	4	chr5: 30613078 - 30616836	0.0e+00
MtWRKY27	Medtr1g099600	939	312	2	chr1: 28931060 - 28932347	1.3e-208
MtWRKY32	Medtr4g130900	1494	497	4	chr4: 46014841 - 46020092	0.0e+00
MtWRKY33-1	Medtr5g074200	984	327	0	chr5: 30495461 - 30496444	8.6e-215
MtWRKY33-2	Medtr3g036720	984	327	0	chr3: 12143365 - 12144407	8.6e-215
MtWRKY33-3	Medtr5g093540	750	249	3	chr5: 39805482 - 39806424	1.7e-162
MtWRKY33-4	Medtr3g031220	1527	508	4	chr3: 9496559 - 9500669	0.0e+00
MtWRKY33-5	Medtr7g071120	1524	507	4	chr7: 18554374 - 18557213	0.0e+00
MtWRKY35	Medtr7g109600	798	265	2	chr7: 34798691 - 34800638	2.9e-170
MtWRKY38-1	Medtr2g075750	567	188	2	chr2: 23170272 - 23171511	1.5e-98
MtWRKY38-2	Medtr2g075610	570	189	2	chr2: 23121051 - 23122626	1.9e-124
MtWRKY38-3	Medtr2g075700	933	310	2	chr2: 23151232 - 23155746	5.9e-209

MtWRKY38-4	Medtr2g075680	921	306	2	chr2: 23141005 - 23143137	2.3e-207
MtWRKY38-5	Medtr2g075690	903	300	2	chr2: 23148025 - 23150151	2.4e-203
MtWRKY40-1	Medtr4g007060	954	317	4	chr4: 1057359 - 1059599	7.3e-205
MtWRKY40-2	Medtr3g106060	885	294	3	chr3: 37454723 - 37458634	1.6e-192
MtWRKY40-3	Medtr1g013760	858	285	3	chr1: 3519314 - 3521073	2.0e-186
MtWRKY41-1	Medtr7g117200	987	328	2	chr7: 38173531 - 38175316	1.9e-220
MtWRKY41-2	Medtr3g090860	1023	340	2	chr3: 30788232 - 30789490	1.1e-230
MtWRKY42-1	Medtr6g045670	1479	492	5	chr6: 9154734 - 9156698	0.0e+00
MtWRKY42-2	Medtr4g132430	1479	492	5	chr4: 46819313 - 46821277	0.0e+00
MtWRKY42-3	Medtr5g044700	816	271	6	chr5: 19196079 - 19199060	2.7e-95
MtWRKY43	Medtr7g079010	660	219	1	chr7: 22168220 - 22171006	4.3e-148
MtWRKY44-1	Medtr7g100510	1317	438	6	chr7: 31666229 - 31670642	2.5e-295
MtWRKY44-2	Medtr7g080000	2115	704	4	chr7: 22639699 - 22645235	0.0e+00
MtWRKY44-3	Medtr7g080020	2148	805	3	chr7: 22653293 - 22657549	0.0e+00
MtWRKY47	Medtr7g110720	1302	433	5	chr7: 35383726 - 35386918	2.4e-289
MtWRKY48-1	Medtr2g033820	1071	356	2	chr2: 11689454 - 11691140	1.9e-236
MtWRKY48-2	Medtr5g029850	348	115	2	chr5: 12309260 - 12310880	2.4e-34
MtWRKY49	Medtr3g095040	474	157	2	chr3: 32600007 - 32600956	4.2e-89
MtWRKY50-1	Medtr3g104750	495	164	2	chr3: 36821149 - 36822012	5.4e-110
MtWRKY50-2	Medtr1g015140	489	162	2	chr1: 4119896 - 4120611	1.2e-107
MtWRKY51-1	Medtr3g095030	273	90	1	chr3: 32599270 - 32599654	2.8e-39
MtWRKY51-2	Medtr8g092010	555	184	2	chr8: 25980319 - 25982171	2.1e-83
MtWRKY53	AC233680_9	651	217	2	AC233680.1: 33048 - 35491	2.0e-139
MtWRKY55	Medtr5g067680	1074	357	2	chr5: 27618450 - 27622967	1.2e-244
MtWRKY57	Medtr5g043880	1098	365	4	chr5: 18854021 - 18861097	4.6e-178
MtWRKY61	Medtr4g107970	1842	613	4	chr4: 37140579 - 37144825	0.0e+00
MtWRKY65	Medtr8g087000	735	244	2	chr8: 24099398 - 24100512	3.1e-164
MtWRKY69	Medtr2g083870	786	261	2	chr2: 25558124 - 25562407	1.4e-177
MtWRKY70-1	Medtr7g073380	885	294	2	chr7: 19618880 - 19620570	2.2e-201
MtWRKY70-2	Medtr7g073430	852	283	2	chr7: 19635883 - 19638509	1.9e-195
MtWRKY70-3	Medtr8g005750	840	279	2	chr8: 438924 - 440198	9.2e-188
MtWRKY70	Medtr5g067700	711	236	2	chr5: 27630061 - 27634595	4.5e-158
MtWRKY71	Medtr5g091390	933	310	2	chr5: 38795236 - 38797564	3.0e-204

MtWRKY72	Medtr7g009730	1746	581	3	chr7: 1692122 - 1695833	0.0e+00
Micromonas pusilla						
MpWRKY4	EuGene.1200010296	1242	413	1	Chr_12: 451782 - 453417	6.7e-45
MpWRKY45	EuGene.0900010102	816	271	0	Chr_09: 217114 - 217929	1.2e-179
Mimulus guttatus						
MgWRKY2	mgv1a002460m.g	2016	671	4	scaffold_4: 1069186 - 1071980	0.0e+00
MgWRKY3	mgv1a006365m.g	1344	447	4	scaffold_66: 70065 - 72996	5.3e-305
MgWRKY4-1	mgv11b004965m.g	1380	459	3	scaffold_22: 339090 - 341822	0.0e+00
MgWRKY4-2	mgv1a021306m.g	1614	537	3	scaffold_11: 79091 - 81668	0.0e+00
MgWRKY4-3	mgv1a004979m.g	1509	502	3	scaffold_37: 847626 - 850286	0.0e+00
MgWRKY5	mgv1a026438m.g	747	248	3	scaffold_151: 497534 - 499999	1.6e-173
MgWRKY6-1	mgv1a003939m.g	1662	553	4	scaffold_4: 2203235 - 2205593	0.0e+00
MgWRKY6-2	mgv1a008427m.g	1125	374	2	scaffold_24: 1831329 - 1833050	5.5e-250
MgWRKY6-3	mgv1a004322m.g	1602	533	4	scaffold_235: 237247 - 239831	0.0e+00
MgWRKY6-4	mgv1a007828m.g	1185	394	5	scaffold_4: 335912 - 338457	1.0e-261
MgWRKY7-1	mgv1a009584m.g	1014	337	2	scaffold_5: 1038773 - 1040888	1.6e-223
MgWRKY7-2	mgv1a009272m.g	1044	347	2	scaffold_2: 2166770 - 2168346	1.9e-230
MgWRKY11	mgv1a009891m.g	987	328	2	scaffold_69: 1074670 - 1076442	5.6e-218
MgWRKY12	mgv1a009879m.g	987	328	4	scaffold_54: 840248 – 842157	4.1e-205
MgWRKY14	mgv1a006007m.g	1386	461	3	scaffold_83: 342250 - 344625	3.9e-315
MgWRKY15	mgv1a009353m.g	1035	344	2	scaffold_11: 1407100 - 1408532	1.2e-219
MgWRKY18	mgv1a025068m.g	768	255	2	scaffold_36: 921976 - 923228	4.8e-169
MgWRKY19	mgv1a019464m.g	1026	341	1	scaffold_207: 197618 - 199136	1.6e-148
MgWRKY20	mgv1a003822m.g	1689	562	5	scaffold_4: 604438 - 607772	0.0e+00
MgWRKY21-1	mgv1a021173m.g	357	118	2	scaffold_1: 3113096 - 3113687	7.1e-76
MgWRKY21-2	mgv1a008853m.g	1083	360	2	scaffold_92: 142692 - 144552	5.1e-244
MgWRKY22-1	mgv1a009333m.g	1038	345	2	scaffold_27: 1432873 - 1434252	2.6e-233
MgWRKY22-2	mgv1a009324m.g	1038	345	2	scaffold_211: 373046 - 375187	4.7e-233
MgWRKY23	mgv1a010400m.g	942	313	2	scaffold_1: 4629525 - 4630949	8.9e-211
MgWRKY26-1	mgv1a004369m.g	1596	531	4	scaffold_13: 2228743 - 2231285	0.0e+00
MgWRKY26-2	mgv1a004351m.g	1596	531	4	scaffold_1: 820220 - 822528	0.0e+00
MgWRKY27	mgv1a023144m.g	1158	385	2	scaffold_51: 1032810 - 1034148	1.1e-259
MgWRKY29	mgv1a024960m.g	1005	334	2	scaffold_60: 973453 - 974789	5.8e-228

MgWRKY30	mgv1a019824m.g	492	163	2	scaffold_50: 841141 - 841923	1.0e-103
MgWRKY32-1	mgv1a009827m.g	993	330	3	scaffold_209: 70744 - 72608	1.8e-227
MgWRKY32-2	mgv1a004242m.g	1617	538	4	scaffold_228: 345762 - 349615	0.0e+00
MgWRKY34-1	mgv1a018837m.g	1488	496	6	scaffold_16: 38873 - 41198	0.0e+00
MgWRKY34-2	mgv1a019661m.g	1701	566	5	scaffold_24: 1182309 - 1185523	0.0e+00
MgWRKY35-1	mgv1a017713m.g	1281	426	2	scaffold_12: 1781651 - 1783461	4.5e-291
MgWRKY35-2	mgv1a012761m.g	726	241	2	scaffold_1: 4567726 - 4568965	5.7e-162
MgWRKY36	mgv1a026752m.g	1428	476	2	scaffold_37: 277160 - 278787	1.5e-314
MgWRKY40	mgv1a010320m.g	951	316	3	scaffold_7: 2928201 - 2929833	7.3e-204
MgWRKY41	mgv1a009487m.g	1023	340	2	scaffold_158: 114439 - 115917	2.5e-234
MgWRKY43	mgv1a020274m.g	579	192	1	scaffold_60: 963815 - 964718	5.1e-129
MgWRKY46-1	mgv1a008753m.g	1092	363	2	scaffold_6: 1516945 - 1521190	1.9e-238
MgWRKY46-2	mgv1a025234m.g	897	298	2	scaffold_6: 1565196 - 1567928	4.9e-202
MgWRKY48-1	mgv1a020914m.g	1047	348	2	scaffold_102: 149194 - 150830	2.8e-237
MgWRKY48-2	mgv1a023570m.g	600	199	1	scaffold_335: 147749 - 148436	7.4e-132
MgWRKY49	mgv1a014828m.g	534	177	1	scaffold_13: 1208143 - 1209097	1.2e-116
MgWRKY50	mgv1a014674m.g	549	182	3	scaffold_122: 157885 - 159258	1.2e-120
MgWRKY51-1	mgv1a022461m.g	516	171	3	scaffold_36: 1679897 - 1680930	1.3e-112
MgWRKY51-2	mgv1a014924m.g	525	174	2	scaffold_79: 448317 - 449226	8.8e-116
MgWRKY53-1	mgv1a010015m.g	975	324	2	scaffold_27: 1649630 - 1651800	2.3e-220
MgWRKY53-2	mgv1a020603m.g	603	201	2	scaffold_168: 433994 - 435409	7.2e-132
MgWRKY53-3	mgv1a019981m.g	1071	356	2	scaffold_51: 1206299 - 1208096	1.1e-243
MgWRKY53-4	mgv1a021631m.g	759	253	2	scaffold_60: 707384 - 708336	4.1e-176
MgWRKY55	mgv1a024310m.g	1098	365	2	scaffold_8: 3166914 - 3169937	1.9e-249
MgWRKY57-1	mgv1a009929m.g	984	327	3	scaffold_66: 28110 - 30702	1.3e-219
MgWRKY57-2	mgv1a011170m.g	873	290	2	scaffold_37: 986084 - 988147	3.9e-195
MgWRKY60	mgv1a013791m.g	633	210	3	scaffold_69: 534705 - 535855	7.9e-141
MgWRKY65	mgv1a014940m.g	639	212	1	scaffold_13: 16480142 - 16480899	8.6e-121
MgWRKY69	mgv1a012827m.g	720	239	3	scaffold_186: 211229 - 214310	3.0e-100
MgWRKY70-1	mgv1a010128m.g	966	321	2	scaffold_8: 3172300 - 3174663	9.4e-219
MgWRKY70-2	mgv1a008874m.g	1083	360	2	scaffold_13: 2474424 - 2476158	6.0e-247
MgWRKY71-1	mgv1a023072m.g	918	305	2	scaffold_4: 4037622 - 4038734	1.1e-204
MgWRKY71-2	mgv1a022795m.g	1053	350	2	scaffold_12: 2215926 - 2217540	2.3e-234

MgWRKY71-3	mgv1a022066m.g	996	331	2	scaffold_34: 1655529 - 1657135	9.1e-226
MgWRKY72-1	mgv1a021188m.g	1698	565	5	scaffold_20: 1887952 - 1891371	1.0e-259
MgWRKY72-2	mgv1a025718m.g	1398	465	2	scaffold_233: 216049 - 217695	3.3e-315
MgWRKY75	mgv1a021286m.g	387	128	1	scaffold_104: 931637 - 932636	8.8e-84
			Oryza sativa			
OsWRKY1	LOC_Os01g14440	1752	583	4	Chr1: 8084372 - 8087044	0.0e+00
OsWRKY2	LOC_Os10g42850	900	299	1	Chr10: 23107503 - 23108646	2.0e-204
OsWRKY3	LOC_Os03g55080	945	314	2	Chr3: 31323190 - 31326926	3.7e-204
OsWRKY4	LOC_Os03g55164	3009	1002	11	Chr3: 31391390 - 31399050	0.0e+00
OsWRKY5	LOC_Os05g04640	1509	502	5	Chr5: 2179520 - 2184940	0.0e+00
OsWRKY6	LOC_Os03g58420	1143	380	2	Chr3: 33286932 - 33288483	9.0e-245
OsWRKY7	LOC_Os05g46020	666	221	2	Chr5: 26682472 - 26684208	1.9e-141
OsWRKY8	LOC_Os05g50610	1512	503	6	Chr5: 29009136 - 29013100	0.0e+00
OsWRKY9	LOC_Os01g18584	1812	603	5	Chr1: 10470540 - 10478800	0.0e+00
OsWRKY10	LOC_Os01g09100	660	219	0	Chr1: 4572448 - 4573409	1.9e-146
OsWRKY11	LOC_Os01g43650	1140	379	3	Chr1: 25009453 - 25012236	8.4e-258
OsWRKY12	LOC_Os01g43550	1053	350	2	Chr1: 24945286 - 24947296	6.7e-230
OsWRKY13	LOC_Os01g54600	951	316	2	Chr1: 31409004 - 31410978	6.6e-207
OsWRKY14	LOC_Os01g53040	960	319	1	Chr1: 30480134 - 30482161	1.9e-210
OsWRKY15	LOC_Os01g46800	825	274	2	Chr1: 26687377 - 26688416	1.2e-181
OsWRKY16	LOC_Os01g47560	1743	580	4	Chr1: 27196417 - 27200990	0.0e+00
OsWRKY17	LOC_Os01g74140	1233	410	2	Chr1: 42946753 - 42948750	2.6e-279
OsWRKY18	LOC_Os01g60520	852	283	2	Chr1: 34996792 - 35003226	1.1e-194
OsWRKY19	LOC_Os05g49620	834	277	2	Chr5: 28471802 - 28473061	2.6e-183
OsWRKY20	LOC_Os01g60540	1128	375	2	Chr1: 35008866 - 35011098	3.0e-249
OsWRKY21	LOC_Os01g60640	843	280	1	Chr1: 35062734 - 35064940	3.6e-182
OsWRKY22	LOC_Os01g60490	798	265	2	Chr1: 34981468 - 34985447	2.5e-182
OsWRKY23	LOC_Os01g53260	765	254	1	Chr1: 30604295 - 30608077	8.0e-173
OsWRKY24	LOC_Os01g61080	1668	555	4	Chr1: 35347978 - 35350645	0.0e+00
OsWRKY25	LOC_Os08g13840	924	307	0	Chr8: 8258575 - 8259595	4.7e-199
OsWRKY26	LOC_Os01g51690	738	245	2	Chr1: 29720923 - 29723065	9.2e-163
OsWRKY27	LOC_Os01g40430	933	310	2	Chr1: 22824809 - 22827116	6.6e-208
OsWRKY28	LOC_Os06g44010	1191	396	2	Chr6: 26509919 - 26511699	6.3e-257

OsWRKY29	LOC_Os07g02060	873	290	2	Chr7: 630562 - 634316	7.4e-192
OsWRKY30	LOC_Os08g38990	2025	674	4	Chr8: 24645860 - 24649085	0.0e+00
OsWRKY31	LOC_Os06g30860	1041	346	1	Chr6: 17915923 - 17917083	1.8e-236
OsWRKY32	LOC_Os02g53100	1815	604	5	Chr2: 32489017 - 32495070	0.0e+00
OsWRKY34	LOC_Os02g43560	570	189	2	Chr2: 26280253 - 26283914	1.9e-127
OsWRKY35	LOC_Os04g39570	2997	998	9	Chr4: 23579869 - 23587188	0.0e+00
OsWRKY36	LOC_Os04g46060	753	250	3	Chr4: 27284275 - 27290983	3.6e-175
OsWRKY37	LOC_Os04g50920	1545	514	3	Chr4: 30132491 - 30136547	0.0e+00
OsWRKY39	LOC_Os02g16540	1086	361	2	Chr2: 9446492 - 9448847	9.0e-240
OsWRKY40	LOC_Os11g02530	963	320	2	Chr11: 787542 - 789030	4.5e-217
OsWRKY41	LOC_Os11g45920	1203	400	4	Chr11: 27785982 - 27779324	1.6e-271
OsWRKY42	LOC_Os02g26430	762	253	2	Chr2: 15521497 - 15522724	9.1e-166
OsWRKY43	LOC_Os05g49210	1878	625	5	Chr5: 28238562 - 28241041	0.0e+00
OsWRKY44	LOC_Os03g21710	1071	356	2	Chr3: 12394669 - 12396898	2.2e-231
OsWRKY45	LOC_Os05g25770	981	326	2	Chr5: 14991579 - 14993800	2.7e-216
OsWRKY46-1	LOC_Os11g02480	675	224	2	Chr11: 759587 - 763334	9.8e-149
OsWRKY46-2	LOC_Os12g02420	675	224	2	Chr12: 802489 - 806097	8.9e-151
OsWRKY47	LOC_Os07g48260	1002	333	3	Chr7: 28828793 - 28832398	1.8e-219
OsWRKY48	LOC_Os05g40060	996	331	1	Chr5: 23529423 - 23530499	1.9e-217
OsWRKY49	LOC_Os05g49100	1260	419	2	Chr5: 28154693 - 28157989	2.2e-285
OsWRKY50	LOC_Os11g02540	993	330	2	Chr11: 795608 - 793116	6.0e-224
OsWRKY51	LOC_Os04g21950	981	326	2	Chr4: 12425564 - 12427457	1.6e-215
OsWRKY52	LOC_Os11g02470	1008	335	2	Chr11: 749998 - 751210	1.0e-227
OsWRKY53	LOC_Os05g27730	1464	487	4	Chr5: 16150266 - 16152747	0.0e+00
OsWRKY54	LOC_Os05g40080	987	328	1	Chr5: 23550611 - 23551716	1.1e-218
OsWRKY55	LOC_Os03g20550	633	210	2	Chr3: 11650824 - 11652144	3.4e-144
OsWRKY56	LOC_Os12g02440	795	264	5	Chr12: 817003 - 823002	6.3e-181
OsWRKY57	LOC_Os12g01180	1371	456	2	Chr12: 100799 - 104008	0.0e+00
OsWRKY58	LOC_Os05g45230	546	181	1	Chr5: 26256951 - 26257809	3.2e-113
OsWRKY59	LOC_Os11g45750	3609	1201	1	Chr11: 27690288 - 27683640	0.0e+00
OsWRKY60	LOC_Os03g45450	447	148	2	Chr3: 25651039 - 25652125	4.2e-98
OsWRKY61	LOC_Os11g45850	780	259	2	Chr11: 27740142 - 27741375	8.5e-180
OsWRKY62	LOC_Os09g25070	957	318	3	Chr9: 14992803 - 14994888	7.6e-204

OsWRKY64	LOC_Os12g02450	966	321	3	Chr12: 824302 - 825793	1.4e-216
OsWRKY65	LOC_Os12g02470	1025	342	2	Chr12: 838755 - 836516	2.6e-230
OsWRKY66	LOC_Os02g47060	1521	506	2	Chr2: 28726783 - 28730933	0.0e+00
OsWRKY67	LOC_Os05g09020	585	194	2	Chr5: 4998210 - 4999626	4.8e-127
OsWRKY68	LOC_Os04g51560	930	309	2	Chr4: 30545175 - 30546577	2.3e-200
OsWRKY69	LOC_Os08g29660	960	319	1	Chr8: 18220041 - 18222408	2.3e-214
OsWRKY70	LOC_Os05g39720	1719	572	4	Chr5: 23310474 - 23313449	0.0e+00
OsWRKY71	LOC_Os02g08440	1047	348	3	Chr2: 4542762 - 4544983	2.6e-231
OsWRKY72	LOC_Os11g29870	729	242	1	Chr11: 17352085 - 17355820	7.0e-159
OsWRKY73	LOC_Os06g05380	1584	527	2	Chr6: 2429149 - 2433254	0.0e+00
OsWRKY74	LOC_Os09g16510	1086	361	2	Chr9: 10128825 - 10131136	2.3e-240
OsWRKY75	LOC_Os05g25700	Not found				
OsWRKY76	LOC_Os09g25060	984	327	1	Chr9: 14975932 - 14977713	4.1e-219
OsWRKY77	LOC_Os01g40260	741	246	2	Chr1: 22731943 - 22733240	1.6e-163
OsWRKY78	LOC_Os07g39480	1857	618	5	Chr7: 23654076 - 23659625	0.0e+00
OsWRKY79	LOC_Os03g21710	1071	356	2	Chr3: 12394669 - 12396898	2.2e-231
OsWRKY80	LOC_Os09g30400	1902	633	5	Chr9: 18496949 - 18500579	0.0e+00
OsWRKY81	LOC_Os12g02400	1014	337	2	Chr12: 789403 - 790687	4.1e-231
OsWRKY82	LOC_Os08g17400	1653	550	3	Chr8: 10633195 - 10639603	0.0e+00
OsWRKY83	LOC_Os12g40570	1098	365	4	Chr12: 25100479 - 25104175	6.8e-244
OsWRKY84	LOC_Os03g33020	1434	478	3	Chr3:18882373 - 18893375	2.7e-254
OsWRKY85	LOC_Os12g32250	1623	540	5	Chr12: 19473728 - 19478606	0.0e+00
OsWRKY86	LOC_Os01g60600	1077	358	2	Chr1: 35032511 - 35035321	1.3e-237
OsWRKY87	LOC_Os03g53050	1164	387	3	Chr3: 30422191 - 30425543	1.6e-259
OsWRKY88	LOC_Os03g63810	1164	384	2	Chr3: 36039164 - 36043822	3.7e-264
OsWRKY89	LOC_Os05g03900	741	246	3	Chr5: 1743366 - 1745281	1.3e-164
OsWRKY90	LOC_Os05g14370	927	308	2	Chr5: 8086892 - 8088575	4.5e-211
OsWRKY91	LOC_Os05g40070	843	280	2	Chr5: 23536113 – 23539013	1.6e-186
OsWRKY92	LOC_Os05g50700	990	329	2	Chr5: 29064582 - 29065815	2.2e-223
OsWRKY93	LOC_Os06g06360	1176	391	2	Chr6: 2958991 - 2963006	2.6e-267
OsWRKY94	LOC_Os07g17230	4074	1357	1	Chr7: 10155452 - 10159775	0.0e+00
OsWRKY95	LOC_Os07g27670	783	260	2	Chr7: 16147913 - 16149483	4.8e-178
OsWRKY96	LOC_Os07g40570	1299	432	3	Chr7: 24311898 - 24315383	5.5e-298

OsWRKY97	LOC_Os01g09080	1524	507	4	Chr1: 4566304 - 4568617	0.0e+00
OsWRKY98	LOC_Os09g09630	876	291	2	Chr9: 5187963 - 5190200	3.3e-201
OsWRKY100	LOC_Os11g02520	843	280	2	Chr11: 782358 - 783511	4.9e-194
OsWRKY101	LOC_Os10g18099	831	276	2	Chr10: 9184625 - 9192018	5.0e-190
OsWRKY102	LOC_Os01g08710	1275	424	7	Chr1: 4340849 - 4356383	4.7e-287
OsWRKY103	LOC_Os01g62514	588	195	1	Chr1: 36193840 - 36194611	1.5e-130
OsWRKY104	LOC_Os11g02520	843	280	2	Chr11: 782358 - 783511	4.9e-194
OsWRKY105	LOC_Os03g33012	1434	477	3	Chr3: 18882373 - 18893375	2.7e-245
OsWRKY106	LOC_Os01g62510	612	203	1	Chr1: 36188702 - 36191681	1.0e-134
Ostreococcus lucimarinus						
OlWRKY4	eugene.1100010253	735	244	1	Chr_11: 473735 - 474522	5.4e-155
OlWRKY51	ost_08_010_039	1047	348	0	Chr_8: 362741 - 363787	3.3e-231
Panicum hallii						
PhWRKY1	Pahal.0474s0006	1491	496	3	scaffold_474: 37316 - 40177	1.3e-156
PhWRKY2	Pahal.0196s0053	1275	424	2	scaffold_196: 500807 - 502198	1.0e-191
PhWRKY3	Pahal.0139s0013	963	320	3	scaffold_139: 52649 - 56695	2.2e-159
PhWRKY4	Pahal.0139s0024	2451	816	10	scaffold_139: 121641 - 127290	0.0e+00
PhWRKY5	Pahal.0632s0010	1590	529	5	scaffold_632: 39054 - 43367	1.3e-177
PhWRKY6	Pahal.0024s0166	1194	397	2	scaffold_24: 932212 - 934483	5.1e-221
PhWRKY7	Pahal.0217s0007	666	221	2	scaffold_217: 31806 - 33464	5.3e-113
PhWRKY8	Pahal.0019s0134	1011	336	1	scaffold_19: 1183837 - 1186270	5.5e-159
PhWRKY9	Pahal.0018s0331	1716	571	5	scaffold_18: 2364616 - 2369464	5.1e-305
PhWRKY10	Pahal.0003s0370	711	236	2	scaffold_3: 2862259 - 2863688	2.0e-100
PhWRKY11	Pahal.0048s0075	1089	362	2	scaffold_48: 562376 - 565496	1.9e-186
PhWRKY13	Pahal.0083s0190	900	299	2	scaffold_83: 1167824 - 1169714	7.4e-166
PhWRKY14	Pahal.0083s0050	918	305	1	scaffold_83: 333065 - 334755	1.9e-172
PhWRKY15	Pahal.0022s0158	975	324	2	scaffold_22: 2108229 - 2110450	1.3e-154
PhWRKY16	Pahal.0109s0057	1194	397	2	scaffold_109: 575915 - 578958	1.1e-168
PhWRKY17	Pahal.0043s0324	1083	360	2	scaffold_43: 1915052 - 1917228	2.8e-92
PhWRKY18	Pahal.0006s0269	780	259	2	scaffold_6: 1586880 - 1590839	6.8e-150
PhWRKY19	Pahal.0019s0243	864	287	2	scaffold_19: 1793832 - 1795328	4.4e-124
PhWRKY20	Pahal.0006s0268	1056	351	2	scaffold_6: 1582885 - 1585626	2.1e-131
PhWRKY21	Pahal.0006s0265	846	281	1	scaffold_6: 1568159 - 1569821	1.7e-107

PhWRKY22	Pahal.0006s0270	735	244	2	scaffold_6: 1595459 - 1599053	2.5e-135
PhWRKY23-1	Pahal.0083s0066	759	252	1	scaffold_83: 423913 - 426876	5.7e-114
PhWRKY23-2	Pahal.0078s0165	651	216	1	scaffold_78: 1217115 - 1218197	1.9e-92
PhWRKY24	Pahal.0006s0232	1659	552	4	scaffold_6: 1365755 - 1368551	0.0e+00
PhWRKY25	Pahal.0551s0001	663	220	1	scaffold_551: 22670 - 24109	1.8e-94
PhWRKY26	Pahal.0306s0023	663	220	2	scaffold_306: 180238 - 181516	4.2e-98
PhWRKY27	Pahal.0026s0109	981	326	1	scaffold_26: 1421035 - 1422589	4.8e-144
PhWRKY28	Pahal.0001s0452	993	330	2	scaffold_1: 4991854 - 4993722	6.3e-175
PhWRKY29	Pahal.0175s0048	1071	356	2	scaffold_175: 357094 - 360467	1.6e-118
PhWRKY30	Pahal.0010s0278	2064	687	5	scaffold_10: 1751601 - 1756098	0.0e+00
PhWRKY31-1	Pahal.0151s0007	1203	400	1	scaffold_151: 103717 - 105251	1.4e-158
PhWRKY31-2	Pahal.0955s0002	957	318	1	scaffold_955: 18388 - 19580	2.6e-51
PhWRKY32	Pahal.0062s0040	1677	558	4	scaffold_62: 257298 - 259819	1.6e-308
PhWRKY34	Pahal.0015s0239	684	227	3	scaffold_15: 1652148 - 1655345	4.4e-145
PhWRKY35	Pahal.0126s0160	2115	704	6	scaffold_126: 891940 - 897553	0.0e+00
PhWRKY36	Pahal.0349s0045	693	230	3	scaffold_349: 422953 - 426749	2.6e-140
PhWRKY37	Pahal.0094s0096	1428	475	2	scaffold_94: 537788 - 541309	3.6e-288
PhWRKY39-1	Pahal.0055s0106	1134	377	2	scaffold_55: 1000426 - 1001785	2.9e-148
PhWRKY39-2	Pahal.0180s0035	1134	377	2	scaffold_180: 519069 - 522098	8.4e-182
PhWRKY40-1	Pahal.0072s0016	873	290	2	scaffold_72: 355868 - 357580	3.9e-157
PhWRKY40-2	Pahal.0046s0303	792	263	2	scaffold_46: 1976732 - 1978158	3.2e-126
PhWRKY42	Pahal.0105s0041	921	306	2	scaffold_105: 908459 - 909804	2.0e-126
PhWRKY43	Pahal.0019s0278	1755	584	5	scaffold_19: 1964519 - 1967309	6.2e-313
PhWRKY44	Pahal.0056s0177	990	329	2	scaffold_56: 1345550 - 1346844	2.4e-152
PhWRKY45	Pahal.0025s0214	939	312	3	scaffold_25: 2393948 - 2395788	1.8e-165
PhWRKY46-1	Pahal.0015s0207	678	225	2	scaffold_15: 1437222 - 1439847	4.0e-125
PhWRKY46-2	Pahal.0072s0015	678	225	2	scaffold_72: 348176 - 350725	9.7e-126
PhWRKY47	Pahal.0230s0011	1050	349	2	scaffold_230: 75257 - 77570	4.8e-156
PhWRKY48-1	Pahal.0078s0201	906	301	2	scaffold_78: 1369018 - 1370508	1.4e-94
PhWRKY48-2	Pahal.0078s0202	861	286	2	scaffold_78: 1374042 - 1375577	2.0e-93
PhWRKY48-3	Pahal.0078s0205	747	248	1	scaffold_78: 1405117 - 1405970	3.2e-35
PhWRKY49	Pahal.0019s0289	1227	408	2	scaffold_19: 2029920 - 2032832	1.7e-223
PhWRKY50	Pahal.0328s0049	939	312	2	scaffold_328: 351020 - 352910	6.2e-96

PhWRKY51	Pahal.0087s0003	1137	378	2	scaffold_87: 44068 - 45978	2.8e-201
PhWRKY52	Pahal.0015s0205	1002	333	1	scaffold_15: 1428362 - 1430052	1.5e-94
PhWRKY53-1	Pahal.0164s0054	1002	333	0	scaffold_164: 633409 - 634410	1.1e-154
PhWRKY53-2	Pahal.0150s0009	1461	486	2	scaffold_150: 164449 - 166660	1.3e-259
PhWRKY55	Pahal.0056s0090	657	218	2	scaffold_56: 744317 - 746729	4.1e-142
PhWRKY57	Pahal.0010s0053	594	197	2	scaffold_10: 301956 - 303811	1.5e-126
PhWRKY59	Pahal.0107s0079	4899	1632	15	scaffold_107: 1029515 - 1042493	0.0e+00
PhWRKY60	Pahal.0061s0164	468	155	2	scaffold_61: 1809623 - 1811489	2.0e-56
PhWRKY62	Pahal.0290s0006	804	267	2	scaffold_290: 44785 - 46038	4.9e-145
PhWRKY64	Pahal.0046s0306	873	290	2	scaffold_46: 1991725 - 1992945	5.9e-162
PhWRKY65	Pahal.0046s0302	966	321	2	scaffold_46: 1972224 - 1973933	4.3e-96
PhWRKY66	Pahal.0516s0013	1422	473	2	scaffold_516: 75158 - 78712	3.2e-270
PhWRKY67	Pahal.0085s0241	603	200	2	scaffold_85: 1496253 - 1497833	1.7e-107
PhWRKY68	Pahal.0094s0159	960	319	2	scaffold_94: 874900 - 876458	1.8e-189
PhWRKY70-1	Pahal.0121s0192	1806	601	3	scaffold_121: 1017038 - 1019971	0.0e+00
PhWRKY70-2	Pahal.0004s0019	576	191	1	scaffold_4: 127995 - 129912	2.1e-48
PhWRKY71	Pahal.0423s0011	1065	354	1	scaffold_423: 247618 - 249374	2.6e-180
PhWRKY72	Pahal.1077s0003	669	222	1	scaffold_1077: 9748 - 13055	6.8e-103
PhWRKY73	Pahal.0030s0057	1812	603	4	scaffold_30: 212864 - 217247	6.4e-286
PhWRKY74-1	Pahal.0420s0013	957	318	2	scaffold_420: 142651 - 144762	2.9e-143
PhWRKY74-2	Pahal.0420s0011	999	332	2	scaffold_420: 110369 - 112492	2.1e-158
PhWRKY76	Pahal.0003s0094	999	332	2	scaffold_3: 982145 - 983831	1.4e-173
PhWRKY77	Pahal.0026s0093	726	241	2	scaffold_26: 1300307 - 1301952	6.2e-75
PhWRKY78	Pahal.0020s0047	1815	604	5	scaffold_20: 895804 - 901643	0.0e+00
PhWRKY80	Pahal.0262s0053	1734	577	6	scaffold_262: 396251 - 400370	0.0e+00
PhWRKY81-1	Pahal.0046s0311	1029	342	1	scaffold_46: 2014316 - 2015902	4.6e-84
PhWRKY81-2	Pahal.0203s0008	1119	372	3	scaffold_203: 79074 - 85104	2.4e-228
PhWRKY82	Pahal.0316s0009	1752	583	3	scaffold_316: 385612 - 391611	1.3e-311
PhWRKY83-1	Pahal.0264s0011	1074	357	4	scaffold_264: 117760 - 121285	6.0e-224
PhWRKY83-2	Pahal.0007s0432	1047	348	3	scaffold_7: 3301706 - 3304737	3.7e-228
PhWRKY85	Pahal.0130s0066	1467	488	3	scaffold_130: 1067345 - 1072944	9.2e-298
PhWRKY86	Pahal.0006s0267	975	324	2	scaffold_6: 1577881 - 1580642	1.2e-239
PhWRKY87	Pahal.0052s0090	1209	402	3	scaffold_52: 980115 - 983266	1.2e-239

PhWRKY88	Pahal.0075s0122	1119	372	2	scaffold_75: 1371272 - 1375583	3.2e-156
PhWRKY90	Pahal.0030s0295	819	272	2	scaffold_30: 1734151 - 1735552	3.1e-126
PhWRKY91	Pahal.0078s0203	849	282	2	scaffold_78: 1391599 - 1396139	1.3e-92
PhWRKY92	Pahal.0019s0131	969	322	2	scaffold_19: 1163935 - 1165786	1.8e-170
PhWRKY93	Pahal.0081s0136	1122	373	2	scaffold_81: 947768 - 951200	5.3e-190
PhWRKY94	Pahal.0037s0094	4065	1354	4	scaffold_37: 851836 - 857889	0.0e+00
PhWRKY95	Pahal.0025s0225	1221	406	2	scaffold_25: 2678329 - 2681858	6.1e-189
PhWRKY97	Pahal.0003s0369	1503	500	3	scaffold_3: 2859009 - 2861211	1.2e-268
PhWRKY101	Pahal.0126s0030	777	258	3	scaffold_126: 211968 - 216067	2.5e-122
PhWRKY102	Pahal.0003s0338	792	263	2	scaffold_3: 2699440 - 2704838	4.4e-116
PhWRKY103	Pahal.0006s0108	1023	340	3	scaffold_6: 673038 - 677006	2.2e-132
Panicum virgatum						
PvWRKY1-1	Pavirv00018517m.g	1410	469	3	sg0.contig49092: 3069 - 5355	7.6e-150
PvWRKY1-2	Pavirv00024877m.g	1383	460	2	sg0.contig35974: 4057 - 5856	8.4e-134
PvWRKY2-1	Pavirv00010634m.g	1341	446	1	sg0.contig05245: 5189 - 6636	9.1e-180
PvWRKY2-2	Pavirv00062333m.g	1305	434	1	sg0.contig01221: 13249 - 14653	3.1e-171
PvWRKY3-1	Pavirv00031640m.g	840	279	2	sg0.contig124719: 1597 - 3113	2.2e-79
PvWRKY3-2	Pavirv00017243m.g	1077	358	2	sg0.contig49727: 1421 - 3912	4.4e-126
PvWRKY3-3	Pavirv00049589m.g	939	312	2	sg0.contig71071: 813 - 3531	6.0e-150
PvWRKY3-4	Pavirv00065590m.g	792	263	2	sg0.contig116611: 548 - 2962	9.6e-128
PvWRKY4-1	Pavirv00053344m.g	1308	435	3	sg0.contig112239: 388 - 2451	1.2e-239
PvWRKY4-2	Pavirv00061788m.g	2571	856	10	sg0.contig00886: 3079 - 8690	0.0e+00
PvWRKY5-1	Pavirv00001058m.g	1101	367	3	sg0.contig103473: 18 - 3670	1.7e-200
PvWRKY5-2	Pavirv00063802m.g	969	323	4	sg0.contig22823: 1207 - 4006	4.3e-155
PvWRKY5-3	Pavirv00022325m.g	1071	356	2	sg0.contig03365: 6821 - 9194	7.1e-172
PvWRKY6-1	Pavirv00069439m.g	1215	404	2	sg0.contig09946: 6513 - 8168	9.5e-213
PvWRKY6-2	Pavirv00038078m.g	1212	403	2	sg0.contig00208: 38497 - 40144	3.9e-207
PvWRKY7-1	Pavirv00040067m.g	669	222	2	sg0.contig55951: 1444 - 2405	1.7e-116
PvWRKY7-2	Pavirv00008412m.g	669	222	2	sg0.contig04214: 14877 - 16267	7.3e-117
PvWRKY8-1	Pavirv00050027m.g	1047	348	1	sg0.contig69331: 2322 - 4581	4.6e-157
PvWRKY8-2	Pavirv00004467m.g	1041	346	1	sg0.contig39094: 1342 - 3125	1.3e-153
PvWRKY10-1	Pavirv00065592m.g	705	234	2	sg0.contig117475: 1106 - 2012	4.9e-92
PvWRKY10-2	Pavirv00047822m.g	720	239	2	sg0.contig16283: 8164 - 9064	4.0e-104

PvWRKY11-1	Pavirv00038371m.g	1098	365	2	sg0.contig00561: 5238 - 7645	4.4e-200
PvWRKY11-2	Pavirv00027741m.g	651	216	1	sg0.contig35531: 4947 - 6434	3.6e-104
PvWRKY12-1	Pavirv00027106m.g	792	263	2	sg0.contig25372: 6766 - 8231	2.3e-125
PvWRKY12-2	Pavirv00068239m.g	858	285	2	sg0.contig09482: 11318 - 13089	2.6e-132
PvWRKY13-1	Pavirv00050645m.g	885	294	2	sg0.contig69592: 2292 - 4530	1.2e-164
PvWRKY13-2	Pavirv00027885m.g	726	241	1	sg0.contig349583: 1 - 1095	5.4e-135
PvWRKY14	Pavirv00061092m.g	897	298	1	sg0.contig01311: 15630 - 16750	1.0e-156
PvWRKY15-1	Pavirv00001985m.g	942	313	2	sg0.contig10525: 4689 - 5878	4.5e-134
PvWRKY15-2	Pavirv00037070m.g	945	314	2	sg0.contig00519: 10934 - 12257	5.1e-144
PvWRKY16	Pavirv00018854m.g	1212	403	2	sg0.contig51694: 2331 - 5342	9.7e-219
PvWRKY17	Pavirv00035347m.g	1140	379	2	sg0.contig91525: 555 - 2492	8.2e-93
PvWRKY18-1	Pavirv00016957m.g	780	259	3	sg0.contig64038: 678 - 4303	4.6e-124
PvWRKY18-2	Pavirv00044062m.g	654	217	4	sg0.contig37716: 479 - 5928	5.6e-82
PvWRKY18-3	Pavirv00017521m.g	690	229	2	sg0.contig47900: 2652 - 5498	1.9e-91
PvWRKY18-4	Pavirv00049300m.g	642	213	2	sg0.contig69313: 1755 - 3789	5.0e-120
PvWRKY18-5	Pavirv00050600m.g	573	191	1	sg0.contig72711: 3131 - 4552	5.0e-105
PvWRKY19-1	Pavirv00009895m.g	879	292	2	sg0.contig04287: 13252 - 14359	5.6e-126
PvWRKY19-2	Pavirv00063052m.g	876	291	2	sg0.contig01453: 4664 - 5780	1.4e-128
PvWRKY20	Pavirv00039691m.g	1026	341	2	sg0.contig55507: 2069 - 4005	1.9e-111
PvWRKY21-1	Pavirv00026598m.g	879	292	1	sg0.contig25361: 3239 - 4245	6.5e-136
PvWRKY21-2	Pavirv00043464m.g	849	282	1	sg0.contig30566: 6017 - 7005	9.0e-135
PvWRKY22-1	Pavirv00044157m.g	711	236	2	sg0.contig37770: 1210 - 4674	3.4e-137
PvWRKY22-2	Pavirv00069037m.g	732	243	2	sg0.contig09817: 8254 - 12069	4.2e-136
PvWRKY23-1	Pavirv00062996m.g	618	205	1	sg0.contig01001: 27293 - 28028	5.6e-74
PvWRKY23-2	Pavirv00009408m.g	597	198	1	sg0.contig05232: 1090 - 1992	3.9e-81
PvWRKY23-3	Pavirv00009708m.g	759	252	1	sg0.contig04558: 6150 - 9297	6.3e-111
PvWRKY24-1	Pavirv00042576m.g	1677	558	4	sg0.contig16677: 3516 - 5798	0.0e+00
PvWRKY24-2	Pavirv00069894m.g	1596	531	5	sg0.contig08416: 11163 - 13409	7.6e-299
PvWRKY25	Pavirv00016428m.g	867	288	0	sg0.contig61609: 1582 - 2448	9.3e-136
PvWRKY26-1	Pavirv00052099m.g	696	231	2	sg0.contig111419: 1980 - 2972	6.7e-96
PvWRKY26-2	Pavirv00070662m.g	705	234	2	sg0.contig33064: 1507 - 2529	9.6e-93
PvWRKY27-1	Pavirv00050721m.g	963	320	1	sg0.contig69032: 919 - 2089	1.7e-137
PvWRKY27-2	Pavirv00056372m.g	981	326	1	sg0.contig79649: 1242 - 2422	2.7e-135

PvWRKY30-3	Pavirv00041171m.g	1701	566	4	sg0.contig37240: 2165 - 4832	6.5e-316
PvWRKY30-1	Pavirv00038903m.g	2052	683	4	sg0.contig55239: 1176 - 4382	0.0e+00
PvWRKY30-2	Pavirv00048892m.g	711	236	1	sg0.contig158362: 1754 - 2705	1.0e-151
PvWRKY31-1	Pavirv00055861m.g	1002	333	1	sg0.contig76759: 1866 - 2995	4.9e-112
PvWRKY31-2	Pavirv00021649m.g	1170	389	1	sg0.contig14768: 4582 - 5872	3.0e-139
PvWRKY31-3	Pavirv00043094m.g	1266	421	0	sg0.contig305158: 30 - 1295	1.2e-97
PvWRKY32-1	Pavirv00032166m.g	1563	520	5	sg0.contig127640: 2 - 2323	2.6e-231
PvWRKY32-2	Pavirv00061157m.g	1695	564	4	sg0.contig01438: 10394 - 12730	2.6e-297
PvWRKY34-1	Pavirv00002817m.g	492	164	2	sg0.contig345092: 277 - 956	4.4e-97
PvWRKY34-2	Pavirv00003202m.g	549	182	2	sg0.contig312236: 347 - 1257	2.7e-99
PvWRKY35	Pavirv00011336m.g	1737	578	3	sg0.contig89891: 210 - 3135	1.8e-306
PvWRKY36-1	Pavirv00055062m.g	732	243	3	sg0.contig76110: 44 - 3413	1.7e-130
PvWRKY36-2	Pavirv00010062m.g	654	217	3	sg0.contig04727: 3455 - 6904	1.7e-118
PvWRKY36-3	Pavirv00065780m.g	678	225	3	sg0.contig117034: 517 - 3377	3.7e-143
PvWRKY37-1	Pavirv00043162m.g	1404	467	2	sg0.contig30337: 2348 - 5136	6.0e-268
PvWRKY37-2	Pavirv00057597m.g	1452	483	2	sg0.contig28707: 3570 - 6473	4.5e-280
PvWRKY39-1	Pavirv00035415m.g	1122	373	2	sg0.contig94203: 603 - 3134	1.3e-170
PvWRKY39-2	Pavirv00000224m.g	1077	358	3	sg0.contig39325: 1744 - 3868	1.4e-156
PvWRKY39-3	Pavirv00067903m.g	1047	348	2	sg0.contig09513: 3475 - 4751	9.9e-128
PvWRKY39-4	Pavirv00002176m.g	990	330	2	sg0.contig105785: 14 - 1228	3.6e-125
PvWRKY41	Pavirv00005806m.g	978	326	1	sg0.contig18022: 8423 - 9626	3.0e-189
PvWRKY42-1	Pavirv00053526m.g	951	316	2	sg0.contig113742: 1992 - 3126	4.0e-123
PvWRKY42-2	Pavirv00057672m.g	945	314	2	sg0.contig289144: 22 - 1156	2.0e-114
PvWRKY43-1	Pavirv00038700m.g	1500	500	5	sg0.contig00265: 37885 - 40068	2.7e-272
PvWRKY43-2	Pavirv00032754m.g	1365	455	5	sg0.contig12738: 9353 - 11404	5.5e-255
PvWRKY44	Pavirv00049526m.g	1011	336	2	sg0.contig72880: 1882 - 3094	1.7e-151
PvWRKY45-1	Pavirv00026100m.g	738	245	2	sg0.contig246151: 2 - 1279	9.4e-126
PvWRKY45-2	Pavirv00007067m.g	936	311	2	sg0.contig45687: 3828 - 5085	7.7e-168
PvWRKY46	Pavirv00041424m.g	675	224	2	sg0.contig37453: 687 - 4554	1.2e-127
PvWRKY47-1	Pavirv00013500m.g	1089	362	3	sg0.contig18441: 4077 - 6204	1.6e-119
PvWRKY47-2	Pavirv00058001m.g	753	250	2	sg0.contig195219: 654 - 2308	5.7e-117
PvWRKY48-1	Pavirv00038896m.g	567	188	1	sg0.contig56245: 16 - 735	2.3e-51
PvWRKY48-2	Pavirv00058430m.g	816	271	3	sg0.contig20007: 3447 - 4887	4.9e-79

PvWRKY48-3	Pavirv00052423m.g	606	201	3	sg0.contig111199: 1897 - 3115	3.4e-90
PvWRKY49-1	Pavirv00046378m.g	750	250	3	sg0.contig135288: 1131 - 2953	4.3e-115
PvWRKY49-2	Pavirv00020779m.g	1212	403	2	sg0.contig14381: 1275 - 3648	7.0e-211
PvWRKY50	Pavirv00022047m.g	828	275	2	sg0.contig144298: 880 - 2383	5.9e-63
PvWRKY51	Pavirv00042908m.g	1014	337	2	sg0.contig17085: 6994 - 8212	3.3e-183
PvWRKY52	Pavirv00004601m.g	1044	348	1	sg0.contig176760: 1265 - 2443	4.5e-76
PvWRKY55	Pavirv00059292m.g	663	220	2	sg0.contig14995: 67 - 1279	2.8e-138
PvWRKY57-1	Pavirv00042162m.g	1344	447	2	sg0.contig16644: 5352 - 8388	2.1e-209
PvWRKY57-2	Pavirv00048768m.g	828	275	3	sg0.contig164542: 113 - 2644	3.2e-112
PvWRKY57-3	Pavirv00047449m.g	804	267	3	sg0.contig164372: 102 - 2647	3.4e-104
PvWRKY58	Pavirv00036176m.g	630	209	2	sg0.contig97021: 1392 - 2478	1.5e-111
PvWRKY60	Pavirv00027198m.g	354	117	1	sg0.contig248491: 584 - 1304	7.5e-54
PvWRKY61	Pavirv00003238m.g	1146	382	2	sg0.contig314730: 84 - 1442	1.1e-175
PvWRKY62-1	Pavirv00012739m.g	828	275	2	sg0.contig99172: 1733 - 2857	6.4e-144
PvWRKY62-2	Pavirv00070849m.g	825	274	2	sg0.contig33194: 3466 - 4577	6.1e-149
PvWRKY64-1	Pavirv00011287m.g	873	290	2	sg0.contig84741: 1301 - 2414	6.3e-159
PvWRKY64-2	Pavirv00012439m.g	864	287	2	sg0.contig89171: 967 - 2167	1.9e-157
PvWRKY64-3	Pavirv00010955m.g	864	287	2	sg0.contig89320: 930 - 2133	6.3e-156
PvWRKY64	Pavirv00017999m.g	978	325	3	sg0.contig48629: 2301 - 3805	8.1e-123
PvWRKY65	Pavirv00036260m.g	969	322	2	sg0.contig95572: 2098 - 3815	9.0e-75
PvWRKY66	Pavirv00055255m.g	1425	474	2	sg0.contig74356: 1124 - 4505	8.4e-267
PvWRKY67-1	Pavirv00043826m.g	618	205	2	sg0.contig30467: 639 - 2082	5.9e-104
PvWRKY67-2	Pavirv00068323m.g	606	201	2	sg0.contig09123: 8703 - 9661	4.1e-104
PvWRKY68-1	Pavirv00027420m.g	948	315	2	sg0.contig35227: 839 - 2199	1.8e-190
PvWRKY68-2	Pavirv00069838m.g	951	316	2	sg0.contig08600: 1315 - 2659	2.3e-188
PvWRKY69-1	Pavirv00049728m.g	1005	334	0	sg0.contig71241: 3556 - 4638	3.1e-149
PvWRKY69-2	Pavirv00006996m.g	993	330	0	sg0.contig45488: 3707 - 4699	2.2e-147
PvWRKY70-1	Pavirv00031145m.g	1683	560	3	sg0.contig05929: 12408 - 15172	1.4e-307
PvWRKY70-2	Pavirv00010141m.g	1701	566	3	sg0.contig05106: 354 - 2758	3.4e-307
PvWRKY71-1	Pavirv00005222m.g	966	321	1	sg0.contig178168: 38 - 1255	1.3e-157
PvWRKY71-2	Pavirv00012070m.g	975	324	2	sg0.contig85921: 1492 - 2834	3.4e-174
PvWRKY71-3	Pavirv00037083m.g	993	330	2	sg0.contig00109: 9105 - 10290	1.3e-171
PvWRKY72-1	Pavirv00020938m.g	231	76	0	sg0.contig145844: 383 - 613	2.1e-53

PvWRKY72-1	Pavir.8NG230700	231	76	0	Chr08N:51823851..51826337	5.1e-42
PvWRKY74-1	Pavirv00028854m.g	996	331	2	sg0.contig07290: 12580 - 14276	3.6e-157
PvWRKY74-2	Pavirv00065647m.g	966	321	2	sg0.contig116216: 1270 - 2655	1.0e-136
PvWRKY74-3	Pavirv00056968m.g	741	246	2	sg0.contig28757: 10 - 1204	1.1e-108
PvWRKY76-1	Pavirv00046266m.g	975	324	2	sg0.contig134911: 1093 - 2265	1.7e-165
PvWRKY76-2	Pavirv00055081m.g	999	332	2	sg0.contig77257: 2060 - 3273	1.1e-163
PvWRKY77-1	Pavirv00070170m.g	675	224	2	sg0.contig332348: 33 - 1227	5.7e-72
PvWRKY77-2	Pavirv00021956m.g	678	225	2	sg0.contig142463: 666 - 1537	3.1e-54
PvWRKY78-1	Pavirv00042824m.g	1464	487	3	sg0.contig16674: 5505 - 8504	4.8e-291
PvWRKY78-2	Pavirv00070457m.g	1389	462	3	sg0.contig33041: 628 - 3582	3.5e-282
PvWRKY79	Pavirv00068795m.g	999	332	2	sg0.contig09315: 1529 - 2720	2.8e-151
PvWRKY80	Pavirv00039586m.g	1878	626	4	sg0.contig57260: 3 - 3513	0.0e+00
PvWRKY81	Pavirv00064554m.g	999	332	1	sg0.contig11990: 1431 - 2552	7.6e-78
PvWRKY82-1	Pavirv00029491m.g	1143	380	2	sg0.contig06834: 11315 - 14566	4.7e-227
PvWRKY82-2	Pavirv00007070m.g	1197	398	2	sg0.contig46195: 1024 - 5082	3.9e-245
PvWRKY82-3	Pavirv00061527m.g	996	331	2	sg0.contig01473: 9271 - 10686	3.0e-94
PvWRKY83-1	Pavirv00049214m.g	1074	357	2	sg0.contig73841: 1309 - 3642	7.9e-223
PvWRKY83-2	Pavirv00022316m.g	1047	348	3	sg0.contig03486: 10920 - 13670	3.6e-227
PvWRKY84	Pavirv00009420m.g	648	215	0	sg0.contig05260: 10446 - 11093	1.7e-69
PvWRKY85	Pavirv00008969m.g	1479	492	3	sg0.contig05523: 134 - 6603	5.2e-293
PvWRKY86	Pavirv00050108m.g	981	326	2	sg0.contig67227: 145 - 2157	7.3e-130
PvWRKY87-1	Pavirv00053696m.g	1185	394	3	sg0.contig11424: 8688 – 11335	4.0e-236
PvWRKY87-2	Pavirv00007485m.g	1206	401	2	sg0.contig43394: 2014 - 4385	2.1e-237
PvWRKY88-1	Pavirv00060853m.g	720	239	2	sg0.contig151986: 150 - 2762	4.3e-98
PvWRKY88-2	Pavirv00051308m.g	1143	380	2	sg0.contig22934: 4292 - 8437	3.4e-155
PvWRKY90-1	Pavirv00048744m.g	771	256	2	sg0.contig158726: 419 - 1688	3.3e-111
PvWRKY90-2	Pavirv00033119m.g	471	156	1	sg0.contig125052: 326 - 1007	1.7e-76
PvWRKY90-3	Pavirv00036392m.g	1734	577	4	sg0.contig90205: 1506 - 4003	0.0e+00
PvWRKY91	Pavirv00014140m.g	783	260	2	sg0.contig18533: 5511 - 8251	1.8e-127
PvWRKY92-1	Pavirv00002955m.g	945	314	2	sg0.contig34558: 5262 - 6431	2.1e-155
PvWRKY92-2	Pavirv00015878m.g	984	327	2	sg0.contig60612: 3406 - 4593	7.3e-171
PvWRKY93-1	Pavirv00024218m.g	1122	373	2	sg0.contig02645: 4337 - 6974	1.2e-188
PvWRKY93-2	Pavirv00053037m.g	810	269	1	sg0.contig111223: 2357 - 3389	6.7e-129

PvWRKY94-1	Pavirv00064303m.g	4560	1519	4	sg0.contig22731: 1949 - 8065	0.0e+00
PvWRKY94-2	Pavirv00057386m.g	618	206	2	sg0.contig28641: 5157 - 7720	8.9e-88
PvWRKY94-3	Pavirv00023378m.g	1047	348	3	sg0.contig02290: 6945 – 9748	2.2e-228
PvWRKY95-1	Pavirv00033647m.g	471	157	1	sg0.contig271618: 268 - 1661	4.0e-53
PvWRKY95-2	Pavirv00063150m.g	729	242	1	sg0.contig01581: 15669 - 16985	7.2e-51
PvWRKY95-3	Pavirv00029042m.g	735	244	1	sg0.contig07909: 12755 - 13713	1.8e-35
PvWRKY96	Pavirv00036578m.g	1278	426	2	sg0.contig95399: 107 - 2572	1.3e-253
PvWRKY97-1	Pavirv00026819m.g	1512	503	3	sg0.contig24751: 1381 - 3219	4.6e-260
PvWRKY97-2	Pavirv00051698m.g	1527	508	3	sg0.contig23371: 5177 - 7061	2.1e-265
PvWRKY102	Pavirv00035850m.g	654	217	1	sg0.contig91650: 1875 - 2657	1.1e-111
PvWRKY103-1	Pavirv00004891m.g	564	187	1	sg0.contig17756: 7943 - 8616	4.2e-85
PvWRKY103-2	Pavirv00030906m.g	1497	498	7	sg0.contig07579: 4336 - 8591	0.0e+00
PvWRKY105-1	Pavirv00003246m.g	1434	477	4	sg0.contig31589: 1336 - 5815	9.1e-269
PvWRKY105-2	Pavirv00058232m.g	1218	405	3	sg0.contig19912: 4649 - 9125	1.3e-225
PvWRKY106	Pavirv00019417m.g	504	167	0	sg0.contig27527: 6138 - 6641	1.3e-63
Phaseolus vulgaris						
PvulWRKY1-1	Phvul.008G286100	1518	505	4	Chr08: 59105885 - 59109637	0.0e+00
PvulWRKY1-2	Phvul.008G270500	1371	456	5	Chr08: 58044223 - 58046591	4.1e-305
PvulWRKY1-3	Phvul.005G116000	1854	617	4	Chr05: 33530600 - 33533721	0.0e+00
PvulWRKY1-4	Phvul.011G101900	1800	599	4	Chr11: 11097802 - 11100927	0.0e+00
PvulWRKY1-5	Phvul.006G147800	1836	611	6	Chr06: 26094790 - 26097688	0.0e+00
PvulWRKY1-6	Phvul.006G047300	1335	444	5	Chr06: 16091296 - 16095072	4.7e-290
PvulWRKY2	Phvul.008G054100	2145	714	4	Chr08: 4839408 - 4843253	0.0e+00
PvulWRKY3	Phvul.002G103400	1314	437	3	Chr02: 20868782 - 20871665	6.3e-297
PvulWRKY4	Phvul.002G091100	1365	454	3	Chr02: 15467166 - 15471721	3.2e-309
PvulWRKY5-1	Phvul.003G124000	1200	399	6	Chr03: 30385985 - 30389924	2.1e-261
PvulWRKY5-2	Phvul.009G220700	1251	416	5	Chr09: 32729678 - 32733766	1.8e-281
PvulWRKY5-3	Phvul.008G048100	672	223	3	Chr08: 4250703 - 4259563	7.8e-133
PvulWRKY7	Phvul.001G213600	786	261	2	Chr01: 47682848 - 47684741	5.4e-170
PvulWRKY9-1	Phvul.002G202500	1461	486	4	Chr02: 36234759 - 36237703	0.0e+00
PvulWRKY9-2	Phvul.008G043000	1620	539	3	Chr08: 3709079 - 3711997	0.0e+00
PvulWRKY11-1	Phvul.009G233900	903	300	2	Chr09: 34493623 - 34495359	4.8e-200
PvulWRKY11-2	Phvul.003G139500	963	320	2	Chr03: 33212871 - 33214510	1.4e-127

PvulWRKY11-3	Phvul.009G101900	891	296	2	Chr09: 15494961 - 15496400	1.4e-197
PvulWRKY13-1	Phvul.009G138600	708	235	2	Chr09: 20401487 - 20404607	3.8e-159
PvulWRKY13-2	Phvul.002G266400	690	229	2	Chr02: 43213877 - 43215924	1.9e-155
PvulWRKY14	Phvul.006G123000	1488	495	2	Chr06: 23845672 - 23849501	0.0e+00
PvulWRKY15-1	Phvul.002G016100	978	325	2	Chr02: 1777772 - 1779281	4.8e-212
PvulWRKY15-2	Phvul.003G240900	960	319	2	Chr03: 46413673 - 46415523	5.8e-210
PvulWRKY17	Phvul.001G060200	960	319	2	Chr01: 7241162 - 7243932	7.1e-214
PvulWRKY19-1	Phvul.008G081800	915	304	2	Chr08: 7871958 - 7873756	8.4e-200
PvulWRKY19-2	Phvul.010G057900	858	285	2	Chr10: 9634279 - 9635768	6.4e-193
PvulWRKY18	Phvul.009G087400	873	290	3	Chr09: 13639295 - 13640736	1.5e-192
PvulWRKY20-1	Phvul.008G275300	1755	584	5	Chr08: 58435600 - 58441263	0.0e+00
PvulWRKY20-2	Phvul.006G053300	1746	581	5	Chr06: 16845731 - 16851077	0.0e+00
PvulWRKY21	Phvul.007G177900	1077	358	2	Chr07: 41387786 - 41390521	3.3e-240
PvulWRKY22-1	Phvul.010G104200	1089	362	2	Chr10: 36231937 - 36233733	2.3e-240
PvulWRKY22-2	Phvul.008G058300	1041	346	2	Chr08: 5181941 - 5183860	3.1e-226
PvulWRKY23-1	Phvul.007G212900	960	319	2	Chr07: 45186238 - 45188369	1.4e-212
PvulWRKY23-2	Phvul.001G214400	882	293	2	Chr01: 47770993 - 47773054	2.2e-196
PvulWRKY26-1	Phvul.008G251300	1719	572	4	Chr08: 56596899 - 56600140	0.0e+00
PvulWRKY26-2	Phvul.006G074600	1614	537	4	Chr06: 19393601 - 19396850	0.0e+00
PvulWRKY27-1	Phvul.004G105800	1206	401	2	Chr04: 34620507 - 34622280	2.1e-272
PvulWRKY27-2	Phvul.007G075400	948	315	2	Chr07: 6907213 - 6908842	1.9e-212
PvulWRKY28	Phvul.001G042100	810	269	3	Chr01: 4312182 - 4314073	2.7e-174
PvulWRKY29-1	Phvul.002G160100	720	239	1	Chr02: 30201373 - 30202358	1.7e-162
PvulWRKY29-2	Phvul.002G293200	879	292	1	Chr02: 45663178 - 45664544	3.3e-201
PvulWRKY32-1	Phvul.003G156300	1515	504	4	Chr03: 36109027 - 36115421	0.0e+00
PvulWRKY32-2	SPhvul.009G195200	1425	474	4	Chr09: 28905568 - 28913126	0.0e+00
PvulWRKY33-1	Phvul.010G062500	1491	496	4	Chr10: 11258171 - 11261871	0.0e+00
PvulWRKY33-2	Phvul.008G090300	1620	539	4	Chr08: 9198199 - 9201344	0.0e+00
PvulWRKY33-3	Phvul.008G251700	990	329	0	Chr08: 56633693 - 56635151	2.0e-225
PvulWRKY33-4	Phvul.008G251800	960	329	4	Chr08: 56635386 - 56639980	1.6e-216
PvulWRKY34	Phvul.005G005800	2223	740	5	Chr05: 452542 - 457216	0.0e+00
PvulWRKY35	Phvul.002G202000	1308	435	2	Chr02: 36152085 - 36156289	6.0e-295
PvulWRKY40-1	Phvul.005G181800	912	303	4	Chr05: 40322573 - 40324669	1.8e-195

PvulWRKY40-2	Phvul.010G161100	945	314	5	Chr10: 42927172 - 42930758	1.2e-204
PvulWRKY43-1	Phvul.010G104700	609	202	1	Chr10: 36413066 - 36414301	6.3e-136
PvulWRKY43-2	Phvul.008G058000	540	179	2	Chr08: 5159821 - 5163155	1.0e-93
PvulWRKY44	Phvul.001G173700	1413	470	6	Chr01: 43670815 - 43675762	0.0e+00
PvulWRKY45-1	Phvul.008G185800	771	256	2	Chr08: 48910744 - 48918666	7.2e-173
PvulWRKY45-2	Phvul.008G185700	933	310	2	Chr08: 48904565 - 48906193	6.0e-213
PvulWRKY45-3	Phvul.009G043100	849	282	2	Chr09: 8430030 - 8432516	2.0e-189
PvulWRKY46-1	Phvul.006G111700	1122	373	2	Chr06: 22762481 - 22764805	9.5e-256
PvulWRKY46-2	Phvul.010G111900	1110	369	2	Chr10: 37576223 - 37578860	6.3e-246
PvulWRKY47-1	Phvul.001G218500	1560	519	5	Chr01: 48108877 - 48112906	0.0e+00
PvulWRKY47-2	Phvul.007G209000	1389	462	6	Chr07: 44775550 - 44778536	5.6e-313
PvulWRKY48-1	Phvul.003G116300	1197	398	2	Chr03: 29019242 - 29021426	1.3e-266
PvulWRKY48-2	Phvul.006G119100	1056	351	2	Chr06: 23452021 - 23455089	1.9e-240
PvulWRKY49	Phvul.008G119400	936	311	2	Chr08: 15127288 - 15130305	1.0e-210
PvulWRKY50	Phvul.002G265400	564	187	2	Chr02: 43085670 - 43087004	9.9e-125
PvulWRKY51-1	Phvul.009G080000	477	158	2	Chr09: 12910792 - 12911776	1.2e-104
PvulWRKY51-2	Phvul.001G039900	459	152	2	Chr01: 4074888 - 4076353	2.2e-102
PvulWRKY58	Phvul.008G039900	1557	518	3	Chr08: 3354570 - 3359078	0.0e+00
PvulWRKY59-1	Phvul.007G118200	1098	365	3	Chr07: 18968507 - 18974116	8.0e-252
PvulWRKY59-2	Phvul.007G118100	1074	357	4	Chr07: 18951902 - 18954607	4.8e-245
PvulWRKY60	Phvul.001G042200	843	280	3	Chr01: 4326381 - 4327985	3.1e-185
PvulWRKY65	Phvul.002G240900	774	257	2	Chr02: 40784105 - 40786025	3.1e-173
PvulWRKY68	Phvul.009G138900	582	193	2	Chr09: 20451693 - 20454202	2.8e-209
PvulWRKY69-1	Phvul.002G163400	1041	346	2	Chr02: 30515098 - 30516646	1.6e-236
PvulWRKY69-2	Phvul.002G297100	1086	361	2	Chr02: 46023368 - 46025419	2.3e-250
PvulWRKY69-3	Phvul.009G137500	1059	352	2	Chr09: 20185631 - 20187441	4.4e-172
PvulWRKY69-3	Phvul.005G093800	762	253	2	Chr05: 27948848 - 27951057	4.4e-172
PvulWRKY71-1	PPhvul.002G196800	1077	358	2	Chr02: 35413372 - 35415513	7.3e-243
PvulWRKY71-2	Phvul.008G192800	1002	333	2	Chr08: 49929870 - 49931980	4.3e-225
PvulWRKY73-1	Phvul.003G192000	1935	644	4	Chr03: 40445375 - 40449886	0.0e+00
PvulWRKY73-2	Phvul.009G189700	1788	595	4	Chr09: 28139133 - 28142787	0.0e+00
PvulWRKY73-3	Phvul.003G068700	1392	463	3	Chr03: 10126584 - 10128409	1.8e-312
PvulWRKY73-4	Phvul.002G089700	1317	438	2	Chr02: 14907240 - 14909229	3.7e-299

PvulWRKY75	Phvul.001G088200	771	256	1	Chr01: 15930050 - 15932342	8.3e-172
PvulWRKY79-1	Phvul.005G080200	906	301	2	Chr05: 18309095 - 18311322	7.9e-207
PvulWRKY79-2	Phvul.005G080400	843	280	2	Chr05: 18435912 - 18437363	4.6e-190
PvulWRKY79-3	Phvul.005G080300	915	304	2	Chr05: 18328284 - 18331020	5.0e-202
PvulWRKY97	Phvul.002G081600	879	292	4	Chr02: 12429691 - 12436481	1.5e-196
Physcomitrella patens						
PpWRKY3	Pp1s135_80V6	2247	748	5	scaffold_135: 645772 - 650727	4.7e-170
PpWRKY4	Pp1s426_27V6	1314	437	2	scaffold_426: 133251 - 135010	8.1e-54
PpWRKY9-1	Pp1s2_539V6	1230	409	6	scaffold_2: 3255923 - 3259213	2.8e-223
PpWRKY9-2	Pp1s11_241V6	1476	491	2	scaffold_11: 1923004 - 1925119	2.1e-67
PpWRKY13	Pp1s5_354V6	1731	576	3	scaffold_5: 2573388 - 2577075	6.0e-64
PpWRKY15	Pp1s48_141V6	1188	395	4	scaffold_48: 1159423 - 1162577	1.5e-261
PpWRKY20	Pp1s35_336V6	2118	705	4	scaffold_35: 1948237 - 1952437	2.9e-112
PpWRKY21-1	Pp1s163_112V6	1191	396	4	scaffold_163: 806509 - 808836	6.5e-266
PpWRKY21-2	Pp1s42_169V6	1032	343	2	scaffold_42: 1397607 - 1401930	1.7e-45
PpWRKY21-3	Pp1s7_13V6	1188	395	3	scaffold_7: 59439 - 61638	4.2e-264
PpWRKY23	Pp1s39_217V6	1521	506	2	scaffold_39: 1014881 - 1017338	3.5e-76
PpWRKY28-1	Pp1s235_126V6	1854	617	2	scaffold_235: 597735 - 600433	4.9e-54
PpWRKY28-2	Pp1s159_105V6	2172	723	2	scaffold_159: 530929 - 533445	7.0e-60
PpWRKY28-3	Pp1s109_123V6	1602	533	2	scaffold_109: 814971 - 818298	1.9e-66
PpWRKY33	Pp1s157_29V6	2055	684	3	scaffold_157: 177897 - 181275	2.3e-118
PpWRKY42-1	Pp1s158_166V6	2298	765	5	scaffold_158: 827946 - 831738	1.1e-70
PpWRKY42-2	Pp1s47_251V6	2511	836	7	scaffold_47: 1597512 - 1602301	8.4e-76
PpWRKY42-3	Pp1s246_89V6	2247	748	3	scaffold_246: 427002 - 430434	4.1e-69
PpWRKY46-1	Pp1s84_106V6	1608	535	4	scaffold_84: 596548 - 599697	0.0e+00
PpWRKY46-2	Pp1s195_75V6	1827	608	6	scaffold_195: 526157 - 529122	5.3e-293
PpWRKY50	Pp1s426_30V6	1755	584	2	scaffold_426: 161343 - 164316	6.4e-183
PpWRKY55-1	Pp1s36_309V6	1596	531	5	scaffold_36: 2059942 - 2063011	1.4e-303
PpWRKY55-2	Pp1s280_53V6	1464	487	3	scaffold_280: 365791 - 367965	0.0e+00
PpWRKY48-1	Pp1s244_77V6	486	161	1	scaffold_244: 571901 - 573568	2.8e-56
PpWRKY48-2	Pp1s39_212V6	1536	511	2	scaffold_39: 994890 - 997050	7.2e-51
PpWRKY57-1	Pp1s407_55V6	1515	504	2	scaffold_407: 349280 - 352092	1.6e-56
PpWRKY57-2	Pp1s35_401V6	2574	857	4	scaffold_35: 2394239 - 2399016	1.5e-53

PpWRKY57-3	Pp1s73_70V6	1677	558	2	scaffold_73: 359539 - 362169	4.0e-63
PpWRKY57-4	Pp1s140_87V6	1731	576	2	scaffold_140: 533870 - 536059	1.2e-80
PpWRKY57-5	Pp1s115_10V6	2082	693	3	scaffold_115: 59551 - 63343	1.1e-53
PpWRKY57-6	Pp1s166_34V6	1767	588	2	scaffold_166: 286374 - 288922	1.1e-53
PpWRKY57-7	Pp1s198_83V6	2277	758	2	scaffold_198: 445752 - 450271	2.1e-59
PpWRKY57-8	Pp1s137_252V6	1701	566	2	scaffold_137: 889823 - 891759	6.9e-49
PpWRKY57-9	Pp1s25_173V6	1749	582	4	scaffold_25: 1200807 - 1204925	4.5e-51
PpWRKY74	Pp1s65_236V6	1158	385	3	scaffold_65: 1330173 - 1332187	4.7e-257
Picea abies						
PaWRKY6-1	MA_103616g0010	1941	646	5	MA_10361:27192..30835	1.7e-192
PaWRKY6-2	MA_161426g0010	1083	360	4	MA_161426:4031..10043	2.3e-93
PaWRKY6-3	MA_14480g0020	1158	385	4	MA_14480:16357..18219	4.4e-62
PaWRKY6-4	MA_47052g0010	1089	362	3	MA_47052:12328..13765	4.2e-58
PaWRKY6-5	MA_6918834g0010	672	223	2	MA_6918834: -19..1545	2.8e-46
PaWRKY6-6	MA_558583g0010	894	297	3	MA_558583:1596..3027	2.0e-58
PaWRKY6-7	MA_175750g0010	1029	342	3	MA_175750:11808..14051	9.2e-59
PaWRKY6-8	MA_10436051g0010	1065	354	4	MA_10436051: 7403..8951	1.5e-84
PaWRKY6-9	MA_212937g0010	1878	625	5	MA_212937:1948..4339	1.3e-206
PaWRKY7	MA_88419g0010	969	322	2	MA_88419: 10736..13638	4.9e-211
PaWRKY9	MA_381058g0010	591	196	2	MA_381058:3296..4322	1.9e-28
PaWRKY10	MA_76002g0010	333	110	1	MA_76002:12209..12733	2.1e-33
PaWRKY11-1	MA_10436931g0040	294	97	1	MA_10436931:15746..16409	1.1e-53
PaWRKY11-2	MA_107950g0010	1182	393	2	MA_107950:1058..3100	2.0e-247
PaWRKY11-3	MA_10428770g0010	643	213	2	MA_10428770:-19..1984	3.2e-51
PaWRKY13	MA_133396g0010	786	261	3	MA_133396:18090..19553	2.9e-60
PaWRKY14-1	MA_138256g0010	1326	441	2	MA_138256:4131..6703	5.5e-58
PaWRKY14-2	MA_109104g0010	1470	489	3	MA_109104:3506..6286	9.8e-84
PaWRKY20-1	MA_120969g0010	1584	527	5	MA_120969:10441..13650	0.0e+00
PaWRKY20-2	MA_10426648g0010	1744	580	5	MA_10426648:-19..24269	5.0e-205
PaWRKY21-1	MA_11492g0010	1347	448	5	MA_11492:70221..74699	3.9e-107
PaWRKY21-2	MA_10428170g0010	1029	342	2	MA_10428170:26510..31119	4.6e-223
PaWRKY22	MA_8710165g0010	711	236	1	MA_8710165:47..977	9.9e-64
PaWRKY23	MA_54954g0010	573	190	2	MA_54954:514..1812	4.2e-46

PaWRKY24	MA_10426942g0010	771	256	2	MA_10426942:6661..9859	4.8e-56
PaWRKY26-1	MA_16118g0010	2700	899	5	MA_16118:25474..35490	0.0e+00
PaWRKY26-2	MA_53351g0010	1956	651	5	MA_53351:4859..16486	1.9e-104
PaWRKY31-1	MA_164675g0010	1074	357	3	MA_164675:15102..17482	6.6e-97
PaWRKY31-2	MA_134559g0010	870	289	3	MA_134559:1038..2826	1.5e-40
PaWRKY33	MA_10434651g0010	1896	631	4	MA_10434651:16875..34465	4.3e-106
PaWRKY35-1	MA_136551g0010	666	221	2	MA_136551:4606..7880	3.8e-47
PaWRKY35-2	MA_10432914g0010	1389	462	2	MA_10432914:5215..7238	1.9e-66
PaWRKY35-3	MA_10433300g0020	873	290	1	MA_10433300:18109..19234	5.3e-68
PaWRKY42-1	MA_5558504g0010	645	214	2	MA_5558504:-19..860	1.1e-40
PaWRKY42-2	MA_47307g0010	1194	397	4	MA_47307:21340..23609	1.3e-66
PaWRKY42-3	MA_52928g0010	1125	374	3	MA_52928:6670..8698	2.3e-59
PaWRKY42-4	MA_934202g0010	1104	367	4	MA_934202:479..2352	5.8e-137
PaWRKY42-5	MA_6790522g0010	636	211	1	MA_6790522:407..1200	6.2e-25
PaWRKY42-6	MA_46529g0010	978	325	3	MA_46529:13169..14784	9.4e-89
PaWRKY42-7	MA_136063g0010	519	172	1	MA_136063:13616..14292	7.0e-46
PaWRKY49	MA_10434450g0010	1359	452	2	MA_10434450:7431..11373	2.8e-40
PaWRKY50	MA_306130g0010	486	161	2	MA_306130:1659..2410	5.4e-45
PaWRKY51-1	MA_65782g0010	843	280	2	MA_65782:16207..17418	5.6e-50
PaWRKY51-2	MA_23415g0010	798	265	2	MA_23415:9281..10359	2.6e-81
PaWRKY55-1	MA_6177g0010	1719	572	5	MA_6177:31773..40849	1.5e-29
PaWRKY55-2	MA_2290g0010	1071	356	4	MA_2290:12895..21921	1.3e-42
PaWRKY57-1	MA_126273g0010	1416	471	3	MA_126273:22086..24134	5.4e-72
PaWRKY57-2	MA_10434976g0010	1362	453	2	MA_10434976:6409..8267	1.7e-113
PaWRKY57-3	MA_120697g0010	1365	454	2	MA_120697:8599..10217	5.4e-113
PaWRKY57-4	MA_20468g0010	732	243	2	MA_20468:4225..6187	2.8e-48
PaWRKY57-5	MA_93790g0010	525	174	2	MA_93790:13764..14883	1.3e-46
PaWRKY57-6	MA_10432362g0010	657	218	2	MA_10432362:30..1918	4.0e-48
PaWRKY65	MA_124797g0010	1125	374	3	MA_124797:9012..11092	1.8e-113
PaWRKY69	MA_6943842g0010	1056	351	1	MA_6943842:800..2021	6.6e-82
PaWRKY71-1	MA_740729g0010	267	88	1	MA_740729:1169..1573	1.3e-52
PaWRKY71-2	MA_7513437g0010	611	203	2	MA_7513437:-19..964	5.7e-49
PaWRKY71-3	MA_179641g0010	624	207	2	MA_179641:6210..7224	6.2e-48

PaWRKY71-4	MA_496691g0010	582	193	2	MA_496691:6053..7260	1.0e-29
PaWRKY72-1	MA_10428091g0010	435	144	3	MA_10428091:4888..7987	3.4e-137
PaWRKY72-2	MA_74833g0010	2196	731	4	MA_74833:9458..13719	1.8e-137
PaWRKY72	MA_892467g0010	2121	706	5	MA_892467:2865..7140	8.9e-49
PaWRKY75	MA_10425932g0020	819	272	3	MA_10425932:24588..38975	1.2e-177
Populous trichocarpa						
PtWRKY1-1	Potri.014G164300	1458	485	5	Chr14: 12985555 - 12989438	0.0e+00
PtWRKY1-2	Potri.002G221600	1107	368	4	Chr02: 20862009 - 20864972	5.0e-203
PtWRKY2	Potri.011G169300	2178	725	5	Chr11: 18450851 - 18455892	0.0e+00
PtWRKY3	Potri.010G163000	1500	499	3	Chr10: 16770595 - 16774139	0.0e+00
PtWRKY4-1	Potri.017G088300	1608	535	3	Chr17: 10559295 - 10564769	0.0e+00
PtWRKY4-2	Potri.004G120800	1632	543	3	Chr04: 11338804 - 11343998	3.4e-299
PtWRKY4-3	Potri.008G091900	1479	492	3	Chr08: 5759575 - 5763061	0.0e+00
PtWRKY5-1	Potri.002G138900	612	203	3	Chr02: 10319005 - 10322693	1.2e-138
PtWRKY5-2	PPotri.014G050000	687	228	3	Chr14: 4012998 - 4017289	9.0e-155
PtWRKY6-1	Potri.004G007500	1737	578	6	Chr04: 479125 - 481848	0.0e+00
PtWRKY6-2	Potri.011G007800	1782	593	5	Chr11: 576073 - 578528	0.0e+00
PtWRKY6-3	Potri.002G228400	1854	617	4	Chr02: 21820126 - 21822588	0.0e+00
PtWRKY6-4	Potri.014G155100	1875	624	4	Chr14: 12072684 - 12075554	0.0e+00
PtWRKY7-1	Potri.002G123300	1077	358	2	Chr02: 9218413 - 9220464	1.5e-238
PtWRKY7-2	PPotri.014G024200	1167	388	3	Chr14: 2074611 - 2077146	3.0e-257
PtWRKY9	Potri.001G208600	1560	519	4	Chr01: 20898737 - 20901171	0.0e+00
PtWRKY10	Potri.001G002400	408	135	3	Chr01: 152953 - 155561	4.3e-87
PtWRKY11-1	Potri.006G072400	906	301	2	Chr06: 5375343 - 5377292	2.1e-199
PtWRKY11-2	Potri.018G139300	903	300	2	Chr18: 15890044 - 15892257	5.0e-200
PtWRKY11-3	Potri.018G008500	1017	338	2	Chr18: 587550 - 590270	4.2e-225
PtWRKY13-1	Potri.005G086400	672	223	2	Chr05: 6445488 - 6450574	1.9e-139
PtWRKY13-2	Potri.007G078200	702	233	3	Chr07: 10194776 - 10198496	1.1e-157
PtWRKY14-1	Potri.011G157100	1353	450	3	Chr11: 17485027 - 17487804	3.7e-299
PtWRKY14-2	Potri.001G460600	1272	423	3	Chr01: 49433744 - 49436406	2.6e-288
PtWRKY14-3	Potri.003G132700	993	330	2	Chr03: 15201353 - 15202963	6.4e-221
PtWRKY15-1	Potri.005G141400	996	331	2	Chr05: 11954643 - 11956649	1.1e-217
PtWRKY15-2	Potri.007G047400	1008	335	2	Chr07: 4352721 - 4355025	8.0e-221

PtWRKY18	Potri.006G263600	963	320	4	Chr06: 26679119 - 26680878	7.7e-205
PtWRKY19	Potri.014G009500	513	170	2	Chr14: 968858 - 969713	5.4e-113
PtWRKY20-1	Potri.011G087900	1686	561	5	Chr11: 10507794 - 10514869	0.0e+00
PtWRKY20-2	Potri.001G361600	1677	558	5	Chr01: 37367919 - 37373720	0.0e+00
PtWRKY20-3	Potri.019G053900	1551	516	5	Chr19: 8279037 - 8281782	1.5e-213
PtWRKY20-4	Potri.013G086000	2151	716	4	Chr13: 8100301 - 8104436	0.0e+00
PtWRKY21-1	Potri.002G043500	1056	351	2	Chr02: 2783127 - 2786060	6.8e-232
PtWRKY21-2	Potri.005G219500	1044	347	2	Chr05: 23151059 - 23153203	5.9e-231
PtWRKY22-1	Potri.002G164400	1065	354	2	Chr02: 12421400 - 12423249	7.8e-240
PtWRKY22-2	Potri.014G090300	1050	349	2	Chr14: 7098996 - 7100670	2.6e-235
PtWRKY23-1	Potri.002G193000	954	317	2	Chr02: 15398964 - 15402809	4.7e-209
PtWRKY23-2	Potri.014G118200	960	319	2	Chr14: 9188997 - 9190859	4.1e-193
PtWRKY26-1	Potri.019G123500	1443	480	4	Chr19: 15161763 - 15164710	0.0e+00
PtWRKY26-2	Potri.013G153400	1776	591	4	Chr13: 15672152 - 15675074	0.0e+00
PtWRKY27-1	Potri.004G072000	1293	430	2	Chr04: 6057099 - 6058855	8.6e-291
PtWRKY27-2	Potri.017G149000	1239	412	2	Chr17: 15509535 - 15511261	6.4e-279
PtWRKY29	Potri.001G099000	504	167	1	Chr01: 7812739 - 7813453	9.9e-109
PtWRKY32-1	Potri.018G107000	1596	531	4	Chr18: 13340738 - 13347744	0.0e+00
PtWRKY32-2	Potri.006G184800	1569	522	4	Chr06: 19846841 - 19852326	0.0e+00
PtWRKY32-3	Potri.006G264000	1434	477	4	Chr06: 26721773 - 26724592	0.0e+00
PtWRKY32-4	Potri.018G019000	1401	466	4	Chr18: 1444549 - 1447195	2.0e-282
PtWRKY33-1	Potri.016G128300	1740	579	4	Chr16: 13096393 - 13099016	0.0e+00
PtWRKY33-2	Potri.006G105300	1641	546	4	Chr06: 8125329 - 8128097	0.0e+00
PtWRKY34	Potri.001G472800	2199	732	5	Chr01: 50403473 - 50408374	0.0e+00
PtWRKY35	Potri.017G104800	714	237	2	Chr17: 12179899 - 12182330	9.4e-178
PtWRKY39	Potri.013G042600	1065	354	2	Chr13: 2993743 - 2996226	6.4e-239
PtWRKY40-1	Potri.003G182200	960	319	4	Chr03: 18904988 - 18907381	2.5e-208
PtWRKY40-2	Potri.001G044500	957	318	3	Chr01: 3209312 - 3211607	1.5e-208
PtWRKY40-3	Potri.018G019700	816	271	3	Chr18: 1491298 - 1492943	7.9e-180
PtWRKY41-1	Potri.014G096200	1098	365	2	Chr14: 7526597 - 7529192	1.6e-250
PtWRKY41-2	Potri.002G168700	1092	363	2	Chr02: 12778165 - 12781030	1.6e-248
PtWRKY43-1	Potri.014G090700	570	189	1	Chr14: 7118925 - 7121224	6.7e-128
PtWRKY43-2	Potri.002G164900	582	193	1	Chr02: 12450751 - 12452084	1.5e-129

PtWRKY44-1	Potri.016G083600	1410	469	7	Chr16: 6509598 - 6514741	1.1e-252
PtWRKY44-2	Potri.006G133200	1428	475	6	Chr06: 10969866 - 10974789	0.0e+00
PtWRKY45-1	PPotri.012G101000	561	186	1	Chr12: 12608185 - 12609465	7.6e-126
PtWRKY45-2	Potri.015G099200	537	178	1	Chr15: 11835261 - 11837075	5.1e-119
PtWRKY47-1	Potri.014G111900	1509	502	5	Chr14: 8765075 - 8768810	0.0e+00
PtWRKY47-2	Potri.002G186600	1521	506	5	Chr02: 14641279 - 14644811	0.0e+00
PtWRKY48-1	Potri.010G147700	1125	374	2	Chr10: 15695587 - 15697571	9.2e-249
PtWRKY48-2	Potri.008G103300	1107	368	2	Chr08: 6548117 - 6549941	3.2e-245
PtWRKY49-1	Potri.006G087000	915	304	2	Chr06: 6643583 - 6645418	2.3e-202
PtWRKY49-2	Potri.016G099900	930	309	2	Chr16: 9770417 - 9772150	2.6e-206
PtWRKY50	Potri.006G224100	498	165	2	Chr06: 23609786 - 23611128	5.1e-109
PtWRKY51-1	Potri.007G079800	579	192	2	Chr07: 10382370 - 10385056	1.0e-122
PtWRKY51-2	Potri.005G085200	621	206	2	Chr05: 6324424 - 6327310	7.9e-135
PtWRKY53-1	Potri.001G092900	1017	338	2	Chr01: 7326486 - 7329009	3.6e-231
PtWRKY53-2	Potri.003G138600	1029	342	2	Chr03: 15656901 - 15658916	3.5e-234
PtWRKY53-3	Potri.012G031700	1116	371	2	Chr12: 2820069 - 2822264	1.5e-250
PtWRKY54-1	Potri.016G137900	966	321	2	Chr16: 14049379 - 14051741	1.0e-218
PtWRKY54-2	Potri.006G109100	1002	333	2	Chr06: 8522038 - 8524071	2.7e-229
PtWRKY55-1	Potri.019G059300	756	251	2	Chr19: 9106420 - 9108112	3.2e-142
PtWRKY55-2	Potri.013G090400	1059	352	2	Chr13: 9549330 - 9551441	7.4e-223
PtWRKY57-1	Potri.010G160100	882	293	4	Chr10: 16599809 - 16606223	6.7e-198
PtWRKY57-2	Potri.008G094000	882	293	3	Chr08: 5884501 - 5889490	1.3e-200
PtWRKY60	Potri.018G019800	963	320	4	Chr18: 1505147 - 1507026	2.8e-208
PtWRKY65-1	Potri.004G060400	789	262	2	Chr04: 4884455 - 4886727	3.6e-177
PtWRKY65-2	Potri.004G060900	789	262	2	Chr04: 4967273 - 4969191	3.6e-177
PtWRKY65-3	Potri.011G070100	807	268	2	Chr11: 6568512 - 6570537	2.5e-182
PtWRKY69-1	Potri.003G111900	933	310	2	Chr03: 13552524 - 13555165	6.9e-208
PtWRKY69-2	Potri.002G195300	738	245	3	Chr02: 15629980 - 15631893	1.8e-165
PtWRKY69-3	PtPotri.014G119800	789	262	2	Chr14: 9270774 - 9272792	1.8e-165
PtWRKY69-4	Potri.001G121300	945	314	2	Chr01: 9788352 - 9790660	8.9e-209
PtWRKY70	Potri.013G090300	975	324	2	Chr13: 9541636 - 9543313	5.3e-221
PtWRKY71-1	Potri.011G079300	921	306	2	Chr11: 7865659 - 7867200	2.4e-201
PtWRKY71-2	Potri.001G352400	939	312	2	Chr01: 36029746 - 36031662	2.7e-211

PtWRKY71-3	Potri.002G059100	978	325	2	Chr02: 3993054 - 3995674	1.4e-219
PtWRKY71-4	Potri.005G203200	969	322	2	Chr05: 21797854 - 21801101	5.9e-220
PtWRKY72-1	Potri.017G079500	1617	538	3	Chr17: 9321138 - 9323815	0.0e+00
PtWRKY72-2	Potri.015G064100	1887	628	6	Chr15: 8835584 - 8840603	0.0e+00
PtWRKY74	Potri.005G055300	1062	353	2	Chr05: 3917572 - 3920158	4.2e-241
PtWRKY75-1	Potri.T043800	561	186	1	scaffold_41: 135129 - 137486	1.4e-103
PtWRKY75-2	Potri.001G328000	804	267	2	Chr01: 33209754 - 33213251	6.0e-124
PtWRKY75-3	Potri.003G169100	474	157	1	Chr03: 17915557 - 17918119	1.4e-103
PtWRKY75-4	Potri.001G058800	483	160	1	Chr01: 4503179 - 4509102	9.1e-86
Prunus persica						
PperWRKY1	ppa004905m.g	1461	486	3	scaffold_3: 15682525 - 15685261	0.0e+00
PperWRKY2-1	ppb022557m.g	2256	751	4	scaffold_6: 28587954 - 28591793	0.0e+00
PperWRKY2-2	ppa001924m.g	2223	740	4	scaffold_6: 2468104 - 2471544	0.0e+00
PperWRKY3	ppa004312m.g	1554	517	3	scaffold_1: 27443164 - 27446022	0.0e+00
PperWRKY4	ppa003809m.g	1644	547	3	scaffold_3: 19419766 - 19423127	0.0e+00
PperWRKY5	ppa022287m.g	735	244	3	scaffold_2: 15597322 - 15600214	2.8e-167
PperWRKY6-1	ppa022758m.g	1542	513	5	scaffold_4: 13666776 - 13669306	0.0e+00
PperWRKY6-2	ppa002619m.g	1956	651	4	scaffold_3: 16460557 - 16463047	0.0e+00
PperWRKY6-3	ppa003574m.g	1695	564	6	scaffold_4: 820970 - 825131	0.0e+00
PperWRKY7-1	ppa008613m.g	978	325	2	scaffold_6: 21500388 - 21502030	2.1e-212
PperWRKY7-2	ppa007930m.g	1056	351	2	scaffold_1: 35966149 - 35967624	1.1e-234
PperWRKY9	ppa016401m.g	1686	561	4	scaffold_1: 43717207 - 43719518	0.0e+00
PperWRKY11	ppa009700m.g	846	281	2	scaffold_1: 4817218 - 4818580	1.2e-185
PperWRKY13	ppa020629m.g	696	231	2	scaffold_6: 15369732 - 15373318	5.7e-157
PperWRKY14	ppa004535m.g	1515	504	2	scaffold_4: 5152653 - 5155480	0.0e+00
PperWRKY17	ppa008846m.g	954	317	3	scaffold_1: 37472989 - 37474731	2.7e-210
PperWRKY18	ppa008566m.g	981	326	5	scaffold_1: 34009489 - 34011202	8.7e-219
PperWRKY19-1	ppa021559m.g	357	119	2	scaffold_16: 32444 - 34188	1.2e-74
PperWRKY19-2	ppa026260m.g	228	76	1	scaffold_5: 11029791 - 11030494	8.5e-44
PperWRKY20	ppa003305m.g	1761	586	5	scaffold_4: 14951801 - 14956955	0.0e+00
PperWRKY21-1	ppa021657m.g	900	299	2	scaffold_8: 19829267 - 19831289	1.1e-231
PperWRKY21-2	ppa007838m.g	1065	354	2	scaffold_6: 27763305 - 27764742	6.8e-243
PperWRKY21-3	ppa023256m.g	1023	340	3	scaffold_5: 8848352 - 8851174	1.4e-224

PperWRKY22-1	ppa007745m.g	1074	357	2	scaffold_2: 18665651 - 18667118	1.0e-240
PperWRKY22-2	ppa025013m.g	852	283	3	scaffold_5: 11272595 - 11273677	1.9e-190
PperWRKY23-1	ppa008731m.g	966	321	2	scaffold_10: 526239 - 528128	8.3e-218
PperWRKY23-2	ppa006909m.g	1173	390	2	scaffold_1: 8640398 - 8642407	1.4e-255
PperWRKY26	ppa003333m.g	1755	584	4	scaffold_6: 1828828 - 1831501	0.0e+00
PperWRKY27	ppa025042m.g	1209	402	3	scaffold_2: 25381962 - 25384144	5.1e-278
PperWRKY32	ppa004042m.g	1602	533	4	scaffold_7: 22197495 - 22201494	0.0e+00
PperWRKY33-1	ppa015765m.g	402	134	2	scaffold_5: 12459036 - 12459687	1.4e-86
PperWRKY33-2	ppa004009m.g	1608	535	4	scaffold_6: 24639609 - 24641627	0.0e+00
PperWRKY35	ppa010217m.g	777	258	2	scaffold_8: 21517865 - 21519013	1.4e-169
PperWRKY40-1	ppa010796m.g	711	236	2	scaffold_3: 6662580 - 6663950	1.1e-147
PperWRKY40-2	ppa009939m.g	816	271	3	scaffold_1: 34029320 - 34030841	3.6e-177
PperWRKY41	ppa007986m.g	1050	349	2	scaffold_2: 25613734 - 25614985	1.9e-237
PperWRKY43-1	ppa015755m.g	666	221	1	scaffold_2: 18683476 - 18685191	5.2e-149
PperWRKY43-2	ppa024540m.g	525	174	1	scaffold_2: 21972625 - 21973268	1.9e-117
PperWRKY44	ppa005152m.g	1428	475	4	scaffold_6: 22336216 - 22339034	0.0e+00
PperWRKY45	ppa012414m.g	513	170	1	scaffold_5: 13581887 - 13583455	4.0e-113
PperWRKY46	ppa007708m.g	1077	358	2	scaffold_2: 19059670 - 19062101	1.4e-242
PperWRKY47	ppa018075m.g	1704	567	4	scaffold_10: 162785 - 165574	0.0e+00
PperWRKY49	ppa019550m.g	900	299	2	scaffold_6: 23123896 - 23125761	2.6e-201
PperWRKY51	ppa015480m.g	591	196	2	scaffold_6: 15635236 - 15637392	3.9e-132
PperWRKY53	ppa008573m.g	981	326	3	scaffold_5: 11878403 - 11880061	7.3e-220
PperWRKY55	ppa014826m.g	1047	348	4	scaffold_2: 23638750 - 23643347	9.0e-238
PperWRKY57	ppa008480m.g	993	330	3	scaffold_1: 27581955 - 27585622	3.4e-225
PperWRKY61-2	ppa017919m.g	1602	533	2	scaffold_1: 26674923 - 26676963	0.0e+00
PperWRKY61-1	ppa021195m.g	1941	646	3	scaffold_5: 15492412 - 15495700	0.0e+00
PperWRKY65	ppa009647m.g	852	283	2	scaffold_4: 3206039 - 3207150	1.1e-189
PperWRKY69	ppa025586m.g	834	277	3	scaffold_3: 8609429 - 8613217	1.1e-186
PperWRKY70-1	ppa025912m.g	1053	350	2	scaffold_6: 25157893 - 25159753	2.2e-244
PperWRKY70-2	ppa026704m.g	861	286	3	scaffold_6: 25167296 - 25170022	3.2e-197
PperWRKY70-3	ppa020736m.g	1008	335	3	scaffold_6: 25162460 - 25164336	4.8e-222
PperWRKY70-4	ppa026125m.g	972	323	2	scaffold_2: 23642489 - 23644464	4.8e-222
PperWRKY71-1	ppa026831m.g	1083	360	2	scaffold_3: 13803400 - 13805785	3.5e-245

PperWRKY71-2	ppa024027m.g	1014	337	2	scaffold_4: 3695838 - 3697364	2.2e-229
PperWRKY72	ppa020780m.g	1446	481	3	scaffold_3: 19839824 - 19842047	0.0e+00
PperWRKY75-1	ppa016153m.g	558	185	1	scaffold_1: 22733682 - 22735582	1.2e-123
PperWRKY75-2	ppa016459m.g	672	223	1	scaffold_3: 21511993 - 21513894	2.6e-151
Ricinus communis						
RcWRKY1	29949.t000007	1455	484	3	29949: 50158 - 52013	0.0e+00
RcWRKY3	30174.t000563	1407	468	3	30174: 3380314 - 3384496	9.8e-316
RcWRKY5	29709.t000007	558	185	3	29709: 41452 - 43070	8.4e-122
RcWRKY6	30064.t000028	1680	559	5	30064: 203340 - 205783	0.0e+00
RcWRKY7-1	30170.t000244	1134	377	2	30170: 1224146 - 1225662	1.0e-250
RcWRKY7-2	29644.t000015	318	105	1	29644: 79578 - 80028	3.9e-67
RcWRKY9	29990.t000001	1599	532	4	29990: 4397 - 6433	0.0e+00
RcWRKY13	28040.t000001	311	103	1	28040: 28206 - 31334	3.3e-67
RcWRKY17	29848.t000020	966	321	2	29848: 94812 - 96608	2.2e-212
RcWRKY18-1	30131.t000001	519	172	1	30131: 4672 - 6497	6.7e-108
RcWRKY18-2	29848.t000100	420	139	2	29848: 522197 - 523071	3.8e-90
RcWRKY20	27613.t000032	1689	562	5	27613: 207515 - 213650	0.0e+00
RcWRKY21-1	29883.t000063	1062	353	2	29883: 450119 - 452078	1.1e-237
RcWRKY21-2	29598.t000004	792	263	3	29598: 22752 - 24172	2.7e-174
RcWRKY22	30174.t000060	1044	347	2	30174: 2058419 - 2060079	5.5e-236
RcWRKY23	30076.t000187	954	317	2	30076: 1244284 - 1245583	4.1e-212
RcWRKY26	29820.t000050	1677	558	4	29820: 291661 - 294372	0.0e+00
RcWRKY27	30169.t000358	1380	459	2	30169: 1998907 - 2000467	0.0e+00
RcWRKY29	30190.t000512	1005	334	2	30190: 3016527 - 3017971	3.4e-222
RcWRKY30-1	28690.t000001	927	308	2	28690: 12224 - 13680	1.4e-226
RcWRKY30-2	30174.t000048	1002	333	2	30174: 1952662 - 1954347	2.1e-184
RcWRKY31	29842.t000052	1743	580	5	29842: 273087 - 275527	0.0e+00
RcWRKY32-1	29687.t000003	1512	503	5	29687: 15684 - 21009	0.0e+00
RcWRKY32-2	29848.t000095	1119	372	2	29848: 492201 - 493883	1.5e-248
RcWRKY33	29717.t000002	1728	575	4	29717: 10576 - 13600	0.0e+00
RcWRKY34-1	29929.t000090	1830	609	4	29929: 510214 - 512861	0.0e+00
RcWRKY34-2	28966.t000003	2202	733	4	28966: 23998 - 27612	0.0e+00
RcWRKY35-1	30076.t000212	798	265	2	30076: 1383612 - 1384793	9.8e-316

RcWRKY35-2	27996.t000002	1401	466	4	27996: 7134 - 9912	4.2e-177
RcWRKY40	29848.t000101	636	211	3	29848: 537211 - 538602	1.5e-134
RcWRKY41	30169.t000310	1113	370	2	30169: 1742567 - 1744656	6.6e-251
RcWRKY42	30010.t000025	1959	652	4	30010: 384306 - 386731	0.0e+00
RcWRKY43-1	30174.t000066	579	192	1	30174: 2076040 - 2077754	3.8e-129
RcWRKY43-2	30190.t000514	558	185	1	30190: 3026476 - 3027219	7.8e-124
RcWRKY44	29805.t000035	1425	474	4	29805: 189773 - 191900	0.0e+00
RcWRKY45	30147.t000745	495	164	1	30147: 2203761 - 2204393	1.2e-106
RcWRKY47	30076.t000112	1497	498	5	30076: 678694 - 681282	0.0e+00
RcWRKY48	29889.t000087	1056	351	2	29889: 412384 - 414133	1.4e-232
RcWRKY49	29767.t000010	891	296	1	29767: 163654 - 166469	2.3e-199
RcWRKY50	29739.t000022	480	159	2	29739: 136858 - 137722	3.3e-106
RcWRKY51-1	29771.t000001	648	215	2	29771: 4111 - 8719	2.8e-147
RcWRKY51-2	28644.t000022	507	168	2	28644: 112321 - 114401	7.6e-113
RcWRKY53	30190.t000050	1017	338	2	30190: 3507055 - 3508463	1.8e-229
RcWRKY54	29729.t000102	945	314	2	29729: 563671 - 564936	6.9e-215
RcWRKY55-2	29729.t000063	957	318	2	29729: 342169 - 344149	5.8e-217
RcWRKY55-1	29729.t000103	996	331	2	29729: 568893 - 570667	7.6e-113
RcWRKY57	30174.t000532	942	313	2	30174: 3221434 - 3224608	2.0e-216
RcWRKY59	28455.t000009	1104	367	3	28455: 134590 - 136512	2.5e-247
RcWRKY60	43951.t000001	586	195	2	43951: 31 - 916	2.2e-124
RcWRKY61	29736.t000019	1689	562	5	29736: 182805 - 186919	0.0e+00
RcWRKY65	30026.t000025	804	267	2	30026: 178343 - 179952	1.2e-180
RcWRKY70	29915.t000015	993	330	2	29915: 141304 - 143043	6.4e-221
RcWRKY71	29693.t000098	933	330	2	29693: 676301 - 677606	2.1e-211
RcWRKY72-1	29822.t000159	1683	560	4	29822: 915514 - 919280	0.0e+00
RcWRKY72-2	30147.t000358	1956	651	5	30147: 4428301 - 4432024	0.0e+00
RcWRKY75-1	29929.t000127	612	203	1	29929: 718638 - 720485	1.9e-131
RcWRKY75-2	30190.t000144	585	194	1	30190: 612076 - 613585	2.3e-128
Selaginella moellendorffii						
SmWRKY2	56793	861	287	4	scaffold_39: 1056465 - 1057945	1.5e-195
SmWRKY6	29146	318	106	0	scaffold_1: 5239472 - 5239789	1.6e-65
SmWRKY7-1	73225	153	51	1	scaffold_14: 996802 - 997039	1.7e-29

SmWRKY7-2	147026	876	291	3	scaffold_14: 1116310 - 1117972	6.0e-195
SmWRKY8	69423	261	87	1	scaffold_8: 478098 - 478423	1.1e-61
SmWRKY9	81371	186	62	1	scaffold_4: 2643379 - 2643634	3.3e-43
SmWRKY15	108669	186	62	1	scaffold_37: 849679 - 849932	4.4e-38
SmWRKY21	73221	177	59	1	scaffold_14: 1266414 - 1266697	1.5e-36
SmWRKY26	66769	813	271	4	scaffold_11: 2777016 - 2778560	2.3e-181
SmWRK28	69431	261	87	1	scaffold_0: 4893341 - 4893667	1.1e-61
SmWRK33	24407	777	259	2	scaffold_4: 1503216 - 1504146	7.6e-175
SmWRKY34	125080	603	200	2	scaffold_77: 184904 - 185758	8.7e-135
SmWRK35	77979	285	95	1	scaffold_2: 1361130 - 1361619	4.6e-63
SmWRKY39	73220	177	59	1	scaffold_4: 206074 - 206417	5.1e-37
SmWRK42	92733	378	126	1	scaffold_13: 1393034 - 1393523	1.4e-82
SmWRKY48	69405	246	82	1	scaffold_4: 2918228 - 2918710	4.2e-57
SmWRKY54	29692	201	67	1	scaffold_1: 207818 - 208376	4.3e-45
SmWRKY55	29703	192	64	1	scaffold_5: 3733269 - 3733528	8.8e-47
SmWRKY57	29247	240	80	1	scaffold_19: 680055 - 680520	3.0e-65
Setaria italica						
SiWRKY2	Si038537m.g	1266	421	1	scaffold_9: 35337925 - 35339264	7.8e-285
SiWRKY3	Si036692m.g	951	316	3	scaffold_9: 5125810 - 5129451	8.7e-205
SiWRKY4	Si034188m.g	2628	875	10	scaffold_9: 5055630 - 5061547	0.0e+00
SiWRKY5	Si022468m.g	1077	358	2	scaffold_3: 3470860 - 3472976	1.3e-229
SiWRKY6	Si040171m.g	1209	402	2	scaffold_9: 3108737 - 3110314	5.6e-264
SiWRKY7	Si023194m.g	663	220	2	scaffold_3: 12073118 - 12074773	3.9e-140
SiWRKY8	Si022568m.g	1032	343	1	scaffold_3: 9086074 - 9088318	1.1e-231
SiWRKY9	Si000824m.g	1725	574	5	scaffold_5: 3128175 - 3133163	0.0e+00
SiWRKY10	Si002621m.g	774	257	2	scaffold_5: 11385866 - 11387183	2.8e-173
SiWRKY11	Si002019m.g	1068	355	2	scaffold_5: 29626945 - 29629320	2.9e-244
SiWRKY12	Si001863m.g	1137	378	2	scaffold_5: 29517728 - 29519669	2.6e-247
SiWRKY13	Si002364m.g	918	305	2	scaffold_5: 36906836 - 36908901	7.8e-199
SiWRKY14	Si002365m.g	918	305	1	scaffold_5: 35946493 - 35948069	1.7e-201
SiWRKY15	Si002203m.g	987	328	2	scaffold_5: 31163423 - 31164962	9.7e-219
SiWRKY16-1	Si001816m.g	1158	385	2	scaffold_5: 32436584 - 32439385	5.6e-261
SiWRKY16-2	Si024841m.g	531	177	1	scaffold_3: 3216727 - 3217377	1.8e-115

SiWRKY17	Si005025m.g	1143	380	2	scaffold_5: 47112340 - 47113983	8.0e-252
SiWRKY18	Si005138m.g	774	257	2	scaffold_5: 40034269 - 40038719	1.8e-177
SiWRKY19	Si022843m.g	882	293	2	scaffold_3: 9758402 - 9760416	6.7e-199
SiWRKY20	Si003782m.g	999	332	2	scaffold_5: 40040883 - 40043080	3.2e-227
SiWRKY21	Si002401m.g	897	298	1	scaffold_5: 40062376 - 40063545	1.7e-204
SiWRKY22	Si004791m.g	693	230	2	scaffold_5: 40023634 - 40027402	3.4e-159
SiWRKY23-1	Si002751m.g	714	237	1	scaffold_5: 36074399 - 36077406	2.6e-160
SiWRKY23-2	Si025086m.g	705	234	1	scaffold_3: 12588817 - 12589663	1.3e-155
SiWRKY24-1	Si000906m.g	1659	552	4	scaffold_5: 40361245 - 40363952	0.0e+00
SiWRKY24-2	Si026466m.g	1188	395	3	scaffold_8: 10797932 - 10800694	6.9e-272
SiWRKY25	Si014163m.g	897	298	0	scaffold_6: 1207862 - 1209078	2.4e-190
SiWRKY26	Si004464m.g	657	218	2	scaffold_5: 35093336 - 35094332	1.4e-147
SiWRKY27	Si002295m.g	954	317	1	scaffold_5: 31804633 - 31805848	3.1e-210
SiWRKY28	Si006914m.g	969	322	2	scaffold_4: 31570795 - 31572497	3.4e-210
SiWRKY29	Si030377m.g	1038	345	1	scaffold_2: 189686 - 191781	9.6e-230
SiWRKY30	Si013374m.g	2010	669	6	scaffold_6: 31988939 - 31992934	0.0e+00
SiWRKY31-1	Si008820m.g	1032	343	1	scaffold_4: 25418195 - 25419340	1.4e-230
SiWRKY31-2	Si008215m.g	966	322	2	scaffold_4: 23966320 - 23967446	6.3e-221
SiWRKY32	Si016791m.g	1695	564	4	scaffold_1: 39238452 - 39240738	0.0e+00
SiWRKY34	Si018283m.g	675	224	3	scaffold_1: 33079967 - 33084064	4.3e-157
SiWRKY35	Si012785m.g	2037	678	4	scaffold_7: 22557955 - 22561463	0.0e+00
SiWRKY36	Si030985m.g	711	236	3	scaffold_2: 22792365 - 22795907	1.0e-162
SiWRKY37	Si011735m.g	1419	472	2	scaffold_7: 28832235 - 28835104	0.0e+00
SiWRKY39	Si017529m.g	1137	378	2	scaffold_1: 747341 - 748929	3.1e-252
SiWRKY40	Si019098m.g	993	330	2	scaffold_1: 3503350 - 3504664	1.0e-230
SiWRKY42	Si017889m.g	924	307	2	scaffold_1: 21363712 - 21365226	4.8e-201
SiWRKY43	Si024078m.g	1734	577	5	scaffold_3: 10070045 - 10072439	0.0e+00
SiWRKY45	Si022702m.g	951	316	2	scaffold_3: 27173841 - 27175564	4.8e-208
SiWRKY46-1	Si011007m.g	672	223	2	scaffold_7: 34178729 - 34181807	4.8e-150
SiWRKY46-2	Si026831m.g	672	223	2	scaffold_8: 746745 - 749572	9.5e-150
SiWRKY47	Si030395m.g	1032	343	2	scaffold_2: 48387967 - 48389788	7.3e-225
SiWRKY49	Si022220m.g	1215	404	2	scaffold_3: 10306902 - 10309504	1.5e-274
SiWRKY51	Si012025m.g	1098	365	2	scaffold_7: 13130951 - 13132232	2.9e-243

SiWRKY52	Si010706m.g	900	299	1	scaffold_7: 34171483 - 34172498	7.3e-203
SiWRKY53	Si021956m.g	1410	469	1	scaffold_3: 26202837 - 26205056	0.0e+00
SiWRKY54-1	Si027574m.g	738	246	2	scaffold_8: 33523376 - 33524318	5.9e-167
SiWRKY54-2	Si024816m.g	624	208	2	scaffold_3: 15583218 - 15584065	3.8e-139
SiWRKY55	Si039519m.g	654	217	2	scaffold_9: 47763258 - 47764313	4.6e-145
SiWRKY57	Si014939m.g	534	177	1	scaffold_6: 33364162 - 33364874	4.1e-122
SiWRKY59-1	Si028710m.g	3873	1290	5	scaffold_2: 26415481 - 26421105	0.0e+00
SiWRKY59-2	Si013326m.g	2199	732	5	scaffold_6: 2354633 - 2359056	0.0e+00
SiWRKY60-1	Si038955m.g	282	93	1	scaffold_9: 9759533 - 9761173	5.9e-60
SiWRKY60-2	Si038001m.g	399	132	2	scaffold_9: 9767027 - 9768526	2.9e-83
SiWRKY62	Si030794m.g	813	270	2	scaffold_2: 30748506 - 30749602	9.6e-177
SiWRKY64-1	Si010764m.g	864	287	2	scaffold_7: 34194819 - 34196066	3.7e-193
SiWRKY64-2	Si028202m.g	876	291	2	scaffold_8: 12982860 - 12983901	2.1e-202
SiWRKY65	Si010813m.g	825	274	2	scaffold_7: 34202591 - 34204306	2.7e-185
SiWRKY66	Si017095m.g	1425	474	2	scaffold_1: 35418955 - 35422712	0.0e+00
SiWRKY67	Si023279m.g	612	203	2	scaffold_3: 7209438 - 7210943	4.8e-132
SiWRKY68	Si010545m.g	1014	337	3	scaffold_7: 29191315 - 29193248	1.6e-215
SiWRKY69	Si015433m.g	1035	344	1	scaffold_6: 23660441 - 23661481	1.3e-230
SiWRKY70	Si021634m.g	1689	562	3	scaffold_3: 16100904 - 16103618	0.0e+00
SiWRKY71	Si017443m.g	1188	395	2	scaffold_1: 5877131 - 5879314	3.0e-261
SiWRKY72	Si026859m.g	624	207	1	scaffold_8: 23524892 - 23527940	3.0e-138
SiWRKY73	Si006217m.g	1602	533	2	scaffold_4: 2226437 - 2230067	0.0e+00
SiWRKY74-1	Si030528m.g	975	324	1	scaffold_2: 27231842 - 27233604	2.8e-216
SiWRKY74-2	Si030531m.g	972	323	2	scaffold_2: 27207918 - 27209792	1.1e-213
SiWRKY76	Si030429m.g	1014	337	2	scaffold_2: 30729585 - 30731008	1.1e-220
SiWRKY77	Si002862m.g	657	218	1	scaffold_5: 31907150 - 31908059	1.3e-144
SiWRKY78	Si029245m.g	1842	613	5	scaffold_2: 44372289 - 44377436	0.0e+00
SiWRKY79	Si036565m.g	993	330	2	scaffold_9: 47109496 - 47110923	2.4e-214
SiWRKY80	Si029342m.g	1722	573	5	scaffold_2: 35162754 - 35166321	0.0e+00
SiWRKY81-1	Si026670m.g	930	309	5	scaffold_8: 762199 - 763393	4.1e-210
SiWRKY81-2	Si027328m.g	843	280	2	scaffold_8: 12912885 - 12914076	1.4e-192
SiWRKY81-3	Si027769m.g	873	290	2	scaffold_8: 12966027 - 12967302	2.7e-200
SiWRKY81-4	Si027998m.g	873	290	2	scaffold_8: 12959461 - 12960684	5.3e-200

SiWRKY81-5	Si027946m.g	912	303	2	scaffold_8: 12904286 - 12905548	5.4e-210
SiWRKY81-6	Si028270m.g	876	291	3	scaffold_8: 12997035 - 12998335	2.9e-199
SiWRKY82-1	Si013488m.g	1722	573	3	scaffold_6: 12823975 - 12830187	0.0e+00
SiWRKY82-2	Si027876m.g	753	250	2	scaffold_8: 725949 - 727164	2.2e-168
SiWRKY83-1	Si022461m.g	1077	358	3	scaffold_3: 47920504 - 47923359	6.0e-238
SiWRKY83-2	Si036408m.g	1050	349	3	scaffold_9: 10990005 - 10993009	8.8e-234
SiWRKY85	Si021859m.g	1485	494	3	scaffold_3: 45536398 - 45541486	0.0e+00
SiWRKY86	Si002121m.g	1017	338	2	scaffold_5: 40052992 - 40055657	1.4e-224
SiWRKY87	Si036039m.g	1185	394	3	scaffold_9: 5904751 - 5907896	1.8e-262
SiWRKY88	Si038727m.g	1122	373	2	scaffold_9: 322262 - 326601	7.1e-254
SiWRKY90-1	Si025181m.g	810	270	3	scaffold_3: 5270636 - 5272093	1.3e-183
SiWRKY90-2	Si031470m.g	414	137	5	scaffold_2: 6029825 - 6031654	1.6e-89
SiWRKY91	Si022933m.g	828	275	2	scaffold_3: 15637590 - 15640378	1.2e-183
SiWRKY92	Si022676m.g	966	321	2	scaffold_3: 9059875 - 9061197	1.1e-216
SiWRKY93	Si008812m.g	1080	359	2	scaffold_4: 5139966 - 5142417	2.4e-244
SiWRKY95	Si025307m.g	1113	370	2	scaffold_3: 27182439 - 27185445	1.7e-250
SiWRKY96	Si035317m.g	1506	501	3	scaffold_9: 41983508 - 41991013	0.0e+00
SiWRKY97	Si001109m.g	1527	508	3	scaffold_5: 11382193 - 11384494	0.0e+00
SiWRKY100	Si010832m.g	816	271	2	scaffold_7: 34186596 - 34187853	7.4e-185
SiWRKY101	Si012735m.g	771	256	2	scaffold_7: 21739485 - 21741091	1.2e-179
SiWRKY102	Si003805m.g	627	209	1	scaffold_5: 11112146 - 11112866	3.4e-135
SiWRKY103	Si002957m.g	618	205	1	scaffold_5: 41194496 - 41195255	1.1e-136
SiWRKY104	Si027360m.g	738	245	2	scaffold_8: 736074 - 737116	2.3e-167
SiWRKY106	Si003876m.g	516	171	0	scaffold_5: 41188553 - 41189068	3.8e-112
Solanum lycopersicum						
SlWRKY1-1	Solyc07g047960.2	1242	413	4	chr07: 56499981 - 56504055	3.3e-272
SlWRKY1-2	Solyc12g006170.1	1248	415	3	chr12: 712968 - 715497	2.2e-277
SlWRKY2-1	Solyc07g066220.2	2220	739	5	chr07: 64918419 - 64923896	0.0e+00
SlWRKY2-2	Solyc10g005680.1	2112	703	4	chr10: 551292 - 554630	0.0e+00
SlWRKY3	Solyc02g088340.2	1383	460	3	chr02: 45045827 - 45048579	2.0e-315
SlWRKY4-1	Solyc03g104810.2	1461	486	3	chr03: 54885728 - 54891292	0.0e+00
SlWRKY4-2	Solyc05g012770.2	1527	508	3	chr05: 5928753 - 5934454	0.0e+00
SlWRKY5	Solyc01g089960.2	711	236	3	chr01: 75418839 - 75424413	5.2e-159

SlWRKY6-1	Solyc02g080890.2	1653	550	5	chr02: 39569094 - 39571649	0.0e+00
SlWRKY6-2	Solyc02g032950.2	1497	498	4	chr02: 20198127 - 20200157	0.0e+00
SlWRKY7	Solyc04g078550.2	1056	351	3	chr04: 60852591 - 60854416	1.1e-230
SlWRKY9	Solyc01g104550.2	1428	475	4	chr01: 84779786 - 84782183	1.3e-312
SlWRKY11	Solyc12g096350.1	1017	338	5	chr12: 63654463 - 63655829	2.8e-223
SlWRKY13	Solyc04g051540.2	705	234	2	chr04: 49935987 - 49940182	1.2e-158
SlWRKY14	Solyc03g082810.1	660	219	0	ch03: 46218868 - 46219527	1.8e-148
SlWRKY15	Solyc02g093050.2	981	326	4	chr02: 48575427 - 48577339	9.8e-212
SlWRKY17	Solyc08g006320.2	987	328	2	chr08: 985013 - 986480	2.8e-217
SlWRKY18	Solyc08g067360.2	777	258	3	chr08: 53615717 - 53617791	9.6e-170
SlWRKY20-1	Solyc03g082750.1	537	178	0	chr03: 46183274 - 46183810	0.0e+00
SlWRKY20-2	Solyc12g014610.1	1836	611	5	chr12: 5610112 - 5616122	0.0e+00
SlWRKY20-3	Solyc07g065260.2	1791	596	5	chr07: 64315902 - 64321013	0.0e+00
SlWRKY21-1	Solyc09g066010.2	996	331	2	chr09: 59882612 - 59884663	9.9e-222
SlWRKY21-2	Solyc06g008610.2	1047	348	2	chr06: 2512503 - 2514892	1.2e-234
SlWRKY22-1	Solyc05g045800.1	762	253	2	chr05: 56734007 - 56735123	9.9e-222
SlWRKY22-2	Solyc01g095100.2	1068	355	2	chr01: 78259686 - 78261279	1.6e-242
SlWRKY22-3	Solyc10g011910.2	936	311	2	chr10: 4187405 - 4189346	5.6e-212
SlWRKY23	Solyc01g079260.2	963	320	2	chr01: 70866242 - 70868698	3.6e-219
SlWRKY26	Solyc06g066370.2	1608	535	4	chr06: 38062111 - 38065102	0.0e+00
SlWRKY27-1	Solyc03g007640.1	1080	359	2	chr03: 2179022 - 2180281	4.7e-235
SlWRKY27-2	Solyc04g050210.1	1101	366	2	chr04: 45295989 - 45297316	8.8e-249
SlWRKY27-3	Solyc05g050060.1	969	322	2	chr05: 59189414 - 59190607	1.2e-216
SlWRKY27-3	Solyc05g050050.1	969	322	2	chr05: 59132157 - 59133345	1.2e-216
SlWRKY27-4	Solyc05g045880.1	966	321	2	chr05: 56755323 - 56756511	3.7e-217
SlWRKY27-5	Solyc05g050040.1	837	278	2	chr05: 59125683 - 59126804	9.2e-187
SlWRKY27-6	Solyc05g045710.1	717	238	2	chr05: 56704002 - 56705190	2.1e-160
SlWRKY28-1	Solyc07g056280.2	969	322	2	chr07: 61460392 - 61463054	2.8e-221
SlWRKY28-2	Solyc12g011200.1	1008	335	2	chr12: 4045306 - 4047487	6.7e-228
SlWRKY29	Solyc08g081610.2	912	303	2	chr08: 61773941 - 61776031	2.7e-204
SlWRKY30	Solyc03g007380.1	1062	353	2	chr03: 1929376 - 1930601	2.8e-240
SlWRKY31	Solyc07g051840.2	1896	631	4	chr07: 57685533 - 57688827	0.0e+00
SlWRKY32	Solyc07g005650.2	1542	513	4	chr07: 518591 - 524775	0.0e+00

SlWRKY34-1	Solyc05g055750.2	1380	459	4	chr05: 64410352 - 64413767	7.8e-315
SlWRKY34-2	Solyc04g056360.2	1206	401	2	chr04: 53281612 - 53283583	1.1e-273
SlWRKY35-1	Solyc02g021680.2	1146	381	2	chr02: 14206227 - 14209227	7.6e-260
SlWRKY35-2	Solyc01g079360.2	723	240	2	chr01: 70960474 - 70962139	2.9e-159
SlWRKY40-1	Solyc03g116890.2	1053	350	4	chr03: 60192911 - 60195340	3.0e-224
SlWRKY40-2	Solyc06g068460.2	1083	360	3	chr06: 38823797 - 38825771	1.6e-234
SlWRKY41-1	Solyc10g009550.2	873	290	2	chr10: 3672838 - 3674913	1.6e-196
SlWRKY41-2	Solyc05g050330.2	735	244	2	chr05: 59596882 - 59598610	1.8e-162
SlWRKY41-3	Solyc05g050340.2	984	327	2	chr05: 59599095 - 59601281	6.5e-223
SlWRKY41-4	Solyc05g050300.1	588	195	1	chr05: 59581810 - 59582488	3.5e-131
SlWRKY43	Solyc08g081630.1	696	231	1	chr08: 61787281 – 61789385	6.2e-158
SlWRKY44	Solyc10g084380.1	1269	422	4	chr10: 63276460 - 63279065	8.9e-284
SlWRKY47	Solyc05g007110.2	1179	392	4	chr05: 1679764 - 1683310	3.3e-260
SlWRKY48	Solyc01g058540.2	975	324	2	chr01: 59717621 - 59719154	2.1e-215
SlWRKY49	Solyc09g010960.2	873	290	2	chr09: 4315614 - 4317328	4.1e-198
SlWRKY50	Solyc08g062490.2	546	181	2	chr08: 48364804 - 48366524	1.5e-120
SlWRKY51	Solyc04g051690.2	525	174	2	chr04: 50163412 - 50166584	4.6e-116
SlWRKY53-1	Solyc08g082110.2	1020	339	2	chr08: 62164391 - 62166398	2.0e-232
SlWRKY53-2	Solyc01g095630.2	1011	336	2	chr01: 78600553 - 78602865	8.7e-222
SlWRKY53-3	Solyc08g008280.2	1083	360	2	chr08: 2704613 - 2706561	5.9e-249
SlWRKY54	Solyc03g095770.2	822	273	2	chr03: 50421318 - 50423492	9.2e-186
SlWRKY55	Solyc06g048870.1	732	243	3	chr06: 28334966 - 28338066	5.7e-168
SlWRKY57	Solyc05g012500.2	984	327	2	chr05: 5742002 - 5745384	8.8e-221
SlWRKY59-1	Solyc12g056750.1	567	188	2	chr12: 48196449 - 48199052	1.3e-123
SlWRKY59-2	Solyc04g072070.2	687	228	2	chr04: 56722272 - 56725055	8.5e-150
SlWRKY60-1	Solyc08g067340.2	762	253	3	chr08: 53602606 - 53604908	6.1e-166
SlWRKY60-2	Solyc12g042590.1	651	216	3	chr12: 43390294 - 43391728	2.4e-142
SlWRKY61	Solyc03g113120.2	1575	524	4	chr03: 57442051 - 57446231	0.0e+00
SlWRKY65-1	Solyc07g055280.2	828	275	2	chr07: 60692504 - 60696127	1.6e-176
SlWRKY65-2	Solyc10g007970.1	768	255	2	chr10: 2131640 - 2134481	1.6e-176
SlWRKY68	Solyc05g053380.2	888	295	2	chr05: 62631053 - 62633674	9.9e-202
SlWRKY69	Solyc02g072190.2	927	308	2	chr02: 36084177 - 36085943	4.6e-205
SlWRKY70	Solyc09g015770.2	876	291	2	chr09: 10987958 - 10989890	9.0e-195

SlWRKY71	Solyc02g071130.2	954	317	2	chr02: 35189131 - 35190735	9.7e-215
SlWRKY72-1	Solyc06g070990.2	1950	649	5	chr06: 39985716 - 39990128	0.0e+00
SlWRKY72-2	Solyc02g067430.2	1323	440	2	chr02: 32190832 - 32193017	2.7e-288
SlWRKY75-1	Solyc05g015850.2	519	172	1	chr05: 12420457 - 12423158	9.2e-113
SlWRKY75-2	Solyc02g094270.1	396	131	1	chr02: 49426008 - 49428482	1.4e-87
Solanum tuberosum						
StWRKY1-1	PGSC0003DMG400006155	1269	422	3	chr07: 41918631 - 41923738	4.0e-279
StWRKY1-2	PGSC0003DMG402028822	1650	549	3	chr12: 6096237 - 6102945	0.0e+00
StWRKY2	PGSC0003DMG400011271	2094	697	4	chr10: 681772 - 685968	0.0e+00
StWRKY3	PGSC0003DMG401005575	1551	516	3	chr03: 31865504 - 31871538	0.0e+00
StWRKY4-1	PGSC0003DMG400001434	1356	451	3	chr02: 67174898 - 67177768	1.2e-311
StWRKY4-2	PGSC0003DMG400023360	1374	457	2	chr05: 60392669 - 60398080	9.4e-315
StWRKY5	PGSC0003DMG400031175	756	251	3	chr01: 75161638 - 75167283	2.8e-167
StWRKY6	PGSC0003DMG400015015	1674	557	4	chr07: 43494519 - 43497505	0.0e+00
StWRKY7-1	PGSC0003DMG400024961	975	324	2	chr02: 64674891 - 64676420	8.3e-211
StWRKY7-2	PGSC0003DMG400007947	1065	354	2	chr04: 59952962 - 59954622	1.5e-233
StWRKY8	PGSC0003DMG400007788	1005	334	2	chr12: 1160881 - 1163790	1.8e-227
StWRKY9	PGSC0003DMG400029779	1242	413	3	chr01: 87440155 - 87442186	4.1e-276
StWRKY11	PGSC0003DMG400029371	939	312	2	chr12: 64688961 - 64690398	2.0e-206
StWRKY12	PGSC0003DMG400044842	780	259	0	chr03: 16851119 - 16851898	1.9e-176
StWRKY13	PGSC0003DMG400019706	705	234	2	chr04: 52346557 - 52351197	8.1e-161
StWRKY14	PGSC0003DMG400015104	1242	413	2	chr02: 36259986 - 36263383	4.1e-280
StWRKY15	PGSC0003DMG400025481	1005	334	2	chr02: 45238525 - 45241113	2.1e-223
StWRKY16	PGSC0003DMG400045376	702	233	0	chr03: 16845755 - 16846456	2.9e-156
StWRKY17	PGSC0003DMG400009530	1005	334	2	chr08: 2675828 - 2677486	1.0e-222
StWRKY18-1	PGSC0003DMG400028520	1083	360	3	chr06: 43630467 - 43632582	2.9e-235
StWRKY18-2	PGSC0003DMG400007387	786	261	3	chr08: 29124121 - 29126305	6.2e-173
StWRKY20-1	PGSC0003DMG400022290	1791	596	5	chr07: 52836856 - 52842040	0.0e+00
StWRKY20-2	PGSC0003DMG400029815	1836	611	5	chr12: 8679675 - 8687286	0.0e+00
StWRKY21-1	PGSC0003DMG400015076	1041	346	2	chr09: 38659728 - 38662245	4.2e-234
StWRKY21-2	PGSC0003DMG400005329	1068	355	2	chr06: 4245698 – 4248974	5.5e-239
StWRKY22-1	PGSC0003DMG400000064	1059	352	2	chr01: 78848764 - 78850536	1.5e-240
StWRKY22-2	PGSC0003DMG400038269	645	214	2	chr04: 39779776 - 39780969	1.5e-142

StWRKY26	PGSC0003DMG400016769	1605	534	4	chr06: 42447361 - 42450142	0.0e+00
StWRKY27-1	PGSC0003DMG400036639	993	330	2	chr05: 51809555 - 51810777	2.0e-222
StWRKY27-2	PGSC0003DMG400035855	993	330	2	chr05: 51817590 - 51818812	2.9e-223
StWRKY27-3	PGSC0003DMG400041197	750	249	2	chr03: 15985048 - 15986023	4.1e-165
StWRKY27-4	PGSC0003DMG400040494	936	311	4	chr04: 39571915 - 39577071	1.5e-212
StWRKY28	PGSC0003DMG400017349	960	319	2	chr07: 47853245 - 47856367	9.1e-219
StWRKY29-1	PGSC0003DMG400039175	750	249	2	chr03: 15899673 - 15900656	1.6e-164
StWRKY29-2	PGSC0003DMG400037700	996	331	2	chr12: 5883133 - 5884370	1.3e-206
StWRKY29-3	PGSC0003DMG400012318	924	307	2	chr08: 42905115 - 42907300	5.9e-224
StWRKY31	PGSC0003DMG400016441	1662	553	5	chr02: 57852200 - 57854704	0.0e+00
StWRKY32	PGSC0003DMG400011186	369	122	0	chr07: 1538218 - 1538879	8.5e-80
StWRKY33	PGSC0003DMG400011633	1227	408	3	chr09: 27794760 - 27797875	2.4e-271
StWRKY34	PGSC0003DMG400022143	2247	748	4	chr07: 52032900 - 52037442	0.0e+00
StWRKY35	PGSC0003DMG400009014	735	244	2	chr01: 68720632 - 68722409	2.7e-161
StWRKY39	PGSC0003DMG401010558	627	208	2	chr10: 3135795 - 3140230	1.6e-143
StWRKY40	PGSC0003DMG400028633	654	217	3	chr12: 55913517 - 55914954	3.6e-142
StWRKY41-1	PGSC0003DMG400017990	906	301	2	chr05: 53958114 - 53961872	7.3e-204
StWRKY41-2	PGSC0003DMG402033880	1326	441	7	chr05: 53943622 - 53952689	2.0e-167
StWRKY41-3	PGSC0003DMG400009103	1071	356	2	chr03: 17726994 - 17728611	4.4e-242
StWRKY41-4	PGSC0003DMG400000211	1041	346	2	chr01: 79359969 - 79362180	2.2e-228
StWRKY42	PGSC0003DMG400022063	1491	496	4	chr02: 39401006 - 39403181	0.0e+00
StWRKY43	PGSC0003DMG400012317	687	228	1	chr08: 42891003 - 42893524	7.0e-155
StWRKY44	PGSC0003DMG400010987	1404	467	4	chr10: 47528890 - 47532355	5.8e-313
StWRKY45	PGSC0003DMG400008188	387	128	1	chr08: 19161502 - 19162198	5.2e-82
StWRKY48-1	PGSC0003DMG400027208	753	250	2	chr05: 57842027 - 57843473	1.7e-169
StWRKY48-2	PGSC0003DMG400011457	1020	339	2	chr01: 31481715 - 31483627	5.2e-225
StWRKY50	PGSC0003DMG401031196	417	138	2	chr04: 45568942 - 45572943	1.5e-92
StWRKY52	PGSC0003DMG400034476	522	173	0	chr03: 16847573 - 16848094	1.3e-114
StWRKY53-1	PGSC0003DMG400005836	918	305	2	chr08: 5076176 - 5080344	1.5e-204
StWRKY53-2	PGSC0003DMG400012160	1020	339	2	chr08: 42432757 - 42435667	5.4e-233
StWRKY53-3	PGSC0003DMG400005835	1083	360	2	chr08: 5091167 - 5093192	3.5e-249
StWRKY54-1	PGSC0003DMG400029207	882	293	2	chr09: 18097627 - 18099365	3.1e-198
StWRKY54-2	PGSC0003DMG401033880	996	331	2	chr05: 53952944 - 53955543	1.0e-226

StWRKY57	PGSC0003DMG400028381	981	326	2	chr05: 369472 - 375911	2.2e-219
StWRKY58	PGSC0003DMG400028335	1527	508	3	chr05: 190450 - 195591	0.0e+00
StWRKY59-1	PGSC0003DMG400023196	642	213	2	chr12: 61495141 - 61497755	6.8e-141
StWRKY59-2	PGSC0003DMG400016957	375	124	1	chr12: 61548171 - 61549216	4.5e-80
StWRKY59-3	PGSC0003DMG400031140	696	213	2	chr04: 53285511 - 53288554	1.9e-39
StWRKY60-1	PGSC0003DMG400019824	1068	355	4	chr03: 42367030 - 42370130	3.3e-229
StWRKY60-2	PGSC0003DMG402007388	762	253	3	chr08: 29105448 - 29107825	4.4e-165
StWRKY61	PGSC0003DMG400018081	1578	525	4	chr03: 37657984 - 37662134	0.0e+00
StWRKY65	PGSC0003DMG400028469	900	299	2	chr02: 53057986 - 53060015	5.0e-199
StWRKY70-1	PGSC0003DMG400027582	672	223	2	chr06: 31181448 - 31183685	7.2e-153
StWRKY70-2	PGSC0003DMG400020608	834	277	2	chr03: 29342617 - 29345097	7.3e-187
StWRKY70-3	PGSC0003DMG400008391	657	218	2	chr10: 45985853 - 45990497	1.0e-146
StWRKY70-4	PGSC0003DMG400033884	885	294	2	chr05: 53931703 - 53933788	4.1e-200
StWRKY74	PGSC0003DMG400020432	771	256	3	chr07: 47052377 - 47056216	2.5e-176
StWRKY71	PGSC0003DMG400009703	957	318	2	chr02: 51758161 - 51759921	8.0e-216
StWRKY72	PGSC0003DMG402006935	1332	443	2	chr02: 47477448 - 47479898	9.2e-291
StWRKY75-1	PGSC0003DMG400021895	519	172	1	chr05: 14500093 - 14502731	2.5e-112
StWRKY75-2	PGSC0003DMG400020206	426	141	1	chr02: 69318661 - 69320526	1.2e-94
StWRKY75-3	PGSC0003DMG400009051	954	317	2	chr01: 68607070 - 68609524	2.9e-217
Sorghum bicolor						
SbWRKY1	Sb03g000240	1713	570	4	chr_3: 65767 - 68080	0.0e+00
SbWRKY2	Sb01g027770	1284	427	1	chr_1: 48360316 - 48361748	7.8e-296
SbWRKY3	Sb01g007570	996	331	2	chr_1: 6524859 - 6527473	3.4e-216
SbWRKY4	Sb01g007480	1275	424	3	chr_1: 6420226 - 6422321	2.0e-292
SbWRKY6	Sb01g005070	885	294	3	chr_1: 4145185 - 4147259	9.9e-194
SbWRKY7	Sb09g026830	660	219	2	chr_9: 56013261 - 56014828	4.3e-138
SbWRKY8	Sb09g029810	1032	343	2	chr_9: 58435466 - 58438688	1.3e-229
SbWRKY9	Sb03g011800	1749	582	5	chr_3: 13453957 - 13459197	0.0e+00
SbWRKY10	Sb03g003360	783	260	2	chr_3: 3574879 - 3576217	3.9e-171
SbWRKY11	Sb03g028530	1086	361	2	chr_3: 56464472 - 56466762	5.3e-246
SbWRKY12	Sb03g028440	1242	413	2	chr_3: 56296964 - 56298895	5.9e-279
SbWRKY13	Sb03g034670	933	310	2	chr_3: 62841197 - 62843114	5.6e-204
SbWRKY14	Sb03g033640	960	319	1	chr_3: 61888491 - 61890257	3.8e-203

SbWRKY15-1	Sb03g029920	1041	346	2	chr_3: 58204806 - 58206278	1.5e-229
SbWRKY15-2	Sb06g013835	702	234	2	chr_6: 38137577 - 38138457	6.5e-156
SbWRKY16	Sb03g030480	1233	410	2	chr_3: 58723799 - 58726767	7.1e-276
SbWRKY17	Sb03g047350	1065	354	2	chr_3: 74261998 - 74264051	1.3e-237
SbWRKY18	Sb03g038180	795	264	2	chr_3: 66110932 - 66116172	1.1e-182
SbWRKY19	Sb09g029050	819	272	2	chr_9: 57799582 - 57800648	1.4e-180
SbWRKY20	Sb03g038190	1134	377	2	chr_3: 66121144 - 66123805	1.0e-259
SbWRKY21	Sb03g038210	834	277	2	chr_3: 66144796 - 66146352	1.2e-189
SbWRKY22	Sb03g038170	711	236	2	chr_3: 66100608 - 66104629	1.8e-163
SbWRKY23-1	Sb03g033780	741	246	1	chr_3: 62036742 - 62039688	1.1e-164
SbWRKY23-2	Sb09g026350	726	241	1	chr_9: 55640076 - 55640932	1.8e-160
SbWRKY24	Sb03g038510	1758	585	3	chr_3: 66433156 - 66435382	0.0e+00
SbWRKY26	Sb03g032800	672	223	2	chr_3: 61257721 - 61258736	1.2e-147
SbWRKY28-2	Sb10g025590	1047	348	2	chr_10: 54957968 - 54959405	7.3e-226
SbWRKY28-1	Sb10g025600	1029	342	2	chr_10: 54965360 - 54966631	2.7e-222
SbWRKY29	Sb02g000960	1101	366	2	chr_2: 788461 - 793201	4.0e-243
SbWRKY30	Sb07g028430	1491	496	2	chr_7: 63403512 - 63405506	0.0e+00
SbWRKY31-1	Sb10g019923	696	232	4	chr_10: 42454390 - 42463629	1.2e-151
SbWRKY31-2	Sb10g020010	1053	350	1	chr_10: 42717205 - 42718403	2.4e-235
SbWRKY32	Sb04g034440	1737	578	4	chr_4: 64267731 - 64270424	0.0e+00
SbWRKY34	Sb04g033240	705	234	3	chr_4: 63147733 - 63151019	4.3e-164
SbWRKY35	Sb06g019710	2205	734	4	chr_6: 49279763 - 49283771	0.0e+00
SbWRKY36	Sb06g024220	747	248	3	chr_6: 53339360 - 53343815	3.1e-172
SbWRKY37	Sb06g027290	1599	532	3	chr_6: 56196796 - 56200982	0.0e+00
SbWRKY39	Sb04g009800	1128	375	2	chr_4: 12451791 - 12453160	3.9e-249
SbWRKY40	Sb08g002560	885	294	2	chr_8: 2649149 - 2650571	1.5e-199
SbWRKY42	Sb04g016540	726	241	2	chr_4: 38362358 - 38364004	7.4e-160
SbWRKY43	Sb09g028750	1791	596	5	chr_9: 57590098 - 57593135	0.0e+00
SbWRKY44	Sb01g036180	987	328	2	chr_1: 59806395 - 59807668	9.6e-211
SbWRKY45	Sb08g005080	1005	334	2	chr_8: 6492893 - 6494646	5.4e-218
SbWRKY46	Sb08g002590	666	221	2	chr_8: 2673615 - 2675815	8.0e-146
SbWRKY47	Sb02g043030	1065	354	2	chr_2: 76813673 - 76816113	4.4e-231
SbWRKY49	Sb09g028660	1275	424	2	chr_9: 57513946 - 57517030	2.0e-285

SbWRKY50-1	Sb08g002520	1026	341	2	chr_8: 2586470 - 2588302	1.2e-228
SbWRKY50-2	Sb07g005740	2250	749	5	chr_7: 8029114 - 8052209	0.0e+00
SbWRKY51	Sb07g006980	957	318	0	chr_7: 10712171 - 10713468	3.0e-202
SbWRKY53	Sb09g015900	1554	517	2	chr_9: 39917155 - 39919621	4.7e-264
SbWRKY55	Sb01g036870	678	225	2	chr_1: 60434864 - 60436282	2.5e-151
SbWRKY57	Sb07g006230	1368	455	3	chr_7: 8885371 - 8890727	2.7e-312
SbWRKY59	Sb02g021226	4755	1584	7	chr_2: 52728488 - 52736582	0.0e+00
SbWRKY60	Sb01g012870	303	100	1	chr_1: 11944441 - 11945284	3.3e-66
SbWRKY62	Sb02g024765	795	264	1	chr_2: 59281563 - 59282468	3.6e-169
SbWRKY66	Sb04g030930	1494	497	2	chr_4: 60932443 - 60936039	0.0e+00
SbWRKY67	Sb09g005700	621	206	2	chr_9: 7601936 - 7603335	1.9e-136
SbWRKY68	Sb06g027710	948	315	2	chr_6: 56529226 - 56530621	7.5e-205
SbWRKY69	Sb07g019400	1023	340	0	chr_7: 50025392 - 50026414	1.5e-227
SbWRKY70	Sb09g023270	1782	593	4	chr_9: 52888054 - 52890562	0.0e+00
SbWRKY71	Sb04g005520	1095	364	1	chr_4: 5399378 - 5401080	5.6e-236
SbWRKY72	Sb05g017130	678	225	1	chr_5: 42145584 - 42148750	1.5e-148
SbWRKY73	Sb03g026280	999	332	1	chr_3: 52879966 - 52881121	1.2e-216
SbWRKY74-1	Sb02g022290	987	328	2	chr_2: 55213197 - 55214862	3.8e-218
SbWRKY74-2	Sb02g022280	933	310	2	chr_2: 55170648 - 55172055	1.7e-209
SbWRKY76	Sb02g024760	984	327	2	chr_2: 59264547 - 59266116	2.0e-210
SbWRKY77	Sb03g026170	651	216	2	chr_3: 52684056 - 52684949	2.8e-143
SbWRKY78	Sb02g037660	1836	611	5	chr_2: 71945697 - 71952038	0.0e+00
SbWRKY80	Sb02g027950	1809	602	4	chr_2: 63184303 - 63187131	0.0e+00
SbWRKY81	Sb05g001170	1086	361	1	chr_5: 1225736 - 1226940	1.0e-245
SbWRKY82	Sb07g016330	1710	569	3	chr_7: 40441219 - 40446915	0.0e+00
SbWRKY83-1	Sb08g020270	1116	371	2	chr_8: 51276577 - 51278999	7.0e-247
SbWRKY83-2	Sb01g014180	1059	352	3	chr_1: 13370277 - 13372707	1.3e-235
SbWRKY85	Sb08g016240	1491	496	3	chr_8: 43243578 - 43248992	0.0e+00
SbWRKY86	Sb03g038200	1002	333	2	chr_3: 66129723 - 66132439	2.5e-223
SbWRKY87	Sb01g008550	1221	406	2	chr_1: 7382814 - 7385101	2.6e-271
SbWRKY88	Sb01g000696	1152	284	2	chr_1: 676390 - 680269	9.3e-261
SbWRKY90	Sb05g001220	753	250	2	chr_5: 1305458 - 1306752	2.2e-168
SbWRKY91	Sb09g023500	708	235	2	chr_9: 53146881 - 53149416	4.4e-156

SbWRKY92	Sb09g029850	1008	335	2	chr_9: 58485871 - 58487483	6.5e-230
SbWRKY93	Sb10g004000	1137	378	2	chr_10: 3530829 - 3534299	2.5e-254
SbWRKY94	Sb02g008880	2865	954	2	chr_2: 12422623 - 12426345	0.0e+00
SbWRKY95	Sb02g011050	651	216	1	chr_2: 17809234 - 17810769	5.9e-145
SbWRKY97	Sb03g003370	1647	548	2	chr_3: 3577819 - 3580200	0.0e+00
SbWRKY98-2	Sb05g001180	771	256	3	chr_5: 1246371 - 1247487	9.9e-174
SbWRKY98-1	Sb08g002570	729	242	2	chr_8: 2657310 - 2658335	1.1e-164
SbWRKY102	Sb03g003640	888	295	2	chr_3: 3846956 - 3853124	8.4e-196
SbWRKY103	Sb03g039550	1134	377	2	chr_3: 67223920 - 67229771	4.0e-255
SbWRKY105	Sb01g032120	1497	498	3	chr_1: 54964329 - 54971032	0.0e+00
SbWRKY106	Sb09g010210	648	215	1	chr_9: 20970162 - 20971240	3.2e-144
Thellungiella halophila						
ThWRKY1	Thhalv10002517m.g	1407	468	4	scaffold_4: 3754720 - 3757964	6.9e-308
ThWRKY2	Thhalv10012829m.g	2100	299	4	scaffold_2: 9972371 - 9976140	0.0e+00
ThWRKY3	Thhalv10004015m.g	1551	516	3	scaffold_6: 6979477 - 6983641	0.0e+00
ThWRKY4	Thhalv10007428m.g	1512	503	3	scaffold_5: 10719471 - 10721984	0.0e+00
ThWRKY5	Thhalv10001624m.g	666	221	3	scaffold_22: 550227 - 551924	2.4e-147
ThWRKY6	Thhalv10023390m.g	1671	556	5	scaffold_8: 2138453 - 2141101	0.0e+00
ThWRKY7	Thhalv10025630m.g	1038	345	2	scaffold_1: 6728184 - 6730215	1.4e-232
ThWRKY8	Thhalv10001240m.g	972	323	2	scaffold_20: 1781088 - 1783559	1.3e-215
ThWRKY9	Thhalv10018824m.g	1017	338	4	scaffold_9: 6039883 - 6042044	5.3e-226
ThWRKY10	Thhalv10012199m.g	1398	465	5	scaffold_7: 313134 - 315486	1.1e-312
ThWRKY11	Thhalv10025646m.g	1026	341	2	scaffold_1: 3593228 - 3594893	6.4e-228
ThWRKY13	Thhalv10025869m.g	888	295	2	scaffold_1: 478583 - 480996	3.0e-202
ThWRKY14	Thhalv10007691m.g	1293	430	2	scaffold_5: 4189335 - 4191990	2.3e-290
ThWRKY15	Thhalv10000270m.g	894	297	2	scaffold_15: 1812727 - 1814105	1.1e-190
ThWRKY17	Thhalv10000242m.g	987	328	2	scaffold_15: 3376509 - 3378173	1.0e-216
ThWRKY18	Thhalv10025785m.g	936	311	4	scaffold_1: 3489560 - 3491347	2.9e-208
ThWRKY20-1	Thhalv10024810m.g	1683	560	5	scaffold_1: 5698571 - 5702218	0.0e+00
ThWRKY20-2	Thhalv10013115m.g	1710	569	5	scaffold_2: 10342364 - 10345867	0.0e+00
ThWRKY21	Thhalv10016853m.g	1083	360	3	scaffold_10: 5806177 - 5808438	6.5e-243
ThWRKY22	Thhalv10028843m.g	903	300	2	scaffold_3: 369110 - 370489	1.4e-197
ThWRKY23	Thhalv10001802m.g	1050	349	2	scaffold_22: 1570865 - 1572508	2.7e-233

ThWRKY24	Thhalv10028213m.g	552	183	1	scaffold_14: 2450962 - 2452027	9.3e-123
ThWRKY25	Thhalv10016764m.g	1182	393	4	scaffold_10: 5698352 - 5700434	7.9e-266
ThWRKY27	Thhalv10013872m.g	1110	369	2	scaffold_2: 12216152 - 12217852	2.5e-250
ThWRKY28	Thhalv10025806m.g	924	307	2	scaffold_1: 9606005 - 9607259	9.1e-206
ThWRKY29	Thhalv10025799m.g	930	309	2	scaffold_1: 7080856 - 7082171	3.7e-207
ThWRKY30	Thhalv10004616m.g	954	317	2	scaffold_6: 3078865 - 3080289	3.7e-214
ThWRKY31	Thhalv10026864m.g	1617	538	5	scaffold_1: 7725182 - 7727910	0.0e+00
ThWRKY32	Thhalv10025126m.g	1401	466	4	scaffold_1: 3853663 - 3856005	4.2e-307
ThWRKY33	Thhalv10016542m.g	1521	506	4	scaffold_10: 9772299 - 9775039	0.0e+00
ThWRKY34	Thhalv10027010m.g	1719	527	3	scaffold_1: 5792491 - 5794492	0.0e+00
ThWRKY35	Thhalv10016714m.g	1266	421	2	scaffold_10: 7945036 - 7947706	3.0e-280
ThWRKY36	Thhalv10018677m.g	1185	394	3	scaffold_9: 4864156 - 4866701	1.1e-261
ThWRKY38	Thhalv10015295m.g	663	220	2	scaffold_2: 7652476 - 7653440	7.4e-150
ThWRKY39	Thhalv10021115m.g	993	330	2	scaffold_13: 8141840 - 8143841	2.4e-221
ThWRKY40	Thhalv10018925m.g	918	305	3	scaffold_9: 416177 - 418000	6.6e-195
ThWRKY41	Thhalv10028794m.g	993	330	2	scaffold_3: 5016482 - 5017908	5.5e-227
ThWRKY42	Thhalv10028586m.g	1539	512	5	scaffold_3: 2472439 - 2475201	0.0e+00
ThWRKY43	Thhalv10001738m.g	297	98	1	scaffold_22: 1103090 - 1103499	3.6e-63
ThWRKY44	Thhalv10016692m.g	1290	429	3	scaffold_10: 9129711 - 9131302	1.1e-288
ThWRKY46	Thhalv10001568m.g	885	294	2	scaffold_22: 1194909 - 1196112	4.8e-193
ThWRKY47	Thhalv10028616m.g	1464	487	5	scaffold_3: 779279 - 782717	0.0e+00
ThWRKY48	Thhalv10004295m.g	1257	418	2	scaffold_6: 4553416 - 4555540	2.6e-276
ThWRKY49	Thhalv10003250m.g	927	308	3	scaffold_17: 2067934 - 2069182	3.4e-211
ThWRKY50	Thhalv10005029m.g	507	168	2	scaffold_6: 3831112 - 3832190	6.7e-111
ThWRKY51	Thhalv10004930m.g	618	205	2	scaffold_6: 1118112 - 1119657	5.0e-138
ThWRKY53	Thhalv10025724m.g	972	323	2	scaffold_1: 6946602 - 6948343	2.5e-220
ThWRKY54	Thhalv10016713m.g	1269	422	4	scaffold_10: 10920790 - 10923282	2.0e-230
ThWRKY55	Thhalv10017926m.g	864	288	2	scaffold_10: 10916595 - 10918437	8.7e-195
ThWRKY56	Thhalv10024097m.g	582	193	1	scaffold_8: 574763 - 577071	5.1e-131
ThWRKY57	Thhalv10018976m.g	846	281	3	scaffold_9: 5230131 - 5233918	1.5e-189
ThWRKY58	Thhalv10020676m.g	1401	466	3	scaffold_13: 9110201 - 9112381	0.0e+00
ThWRKY59	Thhalv10000331m.g	594	197	2	scaffold_15: 497862 - 499813	5.9e-133
ThWRKY60	Thhalv10000288m.g	810	269	4	scaffold_15: 4535360 - 4537096	2.0e-176

ThWRKY61	Thhalv10007461m.g	1485	494	3	scaffold_5: 9013345 - 9015895	0.0e+00
ThWRKY62	Thhalv10014700m.g	618	205	2	scaffold_2: 48116 - 48933	4.6e-138
ThWRKY64	Thhalv10029232m.g	777	258	2	scaffold_3: 8505663 - 8506772	3.8e-177
ThWRKY65	Thhalv10008510m.g	789	262	1	scaffold_5: 5062361 - 5063616	3.8e-176
ThWRKY67	Thhalv10000686m.g	945	314	2	scaffold_15: 4860365 - 4861501	3.6e-216
ThWRKY68	Thhalv10006458m.g	843	280	2	scaffold_19: 375201 - 376245	3.0e-192
ThWRKY69	Thhalv10006157m.g	825	274	2	scaffold_19: 1670395 - 1672193	2.3e-185
ThWRKY70	Thhalv10006146m.g	843	280	2	scaffold_19: 2581646 - 2583479	4.9e-190
ThWRKY71	Thhalv10009672m.g	867	288	2	scaffold_5: 4862758 - 4863999	1.2e-195
ThWRKY72	Thhalv10013146m.g	1674	557	3	scaffold_2: 4987474 - 4990084	0.0e+00
ThWRKY74	Thhalv10004506m.g	1044	347	3	scaffold_6: 4433212 - 4435365	1.3e-234
ThWRKY75	Thhalv10014943m.g	444	147	1	scaffold_2: 4223260 - 4224612	8.3e-95
Theobroma cacao						
TcWRKY1	Thecc1EG002318	1512	503	4	scaffold_1: 13136653 - 13139992	0.0e+00
TcWRKY2	Thecc1EG025248	2271	756	6	scaffold_5: 31469642 - 31478174	0.0e+00
TcWRKY3	Thecc1EG011624	1695	564	4	scaffold_2: 40994929 - 40999502	0.0e+00
TcWRKY5	Thecc1EG005744	624	207	3	scaffold_1: 38058528 - 38061344	7.3e-140
TcWRKY6	Thecc1EG030835	1743	580	5	scaffold_7: 1572272 - 1575629	0.0e+00
TcWRKY7	Thecc1EG033777	1080	359	2	scaffold_8: 126632 - 128568	7.0e-241
TcWRKY9	Thecc1EG006426	1575	524	5	scaffold_2: 2175028 - 2179597	0.0e+00
TcWRKY11	Thecc1EG036832	1023	340	2	scaffold_9: 433148 - 435462	3.3e-223
TcWRKY13	Thecc1EG001282	681	226	2	scaffold_1: 6276279 - 6281251	1.3e-153
TcWRKY14	Thecc1EG026992	1281	426	2	scaffold_6: 672373 - 675634	7.7e-282
TcWRKY15	Thecc1EG000680	1047	348	3	scaffold_1: 2983189 - 2986450	1.5e-226
TcWRKY16	Thecc1EG014419	3237	1078	10	scaffold_3: 22285422 - 22297067	0.0e+00
TcWRKY18	Thecc1EG036912	768	255	3	scaffold_9: 818257 - 820009	3.0e-166
TcWRKY20	Thecc1EG030735	1701	566	5	scaffold_7: 1124618 - 1129178	0.0e+00
TcWRKY21	Thecc1EG004187	1059	352	2	scaffold_1: 30142184 - 30144556	7.7e-235
TcWRKY22-1	Thecc1EG005261	1023	340	2	scaffold_1: 35798144 - 35800148	1.9e-225
TcWRKY22-2	Thecc1EG016343	960	319	2	scaffold_3: 32461030 - 32462557	1.5e-217
TcWRKY23-1	Thecc1EG004843	984	327	2	scaffold_1: 33736924 - 33739413	1.7e-223
TcWRKY23-2	Thecc1EG021400	1086	361	2	scaffold_4: 32908685 - 32910524	4.5e-241
TcWRKY26	Thecc1EG042240	1818	605	5	scaffold_9: 41643864 - 41648918	0.0e+00

TcWRKY27	Thecc1EG046204	1638	545	4	scaffold_112: 1 - 4171	0.0e+00
TcWRKY30	Thecc1EG006109	4350	1449	11	scaffold_2: 820964 - 828678	0.0e+00
TcWRKY31	Thecc1EG002087	1899	632	5	scaffold_1: 11532303 - 11536231	0.0e+00
TcWRKY32	Thecc1EG037060	1524	507	4	scaffold_9: 1542222 - 1546629	0.0e+00
TcWRKY33	Thecc1EG021580	3717	1238	12	scaffold_5: 306561 - 315476	0.0e+00
TcWRKY34	Thecc1EG030504	2115	704	6	scaffold_7: 49476 - 54345	0.0e+00
TcWRKY35-1	Thecc1EG004820	810	269	3	scaffold_1: 33627856 - 33631800	5.2e-178
TcWRKY35-2	Thecc1EG016012	927	308	3	scaffold_3: 30839940 - 30843967	3.3e-206
TcWRKY39	Thecc1EG026314	1059	352	2	scaffold_5: 37657896 - 37660017	1.6e-238
TcWRKY40-1	Thecc1EG027435	930	309	4	scaffold_6: 3870167 - 3872047	3.5e-194
TcWRKY40-2	Thecc1EG036913	930	309	4	scaffold_9: 824894 - 827211	6.1e-204
TcWRKY41-1	Thecc1EG015346	1086	361	3	scaffold_3: 27474680 - 27479693	5.5e-243
TcWRKY41-2	Thecc1EG016426	1080	359	2	scaffold_3: 32846398 - 32848357	2.4e-246
TcWRKY42	Thecc1EG030268	1782	593	5	scaffold_6: 26458212 - 26461307	0.0e+00
TcWRKY43	Thecc1EG005256	660	219	2	scaffold_1: 35781448 - 35783914	4.5e-146
TcWRKY44	Thecc1EG022065	1425	474	7	scaffold_5: 2446310 - 2451444	0.0e+00
TcWRKY45	Thecc1EG014673	492	163	1	scaffold_3: 23785784 - 23786854	3.6e-108
TcWRKY46	Thecc1EG005179	1059	352	2	scaffold_1: 35465515 - 35468270	8.6e-237
TcWRKY47	Thecc1EG004951	1536	511	6	scaffold_1: 34290842 - 34294453	0.0e+00
TcWRKY49	Thecc1EG021911	1200	399	3	scaffold_5: 1731425 - 1735591	8.4e-273
TcWRKY50-1	Thecc1EG035897	696	231	2	scaffold_8: 13601328 - 13603184	1.4e-157
TcWRKY50-2	Thecc1EG037343	480	159	2	scaffold_9: 2972731 - 2974198	1.1e-105
TcWRKY51	Thecc1EG001265	738	245	2	scaffold_1: 6138938 - 6143183	1.0e-168
TcWRKY52	Thecc1EG014427	873	290	3	scaffold_3: 22360304 - 22364674	1.2e-195
TcWRKY53-1	Thecc1EG006116	4143	1380	5	scaffold_2: 845249 - 851848	0.0e+00
TcWRKY53-2	Thecc1EG006103	4596	1531	12	scaffold_2: 805474 - 814178	0.0e+00
TcWRKY54	Thecc1EG045556	888	295	3	scaffold_10r: 25159496 - 25164739	2.7e-200
TcWRKY55	Thecc1EG045555	1134	377	2	scaffold_10r: 25156194 - 25159321	4.9e-253
TcWRKY57	Thecc1EG011578	915	304	3	scaffold_2: 40794286 - 40799502	4.7e-204
TcWRKY58	Thecc1EG018667	1563	520	4	scaffold_4: 17119792 - 17124222	0.0e+00
TcWRKY65	Thecc1EG029654	861	286	2	scaffold_6: 23693151 - 23694650	7.6e-190
TcWRKY70	Thecc1EG019689	858	285	3	scaffold_4: 24307987 - 24314095	4.4e-189
TcWRKY71-1	Thecc1EG001885	951	316	2	scaffold_1: 10031710 - 10034197	7.7e-213

TcWRKY71-2	Thecc1EG029543	1116	371	5	scaffold_6: 23115925 - 23128951	5.8e-252
TcWRKY72-1	Thecc1EG013860	1839	612	5	scaffold_3: 17944872 - 17949398	0.0e+00
TcWRKY72-2	Thecc1EG018403	1713	570	5	scaffold_4: 14416116 - 14432532	0.0e+00
TcWRKY72-3	Thecc1EG011792	1815	604	4	scaffold_2: 41755644 - 41759595	0.0e+00
TcWRKY75-1	Thecc1EG017248	534	177	1	scaffold_4: 2544051 - 2545769	1.7e-117
TcWRKY75-2	Thecc1EG008635	558	185	1	scaffold_2: 15575303 - 15578776	1.9e-121
Vitis vinifera						
VvWRKY1	GSVIVT01030046001	1095	364	5	chr12: 9116731 - 9122807	2.1e-234
VvWRKY2	GSVIVT01014854001	1869	622	8	chr19: 10665036 - 10669055	0.0e+00
VvWRKY3	GSVIVT01001332001	1308	435	5	chr1_random: 297660 - 312015	5.1e-273
VvWRKY5	GSVIVT01018300001	687	228	3	chr15: 11498970 - 11504729	2.0e-118
VvWRKY6	GSVIVT01012682001	1533	510	6	chr10: 618603 - 621252	0.0e+00
VvWRKY7	GSVIVT01009441001	960	319	4	chr18: 8391930 - 8393726	7.3e-211
VvWRKY9	GSVIVT01029688001	1473	490	5	chr12: 13065135 - 13099628	0.0e+00
VvWRKY10	GSVIVT01035965001	1593	530	5	chr4: 6569931 - 6576637	0.0e+00
VvWRKY11	GSVIVT01029265001	840	279	3	chr11: 17821900 - 17823266	2.1e-177
VvWRKY13	GSVIVT01022259001	681	226	2	chr7: 17958306 - 17960930	1.3e-153
VvWRKY14	GSVIVT01021765001	1266	421	3	chr10: 10755760 - 10759820	1.6e-232
VvWRKY15	GSVIVT01022067001	843	280	4	chr7: 16322549 - 16324116	3.2e-143
VvWRKY17	GSVIVT01033188001	804	267	4	chr4: 9363169 - 9365026	9.4e-170
VvWRKY18	GSVIVT01035885001	861	286	5	chr4: 5265806 - 5268041	9.7e-181
VvWRKY20-1	GSVIVT01037775001	1659	552	5	chr19: 7760186 - 7767468	0.0e+00
VvWRKY20-2	GSVIVT01007006001	5625	550	5	chrUn: 29694308 - 29699639	0.0e+00
VvWRKY21-1	GSVIVT01000752001	855	284	4	chr7: 381035 - 383836	3.5e-185
VvWRKY21-2	GSVIVT01036223001	915	304	4	chr14: 8753538 - 8756300	4.9e-194
VvWRKY23	GSVIVT01028147001	909	302	2	chr7: 4200160 - 4202241	2.7e-204
VvWRKY26	GSVIVT01019109001	1461	486	4	chr4: 16664476 - 16666741	2.9e-293
VvWRKY27-1	GSVIVT01026965001	1047	348	2	chr15: 18957231 - 18958817	2.9e-237
VvWRKY27-2	GSVIVT01028823001	549	182	1	chr16: 18360079 - 18360711	2.1e-118
VvWRKY29	GSVIVT01019419001	972	323	2	chr2: 512163 - 513533	1.3e-220
VvWRKY30	GSVIVT01027069001	1083	360	4	chr15: 18191021 - 18193489	4.4e-209
VvWRKY31	GSVIVT01030453001	1497	498	7	chr12: 5678707 - 5681171	4.9e-311
VvWRKY32	GSVIVT01023600001	1500	499	5	chr11: 7835815 - 7846137	0.0e+00

VvWRKY33-1	GSVIVT01030258001	1542	513	4	chr8: 9796056 - 9798907	0.0e+00
VvWRKY33-2	GSVIVT01024624001	1713	570	10	chr6: 8290025 - 8295109	1.9e-314
VvWRKY35	GSVIVT01028129001	729	242	3	chr7: 4044128 - 4045807	4.0e-163
VvWRKY40	GSVIVT01015952001	837	278	5	chr9: 16094219 - 16096253	3.5e-176
VvWRKY41	GSVIVT01019511001	1029	342	2	chr2: 1228314 - 1229702	3.3e-239
VvWRKY42	GSVIVT01037686001	1491	496	5	chr19: 6882419 - 6884988	0.0e+00
VvWRKY43	GSVIVT01026969001	606	201	1	chr15: 18940954 - 18942146	4.1e-135
VvWRKY44	GSVIVT01025562001	1317	438	7	chr8: 14033451 - 14039562	2.6e-287
VvWRKY46	GSVIVT01028718001	1095	364	2	chr16: 19477141 - 19479868	1.7e-246
VvWRKY47	GSVIVT01028244001	1440	479	6	chr7: 4899927 - 4903175	6.7e-310
VvWRKY48	GSVIVT01034968001	930	309	2	chr5: 530084 - 531773	8.8e-204
VvWRKY49	GSVIVT01034148001	900	299	2	chr8: 14828040 - 14830056	2.8e-198
VvWRKY50	GSVIVT01035426001	501	166	2	chr4: 1209585 - 1211712	3.6e-108
VvWRKY51-1	GSVIVT01033194001	471	156	2	chr4: 9399944 - 9400803	2.5e-104
VvWRKY51-2	GSVIVT01033195001	306	101	1	chr4: 9409805 - 9411286	1.4e-67
VvWRKY51-3	GSVIVT01022245001	582	193	2	chr7: 17794379 - 17797240	8.7e-120
VvWRKY54	GSVIVT01030174001	996	331	4	chr8: 10843756 - 10846082	7.1e-216
VvWRKY55	GSVIVT01032662001	927	308	3	chr13: 1716836 - 1718836	6.7e-203
VvWRKY57-1	GSVIVT01012196001	852	283	5	chr1: 628682 - 633595	4.2e-183
VvWRKY57-2	GSVIVT01021397001	960	319	2	chr10: 4894476 - 4896340	7.9e-217
VvWRKY58	GSVIVT01011472001	2670	889	16	chr14: 29916400 - 29925511	0.0e+00
VvWRKY60	GSVIVT01035884001	789	262	3	chr4: 5247592 - 5248886	4.9e-175
VvWRKY61	GSVIVT01020060001	1785	594	4	chr1: 10977206 - 10982423	0.0e+00
VvWRKY69	GSVIVT01021252001	837	278	2	chr10: 3008687 - 3010451	3.4e-187
VvWRKY70	GSVIVT01032661001	867	288	3	chr13: 1719393 - 1720884	1.8e-186
VvWRKY71	GSVIVT01020864001	936	311	2	chr12: 878934 - 881289	2.3e-215
VvWRKY72-1	GSVIVT01008046001	1818	605	5	chr17: 6316168 - 6320317	0.0e+00
VvWRKY72-2	GSVIVT01011356001	1509	502	3	chr14: 28923786 - 28926499	0.0e+00
VvWRKY75-1	GSVIVT01010525001	570	189	1	chr1: 21460123 - 21461397	1.9e-126
VvWRKY75-2	GSVIVT01008553001	456	151	1	chr17: 922600 - 925171	1.3e-101
Volvox carteri						
VcWRKY4	Vocar20009416m.g	1908	635	10	scaffold_1: 13925593 - 13929564	4.6e-250
VcWRKY33	Vocar20013412m.g	3552	1183	20	scaffold_58: 216444 - 231417	0.0e+00

Zea mays						
ZmWRKY1	GRMZM5G871347	1359	453	3	Chr3: 1260878 - 1262711	1.5e-180
ZmWRKY2	GRMZM2G383594	1323	440	1	Chr1: 105951470 - 105952881	2.1e-300
ZmWRKY3-1	GRMZM2G018721	999	332	2	Chr7: 1920108 - 1921464	2.2e-218
ZmWRKY3-2	GRMZM2G143204	996	331	3	Chr1: 278162171 - 278165808	7.5e-219
ZmWRKY4	GRMZM2G008029	1353	450	3	Chr1: 278602439 - 278604715	9.2e-313
ZmWRKY6	GRMZM2G164082	1263	420	2	Chr1: 288695814 - 288697843	4.2e-275
ZmWRKY7	GRMZM5G863420	666	221	2	Chr6: 161477481 - 161478917	7.1e-143
ZmWRKY9-1	GRMZM2G327349	1746	581	5	Chr3: 49562769 - 49566492	0.0e+00
ZmWRKY9-2	GRMZM2G092694	924	307	5	Chr8: 653032 - 655605	6.7e-207
ZmWRKY9-3	GRMZM2G448605	1833	610	5	Chr8: 71057846 - 71061504	0.0e+00
ZmWRKY10-1	GRMZM2G516301	690	229	2	Chr8: 19834281 - 19835285	5.7e-155
ZmWRKY10-2	GRMZM2G475984	822	273	2	Chr3: 8213140 - 8214362	1.9e-180
ZmWRKY11-1	AC198725.4_FG009	1059	352	2	Chr3: 217212884 - 217215223	1.5e-240
ZmWRKY11-2	GRMZM2G145554	1080	359	2	Chr8: 145527152 - 145529521	2.7e-246
ZmWRKY12-1	GRMZM2G105140	1038	345	2	Chr3: 217462232 - 217464089	1.3e-226
ZmWRKY12-2	GRMZM2G414315	1044	347	2	Chr8: 74902322 - 74903632	1.0e-234
ZmWRKY12-3	GRMZM2G138683	1401	466	2	Chr4: 158861929 - 158866266	0.0e+00
ZmWRKY13-1	GRMZM2G141299	921	306	2	Chr3: 198214591 - 198216492	2.1e-203
ZmWRKY13-2	GRMZM2G013391	834	277	2	Chr8: 174376651 - 174378151	4.7e-184
ZmWRKY14	GRMZM2G040298	864	287	1	Chr3: 201013567 - 201015203	3.6e-187
ZmWRKY15-1	GRMZM2G411766	984	327	2	Chr8: 148006878 - 148008698	2.4e-220
ZmWRKY15-2	GRMZM2G408462	1005	334	2	Chr3: 212739888 - 212741309	4.3e-224
ZmWRKY16-1	GRMZM2G151763	1146	381	2	Chr3: 210977929 - 210981079	1.2e-258
ZmWRKY16-2	GRMZM5G812272	1245	414	2	Chr8: 149077318 - 149080011	4.2e-132
ZmWRKY17	GRMZM2G149219	300	99	1	Chr1: 258955026 - 258956380	5.4e-67
ZmWRKY18	GRMZM2G065290	792	263	2	Chr3: 184932051 - 184941349	2.0e-180
ZmWRKY19	GRMZM2G063880	774	257	2	Chr8: 72382998 - 72384377	5.5e-172
ZmWRKY20	GRMZM2G029282	1131	376	2	Chr8: 170352645 - 170355460	2.0e-258
ZmWRKY21-1	GRMZM2G059562	813	270	2	Chr3: 184816891 - 184818185	1.7e-181
ZmWRKY21-2	GRMZM2G432583	954	317	2	Chr8: 119035678 - 119037047	3.7e-216
ZmWRKY22	GRMZM2G382350	723	240	2	Chr3: 185029884 - 185034399	9.6e-165
ZmWRKY23-1	GRMZM2G111354	684	227	1	Chr8: 175697173 - 175698487	6.1e-151

ZmWRKY23-2	GRMZM2G015433	690	229	1	Chr8: 124950950 - 124952109	2.1e-153
ZmWRKY23-3	GRMZM2G401521	1005	334	0	Chr6: 159824142 - 159825486	1.1e-198
ZmWRKY24-1	GRMZM2G148087	1668	555	4	Chr3: 183888339 - 183891343	0.0e+00
ZmWRKY24-2	GRMZM2G036703	1782	593	4	Chr8: 170094714 - 170097223	0.0e+00
ZmWRKY25-1	GRMZM2G148561	921	306	0	Chr4: 69920108 - 69921324	3.7e-197
ZmWRKY25-2	GRMZM2G090594	936	311	0	Chr10: 68725531 - 68726795	2.4e-200
ZmWRKY26	GRMZM2G101405	642	213	2	Chr3: 203083255 - 203084247	7.0e-142
ZmWRKY27-1	GRMZM2G304573	897	298	1	Chr8: 139951730 - 139952759	3.7e-198
ZmWRKY27-2	GRMZM5G851490	1530	509	4	Chr10: 87094728 - 87099466	0.0e+00
ZmWRKY27-3	GRMZM2G354384	516	171	0	Chr3: 221277867 - 221278550	1.1e-110
ZmWRKY28	GRMZM2G111711	1056	351	2	Chr9: 103075648 - 103077219	6.7e-229
ZmWRKY30	GRMZM2G027972	2190	729	3	Chr4: 198130795 - 198133409	0.0e+00
ZmWRKY31-1	GRMZM2G006497	1026	341	1	Chr8: 13136608 - 13138411	7.6e-232
ZmWRKY31-2	GRMZM2G156529	756	251	1	Chr6: 110436475 - 110437394	7.7e-169
ZmWRKY35-1	GRMZM2G031963	2019	672	5	Chr10: 124659347 - 124664396	0.0e+00
ZmWRKY35-2	GRMZM5G816457	2112	703	5	Chr2: 38760033 - 38765436	0.0e+00
ZmWRKY36-1	GRMZM2G123387	855	284	3	Chr2: 21500615 - 21505542	1.0e-196
ZmWRKY36-2	GRMZM2G377217	708	235	3	Chr4: 151070766 - 151082721	3.8e-164
ZmWRKY37	AC208110.2_FG001	1584	527	3	Chr10: 140656185 - 140659057	0.0e+00
ZmWRKY39-1	GRMZM2G038158	1113	370	2	Chr4: 223519261 - 223521139	1.9e-245
ZmWRKY39-2	GRMZM2G048450	1152	383	2	Chr5: 138345948 - 138348076	1.1e-255
ZmWRKY42	GRMZM2G073272	978	325	2	Chr5: 159232314 - 159233527	4.5e-218
ZmWRKY43	GRMZM2G366795	1680	559	5	Chr6: 165021433 - 165023895	0.0e+00
ZmWRKY45	GRMZM2G004060	1146	381	2	Chr10: 12103219 - 12104937	1.8e-248
ZmWRKY46	GRMZM2G063216	687	228	2	Chr4: 184639458 - 184643071	3.8e-153
ZmWRKY47	GRMZM2G381378	1017	338	2	Chr7: 173221792 - 173223897	5.2e-218
ZmWRKY49	GRMZM2G453571	1227	408	2	Chr6: 164799868 - 164802835	8.0e-272
ZmWRKY50	GRMZM2G005207	975	324	2	Chr10: 3992095 - 3994352	1.5e-219
ZmWRKY52	GRMZM2G099593	1053	350	1	Chr2: 151734355 - 151735768	1.1e-238
ZmWRKY53-1	GRMZM2G012724	1470	489	2	Chr6: 141413048 - 141415248	0.0e+00
ZmWRKY53-2	GRMZM2G449681	1497	498	1	Chr8: 110214059 - 110216431	0.0e+00
ZmWRKY55-1	GRMZM2G030272	645	214	2	Chr1: 52919938 - 52921358	6.8e-143
ZmWRKY55-2	GRMZM2G060918	591	196	2	Chr9: 133694708 - 133696240	2.0e-126

ZmWRKY56	GRMZM2G441031	861	286	2	Chr10: 4032506 - 4034042	4.6e-194
ZmWRKY57	GRMZM2G020254	750	249	4	Chr10: 65416247 - 65418035	7.1e-154
ZmWRKY58	GRMZM2G178671	543	181	1	Chr3: 180598307 - 180598998	4.7e-86
ZmWRKY60	GRMZM2G161411	423	140	2	Chr5: 15320902 - 15322507	7.3e-91
ZmWRKY62	GRMZM2G169149	801	266	2	Chr7: 115548526 - 115549758	9.4e-173
ZmWRKY66	GRMZM2G024898	1518	505	2	Chr2: 12799342 - 12803684	0.0e+00
ZmWRKY67	GRMZM2G057116	618	205	2	Chr8: 88963901 - 88965293	5.2e-137
ZmWRKY68-1	GRMZM2G091331	951	316	2	Chr10: 141270670 - 141272273	2.6e-203
ZmWRKY68-2	GRMZM2G071907	897	298	2	Chr2: 11753531 - 11755126	1.0e-194
ZmWRKY69	GRMZM2G324999	1002	333	0	Chr1: 216580897 - 216582541	4.1e-221
ZmWRKY70	GRMZM2G169966	1143	380	4	Chr6: 153264614 - 153267678	3.9e-263
ZmWRKY71	GRMZM2G120320	1191	396	1	Chr5: 92804729 - 92806565	1.4e-260
ZmWRKY72-1	GRMZM2G106560	663	220	1	Chr2: 221753525 - 221758060	2.7e-146
ZmWRKY72-2	GRMZM2G054125	690	229	1	Chr4: 17852745 - 17855630	2.8e-152
ZmWRKY73	GRMZM2G083350	1623	540	2	Chr8: 19851097 - 19853367	0.0e+00
ZmWRKY74-1	GRMZM2G163418	996	331	2	Chr2: 175752568 - 175755074	4.3e-224
ZmWRKY74-2	GRMZM2G061408	975	324	2	Chr8: 109566616 - 109568284	2.9e-216
ZmWRKY74-3	GRMZM2G025895	975	324	2	Chr7: 93164343 - 93166011	2.9e-216
ZmWRKY74-4	GRMZM2G139815	909	302	2	Chr7: 92391794 - 92393825	1.4e-202
ZmWRKY76-1	GRMZM2G057011	1056	351	1	Chr2: 180621363 - 180622626	1.4e-233
ZmWRKY76-2	GRMZM2G125653	909	302	2	Chr7: 115308636 - 115310466	2.2e-197
ZmWRKY77-1	GRMZM2G137802	576	191	3	Chr8: 136039539 - 136041895	1.0e-124
ZmWRKY77-2	GRMZM2G163054	588	195	2	Chr6: 127590620 - 127591919	1.9e-98
ZmWRKY78-1	GRMZM2G130854	1833	610	5	Chr2: 207841392 - 207847758	0.0e+00
ZmWRKY78-2	GRMZM2G398506	1842	613	5	Chr7: 161289900 - 161300473	0.0e+00
ZmWRKY79	GRMZM2G127064	1230	409	2	Chr6: 135702507 - 135705444	2.8e-271
ZmWRKY80	GRMZM2G151407	1752	583	5	Chr7: 131286942 - 131290571	2.1e-220
ZmWRKY81	GRMZM2G461648	1125	374	1	Chr4: 184676411 - 184677679	1.2e-255
ZmWRKY82	GRMZM2G549512	1701	566	3	Chr4: 58331160 - 58349507	0.0e+00
ZmWRKY83-1	GRMZM2G173680	1104	367	3	Chr3: 116024859 - 116027909	4.1e-243
ZmWRKY83-2	GRMZM2G130374	1125	367	2	Chr1: 167896338 - 167898547	1.9e-250
ZmWRKY83-3	GRMZM2G070211	1065	354	3	Chr1: 253502280 - 253505400	3.9e-237
ZmWRKY84	GRMZM2G425430	915	304	2	Chr1: 75169610 - 75170840	1.3e-206

ZmWRKY85-1	GRMZM2G143765	1491	496	3	Chr6: 22907900 - 22912535	0.0e+00
ZmWRKY85-2	GRMZM2G076657	1485	494	3	Chr3: 134915466 - 134922903	0.0e+00
ZmWRKY86	GRMZM2G158328	1002	333	2	Chr3: 184849632 - 184852523	2.5e-221
ZmWRKY87-1	GRMZM2G018487	1194	397	3	Chr1: 274880474 - 274884612	7.2e-265
ZmWRKY87-2	GRMZM2G147880	1188	395	3	Chr5: 7868470 - 7872927	2.0e-261
ZmWRKY88	GRMZM2G083717	1143	380	2	Chr1: 299282977 - 299288556	1.6e-259
ZmWRKY90-1	GRMZM2G400559	936	311	2	Chr2: 151685379 - 151686596	1.4e-211
ZmWRKY90-2	GRMZM2G052671	1704	567	6	Chr2: 189310197 - 189314231	0.0e+00
ZmWRKY90-3	GRMZM2G169564	1038	345	2	Chr4: 184781651 - 184783303	1.3e-87
ZmWRKY91	GRMZM2G034421	687	228	2	Chr8: 118969410 - 118971797	1.8e-150
ZmWRKY92-1	GRMZM2G149683	1050	349	2	Chr8: 145230331 - 145231761	2.2e-234
ZmWRKY92-2	GRMZM5G823157	1455	484	2	Chr5: 199646796 - 199650458	0.0e+00
ZmWRKY93	GRMZM2G003551	1191	396	2	Chr9: 18101309 - 18104762	3.1e-264
ZmWRKY97	GRMZM2G176489	1644	547	2	Chr3: 8217987 - 8220347	0.0e+00
ZmWRKY102	GRMZM2G151444	882	293	2	Chr3: 9234113 - 9242047	7.4e-198
ZmWRKY103	GRMZM2G045560	1029	343	2	Chr8: 168823637 - 168828313	2.0e-144
ZmWRKY104	GRMZM2G451035	918	305	2	Chr4: 184661228 - 184662336	1.5e-209
ZmWRKY105	GRMZM2G171428	1494	497	3	Chr9: 124229921 - 124236498	0.0e+00

References

1. Lamesch, P. *et al.* The Arabidopsis Information Resource (TAIR): improved gene annotation and new tools. *Nucleic Acids Res.* **40,** D1202–10 (2012).
2. Ouyang, S. *et al.* The TIGR Rice Genome Annotation Resource: improvements and new features. *Nucleic Acids Res.* **35,** D883–7 (2007).
3. Goodstein, D. M. *et al.* Phytozome: a comparative platform for green plant genomics. *Nucleic Acids Res.* **40,** D1178–86 (2012).
4. Nordberg, H. *et al.* The genome portal of the Department of Energy Joint Genome Institute: 2014 updates. *Nucleic Acids Res.* **42,** D26–31 (2014).

Supplementary Table 2

Expression profile of WRKY transcription factor in different tissue sample in *Glycine max* and *Phaseolus vulgaris*. Expression datas are presented as FPKM (Fragments per kilobase of exon per million reads mapped).

Gene	Locus ID	Roots	Root Hairs	Leaves	Stems	Flowers	Pods	Seeds	Nodules	Shoot Apical Meristems
					Glycine max					
GmWRKY1-1	Glyma.14G010100	0.386	4.592	2.924	3.649	1.56	3.48	1.14	5.85	1.08
GmWRKY1-2	Glyma.02G306300	12.708	2.405	7.025	5.233	4.584	5.206	3.230	5.302	6.309
GmWRKY2-1	Glyma.09G250500	0.396	0.397	0.221	0.978	0.338	0.717	0.260	0.635	0.796
GmWRKY2-2	Glyma.18G242000	0.320	0.319	0.122	0.479	0.524	0.409	0.359	0.352	0.389
GmWRKY3-1	Glyma.08G240800	18.363	7.784	21.572	9.511	8.389	9.665	4.095	30.341	6.766
GmWRKY3-2	Glyma.18G263400	18.871	6.709	17.044	7.964	2.715	6.499	5.200	2.889	6.407
GmWRKY3-3	Glyma.20G028000	1.192	5.122	6.944	3.946	7.739	1.483	1.354	6.254	2.115
GmWRKY3-4	Glyma.07G227200	0.496	1.169	1.976	0.289	0.661	1.030	0	0.754	0.211
GmWRKY4-1	Glyma.02G112100	14.191	5.911	14.616	2.082	4.110	3.429	1.944	6.305	6.041
GmWRKY4-2	Glyma.01G053800	3.699	7.190	4.343	2.186	0.564	1.158	0.594	7.681	1.854
GmWRKY4-3	Glyma.10G171100	0	0	0	0	0.016	0	0.251	0	0
GmWRKY5-1	Glyma.03G109100	0	0.106	0.271	2.171	1.079	0.364	0	0.503	1.971
GmWRKY5-2	Glyma.07G116300	0	0.228	0.058	0.583	0.862	0.496	0.050	0.019	1.664
GmWRKY6-1	Glyma.08G320200	3.248	1.454	0.106	0	0.003	0.012	0.074	3.931	0
GmWRKY6-2	Glyma.18G092200	5.277	0.982	0.116	0	0.143	0.013	0.110	1.659	0.097
GmWRKY6-3	Glyma.02G293400	0.036	0	0	0	0.136	0	0	0.043	0
GmWRKY6-4	Glyma.09G005700	74.668	17.248	51.394	2.109	34.774	0.646	0.028	12.139	3.516
GmWRKY6-5	Glyma.15G110300	43.341	14.790	81.847	2.746	24.455	1.053	0.171	11.363	2.308
GmWRKY6-6	Glyma.13G310100	21.264	3.890	19.862	1.276	2.391	0.228	0.224	3.768	0.977
GmWRKY6-7	Glyma.17G042300	1.510	0.769	0.046	0.321	0.348	0.155	0.023	0.445	0.063
GmWRKY6-8	Glyma.13G117600	6.002	0.669	1.123	0.435	1.077	0.370	0	1.042	0.107
GmWRKY9	Glyma.05G123000	2.577	7.099	0.612	0.390	0.432	0.758	0	9.275	1.516
GmWRKY10	Glyma.10G171200	0	0	0	0	0	0	0	0	0

GmWRKY11-1	Glyma.06G077400	24.934	19.310	7.858	10.795	10.829	3.655	3.561	25.202	4.504
GmWRKY11-2	Glyma.04G076200	38.845	24.979	6.707	7.046	8.917	5.624	6.612	36.175	4.703
GmWRKY11-3	Glyma.17G057100	27.214	16.569	13.862	10.503	6.052	5.937	3.257	18.965	4.918
GmWRKY11-4	Glyma.13G102000	18.216	19.014	14.031	7.992	8.144	5.209	4.204	20.702	5.434
GmWRKY11-5	Glyma.09G061900	11.106	8.703	17.921	12.466	23.662	9.898	3.196	7.771	6.144
GmWRKY11-6	Glyma.15G168200	22.425	17.389	22.818	24.872	26.079	9.725	2.092	10.665	7.020
GmWRKY11-7	Glyma.14G135400	5.394	19.930	11.951	6.574	2.907	2.039	3.147	21.960	9.635
GmWRKY11-8	Glyma.17G197500	9.295	20.296	21.862	12.516	14.256	7.256	10.077	17.019	18.974
GmWRKY13-1	Glyma.06G147500	0.264	1.013	0.709	1.347	1.693	0.804	1.003	1.387	4.957
GmWRKY13-2	Glyma.04G218400	0.066	0.590	1.844	3.774	1.525	0.519	0.628	0.804	***
GmWRKY13-3	Glyma.08G143400	0.070	1.617	0.879	2.130	0.240	0.309	0.198	2.380	7.000
GmWRKY13-4	Glyma.05G185400	0	0	0	0	0	0	0	0	0
GmWRKY14-1	Glyma.15G135600	1.082	4.796	0.008	1.167	0.329	0.012	0.029	5.637	0.595
GmWRKY14-2	Glyma.09G029800	0.218	4.381	0.026	0.718	0.26	0.017	0.061	5.760	1.421
GmWRKY15-1	Glyma.05G096500	12.552	18.815	47.960	21.294	12.119	4.440	3.519	17.912	12.6006
GmWRKY15-2	Glyma.17G168900	17.894	19.667	39.365	24.886	31.530	5.464	3.539	18.552	10.305
GmWRKY15-3	Glyma.01G189100	6.244	8.360	5.248	7.644	10.277	2.928	4.327	8.962	8.133
GmWRKY15-4	Glyma.11G053100	4.636	4.453	3.615	7.692	9.597	3.637	0.983	3.736	8.462
GmWRKY18	Glyma.10G113800	0	0.210	0.131	1.432	5.17	0.271	0	0.123	0.250
GmWRKY20-1	Glyma.18G081200	2.940	3.178	2.577	4.282	0.888	4.519	2.716	2.999	3.852
GmWRKY20-2	Glyma.08G325800	4.922	7.145	5.155	1.489	8.839	6.441	5.093	5.969	4.458
GmWRKY20-3	Glyma.06G2242200									
GmWRKY20-4	Glyma.02G297400	5.748	5.098	2.346	5.422	0.923	3.938	4.689	3.710	4.893
GmWRKY20-5	Glyma.14G016200	1.639	7.049	7.566	9.558	1.952	6.730	4.130	6.739	7.430
GmWRKY21-1	Glyma.03G159700	0.462	0.705	0.496	0.384	0.254	1.101	****	1.049	1.045
GmWRKY21-2	Glyma.10G032900	2.919	6.311	6.446	15.912	7.046	****	11.200	5.047	12.305
GmWRKY21-3	Glyma.02G141000	4.482	8.565	18.932	47.276	36.608	****	31.762	13.682	18.442
GmWRKY22-1	Glyma.16G031900	12.020	2.094	1.585	0.963	16.380	0.241	0.577	2.443	0.768
GmWRKY22-2	Glyma.09G254800	11.254	3.777	3.833	3.111	2.832	1.175	2.143	3.918	2.172
GmWRKY22-3	Glyma.18G238200	24.601	3.595	7.939	4.965	2.345	1.444	1.715	4.179	3.379
GmWRKY23-1	Glyma.02G010900	5.435	8.800	10.018	11.025	14.490	2.998	0.280	18.424	3.171
GmWRKY23-2	Glyma.10G011300	4.326	5.582	11.089	14.324	7.169	5.954	2.046	****	5.904
GmWRKY23-3	Glyma.19G217800	4.186	1.202	3.119	0.940	3.905	2.653	3.382	1.290	0.597
GmWRKY23-4	Glyma.03G220800	21.360	6.907	10.350	8.666	21.210	10.417	3.279	12.449	3.690

GmWRKY23-5	Glyma.07G238000	0.673	1.087	0.503	1.335	0.909	0.275	0.189	1.361	0.219
GmWRKY26-1	Glyma.11G163300	22.526	6.408	27.415	0.589	15.298	0.124	0.256	7.406	0.178
GmWRKY26-2	Glyma.02G232600	31.105	11.734	80.957	3.869	67.456	1.268	0.254	12.355	2.755
GmWRKY26-3	Glyma.14G200200	22.057	9.232	72.911	4.009	51.836	1.592	0.688	7.450	2.556
GmWRKY27-1	Glyma.16G177000	0.923	0.316	0.248	0.053	0.085	0.233	0.071	0.303	0.055
GmWRKY27-2	Glyma.16G176700									
GmWRKY27-3	Glyma.20G163200	0.845	1.208	1.036	0.903	0.214	0.059	0.042	1.401	0.168
GmWRKY27-4	Glyma.10G230200	0.238	0.468	0.200	0.167	0.059	0.038	0.012	0.212	0.028
GmWRKY29-1	Glyma.11G021200	0.283	0	0.107	0.020	0	0	0	0.042	0
GmWRKY29-2	Glyma.08G018300	8.979	1.029	4.637	0.505	3.301	0.814	0.030	1.763	0.971
GmWRKY30-1	Glyma.06G125600	0.719	0.350	9.889	0.012	0.423	0.040	0	0.558	0.036
GmWRKY30-2	Glyma.04G238300	0.068	0.104	3.597	0.023	0.093	0.006	0.010	0.036	0
GmWRKY32-1	Glyma.12G152600	2.950	3.383	5.081	4.817	3.794	4.776	****	3.835	5.529
GmWRKY32-2	Glyma.17G074000	4.567	4.449	5.329	7.376	6.120	5.522	****	5.617	6.219
GmWRKY32-3	Glyma.02G203800	3.447	8.510	9.890	10.742	8.064	9.191	****	9.471	11.858
GmWRKY33-1	Glyma.01G128100	4.567	0.631	15.595	0.137	0.551	0.045	0.551	0.529	0.285
GmWRKY33-2	Glyma.09G280200	****	1.564	7.791	0.597	3.748	0.044	0.109	****	0.291
GmWRKY33-3	Glyma.18G208800	****	1.313	8.042	0.276	3.48	0.067	0.084	0.597	0.646
GmWRKY35-1	Glyma.08G078700	****	0.200	0.148	0.224	0.791	0.019	0.010	2.075	0.427
GmWRKY35-2	Glyma.05G123600	****	1.956	0.712	0.387	1.383	0.014	0.094	1.923	0.439
GmWRKY35-3	Glyma.03G220100	3.854	4.620	13.297	13.521	15.324	4.793	0.994	8.035	11.878
GmWRKY35-4	Glyma.19G217000	1.222	2.560	3.972	2.285	2.562	4.001	0.049	7.968	4.900
GmWRKY40-1	Glyma.14G102900	0.094	0.058	*****	0.064	1.897	0.167	0	0.066	0.320
GmWRKY40-2	Glyma.17G222500	0.137	0.094	1.730	1.349	1.381	0.425	0	0.307	1.515
GmWRKY40-3	Glyma06g06530									
GmWRKY40-4	Glyma.04G061400	0.712	0.219	4.250	0.058	0.112	0	0	0.236	0
GmWRKY40-5	Glyma.08G218600	3.547	1.877	16.461	0.759	4.719	0.285	0.921	4.147	0.048
GmWRKY40-6	Glyma.07G023300	2.090	1.444	27.883	0.655	10.670	0.045	0.325	2.533	0.182
GmWRKY40-7	Glyma.13G370100	2.146	0.366	10.267	0.211	4.934	1.137	3.078	0.339	2.433
GmWRKY40-8	Glyma.15G003300	12.795	3.064	27.064	1.683	12.293	1.844	0.501	3.748	0.829
GmWRKY40-9	Glyma.14G103100	2.181	0.902	****	0.094	3.219	0.251	0	0.792	0.132
GmWRKY40-10	Glyma.17G222300	0.869	0.482	4.179	0.088	0.840	0.890	0	0.208	0.720
GmWRKY41-1	Glyma.08G021900	1.364	0.666	37.304	1.727	7.809	0.160	0	0.533	0.636
GmWRKY41-2	Glyma.01G224800	6.124	3.699	32.819	0.247	0.826	0.089	0.022	1.484	0.124

GmWRKY41-3	Glyma.05G215900	5.900	2.871	72.788	2.229	4.983	0.853	0.010	2.178	0.426
GmWRKY41-4	Glyma.19G254800	0.917	0.718	****	1.756	6.286	0.914	0.782	0.619	1.110
GmWRKY41-5	Glyma.03G256700	2.359	1.351	15.234	1.788	2.39	1.072	0.743	1.672	2.360
GmWRKY41-6	Glyma.16G026400	2.005	1.980	****	2.309	4.302	1.905	0.507	0.680	4.402
GmWRKY41-7	Glyma.07G057400	12.204	3.527	44.517	2.200	3.021	1.715	0.234	3.082	7.275
GmWRKY42	Glyma.15G186300	4.219	2.233	0.793	0.060	0.116	0.013	0.011	2.939	0.012
GmWRKY44-1	Glyma.03G176600	3.788	1.543	0.030	2.186	1.081	6.777	6.912	1.204	3.755
GmWRKY44-2	Glyma.19G177400	0.510	0.397	0.116	1.019	5.689	17.882	9.595	0.078	2.236
GmWRKY47-1	Glyma.02G007500	*****	0.660	0	0.173	0.080	0.570	0	0.839	0.198
GmWRKY47-2	Glyma.03G224700	*****	6.393	4.763	0.284	1.918	7.240	0.145	6.284	0.974
GmWRKY47-3	Glyma.19G221700	*****	2.652	1.856	0.447	0.425	0.589	0.197	2.209	0.567
GmWRKY48-1	Glyma.15G139000	0.678	0.310	1.550	0.966	0.256	0.355	0.096	0.254	*****
GmWRKY48-2	Glyma.09G034300	0.202	0.141	0.145	1.948	1.326	****	0.273	0.202	1.163
GmWRKY49	Glyma.14G199800	2.624	0.905	1.409	2.760	2.936	1.458	1.564	1.461	1.058
GmWRKY50-1	Glyma.04G054200	0	0.338	****	0.021	0.326	0.129	0	0.022	0
GmWRKY50-2	Glyma.06G054500	0	0.042	0	0	0	0	0	0	0
GmWRKY51-1	Glyma.06G147100	0.083	0.945	****	0.214	0.991	0.071	0	2.013	0.127
GmWRKY51-2	Glyma.04G218700	0.094	0.107	****	0.024	1.895	0.013	0	0.050	0.024
GmWRKY51-3	Glyma.08G142400	7.102	6.607	****	2.082	13.941	2.739	0.110	3.325	0.621
GmWRKY51-4	Glyma.05G184500	4.607	3.793	****	0.599	3.262	0.468	0.022	3.440	0.630
GmWRKY54-1	Glyma.14G186000	0	0	0.213	0.346	2.195	****	0	0	0.020
GmWRKY54-2	Glyma.14G186100	0.124	0	0.133	0.260	0.048	0.260	0	0.022	0.260
GmWRKY55-1	Glyma.06G142100	0.287	0.128	****	0	0.009	0.016	0	0.137	0.015
GmWRKY55-2	Glyma.04G223200	0.024	0.033	1.874	0.025	0.057	0	0	0.052	0
GmWRKY55-3	Glyma.05G165800	0.709	0.516	****	2.098	1.785	1.563	1.747	1.120	1.852
GmWRKY56-1	Glyma.16G031400	0.567	****	0	0.430	0.434	0	0	0.744	0
GmWRKY56-2	Glyma.09G254400	2.909	2.351	0.023	0.699	0.339	0.012	0	2.376	0
GmWRKY56-3	Glyma.18G238600	1.143	1.176	0	0.350	0.287	0.051	0	0.891	0
GmWRKY57-1	Glyma.02G115200	****	6.655	5.936	4.655	6.927	3.632	0.830	9.706	0.469
GmWRKY57-2	Glyma.01G056800	1.856	2.714	4.168	6.934	1.093	5.082	0.271	0	0.975
GmWRKY65-1	Glyma.08G118200	105.342	35.199	3.432	0.461	0.843	0.607	0.412	39.186	0.655
GmWRKY65-2	Glyma.05G160800	****	21.478	16.324	0.856	0.895	1.325	1.452	25.525	0.560
GmWRKY67	Glyma.03G002300	0.974	0.788	0.165	0	0	0.017	0	0.486	0
GmWRKY69-1	Glyma.13G289400	****	15.301	11.764	4.415	11.662	1.519	0.941	17.523	1.047

GmWRKY69-2	Glyma.12G212300	****	8.590	14.376	3.244	8.011	1.536	0.542	8.642	0.816
GmWRKY70-1	Glyma.06G142000	14.003	3.271	****	1.701	3.014	1.208	0	3.348	5.414
GmWRKY70-2	Glyma.04G223300	10.994	2.424	****	3.616	3.010	2.246	0	2.248	12.887
GmWRKY70-3	Glyma.13G267500	****	0.844	0	0	0.015	0	0	2.020	0.046
GmWRKY70-4	Glyma.13G267600	0.202	0.091	0.109	0	0	0	0	0.297	0
GmWRKY70-5	Glyma.13G267700	0.737	0.463	0.043	0.063	0.013	0.127	0	1.037	0.021
GmWRKY70-6	Glyma.18G213200	12.520	14.208	****	7.469	61.053	4.574	0	9.891	16.468
GmWRKY70-7	Glyma.09G274000	1.274	13.618	****	17.662	63.153	23.080	0.230	13.878	35.173
GmWRKY71-1	Glyma.05G127600	1.698	2.059	4.683	0.777	1.577	0.339	0	2.925	4.088
GmWRKY71-2	Glyma.02G285900	****	1.638	0.442	1.802	4.982	0.637	0.024	3.740	2.077
GmWRKY71-3	Glyma.14G028900	11.267	1.363	0.669	3.804	7.746	1.800	0.667	2.867	3.804
GmWRKY72-1	Glyma.02G020300	****	0.075	0.012	0.011	0.006	0	0	1.517	0.075
GmWRKY72-2	Glyma.01G043300	****	0.174	0.012	0	0	0	0	1.944	0
GmWRKY72-3	Glyma.19G020600	29.001	4.910	0.051	0.024	0.160	0.339	0	14.498	0.466
GmWRKY72-4	Glyma.09G240000	2.946	0.399	0.009	0	0	0	0	7.005	0
GmWRKY72-5	Glyma.06G190800	3.422	1.771	4.871	0	0.068	0.039	0	1.539	0.044
GmWRKY72-6	Glyma.05G029000	5.345	3.643	1.801	0	0.037	0	0	3.099	0
GmWRKY75-1	Glyma.08G011300	0.118	0.161	7.643	0.213	0.189	0.133	0.621	0.156	0.060
GmWRKY75-2	Glyma.19G094100	7.910	1.782	17.779	0.636	46.028	1.538	0.064	3.081	1.493
GmWRKY75-3	Glyma.16G054400	11.090	1.889	6.339	0.682	3.741	0.400	0	2.684	1.148

		Phaseolus vulgaris									
		Young Trifoliates	Leaves	Flowers	Flower buds	Young Pods	Stems_ 10	Stems_ 19	Roots_ 10	Roots_ 19	Nodules
PvulWRKY1-1	Phvul.008G286100	9.252	9.045	9.436	6.845	7.967	5.765	8.569	14.380	18.247	19.422
PvulWRKY1-2	Phvul.008G270500	0.151	0	0.318	0.089	0.044	0	0.097	0.027	0.154	0.037
PvulWRKY1-3	Phvul.005G116000	2.086	1.243	1.249	0.567	1.204	4.299	3.701	13.254	9.570	5.510
PvulWRKY1-4	Phvul.011G101900	1.947	1.845	3.814	8.801	0.868	0.573	1.171	1.721	1.697	1.212
PvulWRKY1-5	Phvul.006G147800	5.640	3.980	35.715	2.336	11.086	5.769	4.996	30.353	39.480	29.518
PvulWRKY1-6	Phvul.006G047300	0.052	0.419	0.226	0.0780	0.037	0.321	0.190	5.599	4.205	1.984
PvulWRKY2	Phvul.008G054100	1.561	1.728	0.658	0.846	1.225	2.243	2.302	1.489	1.318	1.736
PvulWRKY3	Phvul.002G103400	5.245	11.979	6.520	1.907	1.293	2.691	2.078	11.98	8.041	4.142
PvulWRKY4	Phvul.002G091100	8.280	9.465	6.719	8.912	6.444	4.656	5.637	15.341	11.156	6.411
PvulWRKY5-1	Phvul.003G124000	0.446	0	0.235	0	0.080	0	0.211	0.069	0.155	0.157
PvulWRKY5-2	Phvul.009G220700	0.496	2.482	0.124	0.139	0.180	0.208	0.453	7.012	8.621	4.665
PvulWRKY5-3	Phvul.008G048100	0.529	0.499	0.325	0	0.484	9.359	16.859	0.407	1.360	0.371
PvulWRKY7	Phvul.001G213600	14.371	19.048	24.284	7.148	7.002	16.799	12.470	29.690	21.775	12.858
PvulWRKY9-1	Phvul.002G202500	1.751	1.052	0.283	1.104	0.391	0.124	0.349	4.926	2.941	2.199
PvulWRKY9-2	Phvul.008G043000	0	0	0.035	0	0	0	0	8.728	15.118	48.761
PvulWRKY11-1	Phvul.009G233900	11.660	8.298	34.141	13.189	24.192	58.075	42.105	26.672	52.750	36.555
PvulWRKY11-2	Phvul.003G139500	21.790	25.292	47.243	50.119	29.453	59.685	49.450	118.27	134.816	79.023
PvulWRKY11-3	Phvul.009G101900	6.223	4.460	7.366	3.787	7.057	10.617	12.083	55.647	42.011	39.200
PvulWRKY13-1	Phvul.009G138600	1.286	1.210	2.057	1.501	1.511	5.186	7.236	1.289	7.236	1.112
PvulWRKY13-2	Phvul.002G266400	0.984	0.759	1.053	1.014	4.036	4.905	9.200	1.068	2.084	0.686
PvulWRKY14	Phvul.006G123000	0.015	0	2.951	2.773	0.429	1.757	2.233	14.661	6.906	3.885
PvulWRKY15-1	Phvul.002G016100	18.590	7.058	12.420	13.027	25.399	33.747	33.331	24.014	29.599	19.726
PvulWRKY15-2	Phvul.003G240900	24.308	13.538	49.015	8.077	41.848	45.011	33.948	50.566	40.304	22.438
PvulWRKY17	Phvul.001G060200	37.519	14.965	66.844	46.894	58.155	61.321	59.206	82.313	68.979	31.322
PvulWRKY19-1	Phvul.008G081800	24.328	16.433	78.755	23.965	38.820	16.646	31.687	2.498	7.812	5.340

PvulWRKY19-2	Phvul.010G057900	1.036	0	0.775	0.565	0.363	0.383	3.340	0.255	0.283	0.336
PvulWRKY18	Phvul.009G087400	0.625	0.049	4.272	0.391	1.986	0.351	0.466	0.605	1.481	0.804
PvulWRKY20-1	Phvul.008G275300	12.309	11.580	3.236	4.211	5.947	8.457	12.566	9.648	13.512	8.168
PvulWRKY20-2	Phvul.006G053300	8.539	8.068	6.237	5.396	7.703	13.851	15.365	12.748	12.920	9.322
PvulWRKY21	Phvul.007G177900	11.453	10.762	16.279	7.975	20.533	25.918	30.637	18.302	23.445	10.298
PvulWRKY22-1	Phvul.010G104200	0.373	0.334	0.024	0.131	0.226	0.740	0.586	2.530	2.137	0.477
PvulWRKY22-2	Phvul.008G058300	4.873	3.911	7.905	7.629	10.299	11.860	14.664	13.807	18.148	6.667
PvulWRKY23-1	Phvul.007G212900	6.732	19.076	37.831	8.541	25.214	13.379	15.331	10.812	24.161	24.955
PvulWRKY23-2	Phvul.001G214400	2.785	1.006	12.746	1.259	14.023	2.097	3.127	5.000	7.483	21.158
PvulWRKY26-1	Phvul.008G251300	13.673	25.724	76.970	5.563	22.151	19.168	14.650	31.800	44.872	27.493
PvulWRKY26-2	Phvul.006G074600	0.090	0.030	4.098	0.602	1.149	0.214	0.291	1.346	7.173	5.947
PvulWRKY27-1	Phvul.004G105800	0	0.132	0.026	0.043	0.049	0.496	0.209	2.798	0.855	0.956
PvulWRKY27-2	Phvul.007G075400	0.773	2.191	0.953	1.525	1.739	4.015	2.247	4.904	3.037	2.140
PvulWRKY28	Phvul.001G042100	1.881	0.191	7.982	1.709	1.616	0.206	6.328	0.137	1.029	3.722
PvulWRKY29-1	Phvul.002G160100	0	0	0.383	0	1.091	0	0	0	0	0
PvulWRKY29-2	Phvul.002G293200	2.540	1.077	0.740	0.206	0.568	0.132	0.858	6.351	7.557	4.118
PvulWRKY32-1	Phvul.003G156300	10.843	6.257	9.099	7.352	18.647	17.133	21.690	14.537	13.683	8.762
PvulWRKY32-2	Phvul.009G195200	1.232	0.433	1.551	1.723	3.634	2.314	3.616	2.314	2.073	0.557
PvulWRKY33-1	Phvul.010G062500	0.456	0.054	0.321	0.063	0.135	0.028	0.137	0.376	3.133	1.964
PvulWRKY33-2	Phvul.008G090300	2.291	0.653	8.266	1.228	7.966	2.338	2.483	6.432	2.483	8.648
PvulWRKY33-3	Phvul.008G251700	1.203	0.633	1.474	0.707	0.743	0.485	0.928	0.626	0.928	0.593
PvulWRKY33-4	Phvul.008G251800	1.323	1.150	1.114	1.352	1.141	1.716	1.268	2.017	2.658	3.180
PvulWRKY34	Phvul.005G005800	10.543	18.7	9.276	4.281	9.776	15.288	14.963	12.995	17.563	9.053
PvulWRKY35	Phvul.002G202000	0	0	0.702	1.269	0.402	1.662	1.694	2.831	2.336	2.590
PvulWRKY40-1	Phvul.005G181800	3.510	1.450	15.359	6.014	3.403	3.169	5.093	6.442	19.924	9.181
PvulWRKY40-2	Phvul.010G161100	2.268	0.182	7.485	1.687	3.108	0.866	2.420	2.539	6.169	4.152
PvulWRKY43-1	Phvul.010G104700	0	0	0.263	0	0.354	0.096	0.390	1.408	2.232	1.765
PvulWRKY43-2	Phvul.008G058000	0.131	0.495	0.105	0	0.110	0	0.263	1.065	3.232	1.931
PvulWRKY44	Phvul.001G173700	5.855	2.206	17.610	19.068	13.720	2.122	3.545	0.579	0.461	0.615
PvulWRKY45-1	Phvul.008G185800	2.802	0.070	8.899	5.160	7.610	0.069	0.375	0	0	0
PvulWRKY45-2	Phvul.008G185700	2.015	0.075	1.181	0.742	5.393	0.307	0.679	0	0	0
PvulWRKY45-3	Phvul.009G043100	10.774	2.145	8.912	8.229	10.296	1.913	9.435	1.040	2.278	1.850
PvulWRKY46-1	Phvul.006G111700	2.231	2.157	10.648	2.256	2.488	1.910	2.786	0.819	4.546	2.844
PvulWRKY46-2	Phvul.010G111900	7.074	12.286	5.549	4.205	5.973	4.610	8.227	5.557	16.908	11.148

PvulWRKY47-1	Phvul.001G218500	7.537	2.307	17.958	4.058	3.476	2.023	3.232	14.132	13.676	7.112
PvulWRKY47-2	Phvul.007G209000	0	0	0.508	0.130	0.795	0.079	0	0.582	4.016	9.313
PvulWRKY48-1	Phvul.003G116300	1.052	0.213	6.642	0.938	0.967	4.263	4.065	2.420	6.170	6.174
PvulWRKY48-2	Phvul.006G119100	0.929	0.296	1.678	1.745	1.850	1.137	2.829	0.586	2.138	2.707
PvulWRKY49	Phvul.008G119400	1.251	0.156	0.358	2.289	0.443	0.214	0.288	2.424	0.770	0.943
PvulWRKY50	Phvul.002G265400	1.662	0.947	9.834	2.798	3.569	0.905	2.859	5.044	7.523	12.968
PvulWRKY51-1	Phvul.009G080000	0	0	0.047	0	0	0	0	0.444	0.302	0.072
PvulWRKY51-2	Phvul.001G039900	0.489	0.333	1.346	1.489	2.655	0.340	2.191	0.077	1.067	0.757
PvulWRKY58	Phvul.008G039900	15.678	18.863	50.788	12.467	20.225	16.703	14.476	19.028	18.364	13.387
PvulWRKY59-1	Phvul.007G118200	0	0	0	0	0	0	0	0	0	0
PvulWRKY59-2	Phvul.007G118100	0	0	0	0	0.180	0	0	0	0	0
PvulWRKY60	Phvul.001G042200	1.505	0.051	4.215	3.181	2.596	0.220	2.535	0.267	0.761	1.602
PvulWRKY65	Phvul.002G240900	0.978	1.286	2.192	0.919	1.014	5.706	1.641	53.960	32.464	17.091
PvulWRKY68	Phvul.009G138900	3.205	2.975	5.550	2.761	3.452	6.880	6.986	3.949	6.729	2.009
PvulWRKY69-1	Phvul.002G163400	0	0	0	0	0	0	0	0	0	0
PvulWRKY69-2	Phvul.002G297100	1.057	0.448	1.664	0.833	0.966	0.825	0.830	0.353	10.213	5.312
PvulWRKY69-3	Phvul.009G137500	0.484	0.928	0.608	0.088	0.224	0.248	0.518	0	1.349	1.046
PvulWRKY69-3	Phvul.005G093800	5.275	8.093	29.935	13.251	6.612	13.090	6.201	83.881	56.929	45.336
PvulWRKY71-1	Phvul.002G196800	11.706	0.865	3.826	0.705	2.911	2.264	5.378	0.614	1.772	2.247
PvulWRKY71-2	Phvul.008G192800	9.345	1.303	4.534	0.518	2.034	2.863	4.888	1.291	6.155	6.753
PvulWRKY73-1	Phvul.003G192000	0.034	0	0.414	0.051	0.125	0.071	0.027	24.059	20.516	5.466
PvulWRKY73-2	Phvul.009G189700	0	0	0	0.030	0	0.117	0	9.662	5.447	1.987
PvulWRKY73-3	Phvul.003G068700	0	0	0.132	0	0.030	0	0	0.150	1.254	1.666
PvulWRKY73-4	Phvul.002G089700	0	0	0	0.056	0.027	0	0.062	0.034	0.354	0.134
PvulWRKY75	Phvul.001G088200	0.324	0.108	39.026	1.813	15.373	3.600	2.214	1.851	3.023	2.485
PvulWRKY79-1	Phvul.005G080200	0	0	0	0	0	0	0	2.622	0.876	0.303
PvulWRKY79-2	Phvul.005G080400	0	0.078	0.094	0.090	0.087	0	0.100	0.522	0.531	0.479
PvulWRKY79-3	Phvul.005G080300	1.039	0.065	0	0	0	0	0	2.450	1.792	1.516
PvulWRKY97	Phvul.002G081600	3.199	3.171	3.313	1.612	5.572	11.458	7.644	3.026	5.062	4.103

Supplementary Figure 1

```
      AcWRKY9   ---------- -FKSTSDQLQ QGG--QMSSP PNRKARVSVR ARCQ--DATM NDGCQ-WRKY GQKIAKGNPC PR-------- ----AYYRCT V--SPGCPVR KQVQRCQE-- -DMSILITTY EGTHNHPLPV
     CsaWRKY9   ---------- -FNSIQNKPQ RPELQAMAPP QNRKARVSVR ARCE--SATM NDGCQ-WRKY GQKIAKGNPC PR-------- ----AYYRCT V--APGCPVR KQVQRCLE-- -DMSILITTY EGTHNHPLPV
     AcWRKY42   GWVPN----K LSKLNP--PK GVDQAS-EAT M-RKARVSVR ARSE--APMI TDGCQ-WRKY GQKMAKGNPC PR-------- ----AYYRCT M--ATGCPVR KQVQRCAE-- -DRTILITTY EGNHNHPLPP
    CsaWRKY31   GWGPNHKTPR FNNSSN--SK PLDQST-EAT M-RKARVSVR ARSE--APMI SDGCQ-WRKY GQKMAKGNPC PR-------- ----AYYRCT M--AVGCPVR KQVQRCAE-- -DRTILITTY EGNHNHPLPP
      PaWRKY6   PESPDHDSWT ANKAQKVTNK CAIDQT-EAT I-RKARVSVR ARSE--APMI SDGCQ-WRKY GQKMAKGNPC PR-------- ----AYYRCT M--AVGCPVR KQVQRCAE-- -DRSVLVTTY EGSHNHPLPP
     AlWRKY42   G--------- -NGSENASNK VIEQAAAEAT M-RKARVSVR ARSE--ANML SDGCQ-WRKY GQKMAKGNPC PR-------- ----AYYRCT M--AVGCPVR KQVQRCAE-- -DRTILITTY EGNHNHPLPP
     ZmWRKY43   GW-------- --LSNKVAKF LPVKGPEPAT M-RKARVSVR ARSE-----I SDGCQ-WRKY GQKMAKGNPC PR-------- ----SYYRCT M--AAGCPVR KQVQRCAE-- -DTTVVVTTY EGNHNHPLPP
      AtWRKY6   K--------- -IQKVNSTTP TTFDQTAEAT M-RKARVSVR ARSE--APMI SDGCQ-WRKY GQKMAKGNPC PR-------- ----AYYRCT M--ATGCPVR KQVQRCAE-- -DRSILITTY EGNHNHPLPP
    BrWRKY6-2   K--------- -VQKVNNSSP TTFDQPGEAI M-RKARVSVR ARSE--APMI SDGCQ-WRKY GQKMAKGNPC PR-------- ----AYYRCT M--ATGCPVR KQV------- ---------- -----HPLPP
   LuWRKY42-1   GWTPNKLPKF EACASSSAEA EERKAENTLS MIKKARVSVR ARSE--AAMM NDGCQ-WRKY GQKMAKGNPC PR-------- ----AYYRCT M--ASGCPVR KQVQRCAE-- -DQSILITTY EGHHSHPLPP
     SiWRKY97   DDAAASPPCA TRKSPSAAGE GVDDEVQQQA --KKARVSVR VKCD--TPTM PDGCQ-WRKY GQKISKGNPC PR-------- ----AYYRCT V--APHCPVR KQVQRCAE-- -DTTILITTY EGQHNHQLPP
     EgWRKY47   P--------- -RLEPSKGGE EQ----LADM PFRKARVSVR ARSE--APSI SDGCQ-WRKY GQKMAKGNPC PR-------- ----AYYRCT M--AVGCPVR KQVQRCAD-- -DKAILITTY EGNHNHPLPP
      OsWRKY9   A--------- -AAAPARENG EQQAAAAAEL PCRKPRVSVR ARSE--APMI SDGCQ-WRKY GQKMAKGNPC PR-------- ----AYYRCT M--AIGCPVR KQVQRCAE-- -DKTVLITTY EGNHNHQLPP
      OsWRKY5   P--------- -AAEMA---- ---------- PCRKARVSVR ARSE--APMI SDGCQ-WRKY GQKMAKGNPC PR-------- ----AYYRCT M--ASQCPVR KQVQRCAE-- -DKSILITTY EGTHSHPLPP
      PhWRKY1   R--------- -QQQEA---- ---------- SMRKARVSVR ALSE--APII ADGCQ-WRKY GQKMAKGNPC PR-------- ----AYYRCT M--ANACPVR KQVQRCAE-- -DRSILITTY EGTHNHPLPP
   CsWRKY72-1   QEPTEIWSPS KINIKSKRSD DQDEEVFQKA QLKKARVSVR ARCD--TPTM NDGCQ-WRKY GQKIAKGNPC PR-------- ----AYYRCT I--SPTCPVR KQVQRWHE-- -DMSILITTY EGTHNHPLPI
   EgWRKY72-2   EA-AETWSPN KV--LKTMRS GGDDEVSQQN PVKKARVSVR ARCD--AATM NDGCQ-WRKY GQKIAKGNPC PR-------- ----AYYRCT V--APSCPVR KQVQRCAE-- -DMSILITTY EGTHNHPLPM
   PtWRKY72-1   EA-GETWPPS KI--IPKRN- GDHDEAAQQS QAKRARVCVR TRCE--TPTM NDGCQ-WRKY GQKISKGNPC PR-------- ----AYYRCT V--APLCPVR KQVQRCAE-- -DTSILITTY EGTHNHPLPV
   VvWRKY72-1   AA-GESWPPS KS--LKTMR- TGDDEISQQN PAKRCRVSVR ARCD--TPTM NDGCQ-WRKY GQKIAKGNPC PR-------- ----AYYRCT V--APSCPVR KQVQRFAE-- -DMSILITTY EGTHNHPLPY
     RcWRKY61   EEPTETWSPN KI--RKTTI- TPDDEAMQQN QIKKTRVSVR ARCD--TPTM NDGCQ-WRKY GQKIAKGNPC PR-------- ----AYYRCT A--SPTCPVR KQVQRCAK-- -DMSVLITTY EGTHNHPLPL
     AcWRKY31   PEHDTLGSWG KNKCPKKIEQ TKSIEQVPDA SLRKARVSVR ARSD--APMI SDGCQ-WRKY GQKMAKGNPC PR-------- ----AYYRCT M--SMGCPVR KQVQRCAE-- -DKTILTTTY EGNHNHPLPP
      AtWRKY9   N--------- ---------- ---------- --RKARVSVR ARCE--TATM NDGCQ-WRKY GQKTAKGNPC PR-------- ----AYYRCT V--APGCPVR KQVQRCLE-- -DMSILITTY EGTHNHPLPV
     PpWRKY23   E--------- -TSNSSSMEQ GQKMKGPKR- -PREPRYEFK TRSE--VDVI DDGYK-WRKY GQKPVKSSPH PR-------- ----NYYRCT ---TANCPVR KRVERSIE-- -DPGLIVTSY EGTHTHPKIN
   EgWRKY28-1   IQDEQSTGCH EDHGREE-KS KNGDK-SKKK -EKRPRQPRF AFLTKSEIDN LEDGYRWRKY GQKAVKNSAF PR-------- ----SYYRCT S---QGCMVK KRVERSFQ-- -DPSIVVTTY EGQHNHHSPA
    PvWRKY3-1   FHKDMMMCTG SKPPAPGKTT GGNNNNKKSG QKKRARQARF AFMTKSDVDH LEDGYRWRKY GQKAVKNSPF PR-------- ----SYYRCT N---SKCTVK KRVERSSD-- -DPSVVITTY EGQHCHHTVT
   GrWRKY51-1   SCW----EVP GCNSFAYQNY DDKGSANTED INGDGLRFKI AFRTKSDIEI MDDGYRWRKY GKKKVKNNPN PR-------- ----NYYQCS S---EGCKVK KRVERERE-- -DPRFVITTY VGKHNHETLS
```

Supplementary Figure 1

Multiple sequence alignment of a single WRKY domain containing (C-terminal) WRKY transcription factor. The sequence alignment shows the presence of conserved W-R-K-Y-G-Q-K, P-R-x-Y-Y-x-C-x_5-C, K-x-V and H-x-H domains. Red indicates a conserved consensus of 90% or more. Multiple sequence alignment was carried out using the Multalin software (http://multalin.toulouse.inra.fr/multalin/) with the default parameters.

Supplementary Fig. 2

```
  AcWRKY26  YNQYAQPT-- QTLRE----Q RRSDDGYNWR KYGQKQVKGS ENPRSYYKCT -FPNCPTKKK VERSLDG-QI TEIVYKGSHN HAKPQSTRRS SASS-QSIQ- AAVVPSSSEV SDHFGSHGNT QMDSVT---- -------TPE NSSI------ ---------- ---------S IGDEDLDRSS
  SlWRKY26  GNQY---N-- QSIRE----Q KRSDDGYNWR KYGQKQVKGS ENPRSYYKCT -YPNCPTKKK VERSLDG-QI TEIVYKGNHN HPKPQSTRRS SSST-ASSAF QSYNTQTNEI PDHQSYGSNG QMDSVA---- -------TPE NSSI------ ---------- ---------S FGDDDHEHTS
  BdWRKY70  YSTAPAGTGV RAQSG----R RSSDDGYNWR KYGQKQMKGS ENPRSYYKCS -APGCPTKKK VEQAPDG-HV TEIVYKGTHN HPKPLQNARR GSSS-GSGAL SSYAMQGAGG ASMNNEVPAA DALS-G---- -------TPE NSSA------ ---------- ---------S YGDDDANV--
 GmWREKY20- ASHVEVRGSG LSVAA----E KTSDDGYNWR KYGQKLVKGS EFPRSYYKCT -HPNCEVKKL FERSHDG-QI TEIVYKGTHD HPKPQPSCRY STGTVMSIQG ERSDKASMAG RDDKATAMYG QVSHAAEPNS -------TPE SSPV------ ---------- ---------A TNDDGLEGVA
 CsaWRKY33- YNR------- ---EQ----Q KSENDGYNWR KYGQKQVKGS ENPRSYYKCT -FPSCPTKKK VERSLDG-QI TEIVYKGTHN HAKPQPTRRS SNSG------ -VYDPSAAET GVLQ------ ---------- ---------E DCSV------ ---------- ---------E VGEEEFEPNS
  CrWRKY58  RNHHNNPANN SRSYNVVNVD KPADDGYNWR KYGQKPIKGC EYPRSYYKCT -HVNCPVKKK VERSSDG-QI TQIIYKGQHD HERPQ--NRR GGGSRDSV-- ---------- ---------- ---------- ---EAGDHIH FVVG------ ---------- ---------G AGHVMESSDD
   MgWRKY3  SSDISNPDQK SQPHNLA-VD KPSEDGYNWR KYGQKQVKGS EFPRSYYKCT -HSNCPVKKK VERSLKG-QI TEIIYKGQHN HPLPN--KRT KDNGNSNS-- ---------- ---------- ---------- ---VKEQVTH ---------- ---------- ---------- -EHVSEYSSD
  MeWRKY4-1 SSDFS-PDQR SQASSLV-VD KPTDDGYNWR KYGQKQVKGS ECPRSYYKCT -QPTCPVKKK VERSLDG-QV TEIIYKGQHN HQAPQP-KRA KDAG-SLNGN SSNQSKFELA SQFQSGNVNK SKD------- ---RKDQESS QATH------ ---------- ---------E LLSGTSDSEE
  PperWRKY3 SSDISHSDQR SQPSSFI-VD KPNDDGYNWR KYGQKQVKGS EFPRSYYKCT -HSNCPVKKK VERSIDG-QI TEIIYKGEHN HERPQS-KRA KDSG-NPIGN I--QANPDLA SQVHGGHLSK SK-------- ----KGQESS QATH------ ---------- ---------D HLSGTSDSEE
  MdWRKY58  SPEASHSDKK YQP-S-S-TD RPADDXYNWR KYGQKQVKGS EFPRSYYKCT -HMNCPVKKK VERSPNG-EI TEIIYKGQHN HEVPQPSKRP KD-G-DLNGP -----KPENG LQRRIGDSNR SSENVASYS- -RXEMDQEST QAAP------ ---------- ---------G QLPGENDNEE
  PvWRKY4-1 LTEFSHSERK YQPPA-A-VD KPTHDGYNWR KYGQKPIKGS EYPRSYYKCT -HLNCPVKKK VERSSDG-QI TEIIYKGQHN HDLPQPNKRS KDCN-DSNGS IHLQSKPEVG SQAQAGNAIK LTETLPAHS- -VIGRDQEST QADP------ ---------- ---------S EPPGPSDSEE
  MdWRKY3-2 TSEITHSDKK YQPSSLP-ID KPADDGYNWR KYGQKQVKGS EYPRSYYKCT -HLNCPVKKK VERAPDG-HI TEIIYKGQHN HEKPQPNRRV KENNSDLNGN ANVQPKSDSN SQGWFGNSNK ISEIVPDSSP PEPESDLTSN QGAI------ ---------- ---------R PRPGSSESEE
  PhWRKY82  CRQLADKPFS SEPKSPHLVN MVA-DGFNWR KYGQKQVKSS ENSRSYYRCT -SSGCSAKKK VEHCPDG-RV VEIIYRGAHN HEPPPKTRFA KEKVTPISVP SGGETLRLVN TEILESSTPT CK-------- ----SDHFAV SETC------ ---------- ---------E QQLFCSSDCE
 GrWRKY32-1 KANQQKSYNL KMVSVPVIVK TPVSDGYNWR KYGQKQVKSP KGSRSYYKCT -FSDCQAKKI ECSDDTG-HV IEVVNKGMHS HKPPRKNNFT RESKIVSSAA ---RASHNIQ PIRIFNDSDP STSS------ ----KESVPE TTVN------ ---------- ---------P ERKGLFSCGS
 PvulWRKY32 KVDKGSPSGG TTLSSVSVTR ASASDGYNWR KYGQKQVKSP TGSRSYYRCT -HSNCCAKKI KFCDHSG-HV IEIVYKSQHS HDPPHKIDST KECKFLPYSE PKMKCSVPKQ SSRVRNDSDP SSSP------ ----KEPLYE SPCS------ ---------- ---------A KRN-LENSSN
   AtWRKY1  SSGALSLQSG SEGNSPFIRE KVMEDGYNWR KYGQKLVKGN EFVRSYYRCT -HPNCKAKKQ LERSAGG-QV VDTVYFGEHD HPKPLAGAVP INQDKRSDVF TAVSKGEQRI DIVSLIYKLC IVS------- ------YDIM FVEKTSGSSV QTLRQTEPPK IHGGLHVSVI PPADDVKTDI
  BrWRKY2-1 EVEEGEGEEQ RGVDSMVGGG APAEDGYNWR KYGQKLVKGS EYPRSYYKCT -NPNCQVKKK VERSREG-HI TEIIYKGAHN HSKPPPNRRS GM-QLDGTEQ AEQQQQQQQR DNTQQQVGSN ENNVEEGN-- -----QSAQT GGQYESGDVI VVDASSTFSN DE-------D EDDRGTHGSA
   CsWRKY2  QDEEGDQRGS GDSMVAGAGG ASSEDGYNWR KYGQKQVKGS EYPRSYYKCT -HPNCQVKKK VERSLEG-HI TEIIYKGAHN HPKPPPNRRS AIGSNP--LD MQLDIHEQAG LQSGADGDPS WANTQKGTAG GT--PEWRHD TVEVTSAAPV GPEYCNQSSA LPAQNGTPYE SGDAVDASST
 RcWRKY34-2 QDEEGDQRAS AEYM-AGSGG TPSEDGYNWR KYGQKQVKGS EYPRSYYKCT -HPNCPVKKK VERSHEG-HI TEIIYKGAHN HPKPPPNRRS AIGSSNSIMD MQLDVPEQTG LQNGTENDPV WASQQKGTAT GT--PDWRHD NVEVTSSPSV GPEFGNHSSA VQALNGTNFE SGDAIDASST
  OsWRKY30  EENATNKN-- -EEYSSDLII TPAEDGYNWR KYGQKQVKNS EHPRSYYKCT -FTNCAVKK- VERSQDG-QI TEIVYKGSHN HPLPPSNRRP NVPFSH-FND LRDDHSEKFG SKSGQATATS WENAANGHL- ----QDVGSE VLTKLSASLT TTEHAEKSV- --------MD KQEAVDISST
  PvWRKY80  PAEEGDTRG- -DYHPAMAAT TPAEDGYSWR KYGQKQVKHS EYPRSYFKCT -HPNCQVKKK VERSHEG-HI TEIIYKGTHS HPKPAQSRRP GVPPVHPFVE ---GAQADAP DNPRPHTNTA EARQAWHNNA GV--KDLPGD GMDAASPPSV PGELCDSSAS MQIHD---AG GVDATSAATA
  EgWRKY2-1 RIDNAEEGDQ KGMYPSVGTV RTSEDGYNWR KYGQKQVKGS EFPRSYYKCT -QPNCQVKKK VERSLDG-QI TEIIYKGTHN HPKPQPIRCG LLRSSFSLNE MS-EMADGSG AANKVEGGSV WRNNQLGSKD VRLGPDWRSD GPEQTSSTSV LTELSGLLPT SQGKCAVAFE SAQTPELSST
  LuWRKY2-1 KIDGMHMEDQ KGTFMGTGGG RTSEDGYSWR KYGQKQVKGS EYPRSYYKCT -SLSCQVKKK IERSHDG-QI TEIIYKGAHN HPKTQSVRRA ---QPDWCHD VP-EIDDGNE SCVKTEAGSG QKNNEPGS-- -----DWRAE GMERTSSPSV VTELSDLLST GQGKPVVTVE SVGTPELSSA
  CpWRKY44  TAAQNLEEDQ KTSSVVVSSD RPSYDGYNWR KYGQKQVKGS EYPRSYYKCT -HPNCPVKKK VERSFDG-QI AEIVYKGEHN HPKPPPPKRS SSGTQGLVLA SEGSAQDGNI SLPNNQLNER TEGSEGRV-- ----ENQNEM GLSC--FQGR YPPAYEPTAA GT------IH VGTGNSENSL
  FvWRKY44  IVSQNMEEDA KNIPAAANSD RPSYDGYNWR KYGQKQVKGS EYPRSYYKCT -HPNCPVKKK VERSLDG-QI AEIVYKGEHN HSKPQPPKRS SSGTQGSGFA SDATGQDYNT RLWNSHLNEK NEGSEGRV-- ----EDQNEV GIPVHSYQSK NIVHYDPLAT GK------LN AGTATPDNSC
  AlWRKY26  MVTQSLPQLD VSKSEIMSRN KTSDDGYNWR KYGQKQVKGS ENPRSYFKCT -YPNCLTKKK VETSLVKGQI TEIVYKGSHN HPKPQSTKRS SSTT------ ---------- ---------- ---------- ---------- ---------- ---------- ---------- --AAAHQNSS

            541                                                                                                                                                                                    720
  AcWRKY26  QRS-RSGGDE F--------- ------DEDE PEAKKWKKES ENEVISA--- ---SGS--RT VREPRVVVQT TSDIDILDDG YRWRKYGQKV VKGNPNPRSY YKCTNAGCPV RKHVERASHD LRAVITTYEG KHNHDVPAAR GSGNHAVNRP LPDNSNHNNA TMPIRPSATT TQSNHNANNT
  SlWRKY26  QKSSRSRGDD L--------- ------DEEE PDSKRWKREN ESEGVSA--- --LGGS--RT VREPRVVVQT TSDIDILDDG YRWRKYGQKV VKGNPNPRSY YKCTSTGCPV RKHVERASQD IRSVITTYEG KHNHDVPAAR GSGNHSINRP MAPT------ ---IRPTVTS HQSNYQV--P
  BdWRKY70  -----NGGEE F--------- ------EVDE PESKRWR--G GGEGAMA--- --ICGN--RT VREPRVVVQT ISDIDILDDG YRWRKYGQKV VKGNPNPRSY YKCTMAGCPV RKHVERASQD LRAVVTTYEG KHNHDVPAAR GSAAAARYRA AMP------- ----MPMPMP QAASGYLQQG
 GmWREKY20- GFVSNRTNEE V--------- ------DNDD PFSKRRKMEL GNVDITP--- --VV----KP IREPRVVVQT LSEVDILDDG YRWRKYGQKV VKGNPNPRSY YKCTNAGCPV RKHVERASHD PKAVITTYEG KHNHDVPAAR NS-SHDMAVP AAAAGGQTRT KLEESDTISL DLGMGISSAA
 CsaWRKY33- PF---SNSIE D--------- ------NENE PEAKRWKGEN ENEGYCG--- ---GGS--RT VKEPRIVVQT TSEIDILPDG YRWRKYGQKV VKGNPNPRSY YKCTSLGCPV RKHVERAAND MRAVITTYEG KHNHEVPAAR GSGGGGYNTI NRP------- ----IPTNIP MALRPLSSVT
  CrWRKY58  SGYGKDHDDD N--------- ------NDDD DGDEDLPGSK IR-KIDG--- --VSTTH-RT VTEPKIIVQT RSEVDLLDDG YRWRKYGQKV VKGNPHPRSY YKCTTANCTV RKHVERASTD AKAVITTYEG KHNHDVPAAR NG------T- AAAGT----- ---SDQHRMR SMSGNNMQQN
   MgWRKY3  SEE-VGNAET R--------- ------NEDE PESKRRNIEV VQ-SSQQ--- --GGPSR-SA VTEPRIIVQT TSEVDLLDDG YRWRKYGQKI VKGNPYPRSY YKCTMSGCTV RKHVERAASD AKAVITTYEG KHNHDVPAAK TS------SH STANA----- ---SSQLRHQ NNPVINRPTV
  MeWRKY4-1 VGDTETGVDE N--------- ------DEDE PDPKRRN--- TDVKVAD--- --PASSH-RT LTESRIIVQT TSEVDLLDDG YRWRKYGQKV VKGNPYPRSY YKCTSAGCRV RKHVERAATD PRAVITTYEG KHNHDVPAAK GS------SH STANS----- ---NSSELRQ QNV--EK---
  PperWRKY3 VGDAETRVDE K--------- ------DEDQ PDPKRRN--- TEVR-AE--- --PASSH-RT VTEPRIIVQT TSEVDLLDDG YRWRKYGQKV VKGNPYPRSY YKCTFPACNV RKHVERASTD PKAVITTYEG KHNHDVPASK TG------SH YAANN----- ---NALNLRA VNAGTEK---
  MdWRKY58  LGDGESREEG K--------- ------AD-E PNAKRRN--- IDVGASE--- --VALPH-KT VTEPKIIVQT RSEVDLLDDG YRWRKYGQKV VKGNPHPRCY YKCTFAGCNV RKHVERASTD AKAVITTYEG KHNHDVPAAR NS------SH NTANN----- ---SASQLKP PASGASQHTL
  PvWRKY4-1 AGDAAVQEEE R--------- ------GDDE PNPKRRQCRQ VDVVTSE--- --ATLPH-KT VTEPKIIVQT RSEVDLLDDG YRWRKYGQKV VKGNPHPRSY YKCTSAGCNV RKHVERAAAD PKAVVTTYEG KHNHDVPAAR NS------SH NTANT----- ---SASQVKP QKVVTEKHPL
  MdWRKY3-2 VGNAENKEEG ---------- ------VDCE PNPKRRS--- IEPAVPE--- --VPPSQ-KT VTEPKIIVQT RSEVDLLDDG YRWRKYGQKV VKGNPHPRSY YKCTSAGCNV RKHVERASTD PKAVITTYEG KHNHDVPAAR NS------SH NTASS----- ---MPS--KP QALVPEKHPL
  PhWRKY82  GDAGNKSEDE H--------- ------PSAE PVPKRRV--- IETTAPN--- --LTPVL-RT VREQKIIVQA GK----MSDG YRWRKYGQKI VKGNPNPRSY YRCTHGGCPV RKHVEKAPDD VNNIVVTYEG KHNHDEPFRS NSIPVSAISP PVATI----- ---EQPNTST TTSDEKPPTI
 GrWRKY32-1 DGNGDVQVKE E--------- ------LTSE PETKKRQAFL MKKGNTV--- --CSDSVLKA RKKPRFVVHA AGDVGISGDG YRWRKYGQKM VKGNPNPRNY YRCTSAGCPV RKHVETAVDN TNAIIITYKG VHDHDMPVPK KRHGQPIAPL VAAAAP---- --ASMNNLQH KKTEGGQSQV
 PvulWRKY32 GEKGKVFLKE A--------- ------HVND PEPKKR---L NKGDDLT--- --CLDSAIKP GKKPKFVVHA AEDMGISSDG YRWRKYGQKL VKGSPHPRNY YRCTSAGCPV RKHVETAVDN TKALIITYKG VHDHDMPVPK KRHGPPSASL VAAAAP---- --ASMNNLQL KRTGLIQNQE
   AtWRKY1  SQSSRITGDN T--------- ----HKDYNS PTAKRRKKGG NIELSPV--- -------ERS TNDSRIVVHT QTLFDIVNDG YRWRKYGQKS VKGSPYPRSY YRCSSPGCPV RKHVERSSHD TKLLITTYEG KHDHDMPPGR VVTH-NNMLD SEVDDKEGDA NKTPQSSTLQ SITKDQHVED
  BrWRKY2-1 SLGYDGGGGD G--------- ----EGGEDE SESKRRKLEA YAIEMSG--- ------ATRA IREPRVVVQT TSGVDILDDG YRWRKYGQKV VKGNPNPRSY YKCTAPGCTV RKHVERASHD LKSVITTYEG KHDHEVPAAR NSSHGGGGGN GNSGVSAAQT NHYSEPPRGR YDRQLNQSQF
   CsWRKY2  F-SNDEDEDD QVTHC---SV G--FDGEEDE SESKRRKIEA YATEMSG--- ------ATRA IREPRVVVQT TSEVDILDDG YRWRKYGQKV VKGNPNPRSY YKCTSAGCTV RKHVERASHD LKSVITTYEG KHNHDVPAAR NSSHVNSGSS SAVAAHAAAA GQ--IHRPEP SQLHNSMARF
 RcWRKY34-2 F-SNDEDEDD RATHG---SV G--YDGEGDE SESKRRKIET YPTDIAG--- ------ATRA IREPRVVVQT TSEVDILDDG YRWRKYGQKV VKGNPNPRSY YKCTNAGCTV RKHVERASHD LKSVITTYEG KHNHDVPAAR NSSHVNSGSS NTVNTQGGTA GQTHVHRPEP AQVPNSMTRF
  OsWRKY30  L-SNEED--D RVTHRAPLSL G--FDANDDY VEHKRRKMDV YAATSTSTNA IDIGAVASRA IREPRVVVQT TSEVDILDDG YRWRKYGQKV VKGNPNPRSY YKCTHPGCSV RKHVERSSHD LKSVITTYEG KHNHEVPAAR NSGHPSSGSA AAPQATNGLL HR----RPEP AQGGGGGSLA
  PvWRKY80  G-SDEVDGED RVTHGSMSQG GGGADADGDE LESKRRKLES YTIDMSA--- ------ASRA VREPRVVIQT TSEVDILDDG YRWRKYGQKV VKGNPNPRSY YKCTHPGCSV RKHVERASHD LKSVITTYEG KHNHEVPAAR NSGGHPSAAT AAGAAAGRRP EHPSVHDGLM RHLAGGCGGA
  EgWRKY2-1 LASTDGDDED GATQG---SM SHGEDADDEE PESKRRKSEN SLSESNL--- ------GSRA VREPRVVIQI ESDIDILDDG YRWRKYGQKV VKGNPNPRSY YKCTSAGCSV RKHVERASDN LKYVITTYEG KHNHEVPAAR NSN-LPSGSG NLSPAAVGAQ ANMAIPRNSR TNKPEAQVQD
  LuWRKY2-1 VVSHE-DDDD GIIIG---GY T----DEEE SDSKRRKTES CMVEANM--- ------TSRA VRQPRVVVQI ESEIDILDDG YRWRKYGQKV VKGNPNPRSY YKCTSPGCTV RKHVERASHN LKYVITTYEG KHNHQVPVAK NGNTLSSNSC GLSSALSNLQ PGSALARGLS SLKQESQVQR
  CpWRKY44  GLSGECEEGS KVV------- ----EGDDDE PRSKRRKNEN QSNEAGL--- ------LGES VQDPHVIVHG STDSEILDDG FRWRKYGQKH VKGNPYPRSY YRCTSLKCNV RKHVERASDD PVAFITTYEG KHNHDMPMRN TTNPVASEPD S
  FvWRKY44  GVSGECEEGS KGV------- ----ETEDYE PRSKRRKSEN QSSEAGI--- ------SGEV MQEPRVVVQS SADTEITGDG FRWRKYGQKV VKGNPYPRSY YRCTSVKCSV RKHVERVSED PKAFITTYEG KHNHDMPLRT ANPGASSEKD PQAPPSSKEK P
  AlWRKY26  HGDGKDIG-- ---------- -------EDE ADAKRWKRE- ---------- --------EN VKEPRVVVQT TSDIDILDDG YRWRKYGQKV VKGNPNPRSY YKCTFTGCGV RKHVERAFQD PKSVITTYEG KHKHQIPTPK RGHTSGF
```

Multiple sequence alignment of double (N- and C-terminal) WRKY domain protein. The N-terminal region shows the presence of conserved D-G-Y-N-W-R-K-Y-G-Q-K, R-S-Y-Y-x-C-x_4-C-x_{22}-H-x-H domain and the C-terminal region shows the presence of conserved D-G-Y-R-W-R-K-Y-G-Q-K, R-S-Y-Y-x-C-x_4-C, V-R-K-H-V-E, and H-x-H domains. Red mark indicates conserved consensuses of 90% or more. Multiple sequence alignment was carried out using multalin software (http://multalin.toulouse.inra.fr/multalin/) with the default parameters.

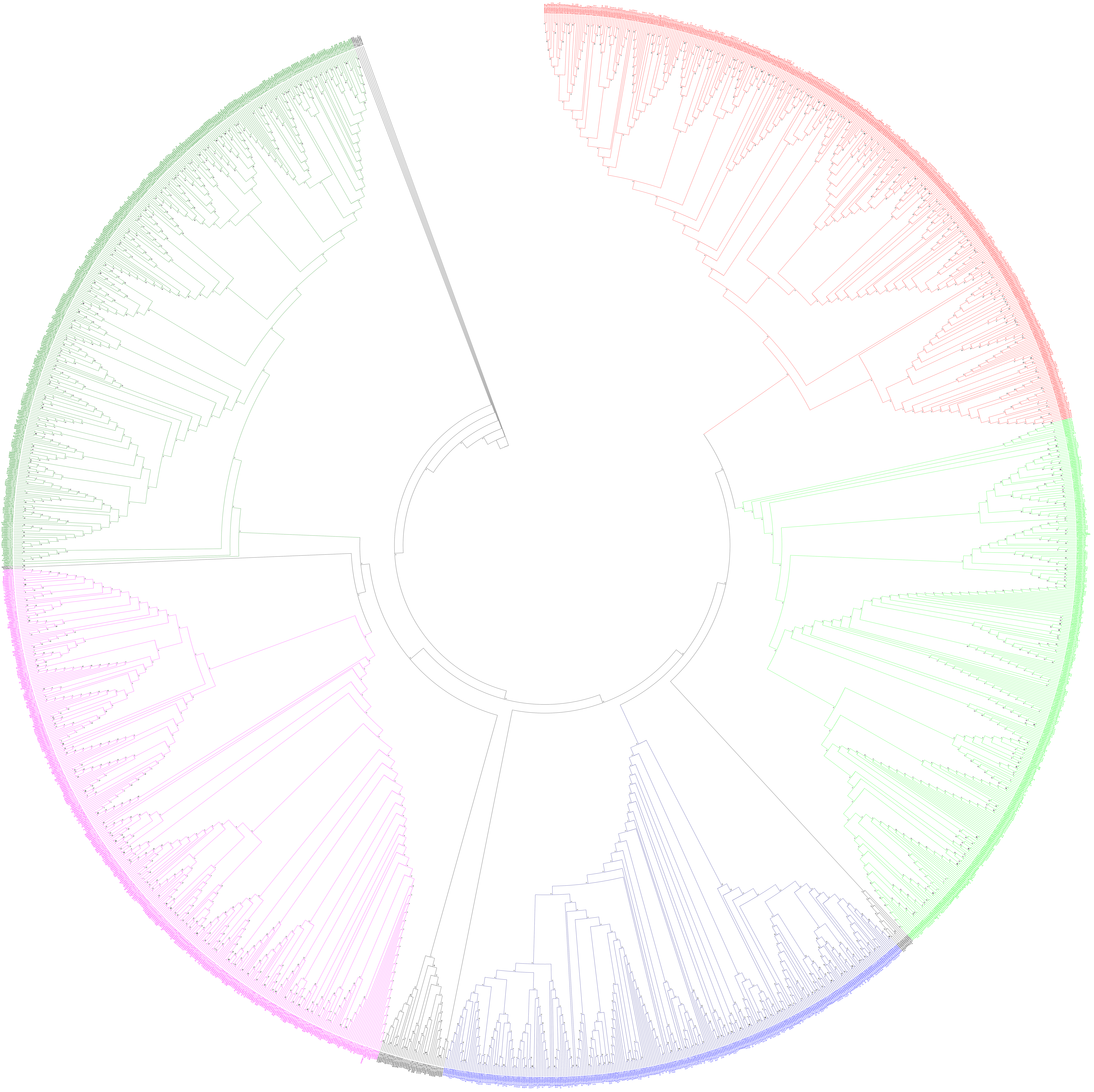

Supplement: Supplementary Information [file srep37309-s1.pdf]
